# Potential scalp stimulation targets for mental disorders: evidence from neuroimaging studies

**DOI:** 10.1186/s12967-021-02993-1

**Published:** 2021-08-10

**Authors:** Jin Cao, Thalia Celeste Chai-Zhang, Yiting Huang, Maya Nicole Eshel, Jian Kong

**Affiliations:** Department of Psychiatry, Massachusetts General Hospital, Harvard Medical School, Charlestown, MA 02129 USA

**Keywords:** Neuroimaging, Meta-analysis, Scalp stimulation, Scalp acupuncture, Transcranial electrical stimulation, Mental disorder

## Abstract

Mental disorders widely contribute to the modern global disease burden, creating a significant need for improvement of treatments. Scalp stimulation methods (such as scalp acupuncture and transcranial electrical stimulation) have shown promising results in relieving psychiatric symptoms. However, neuroimaging findings haven’t been well-integrated into scalp stimulation treatments. Identifying surface brain regions associated with mental disorders would expand target selection and the potential for these interventions as treatments for mental disorders. In this study, we performed large-scale meta-analyses separately on eight common mental disorders: attention deficit hyperactivity disorder, anxiety disorder, autism spectrum disorder, bipolar disorder, compulsive disorder, major depression, post-traumatic stress disorder and schizophrenia; utilizing modern neuroimaging literature to summarize disorder-associated surface brain regions, and proposed neuroimaging-based target protocols. We found that the medial frontal gyrus, the supplementary motor area, and the dorsal lateral prefrontal cortex are commonly involved in the pathophysiology of mental disorders. The target protocols we proposed may provide new brain targets for scalp stimulation in the treatment of mental disorders, and facilitate its clinical application.

**Supplementary Information:**

The online version contains supplementary material available at 10.1186/s12967-021-02993-1.

## Introduction

Mental disorders are a major component of the modern global disease burden. However, the quality of the therapeutic outcomes of pharmacologic treatments for mental disorders is ambiguous due to their side effects, withdrawal symptoms, and risk of abuse. Thus, increasing attention has been given towards non-pharmacological interventions over recent years.

Recently, scalp stimulation methods, i.e., applying transcranial stimulation on the scalp to modulate the function of corresponding brain areas to relive symptoms, have drawn increased attention of investigators. Scalp stimulation methods may include many different current treatments.

For instance, scalp acupuncture, a modern school of acupuncture developed on the basis of anatomical and neurophysiological knowledge [1], may be considered as an early scalp stimulation treatment. Scalp acupuncture aims to modulate certain brain areas, thereby providing therapeutic benefits for a wide scope of diseases through the stimulation of specific scalp areas corresponding to certain cortical areas. Scalp acupuncture may be applied by manual stimulation or electrical stimulation (similar to transcranial alternating current stimulation [tACS]). Accumulating evidence has demonstrated the potential of scalp acupuncture in relieving symptoms of mental disorders [2, 3].

Nevertheless, current prescriptions of scalp acupuncture for varying disorders are mainly based on an understanding of brain functions from the 1970s, when the practice was first introduced. In recent decades, there has been remarkable progress made in understanding the neural circuitry of mental disorders through cutting-edge brain imaging techniques [4–6]. Unfortunately, these advances have yet to be incorporated into scalp acupuncture treatments for mental disorders.

Modern brain stimulation methods such as transcranial electrical stimulation (tES) may also be considered scalp stimulation methods. For example, transcranial direct current stimulation (tDCS), a form of tES, is based on the application of a weak, direct electric current delivered over the scalp to induce polarity dependent changes in cortical excitability (anodal and cathodal stimulation induce increasing and decreasing cortical excitability, respectively), and has shown promising results in ameliorating the symptoms of mental disorders [7–9]. Another method, transcranial alternating current stimulation (tACS), a different form of tES, can modulate the rhythms of endogenous oscillations by applying weak alternating current through the scalp, and has also demonstrated potential in alleviating anxiety, depression, compulsive disorders, etc. [10–12].

Literature suggests that multiple brain regions/networks are involved in the pathophysiology of mental disorders. Yet as of current date, many tES studies have only used the prefrontal cortex as the target area for stimulation [13, 14]. Identifying other brain regions associated with mental disorders (particularly among surface brain areas which are suitable for tES) would expand target selection and may represent a crucial step to increasing the potential for neuromodulation techniques as treatment for mental disorders.

In recent decades, the development of brain neuroimaging techniques has spurred rapid growth of literature on human brain imaging studies of mental disorders and has significantly advanced our understanding of the complex brain pathophysiology associated with these disorders. However, the rich literature also introduces new challenges, i.e., we are now burdened with an excess of data to work through. It has therefore become necessary to develop new techniques for the large-scale aggregation and synthesis of human neuroimaging data [15].

Neurosynth is a new brain mapping framework that can incorporate text-mining, meta-analysis and machine-learning techniques to generate probabilistic mappings between mental disorders and neural states that can be used for a broad range of neuroimaging applications and generate large-scale meta-analyses for hundreds of broad psychological concepts. Previous approaches have relied heavily on researchers’ manual efforts or specific domains/focuses (for example: resting state functional connectivity, task related fMRI associated with a specific disorder), which may have limited the scope and efficiency of resulting analyses [15].

Thus, with the aid of Neurosynth, this study aims to develop neuroimaging-based target protocols for eight common mental disorders (attention deficit hyperactivity disorder (ADHD), anxiety disorder, autism spectrum disorder (ASD), bipolar disorder, compulsive disorder, major depression, post-traumatic stress disorder (PTSD) and schizophrenia) for tES, scalp acupuncture, repetitive transcranial magnetic stimulation (rTMS) [9, 14, 16], focused ultrasound (FUS) [17] and other brain stimulation methods when applicable. Specifically, we first used Neurosynth to generate large-scale meta-analyses for the above eight mental disorders. Then, we further refined/simplified the findings from the neuroimaging analyses, and proposed neuroimaging-based scalp stimulation target protocol for each disorder to facilitate its clinical application. We hypothesize that different mental disorders will be associated with distinguishable scalp stimulation targets, although there may be common/overlapping targets across different disorders.

## Methods

To identify disorder-associated brain regions, we used Neurosynth (http://neurosynth.org/:accessed21September2020) as a metadata reference for neuroimaging literature. In this study, we applied a Bayesian reverse inference term-based meta-analytic approach from Neurosynth that extracted data from all published neuroimaging studies included in the Neurosynth database. This method is different from the classic forward inference that selected voxels for inclusion in a given map based on their positive association with each mental disorder, and instead also included all negative findings thus allowing for greater specificity [15]. Under the search string “disorder name” (i.e., search strings “ADHD”, “anxiety disorder”, “autism spectrum”, “bipolar disorder”, “compulsive disorder”, “major depression”, “PTSD”, and “schizophrenia”), neuroimaging studies were identified, and a uniformity test map was generated to identify disorder-associated brain regions. Complete lists of the studies included for each disorder extracted from Neurosynth can be found in Additional file 1: Tables S1–S8.

Since scalp stimulations such as scalp acupuncture and tES will predominantly influence the surface brain areas, similar to our previous studies [18–21], a brain surface cortical mask was created using the SPM Wake Forest University (WFU) PickAtlas toolbox (http://fmri.wfubmc.edu/software/pickatlas, version 3.0.5) to identify disorder-associated surface brain regions [18–21]. Next, brain regions from the meta-analysis were refined by discerning the overlap of the uniformity test map with the brain surface cortical masks and then using the xjView toolbox (http://www.alivelearn.net/xjview) to identify the coordinates with peak z-scores within the all-surface cluster larger than 30 voxels on the uniformity test map (Additional file 1: Figure S1).

The results from the meta-analysis were mapped onto a standard brain using SurfIce (https://www.nitrc.org/projects/surfice) and a standard head using MRIcroGL (http://www.mccauslandcenter.sc.edu/mricrogl) with the international 10–20 electroencephalography (EEG) system in MNI space. The MNI coordinates of the 10–20 EEG system were extracted from a previous study [22] (Additional file 1: Figure S1).

To facilitate clinical application, we refined the disorder-associated surface clusters to eight/nine clusters (with the largest cluster size/peak intensity among all clusters) using xjView toolbox and identified peak coordinates of these clusters as the potential scalp stimulation targets for corresponding disorders. Furthermore, 2-mm radius spherical masks centered on the identified peak coordinates were created using WFU_PickAtlas toolbox and mapped onto a standard brain using SurfIce with the international 10–20 EEG system in MNI space for indicating the locations. In addition, we applied the international standard acupoints to facilitate identifying the locations. Finally, we visually checked the locations of the brain regions obtained to identify potential brain surface targets that are accessible by scalp acupuncture, tES, and other scalp stimulation methods, and proposed neuroimaging-based scalp stimulation target prescriptions (e.g., ADHD-1 to ADHD-9, ANX-1 to ANX-9, AUT-1 to AUT-9, etc.) for each disorder based on the neuroimaging findings. To help the readers understand the specific brain function of identified areas, we also summarized the brain functions of each identified surface region associated with a corresponding mental disorder (functions were collected and summarized based on https://neurosynth.org and http://www.fmriconsulting.com/brodmann) (Additional file 1: Figure S1).

Since mental disorders are conceived as an interconnected system of symptoms in which certain symptoms are the cause of others and can be dominant in specific brain regions; we also explored the overlap surface regions among the eight mental disorders using xjView toolbox. Results (with peak coordinates showing on each overlap region) were mapped onto a standard brain using MRIcroGL for demonstration.

## Results

### Meta-analysis results

#### A. ADHD

144 studies (extracted from Neurosynth) were included in the data analysis (a complete list of the 144 studies can be found in Additional file 1: Table S1). Twenty-one clusters on the brain surface were identified from the uniformity test map of the meta-analysis (Table 1).

**Table 1:** Coordinates of ADHD-associated surface regions identified from meta-analysis

Cluster ID	Cluster size	Peak T	Peak coordinates			Brain regions
			x	y	z	
1	105	6.98	− 2	50	− 10	L MedFG/OrbMFG
2	79	8.62	42	24	− 8	R IFG/OrbIFG
3	205	8.62	− 36	22	− 8	L IFG/OrbIFG
4	168	6.98	36	24	8	R IFG/TriIFG
5	39	4.51	58	− 44	8	R STG/MTG
6	39	4.51	− 40	46	10	L MFG/TriIFG
7	55	5.33	− 2	46	18	L MedFG/SupMFG/dlPFC/mPFC
8	35	5.33	46	36	24	R MFG/TriIFG
9	67	5.33	− 42	30	18	L MFG/TriIFG
10	66	5.33	− 58	− 50	28	L IPL/SMG
11	36	5.33	− 50	− 64	32	L IPL/AG
12	188	6.98	− 48	10	32	L IFG/dlPFC/mPFC/PreCG
13	99	6.15	58	− 46	30	R IPL/SMG
14	81	6.98	36	26	40	R MFG/dlPFC/mPFC
15	71	5.33	8	− 68	36	R SPL/PCu/cuneus
16	120	6.98	− 40	− 56	46	L IPL/PCu/AG
17	236	8.62	− 2	20	42	L MedFG/SupMFG/mPFC
18	71	5.33	48	− 46	46	R IPL/SMG
19	82	6.15	− 28	− 54	42	L SPL/IPL/PCu
20	36	5.33	− 8	− 6	52	L MedFG/SMA
21	31	5.33	− 42	− 26	52	L SPL/PoCG

L, left; R, right; MFG, middle frontal gyrus; IFG, inferior frontal gyrus; OrbIFG, orbital inferior frontal gyrus; SupMFG, superior medial frontal gyrus; MedFG, medial frontal gyrus; OrbMFG, orbital medial frontal gyrus; TriIFG, triangular inferior frontal gyrus; dlPFC, dorsolateral prefrontal cortex; mPFC, medial prefrontal cortex; STG, superior temporal gyrus; MTG, middle temporal gyrus; SPL, superior parietal lobule; IPL, inferior parietal lobule; SMG, supramarginal gyrus; AG, angular gyrus; PreCG, precentral gyrus; PoCG, postcentral gyrus; PCu, precuneus; SMA, supplementary motor area

**Fig. 1:**
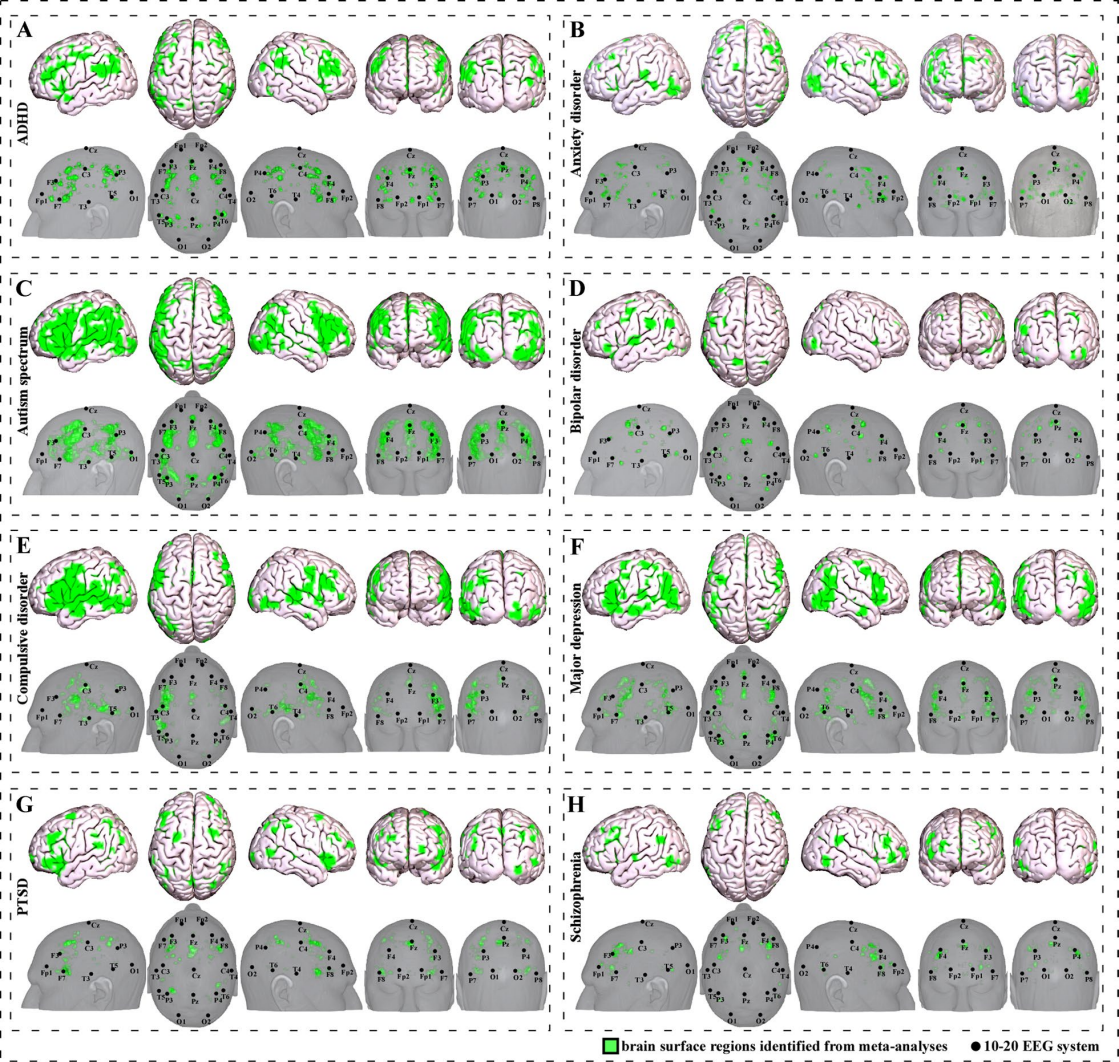
Brain surface regions for scalp stimulation for mental disorders, identified from meta-analyses of neuroimaging studies. **A** ADHD-associated surface regions. **B** Anxiety disorder-associated surface regions. **C** Autism spectrum-associated surface regions. **D** Bipolar disorder-associated surface regions. **E** Compulsive disorder-associated surface regions. **F** Major depression-associated surface regions. G. PTSD-associated surface regions. **H** Schizophrenia-associated surface regions

These brain regions were the bilateral middle frontal gyrus (MFG), inferior frontal gyrus (IFG), orbital inferior frontal gyrus (OrbIFG), triangular inferior frontal gyrus (TriIFG), dorsolateral prefrontal cortex (dlPFC), medial prefrontal cortex (mPFC), superior parietal lobule (SPL), inferior parietal lobule (IPL), supramarginal gyrus (SMG) and precuneus (PCu), the left medial frontal gyrus (MedFG), superior medial frontal gyrus (SupMFG), orbital medial frontal gyrus (OrbMFG), angular gyrus (AG), precentral gyrus (PreCG), postcentral gyrus (PoCG), supplementary motor area (SMA), and superior temporal gyrus (STG) and middle temporal gyrus (MTG) (Table 1, Fig. 1A).

#### B. Anxiety disorder

95 studies (extracted from Neurosynth) were included in the data analysis (a complete list of the 95 studies can be found in Additional file 1: Table S2). Fourteen clusters on the brain surface were identified from the uniformity test map of the meta-analysis (Table 2).

**Table 2:** Coordinates of anxiety-associated surface regions identified from meta-analysis

Cluster ID	Cluster size	Peak T	Peak coordinates			Brain regions
			x	y	z	
1	55	12.80	28	4	− 22	R STP
2	178	6.88	− 32	26	− 6	L IFG/OrbIFG
3	276	6.88	− 2	54	− 12	L MedFG/OrbMFG
4	55	6.88	− 44	− 74	− 8	L MOG/IOG
5	80	6.88	− 56	− 40	2	L MTG
6	30	7.87	46	10	4	R IFO
7	101	5.89	50	22	20	R IFG/dlPFC
8	61	5.89	− 6	56	28	L SFG/SupMFG
9	40	5.89	− 38	14	32	L MFG/IFO
10	47	5.89	56	− 46	34	R SPL/SMG/AG
11	53	5.89	42	0	46	R MFG/PreCG
12	112	7.86	2	18	44	R SMA
13	55	4.90	38	28	42	R MFG
14	51	4.90	24	− 66	44	R SPL/SOG

L, left; R, right; STP, superior temporal pole; IFG, inferior frontal gyrus; OrbIFG, orbital inferior frontal gyrus; MedFG, medial frontal gyrus; OrbMFG, orbital medial frontal gyrus; MOG, middle occipital gyrus; IOG, inferior occipital gyrus; MTG, middle temporal gyrus; IFO, inferior frontal operculum; dlPFC, dorsolateral prefrontal cortex; SFG, superior frontal gyrus; SupMFG, superior medial frontal gyrus; MFG, middle frontal gyrus; SPL, superior parietal lobule; SMG, supramarginal gyrus; AG, angular gyrus; PreCG, precentral gyrus; SMA, supplementary motor area; SOG, superior occipital gyrus

These surface regions included the left superior frontal gyrus (SFG)/superior medial frontal gyrus (SupMFG), middle temporal gyrus (MTG), and middle occipital gyrus (MOG)/inferior occipital gyrus (IOG); the right dorsolateral prefrontal cortex (dlPFC), supplementary motor area (SMA), superior temporal pole (STP), superior parietal lobule (SPL)/supramarginal gyrus (SMG)/angular gyrus (AG), precentral gyrus (PreCG), and superior occipital gyrus (SOG); as well as the bilateral inferior frontal gyrus (IFG), middle frontal gyrus (MFG), and inferior frontal operculum (IFO) (Table 2, Fig. 1B).

#### C. Autism spectrum

170 studies (extracted from Neurosynth) were included in the data analysis (a complete list of the 170 studies can be found in Additional file 1: Table S3). Nineteen clusters on the brain surface were identified from the uniformity test map of the meta-analysis (Table 3).

**Table 3:** Coordinates of autism spectrum disorder-associated surface regions identified from meta-analysis

Cluster ID	Cluster size	Peak T	Peak coordinates			Brain regions
			x	y	z	
1	125	5.97	− 56	− 6	− 16	L MTG/ITG/STG
2	109	5.97	58	− 6	− 16	R MTG/ITG/STG
3	2515	17.27	− 46	10	32	L IFG/TriIFG/OrbIFG/OperIFG/MFG/PreCG/STG/RO
4	74	7.03	44	− 54	− 16	R ITG/MOG
5	2334	11.62	− 30	− 56	40	L IPL/SPL/STG/MTG/PreCG/PoCG/RO/SMG/AG/MOG
6	2160	17.27	34	26	− 6	R IFG/OrbIFG/OperIFG/TriIFG/MFG/PreCG
7	467	7.38	0	54	24	SupMFG/MedFG/OrbMFG/
8	102	6.32	− 22	− 94	− 4	L IOG/MOG/cuneus
9	479	6.68	50	− 70	0	R MTG/STG/ITG/IPL/SMG/AG/MOG/IOG
10	153	5.62	62	− 46	− 8	R MTG/STG
11	164	5.97	− 38	50	4	L IFG/TriIFG/OrbIFG/MFG/SFG
12	100	6.68	− 10	− 84	4	L SOG/cuneus
13	614	13.03	− 2	− 56	26	L PCu/cuneus
14	71	7.03	50	− 14	10	R PreCG/PoCG/RO/SMG/STG
15	1070	16.21	0	14	48	SMA/MedFG/SupMFG
16	468	10.21	34	− 56	44	R IPL/SPL/PCu/SMG/AG/SOG
17	60	4.91	− 10	− 66	52	L SPL/PCu
18	36	5.97	− 28	28	48	L MFG/SFG
19	86	5.97	− 30	− 2	56	L MFG/SFG/PreCG

L, left; R, right; SFG, superior frontal gyrus; MFG, middle frontal gyrus; IFG, inferior frontal gyrus; OrbIFG, orbital inferior frontal gyrus; TriIFG, triangular inferior frontal gyrus; OperIFG, opercular inferior frontal gyrus; SupMFG, superior medial frontal gyrus; MedFG, medial frontal gyrus; OrbMFG, orbital medial frontal gyrus; STG, superior temporal gyrus; MTG, middle temporal gyrus; ITG, inferior temporal gyrus; RO, Rolandic operculum; SPL, superior parietal lobule; IPL, inferior parietal lobule; SMG, supramarginal gyrus; AG, angular gyrus; PreCG, precentral gyrus; PoCG, postcentral gyrus; PCu, precuneus; SMA, supplementary motor area; SOG, superior occipital gyrus; MOG, middle occipital gyrus; IOG, inferior occipital gyrus

These brain regions were the bilateral middle temporal gyrus (MTG), inferior temporal gyrus (ITG), superior temporal gyrus (STG), superior occipital gyrus (SOG), middle occipital gyrus (MOG), inferior occipital gyru (IOG), superior frontal gyrus (SFG), middle frontal gyrus (MFG), inferior frontal gyrus (IFG), orbital inferior frontal gyrus (OrbIFG), triangular inferior frontal gyrus (TriIFG), opercular inferior frontal gyrus (OperIFG), Rolandic operculum (RO), superior parietal lobule (SPL), inferior parietal lobule (IPL), supramarginal gyrus (SMG), angular gyrus (AG), precentral gyrus (PreCG), postcentral gyrus (PoCG) and precuneus (PCu), the left cuneus, as well as the right superior medial frontal gyrus (SupMFG), medial frontal gyrus (MedFG), orbital medial frontal gyrus (OrbMFG) and supplementary motor area (SMA) (Table 3, Fig. 1C).

#### D. Bipolar disorder

130 studies (extracted from Neurosynth) were included in the data analysis (a complete list of the 130 studies can be found in Additional file 1: Table S4). Eight clusters on the brain surface were identified from the uniformity test map of the meta-analysis (Table 4).

**Table 4:** Coordinates of bipolar disorder-associated surface regions identified from meta-analysis

Cluster ID	Cluster size	Peak T	Peak coordinates			Brain regions
			x	y	z	
1	55	7.29	− 40	18	− 20	L IFG/OrbIFG/STP
2	60	7.29	40	− 78	− 4	R MOG/IOG/ITG
3	37	7.29	− 58	− 14	6	L STG
4	38	5.84	30	40	24	R MFG/SFG
5	87	8.74	46	4	30	R IFG/OperIFG/MFG/PreCG
6	54	6.57	32	− 54	40	R IPL/SPL/SMG/AG
7	72	8.02	− 30	− 58	44	L IPL/SPL
8	205	8.02	− 4	8	52	L MedFG/SFG/SMA

L, left; R, right; SFG, superior frontal gyrus; MFG, middle frontal gyrus; IFG, inferior frontal gyrus; OrbIFG, orbital inferior frontal gyrus; OperIFG, opercular inferior frontal gyrus; MedFG, medial frontal gyrus; STG, superior temporal gyrus; ITG, inferior temporal gyrus; STP, superior temporal pole; SPL, superior parietal lobule; IPL, inferior parietal lobule; SMG, supramarginal gyrus; AG, angular gyrus; PreCG, precentral gyrus; SMA, supplementary motor area; MOG, middle occipital gyrus; IOG, inferior occipital gyrus

These brain regions were the bilateral inferior frontal gyrus (IFG), superior frontal gyrus (SFG), superior parietal lobule (SPL) and inferior parietal lobule (IPL), the left orbital inferior frontal gyrus (OrbIFG), superior temporal pole (STP), medial frontal gyrus (MedFG), superior temporal gyrus (STG) and supplementary motor area (SMA), as well as the right middle frontal gyrus (MFG), middle occipital gyrus (MOG), inferior occipital gyrus (IOG), inferior temporal gyrus (ITG), opercular inferior frontal gyrus (OperIFG), SMG, supramarginal gyrus (SMG), angular gyrus (AG) and precentral gyrus (PreCG) (Table 4, Fig. 1D).

#### E. Compulsive disorder

92 studies (extracted from Neurosynth) were included in the data analysis (a complete list of the 92 studies can be found in Additional file 1: Table S5). Seventeen clusters on the brain surface were identified from the uniformity test map of the meta-analysis (Table 5).

**Table 5:** Coordinates of compulsive disorder -associated surface regions identified from meta-analysis

Cluster ID	Cluster size	Peak T	Peak coordinates			Brain regions
			x	y	z	
1	153	7.62	− 54	− 2	− 16	L MTG/STG/ITG/STP
2	67	5.86	46	− 76	− 8	R MOG/IOG/MTG/ITG
3	31	5.87	− 38	32	− 10	L IFG/OrbIFG/MFG
4	846	9.38	− 64	− 34	4	L MTG/STG/ITG/IPL/SMG/MOG/IOG
5	38	6.75	38	24	− 8	R IFG/OrbIFG
6	39	5.87	− 34	26	− 8	L IFG/OrbIFG/TriIFG
7	33	5.87	22	− 92	− 6	R IOG/MOG/cuneus
8	1544	10.26	− 54	10	18	L IFG/TriIFG/OperIFG/MFG/PreCG/PoCG/RO
9	32	5.87	− 36	− 86	− 6	L MOG/IOG
10	285	7.62	62	− 34	0	R STG/MTG
11	149	6.75	− 62	− 14	6	L STG/PreCG/PoCG/RO
12	54	6.75	60	− 4	16	R PreCG/PoCG/RO
13	34	4.99	50	6	20	R IFG/OperIFG/TriIFG/PreCG
14	79	6.75	54	28	22	R MFG/IFG/TriIFG
15	66	5.87	48	− 4	44	R MFG/PreCG/PoCG
16	320	7.62	− 2	14	52	L MedFG/SupMFG/SFG/SMA
17	71	6.75	− 30	− 72	44	L SPL/IPL/PCu/MOG/AG

L, left; R, right; SFG, superior frontal gyrus; MFG, middle frontal gyrus; IFG, inferior frontal gyrus; OrbIFG, orbital inferior frontal gyrus; TriIFG, triangular inferior frontal gyrus; OperIFG, opercular inferior frontal gyrus; SupMFG, superior medial frontal gyrus; MedFG, medial frontal gyrus; STG, superior temporal gyrus; MTG, middle temporal gyrus; ITG, inferior temporal gyrus; STP, superior temporal pole; SPL, superior parietal lobule; IPL, inferior parietal lobule; SMG, supramarginal gyrus; AG, angular gyrus; PreCG, precentral gyrus; PoCG, postcentral gyrus; PCu, precuneus; SMA, supplementary motor area; RO, Rolandic operculum; MOG, middle occipital gyrus; IOG, inferior occipital gyrus

These brain regions were the bilateral middle frontal gyrus (MFG), inferior frontal gyrus (IFG), orbital inferior frontal gyrus (OrbIFG), triangular inferior frontal gyrus (TriIFG), opercular inferior frontal gyrus (OperIFG), superior temporal gyrus (STG), middle temporal gyrus (MTG), inferior temporal gyrus (ITG), precentral gyrus (PreCG), postcentral gyrus (PoCG), Rolandic operculum (RO), middle occipital gyrus (MOG) and inferior occipital gyrus (IOG), the left superior frontal gyrus (SFG), superior medial frontal gyrus (SupMFG), medial frontal gyrus (MedFG), superior temporal pole (STP), superior parietal lobule (SPL), inferior parietal lobule (IPL), supramarginal gyrus (SMG), angular gyrus (AG), precuneus (PCu), supplementary motor area (SMA), as well as the right cuneus (Table 5, Fig. 1E).

#### F. Major depression

77 studies (extracted from Neurosynth) were included in the data analysis (a complete list of the 77 studies can be found in Additional file 1: Table S6). Eighteen clusters on the brain surface were identified from the uniformity test map of the meta-analysis (Table 6).

**Table 6:** Coordinates of major depression-associated surface regions identified from meta-analysis

Cluster ID	Cluster size	Peak T	Peak coordinates			Brain regions
			x	y	z	
1	441	7.76	42	− 78	− 6	R IOG/MOG/STG/ITG/MTG
2	48	4.80	56	− 6	− 22	R MTG/ITG/STG
3	86	7.17	− 60	− 14	− 14	L MTG/ITG
4	858	9.54	− 46	26	− 10	L IFG/OrbIFG/TriIFG/OperIFG/MFG/PreCG/STG
5	51	7.76	34	22	− 10	R IFG/OrbIFG
6	351	7.17	− 2	58	20	L MedFG/SupMFG/OrbMFG/SFG
7	271	8.36	− 46	− 66	0	L MOG/MTG/ITG
8	769	8.95	36	28	2	R IFG/OrbIFG/TriIFG/OperIFG/MFG/PreCG
9	78	5.99	− 56	− 42	2	L MTG/STG
10	141	6.58	54	− 32	6	R MTG/STG
11	236	7.17	50	− 58	24	R STG/MTG/IPL/SMG/AG
12	227	6.58	2	− 54	24	R PCu/PoCG
13	90	5.99	− 48	− 66	28	L MTG/STG/IPL/AG
14	34	4.80	50	− 60	38	R IPL/SMG/AG
15	349	8.36	− 2	10	52	L MedFG/SMA
16	190	5.99	− 40	− 42	48	L IPL/SPL/PCu/PoCG
17	59	4.80	− 46	− 30	52	L IPL/PoCG
18	53	5.39	− 26	0	56	L MFG/SFG/PreCG

L, left; R, right; SFG, superior frontal gyrus; MFG, middle frontal gyrus; IFG, inferior frontal gyrus; OrbIFG, orbital inferior frontal gyrus; TriIFG, triangular inferior frontal gyrus; OperIFG, opercular inferior frontal gyrus; SupMFG, superior medial frontal gyrus; MedFG, medial frontal gyrus; OrbMFG, orbital medial frontal gyrus; STG, superior temporal gyrus; MTG, middle temporal gyrus; ITG, inferior temporal gyrus; SPL, superior parietal lobule; IPL, inferior parietal lobule; SMG, supramarginal gyrus; AG, angular gyrus; PreCG, precentral gyrus; PoCG, postcentral gyrus; PCu, precuneus; SMA, supplementary motor area; MOG, middle occipital gyrus; IOG, inferior occipital gyrus

These brain regions were the bilateral middle frontal gyrus (MFG), inferior frontal gyrus (IFG), orbital inferior frontal gyrus (OrbIFG), triangular inferior frontal gyrus (TriIFG), opercular inferior frontal gyrus (OperIFG), superior temporal gyrus (STG), middle temporal gyrus (MTG), inferior temporal gyrus (ITG), inferior parietal lobule (IPL), angular gyrus (AG), precentral gyrus (PreCG), postcentral gyrus (PoCG), precuneus (PCu) and middle occipital gyrus (MOG), the left superior frontal gyrus (SFG), medial frontal gyrus (MedFG), superior medial frontal gyrus (SupMFG), orbital medial frontal gyrus (OrbMFG), superior parietal lobule (SPL) and supplementary motor area (SMA), as well as the right supramarginal gyrus (SMG) and inferior occipital gyrus (IOG) (Table 6, Fig. 1F).

#### G. PTSD

106 studies (extracted from Neurosynth) were included in the data analysis (a complete list of the 106 studies can be found in Additional file 1: Table S7). Fourteen clusters on the brain surface were identified from the uniformity test map of the meta-analysis (Table 7).

**Table 7:** Coordinates of PTSD-associated surface regions identified from meta-analysis

Cluster ID	Cluster size	Peak T	Peak coordinates			Brain regions
			x	y	z	
1	273	8.26	50	22	− 10	R IFG/OrbIFG/TriIFG/OperIFG
2	169	8.26	− 46	20	− 4	L IFG/OrbIFG/TriIFG
3	57	6.20	− 44	40	− 6	L MFG/OrbIFG
4	95	6.20	− 42	28	14	L IFG/TriIFG/MFG
5	47	5.18	− 48	8	32	L IFG/TriIFG/OperIFG/PreCG
6	54	5.18	− 36	36	28	L MFG/SFG
7	40	7.23	− 56	− 34	36	L IPL/SMG/PoCG
8	54	7.23	− 34	10	40	L MFG/PreCG
9	345	8.26	− 2	20	42	L SMA/MedFG/SupMFG
10	184	8.26	− 40	− 54	44	L IPL/SPL/PCu/AG
11	36	5.18	34	− 70	44	R SPL/PCu/AG
12	98	7.23	44	− 42	48	R IPL/SMG/PoCG
13	60	7.23	34	10	56	R MFG/SFG
14	34	4.15	− 24	2	62	L MFG/SFG

L, left; R, right; SFG, superior frontal gyrus; MFG, middle frontal gyrus; IFG, inferior frontal gyrus; OrbIFG, orbital inferior frontal gyrus; TriIFG, triangular inferior frontal gyrus; OperIFG, opercular inferior frontal gyrus; SupMFG, superior medial frontal gyrus; MedFG, medial frontal gyrus; SPL, superior parietal lobule; IPL, inferior parietal lobule; SMG, supramarginal gyrus; AG, angular gyrus; PreCG, precentral gyrus; PoCG, postcentral gyrus; PCu, precuneus; SMA, supplementary motor area

These brain regions were the bilateral superior frontal gyrus (SFG), middle frontal gyrus (MFG), inferior frontal gyrus (IFG), orbital inferior frontal gyrus (OrbIFG), triangular inferior frontal gyrus (TriIFG), opercular inferior frontal gyrus (OperIFG), superior parietal lobule (SPL), inferior parietal lobule (IPL), supramarginal gyrus (SMG), angular gyrus (AG), postcentral gyrus (PoCG) and precuneus (PCu), as well as the left medial frontal gyrus (MedFG), superior medial frontal gyrus (SupMFG), precentral gyrus (PreCG) and supplementary motor area (SMA) (Table 7, Fig. 1G).

#### H. Schizophrenia

715 studies (extracted from Neurosynth) were included in the data analysis (a complete list of the 715 studies can be found in Additional file 1: Table S8). Eight clusters on the brain surface were identified from the uniformity test map of the meta-analysis (Table 8).

**Table 8:** Coordinates of schizophrenia-associated surface regions identified from meta-analysis

Cluster ID	Cluster size	Peak T	Peak coordinates			Brain regions
			x	y	z	
1	36	4.62	− 6	38	− 14	L MedFG/OrbMFG
2	39	6.83	− 48	− 70	− 4	L MOG/ITG/MTG
3	104	7.94	− 34	40	2	L TriIFG/OrbIFG/MFG
4	35	5.73	− 16	58	8	L MedFG/SupMFG/SFG
5	288	10.15	46	24	24	R TriIFG/OperIFG/IFG/MFG
6	47	6.83	42	6	28	R OperIFG/IFG/PreCG
7	105	6.83	− 46	8	30	L IFG/TriIFG/OperIFG/MFG/PreCG
8	163	10.15	− 2	22	40	L SupMFG/MedFG/SMA

L, left; R, right; SFG, superior frontal gyrus; MFG, middle frontal gyrus; IFG, inferior frontal gyrus; OrbIFG, orbital inferior frontal gyrus; TriIFG, triangular inferior frontal gyrus; OperIFG, opercular inferior frontal gyrus; SupMFG, superior medial frontal gyrus; MedFG, medial frontal gyrus; OrbMFG, orbital medial frontal gyrus; MTG, middle temporal gyrus; ITG, inferior temporal gyrus; PreCG, precentral gyrus; SMA, supplementary motor area; MOG, middle occipital gyrus

These brain regions were the bilateral middle frontal gyrus (MFG), inferior frontal gyrus (IFG), triangular inferior frontal gyrus (TriIFG), opercular inferior frontal gyrus (OperIFG), precentral gyrus (PreCG), as well as the left superior frontal gyrus (SFG), orbital inferior frontal gyrus (OrbIFG), medial frontal gyrus (MedFG), superior medial frontal gyrus (SupMFG), orbital medial frontal gyrus (OrbMFG), middle temporal gyrus (MTG), inferior temporal gyrus (ITG), supplementary motor area (SMA) and middle occipital gyrus (MOG) (Table 8, Fig. 1H).

### Neuroimaging-based scalp stimulation locations

**Fig. 2:**
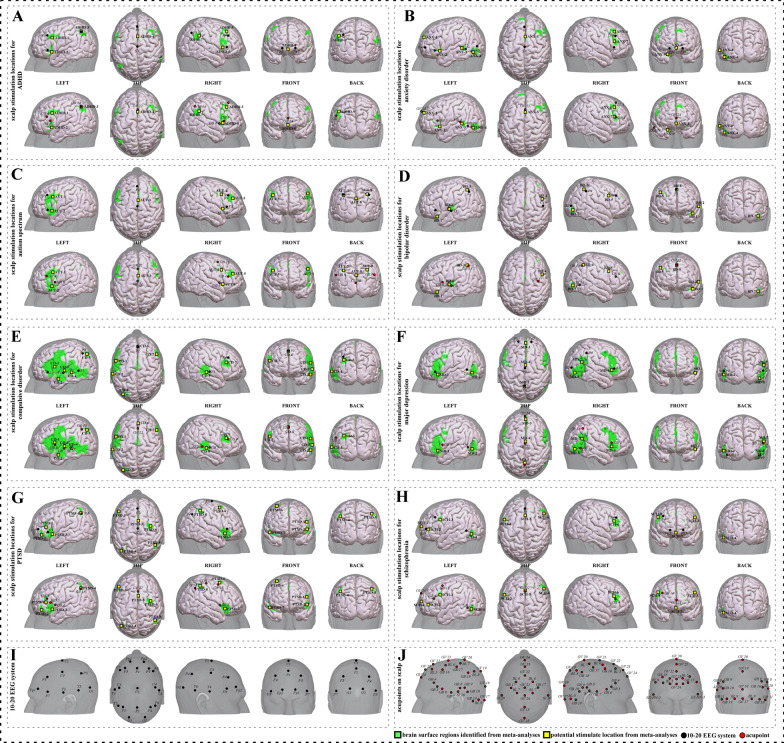
Neuroimaging-based scalp stimulation protocols for mental disorders. **A** to **H** scalp stimulation locations for ADHD, anxiety disorder, autism spectrum disorder, bipolar disorder, compulsive disorder, major depression, PTSD, and schizophrenia, respectively. Upper and lower panels of **A** to **H** applied 10–20 EEG system, and international acupoints, respectively, to facilitate identifying the locations. **I** 10–20 EEG system locations. **J** acupoint locations. ADHD, Attention deficit hyperactivity disorder; ANX, anxiety disorder; AUT, autism spectrum; BD, bipolar disorder; CD, compulsive disorder; MD, major depression; PTSD, post-traumatic stress disorder; SCH, schizophrenia; GV 17, Naohu; GV 18, Qiangjian; GV 19, Houding; GV 20, Baihui; GV 21, Qianding; GV 22, Xinhui; GV 23, Shangxing; GV 24, Shenting; GV 29, Yintang; GB 4, Hanyan; GB 5, Xuanlu; GB 6, Xuanli; GB 7, Qubin; GB 8, Shuaigu; GB 9, Tianchong; GB 10, Fubai; GB 11, Touqiaoyin; GB 15, Toulinqi; GB 16, Muchuang; GB 17, Zhengying; GB 18, Chengling; GB 19, Naokong; BL 3, Meichong; BL 5, Wuchu; BL 6, Chengguang; BL 7, Tongtian; BL 8, Luoque; BL 9, Yuzhen; ST 8, Touwei; SJ 20, Jiaosun; EX-HN 5, Taiyang

To facilitate clinical application, we further refined/simplified the results and proposed neuroimaging-based target protocols for each disorder respectively. We accomplished this by identifying the eight or nine surface regions with peak coordinates of each cluster for each disorder respectively, based on the findings from the meta-analysis. We applied two methods, the 10–20 EEG system and the international standard acupoints, to identify the potential scalp stimulation locations. To help the readers understand the specific brain functions of identified areas, we also summarized the brain functions of each identified brain region associated with a corresponding mental disorder. We named the potential targets in the order of left, top, right, front, and back view of the head. Detailed descriptions of potential targets for each disorder based on 10–20 EEG system coordinates and acupuncture points can be found in Fig. 2, and Tables 9, 10, 11, 12, 13, 14, 15 and 16, respectively.

**Table 9:** Identified scalp stimulation targets for ADHD

Cluster ID	Cluster size	Peak T	Peak coordinates			Brain regions	10–20 EEG system locations	Acupoint locations	Functions
			x	y	z				
ADHD-1	188	6.98	− 48	10	32	L IFG/dlPFC/mPFC/PreCG	1 cm posterior and inferior to F3	0.6 cun posterior and superior to GB 4	Planning complex, coordinated movements
ADHD-2	205	8.62	− 36	22	− 8	L IFG/OrbIFG	1 cm posterior and inferior to F7	0.6 cun inferior to GB 5	Language processing and comprehension, emotional recognition
ADHD-3	120	6.98	− 40	− 56	46	L IPL/PCu/AG	P3	GB 18	Sentence generation, arithmetic learning, abstract coding of numerical magnitude, spatial focusing of attention, performing verbal creative tasks, processing a sequence of actions
ADHD-4	236	8.62	− 2	20	42	L MedFG/SupMFG/mPFC	Midpoint of Fz and Cz	0.1 cun posterior to GV 22	Motor learning/imagery/control, executive control of behavior, language processing, working memory, visuospatial and visuomotor attention, pain anticipation
ADHD-5	81	6.98	36	26	40	R MFG/dlPFC/mPFC	1 cm superior to F4	0.3 cun inferior to BL 5	Planning complex movements
ADHD-6	168	6.98	36	24	8	R IFG/TriIFG	1 cm posterior to F8	Midpoint of GB 5 and GB 6	Semantic tasks (decision/generation/retrieval/working memory processes)
ADHD-7	99	6.15	58	− 46	30	R IPL/SMG	2 cm anterior and inferior to P4	0.6 cun anterior and inferior to GB 18	Reading, spatial focusing of attention, visuospatial processing
ADHD-8	105	6.98	− 2	50	− 10	L MedFG/OrbMFG	Midpoint of Fp1 and Fp2	0.6 cun anterior to GV 24	Working memory, episodic memory, and multiple-task coordination
ADHD-9	82	6.15	− 28	− 54	42	L SPL/IPL/PCu	1 cm lateral to P3	0.3 cun medial and superior to GB 9	Sentence generation, arithmetic learning, abstract coding of numerical magnitude, spatial focusing of attention, performing verbal creative tasks, executive control of behavior

L, left; R, right; MFG, middle frontal gyrus; IFG, inferior frontal gyrus; OrbIFG, orbital inferior frontal gyrus; SupMFG, superior medial frontal gyrus; MedFG, medial frontal gyrus; OrbMFG, orbital medial frontal gyrus; TriIFG, triangular inferior frontal gyrus; dlPFC, dorsolateral prefrontal cortex; mPFC, medial prefrontal cortex; SPL, superior parietal lobule; IPL, inferior parietal lobule; SMG, supramarginal gyrus; AG, angular gyrus; PreCG, precentral gyrus; PCu, precuneus; cm, centimeter; cun, Chinese inches, 1 cun is about 3.33 cm; GV 22, Xinhui; GV 24, Shenting; GB 4, Hanyan; GB 5, Xuanlu; GB 6, Xuanli; GB 9, Tianchong; GB 18, Chengling; BL 5, Wuchu

**Table 10:** Identified scalp stimulation targets for anxiety disorder

Cluster ID	Cluster size	Peak T	Peak coordinates			Brain regions	10–20 EEG system locations	Acupoint locations	Functions
			x	y	z				
ANX-1	61	5.89	− 6	56	28	L SFG/SupMFG	2 cm superior to Fp1	0.1 cun lateral to GV 24	Short term memory, evaluating recency, overriding automatic responses, verbal fluency, error detection, auditory verbal attention, inferring the intention of others, inferring deduction from spatial imagery, inductive reasoning, attributing intention, empathy, self-criticisms, attention to negative emotions
ANX-2	178	6.88	− 32	26	− 6	L IFG/OrbIFG	1 cm posterior to F7	0.6 cun posterior and inferior to GB 5	Language processing and comprehension, emotional recognition
ANX-3	80	6.88	− 56	− 40	2	L MTG	1 cm anterior to T5	0.1 cun posterior and inferior to GB 8	Selective processing of text and speech, semantic processing, word/sentence generation, attribution of intentions to others
ANX-4	55	6.88	− 44	− 74	− 8	L MOG/IOG	2 cm anterior and inferior to O1	0.3 cun posterior and inferior to GB 10	Feature-extracting, shape recognition, attentional, and multimodal integrating
ANX-5	112	7.86	2	18	44	R SMA	Midpoint of Fz and Cz	0.1 cun posterior to GV 22	Motor learning/imagery/control, executive control of behavior, language processing, working memory, memory retrieval, visual attention and control of eye movements, auditory imagery
ANX-6	55	4.90	38	28	42	R MFG	1 cm superior to F4	0.3 cun lateral to BL 5	Motor control, executive control of behavior, planning, language processing, working memory, memory retrieval, visuospatial and visuomotor attention, processing related to uncertainty, pain anticipation
ANX-7	101	5.89	50	22	20	R IFG/dlPFC	Midpoint of F4 and F8	GB 4	Semantic tasks, suppression of response tendencies, understand the mental state of others through observation, inferring, and projecting, music perception
ANX-8	276	6.88	− 2	54	− 12	L MedFG/OrbMFG	Midpoint of Fp1 and Fp2	0.6 cun anterior to GV 24	Scheduling operations in multiple tasks, decision making, working memory
ANX-9	55	12.80	28	4	− 22	R STP	1 cm lateral and inferior to Fp2	1 cun medial to EX-HN-5	Emotional learning, reward, memory modulation

ANX, anxiety disorder; L, left; R, right; STP, superior temporal pole; IFG, inferior frontal gyrus; OrbIFG, orbital inferior frontal gyrus; MedFG, medial frontal gyrus; OrbMFG, orbital medial frontal gyrus; MOG, middle occipital gyrus; IOG, inferior occipital gyrus; MTG, middle temporal gyrus; dlPFC, dorsolateral prefrontal cortex; SFG, superior frontal gyrus; SupMFG, superior medial frontal gyrus; MFG, middle frontal gyrus; SMA, supplementary motor area; cm, centimeter; cun, Chinese inches, 1 cun is about 3.33 cm; EX-HN-5, Taiyang; BL 5, Wuchu; GV 22, Xinhui; GV 24, Shenting; GB 4, Hanyan; GB 5, Xuanlu; GB 8, Shuaigu; GB10, Fubai

**Table 11:** Identified scalp stimulation targets for autism spectrum

Cluster ID	Cluster size	Peak T	Peak coordinates			Brain regions	10–20 EEG system locations	Acupoint locations	Functions
			x	y	z				
AUT-1	674	17.27	− 46	10	32	L IFG/TriIFG/OperIFG/MFG/PreCG	1.5 cm posterior and inferior to F3	0.6 cun posterior and superior to GB 4	Motor sequencing/planning/learning/imagery, language processing, working memory, visuospatial attention, executive control of behavior, processing emotions and self-reflections in decision making
AUT-2	310	13.39	− 38	22	− 6	L IFG/OrbIFG/TriIFG/OperIFG/STG	1 cm posterior to F7	0.3 cun anterior and inferior to GB 6	Motor learning/imagery/control, executive control of behavior, language processing, working memory, auditory imagery, pain anticipation
AUT-3	535	16.21	0	14	48	SMA/MedFG/SupMFG/SFG	Anterior 2/3 point of line Fz and Cz	Midpoint of GV 21 and GV 22	Somesthetic senses
AUT-4	246	14.45	46	8	28	R IFG/OperIFG/TriIFG/MFG/PreCG	1.5 cm posterior to F4	0.6 cun anterior and inferior to GB 16	Semantic tasks, theory of mind (the ability to understand the mental state of others through observation, inferring, and projecting)
AUT-5	97	8.44	40	38	24	R MFG/IFG/TriIFG	1 cm anterior and inferior to F4	0.3 cun anterior and superior to GB 4	Attributing intention, theory of mind, suppressing sadness, working memory, spatial memory, recognition, recall, recognizing the emotions of others, planning, attention to positive emotions
AUT-6	67	17.27	34	26	− 6	R IFG/OrbIFG	1 cm posterior and inferior to F8	0.1 cun anterior and inferior to GB 6	Interoceptive awareness, motor control, self-awareness, social emotions, body representation and subjective emotional experience, salience, auditory perception
AUT-7	158	11.62	− 30	− 56	40	L IPL/SPL/SMG/PCu	P3	0.6 cun medial and superior to GB 9	Visuospatial processing, motor execution/imagery, working memory (motor, visual, auditory, emotional, verbal), pain perception, language processing, processing emotions and self-reflections during decision making
AUT-8	133	13.03	− 2	− 56	26	L PreCG/PCu/SPL	1 cm posterior to midpoint of P3 and P4	0.6 cun superior to GV 18	Processing semantic emotional information, episodic memory retrieval, evaluative judgment, precautionary reasoning, control of self-determined finger movements
AUT-9	50	10.21	34	− 56	44	R IPL/SPL/AG	P4	0.6 cun medial and superior to GB 9	Spatial focusing of attention, visuospatial processing, executive control of behavior, theory of mind

AUT, autism; L, left; R, right; SFG, superior frontal gyrus; MFG, middle frontal gyrus; IFG, inferior frontal gyrus; OrbIFG, orbital inferior frontal gyrus; TriIFG, triangular inferior frontal gyrus; OperIFG, opercular inferior frontal gyrus; SupMFG, superior medial frontal gyrus; MedFG, medial frontal gyrus; STG, superior temporal gyrus; SPL, superior parietal lobule; IPL, inferior parietal lobule; SMG, supramarginal gyrus; AG, angular gyrus; PreCG, precentral gyrus; PCu, precuneus; SMA, supplementary motor area; cm, centimeter; cun, Chinese inches, 1 cun is about 3.33 cm; GB 4, Hanyan; GB 6, Xuanli; GB 9, Tianchong; GB 16, Muchuang; GV 18, Qiangjian; GV 21, Qianding; GV 22, Xinhui

**Table 12:** Identified scalp stimulation targets for bipolar disorder

Cluster ID	Cluster size	Peak T	Peak coordinates			Brain regions	10–20 EEG system locations	Acupoint locations	Functions
			x	y	z				
BD-1	55	7.29	− 40	18	− 20	L IFG/OrbIFG/STP	2 cm inferior to F7	1 cun anterior and inferior to GB 6	Attribution of intentions/mental states to others, self/other distinction, experiencing emotional state, response to threat/fearful stimulus, multimodal memory retrieval, semantic processing, humor comprehension, inferential reasoning
BD-2	37	7.29	− 58	− 14	6	L STG	0.5 cm anterior and superior to T3	0.1 cun anterior and superior to GB 7	Performing basic and higher functions in hearing, language switching
BD-3	72	8.02	− 30	− 58	44	L IPL/SPL	0.5 cm anterior and superior to P3	0.1 cun anterior and superior to GB 18	Sentence generation, reading, calculation, arithmetic learning, performing verbal creative tasks, executive control of behavior
BD-4	87	8.74	46	4	30	R IFG/OperIFG/MFG/PreCG	2 cm anterior to C4	0.6 cun anterior and lateral to GB 17	Motor sequencing/planning/learning/imagery, language processing, working memory, visuospatial attention, visuomotor attention, executive control of behavior, same-different discrimination
BD-5	38	5.84	30	40	24	R MFG/SFG	1.5 cm posterior to F4	1 cun posterior and superior to GB 4	Attributing intention, theory of mind, suppressing sadness, working memory, spatial memory, recognition, recall, recognizing the emotions of others, planning, calculation, attention to positive emotions
BD-6	54	6.57	32	− 54	40	R IPL/SPL/SMG/AG	0.5 cm posterior and superior to P4	0.1 cun anterior and superior to GB 18	Reading, spatial focusing of attention, visuospatial processing, theory of mind, executive control of behavior
BD-7	60	7.29	40	− 78	− 4	R MOG/IOG/ITG	1 cm anterior and inferior to O2	0.6 cun posterior to GB 10	Object recognition, visual function of processing faces
BD-8	205	8.02	− 4	8	52	L MedFG/SFG/SMA	Fz	GV 22	Motor sequencing/planning/learning/imagery, speech motor programming, working memory, episodic long-term memory, topographic memory, attention, planning/solving novel problems, executive control of behavior, processing emotions and self-reflections in decision making

BD, bipolar disorder; L, left; R, right; SFG, superior frontal gyrus; MFG, middle frontal gyrus; IFG, inferior frontal gyrus; OrbIFG, orbital inferior frontal gyrus; OperIFG, opercular inferior frontal gyrus; MedFG, medial frontal gyrus; STG, superior temporal gyrus; ITG, inferior temporal gyrus; STP, superior temporal pole; SPL, superior parietal lobule; IPL, inferior parietal lobule; SMG, supramarginal gyrus; AG, angular gyrus; PreCG, precentral gyrus; SMA, supplementary motor area; MOG, middle occipital gyrus; IOG, inferior occipital gyrus; cm, centimeter; cun, Chinese inches, 1 cun is about 3.33 cm; GB 4, Hanyan; GB 6, Xuanli; GB 7, Qubin; GB 10, Fubai; GB 17, Zhengying; GB 18, Chengling; GV 22, Xinhui

**Table 13:** Identified scalp stimulation targets for compulsive disorder

Cluster ID	Cluster size	Peak T	Peak coordinates			Brain regions	10–20 EEG system locations	Acupoint locations	Functions
			x	y	z				
CD-1	1544	10.26	− 54	10	18	L IFG/TriIFG/OperIFG/MFG/PreCG/PoCG/RO	2 cm posterior and superior to F7	0.1 cun superior to GB 6	Semantic tasks, hand movements, theory of mind (the ability to understand the mental state of others through observation, inferring, and projecting)
CD-2	153	7.62	− 54	− 2	− 16	L MTG/STG/ITG/STP	2 cm anterior and inferior to T3	0.6 cun anterior and inferior to GB 7	Selective processing of text and speech, semantic processing, word/sentence generation, deductive reasoning, observation of motion, attribution of intentions to other, deductive reasoning
CD-3	149	6.75	− 62	− 14	6	L STG/PreCG/PoCG/RO	0.5 cm posterior and superior to T3	0.1 cun superior to GB 7	Performing basic and higher functions in hearing
CD-4	846	9.38	− 64	− 34	4	L MTG/STG/ITG/IPL/SMG/MOG/IOG	2 cm anterior to T5	SJ 20	Selective processing of text and speech, semantic processing, word/sentence generation, deductive reasoning, processing complex sounds, attribution of intentions to other, deductive reasoning
CD-5	71	6.75	− 30	− 72	44	L SPL/IPL/PCu/MOG/AG	1 cm posterior to P3	0.3 cun posterior to GB 18	Sentence generation, reading, calculation, arithmetic learning, abstract coding of numerical magnitude, spatial focusing of attention, performing verbal creative tasks, theory of mind, executive control of behavior
CD-6	320	7.62	− 2	14	52	L MedFG/SupMFG/SFG/SMA	Fz	0.3 cun anterior to GV 22	Motor sequencing/planning/learning/imagery, speech motor programming, working memory, episodic long-term memory, attention, planning/solving novel problems, processing emotions and self-reflections in decision making
CD-7	79	6.75	54	28	22	R MFG/IFG/TriIFG	1 cm posterior and inferior to F4	0.1 cun posterior and superior to GB 4	Attributing intention, theory of mind, suppressing sadness, working memory, spatial memory, recognition, recall, recognizing the emotions of others, planning, calculation, attention to positive emotions
CD-8	285	7.62	62	− 34	0	R STG/MTG	0.5 cm posterior to T4	Midpoint of GB 7 and SJ 20	Prosodic integration, observation of motion, processing complex sounds, attribution of intentions to others

CD, compulsive disorder; L, left; R, right; SFG, superior frontal gyrus; MFG, middle frontal gyrus; IFG, inferior frontal gyrus; TriIFG, triangular inferior frontal gyrus; OperIFG, opercular inferior frontal gyrus; SupMFG, superior medial frontal gyrus; MedFG, medial frontal gyrus; STG, superior temporal gyrus; MTG, middle temporal gyrus; ITG, inferior temporal gyrus; STP, superior temporal pole; SPL, superior parietal lobule; IPL, inferior parietal lobule; SMG, supramarginal gyrus; AG, angular gyrus; PreCG, precentral gyrus; PoCG, postcentral gyrus; PCu, precuneus; SMA, supplementary motor area; RO, Rolandic operculum; MOG, middle occipital gyrus; IOG, inferior occipital gyrus; cm, centimeter; cun, Chinese inches, 1 cun is about 3.33 cm; GB 4, Hanyan; GB 6, Xuanli; GB 7, Qubin; GB 18, Chengling; SJ 20, Jiaosun; GV 22 Xinhui

**Table 14:** Identified scalp stimulation targets for major depression

Cluster ID	Cluster size	Peak T	Peak coordinates			Brain regions	10–20 EEG system locations	Acupoint locations	Functions
			x	y	z				
MD-1	858	9.54	− 46	26	− 10	L IFG/OrbIFG/TriIFG/OperIFG/MFG/PreCG/STG	0.5 cm posterior and inferior to F7	0.6 cun anterior and inferior to GB 6	Language processing and comprehension, emotional recognition
MD-2	271	8.36	− 46	− 66	0	L MOG/MTG/ITG	1.5 cm posterior and inferior to T5	0.3 cun posterior and inferior to GB 10	Tracking visual motion patterns, feature-based attention, orientation-selective attention, visual memory recognition, spatial working memory, inferential reasoning, visual mental imagery
MD-3	351	7.17	− 2	58	20	L MedFG/SupMFG/OrbMFG/SFG	Midpoint of Fp1 and Fp2	0.3 cun posterior to GV 24	Working memory, episodic memory, multiple-task coordination, decision making
MD-4	349	8.36	− 2	10	52	L MedFG/SMA	0.5 cm anterior to Cz	0.1 cun anterior to GV 21	Motor sequencing/planning/learning/imagery, language processing, working memory, episodic long-term memory, visuospatial attention, visuomotor attention, executive control of behavior, processing emotions and self-reflections in decision making
MD-5	227	6.58	2	− 54	24	R PCu/PoCG	Pz	0.1 cun anterior to GV 19	Self-consciousness, attention, episodic memory retrieval, working memory, conscious perception, response inhibition, visuospatial mental operations
MD-6	769	8.95	36	28	2	R IFG/OrbIFG/TriIFG/OperIFG/MFG/PreCG	0.5 cm posterior and inferior to F8	0.3 cun inferior to GB 5	Semantic decision tasks (determining whether a word represents an abstract or a concrete entity), generation tasks (generating a verb associated with a noun), semantic retrieval/working memory processes
MD-7	236	7.17	50	− 58	24	R STG/MTG/IPL/SMG/AG	Midpoint of P4 and O2	Midpoint of GB 10 and GB 18	Reading, spatial focusing of attention, theory of mind, executive control of behavior
MD-8	441	7.76	42	− 78	− 6	R IOG/MOG/STG/ITG/MTG	2 cm anterior and inferior to O2	0.6 cun posterior to GB 10	Visuo-spatial information processing, visual memory recognition, spatial working memory, visual mental imagery

MD, major depression; L, left; R, right; SFG, superior frontal gyrus; MFG, middle frontal gyrus; IFG, inferior frontal gyrus; OrbIFG, orbital inferior frontal gyrus; TriIFG, triangular inferior frontal gyrus; OperIFG, opercular inferior frontal gyrus; SupMFG, superior medial frontal gyrus; MedFG, medial frontal gyrus; OrbMFG, orbital medial frontal gyrus; STG, superior temporal gyrus; MTG, middle temporal gyrus; ITG, inferior temporal gyrus; IPL, inferior parietal lobule; SMG, supramarginal gyrus; AG, angular gyrus; PreCG, precentral gyrus; PoCG, postcentral gyrus; PCu, precuneus; SMA, supplementary motor area; MOG, middle occipital gyrus; IOG, inferior occipital gyrus; cm, centimeter; cun, Chinese inches, 1 cun is about 3.33 cm. GB 5, Xuanlu; GB 6, Xuanli; GB 10, Fubai; GB 18, Chengling; GV 19, Houding; GV 21, Qianding; GV 24, Shenting

**Table 15:** Identified scalp stimulation targets for PTSD

Cluster ID	Cluster size	Peak T	Peak coordinates			Brain regions	10–20 EEG system locations	Acupoint locations	Functions
			**x**	**y**	**z**				
PTSD-1	95	6.20	− 42	28	14	L IFG/TriIFG/MFG	2 cm posterior to Fp1	0.3 cun anterior and inferior to GB 4	Sustaining attention and managing working memory, regulate self-control
PTSD-2	57	6.20	− 44	40	− 6	L MFG/OrbIFG	2 cm anterior and inferior to F7	1 cun anterior and inferior to GB 5	Language processing and comprehension, emotional recognition (fear, disgust, and anger)
PTSD-3	169	8.26	− 46	20	− 4	L IFG/OrbIFG/TriIFG	1 cm posterior and inferior to F7	0.3 cun anterior and inferior to GB 6	Language processing and comprehension, emotional recognition (fear, disgust, and anger)
PTSD-4	184	8.26	− 40	− 54	44	L IPL/SPL/PCu/AG	P3	GB 18	Sentence generation, reading, calculation, arithmetic learning, abstract coding of numerical magnitude, spatial focusing of attention, performing verbal creative tasks, theory of mind, executive control of behavior, processing a sequence of actions
PTSD-5	345	8.26	− 2	20	42	L SMA/MedFG/SupMFG	Midpoint of Fz and Cz	0.3 cun posterior to GV 22	Motor learning/imagery/control, executive control of behavior, speech motor programming, language processing, working memory, visuospatial and visuomotor attention, inductive reasoning, pain anticipation
PTSD-6	60	7.23	34	10	56	R MFG/SFG	2 cm anterior and lateral to Cz	0.1 cun medial to GB 16	Motor sequencing/planning/learning/imagery, language processing, working memory, episodic long-term memory, visuospatial attention, visuomotor attention, executive control of behavior
PTSD-7	273	8.26	50	22	− 10	R IFG/OrbIFG/TriIFG/OperIFG	1 cm posterior and inferior to F8	0.3 cun anterior and inferior to GB 6	Language processing and comprehension, emotional recognition (fear, disgust, and anger)
PTSD-8	98	7.23	44	− 42	48	R IPL/SMG/PoCG	2 cm anterior and superior to P4	Midpoint of GB 17 and GB 18	Semantic processing, retrieval of unpleasant experiences, working memory, executive control of behavior, visuomotor transformation/motor planning, somatosensory spatial discrimination, social perception and empathy, emotions vs. Self-reflections in decision-making

L, left; R, right; SFG, superior frontal gyrus; MFG, middle frontal gyrus; IFG, inferior frontal gyrus; OrbIFG, orbital inferior frontal gyrus; TriIFG, triangular inferior frontal gyrus; OperIFG, opercular inferior frontal gyrus; SupMFG, superior medial frontal gyrus; MedFG, medial frontal gyrus; SPL, superior parietal lobule; IPL, inferior parietal lobule; SMG, supramarginal gyrus; AG, angular gyrus; PoCG, postcentral gyrus; PCu, precuneus; SMA, supplementary motor area; cm, centimeter; cun, Chinese inches, 1 cun is about 3.33 cm; GB 4, Hanyan; GB 5, Xuanlu; GB 6, Xuanli; GB 16, Muchuang; GB 17, Zhengying; GB 18, Chengling; GV 22, Xinhui

**Table 16:** Identified scalp stimulation targets for schizophrenia

Cluster ID	Cluster size	Peak T	Peak coordinates			Brain regions	10–20 EEG system locations	Acupoint locations	Functions
			x	y	z				
SCH-1	35	5.73	− 16	58	8	L IFG/dlPFC/mPFC/PreCG	1 cm medial to Fp1	0.6 cun lateral and inferior to GV 24	Working memory, episodic memory, multiple-task coordination
SCH-2	104	7.94	− 34	40	2	R IFG/TriIFG	0.5 cm posterior to Fp1	0.6 cun anterior and inferior to GB 4	Sustaining attention, managing working memory
SCH-3	105	6.83	− 46	8	30	L IPL/PCu/AG	1 cm posterior and inferior to F3	0.6 cun anterior and inferior to GB 16	Motor sequencing/planning/learning/imagery, language processing, working memory, episodic long-term memory, visuospatial attention, visuomotor attention, executive control of behavior, processing emotions and self-reflections in decision making
SCH-4	39	6.83	− 48	− 70	− 4	L IFG/OrbIFG	1.5 cm posterior and inferior to T5	0.3 cun posterior and inferior to GB 10	Detection of light intensity, tracking visual motion patterns, visual priming, visual memory recognition, spatial working memory, inferential reasoning, visual mental imagery
SCH-5	163	10.15	− 2	22	40	L MedFG/ SupMFG/mPFC	1 cm posterior to Fz	GV 22	Motor learning/imagery/control, executive control of behavior, language processing, working memory, visuospatial and visuomotor attention, inductive reasoning, pain anticipation
SCH-6	47	6.83	42	6	28	R MFG/dlPFC/mPFC	1 cm posterior and inferior to F4	0.1 cun superior and posterior to GB 4	Motor sequencing/planning/learning/imagery, language processing, working memory, episodic long-term memory, visuospatial attention, visuomotor attention, executive control of behavior, same-different discrimination
SCH-7	36	4.62	− 6	38	− 14	L MedFG/OrbMFG	0.5 cm anterior to the midpoint of Fp1 and Fp2	0.3 cun anterior to GV 24	Decision making involving reward, face-name association, nonspeech processing
SCH-8	288	10.15	46	24	24	R IPL/SMG	1 cm lateral to F4	0.3 cun lateral to GB 16	Attributing intention, theory of mind, suppressing sadness, working memory, spatial memory, recognition, recall, recognizing the emotions of others, planning, calculation, semantic and perceptual processing of odors, religiosity, attention to positive emotions

SCH, schizophrenia; L, left; R, right; SFG, superior frontal gyrus; MFG, middle frontal gyrus; IFG, inferior frontal gyrus; OrbIFG, orbital inferior frontal gyrus; TriIFG, triangular inferior frontal gyrus; OperIFG, opercular inferior frontal gyrus; SupMFG, superior medial frontal gyrus; MedFG, medial frontal gyrus; OrbMFG, orbital medial frontal gyrus; MTG, middle temporal gyrus; ITG, inferior temporal gyrus; PreCG, precentral gyrus; SMA, supplementary motor area; MOG, middle occipital gyrus; cm, centimeter; cun, Chinese inches, 1 cun is about 3.33 cm; GB 4, Hanyan; GB 10, Fubai; GB 16, Muchuang; GV 22, Xinhui; GV 24, Shenting

#### A. ADHD

We proposed nine potential targets for treating ADHD (named ADHD-1 to ADHD-9). These targets were located mainly in the frontal gyrus, precentral gyrus, precuneus, parietal lobe, supramarginal gyrus, and angular gyrus (Table 9, Fig. 2A).

#### B. Anxiety disorder

We proposed nine potential targets for treating anxiety disorder (named ANX-1 to ANX-9). These targets were located mainly in the frontal gyrus, supplementary motor area, temporal gyrus, and occipital gyrus (Table 10, Fig. 2B).

#### C. Autism spectrum disorder

We proposed nine potential targets for treating autism spectrum disorder (named AUT-1 to AUT-9). These targets were located mainly in the frontal gyrus, supplementary motor area, precentral gyrus, precuneus, temporal gyrus, parietal lobe, supramarginal gyrus, and angular gyrus (Table 11, Fig. 2C).

#### D. Bipolar disorder

We proposed eight potential targets for treating bipolar disorder (named BD-1 to BD-8). These targets were located mainly in the frontal gyrus, supplementary motor area, precentral gyrus, precuneus, temporal gyrus, parietal lobe, supramarginal gyrus, angular gyrus, and occipital gyrus (Table 12, Fig. 2D).

#### E. Compulsive disorder

We proposed eight potential targets for treating compulsive disorder (named CD-1 to CD-8). These targets were located mainly in the frontal gyrus, supplementary motor area, precentral and postcentral gyrus, temporal gyrus, parietal lobe, supramarginal gyrus, angular gyrus, and occipital gyrus (Table 13, Fig. 2E).

#### F. Major depression

We proposed eight potential targets for treating major depression (named MD-1 to MD-8). These targets were located mainly in the frontal gyrus, supplementary motor area, precentral and postcentral gyrus, precuneus, temporal gyrus, parietal lobe, supramarginal gyrus, angular gyrus, and occipital gyrus (Table 14, Fig. 2F).

#### G. PTSD

We proposed eight potential targets for treating PTSD (named PTSD-1 to PTSD-8). These targets were located mainly in the frontal gyrus, supplementary motor area, postcentral gyrus, precuneus, temporal gyrus, parietal lobe, supramarginal gyrus, and angular gyrus (Table 15, Fig. 2G).

#### H. Schizophrenia

We proposed eight potential targets for treating schizophrenia (named SCH-1 to SCH-8). These targets were located mainly in the frontal gyrus, supplementary motor area, precentral gyrus, precuneus, temporal gyrus, and occipital gyrus (Table 16, Fig. 2H).

### Overlapped surface regions among mental disorders

**Fig. 3:**
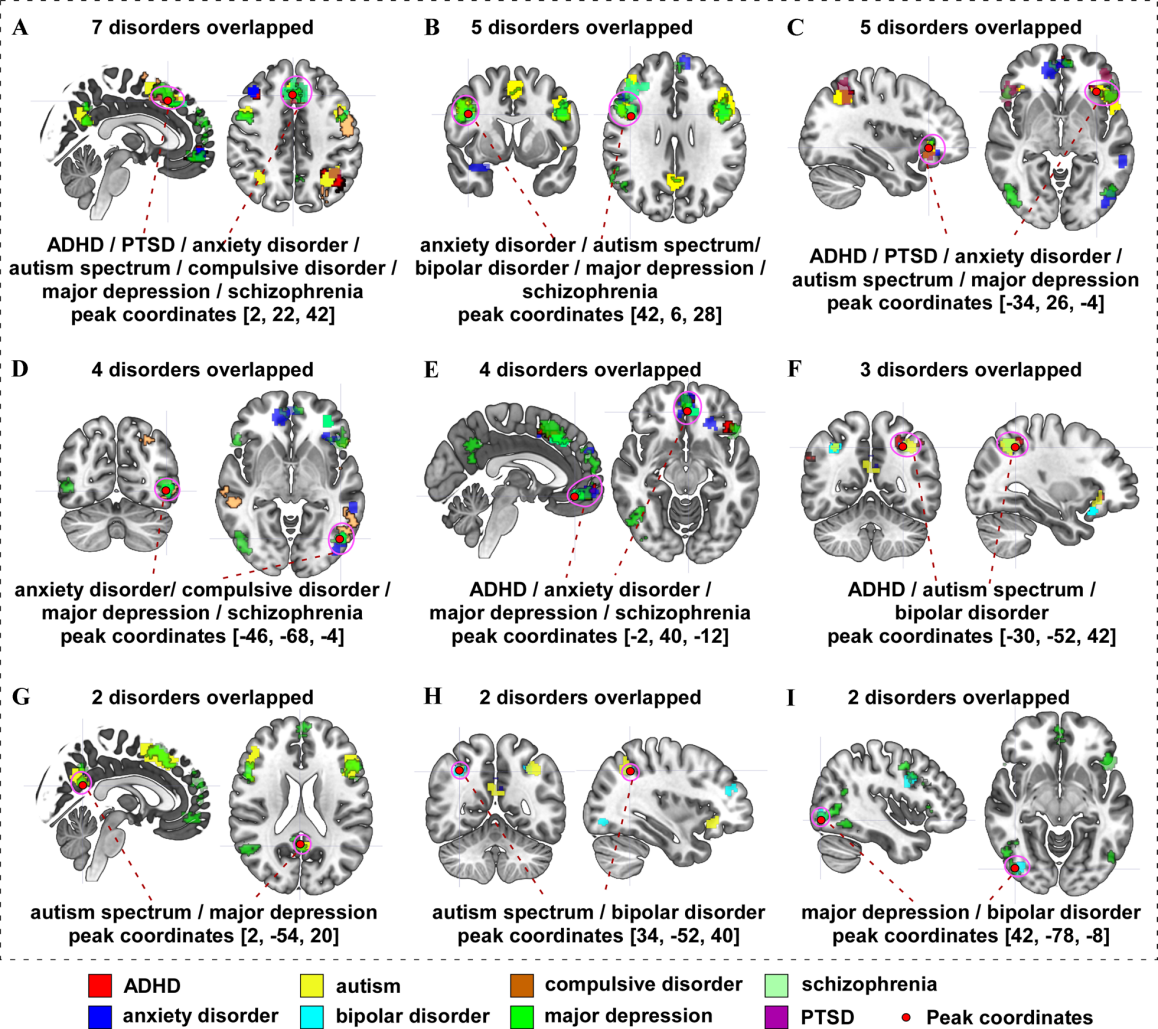
Overlap surface regions among different mental disorders. **A** ADHD, PTSD, anxiety, autism spectrum, compulsive disorder, major depression, and schizophrenia overlapping on right SMA/MedFG. **B** anxiety, autism spectrum, bipolar disorder, major depression, and schizophrenia overlapping on right dlPFC. **C** ADHD, PTSD, anxiety, autism spectrum, and major depression overlapping on left IFG/lateral OrbPFC. **D** anxiety, compulsive disorder, major depression, and schizophrenia overlapping on left MTG/STG/ITG//IOG/MOG. **E** ADHD, anxiety, major depression, and schizophrenia overlapping on left OrbMFG/VenMedFG. **F** ADHD, autism spectrum, and bipolar disorder overlapping on left IPL/SPL/SMG/AG. **G** Autism spectrum and major depression overlapping on right PCu. **H** autism spectrum and bipolar disorder overlapping on right R IPL/SPL/SMG/AG. **I** Major depression and bipolar disorder overlapped on right ITG/MTG/STG/IOG/MOG

We also explored overlap regions across different mental disorders (Table 17 and Fig. 3). We found that: a) ADHD, PTSD, anxiety disorder, autism spectrum disorder, compulsive disorder, major depression, and schizophrenia show an overlap on the medial frontal gyrus (MedFG)/SMA (Fig. 3A); b) the right dorsal lateral prefrontal cortex (dlPFC) is involved in anxiety disorder, autism spectrum disorder, bipolar disorder, major depression, and schizophrenia (Fig. 3B); c) the left IFG/lateral orbital prefrontal cortex (OrbPFC) contributed to ADHD, PTSD, anxiety disorder, autism spectrum disorder, and major depression (Fig. 3C); d) anxiety disorder, compulsive disorder, major depression, and schizophrenia display an overlap on the left MTG/STG/ITG//IOG/MOG (Fig. 3D); e) the orbital medial frontal gyrus (OrbMFG)/ventral medial frontal gyrus (VenMFG) are associated with ADHD, anxiety disorder, major depression, and schizophrenia (Fig. 3E); f) ADHD, autism spectrum disorder, and bipolar disorder are allying with the left IPL/SPL/SMG/AG (Fig. 3F); g) autism spectrum disorder and major depression have an overlap region in the PCu (Fig. 3G); h) autism spectrum disorder and bipolar disorder overlap in the right IPL/SPL/SMG/AG (Fig. 3H); and i) the right ITG/MTG/STG/IOG/MOG areas are involved in major depression and bipolar disorder (Fig. 3I).

**Table 17:** Overlap surface regions among different mental disorders

Cluster ID	Peak coordinates			Overlap brain regions	Overlap disorders
	x	y	z		
A	2	22	42	R SMA/MedFG	7 disorders: ADHD, PTSD, anxiety disorder, autism spectrum disorder, compulsive disorder, major depression, schizophrenia
B	42	6	28	R dlPFC	5 disorders: anxiety disorder, autism spectrum, bipolar disorder, major depression, schizophrenia
C	− 34	26	− 4	L IFG/lateral OrbPFC	5 disorders: ADHD, PTSD, anxiety disorder, autism spectrum disorder, major depression
D	− 46	− 68	− 4	L MTG/STG/ITG//IOG/MOG	4 disorders: anxiety disorder, compulsive disorder, major depression, schizophrenia
E	− 2	40	− 12	L OrbMFG/VenMedFG	4 disorders: ADHD, anxiety disorder, major depression, schizophrenia
F	− 30	− 52	42	L IPL/SPL/SMG/AG	3 disorders: ADHD, autism spectrum disorder, bipolar disorder
G	2	− 54	20	R PCu	2 disorders: autism spectrum disorder, major depression
H	34	− 52	40	R IPL/SPL/SMG/AG	2 disorders: autism spectrum disorder, bipolar disorder
I	42	− 78	− 8	R ITG/MTG/STG/IOG/MOG	2 disorders: major depression, bipolar disorder

L, left; R, right; IFG, inferior frontal gyrus; MedFG, medial frontal gyrus; OrbMFG, orbital medial frontal gyrus; VenMFG ventral medial frontal gyrus; dlPFC, dorsal lateral prefrontal cortex; OrbPFC, orbital prefrontal cortex; MTG, middle temporal gyrus; ITG, inferior temporal gyrus; STG, superior temporal gyrus; IPL, inferior parietal lobe; SPL, superior parietal lobe; SMA, supplementary motor area; SMG, supramarginal gyrus; AG, angular gyrus; PCu, precuneus; MOG, middle occipital gyrus; IOG, inferior occipital gyrus

## Discussion

In this study, we investigated potential targets for scalp stimulation (scalp acupuncture/transcranial stimulation) in the treatment of eight common mental disorders using a meta-analysis method that incorporates text-mining, meta-analysis and machine-learning techniques. To further facilitate its clinical application, we selected eight to nine potential targets (peak coordinates mapped onto brain surface using MRIcroGL and SurfIce) for each disorder based on the significance of each brain region derived from the meta-analysis. Finally, we used the 10–20 EEG system and existing acupoints to aid in locating corresponding regions on the scalp for these targets. We believe that the target protocols may shed light on the development of scalp stimulation for the treatment of mental disorders.

Neurosynth is an automated tool that does not perform content analyses of how terms (each mental disorder) are being used in a literature, and all results such as activations/deactivations/connectivity/cerebral blood flow that appear in the paper will be included indiscriminately. Nevertheless, this does not prevent Neurosynth from supplying robust quantitative reverse inference data consistent with other databases and methods of analysis [15, 25]. We believe this character/feature of Neurosynth will allow us to include all brain regions involved in brain pathophysiology of mental disorders. Lending support to this method’s applicability, our findings are consistent with previous brain imaging studies on ADHD [26–28], anxiety disorder [29–31], autism spectrum disorder [32, 33], bipolar disorder [34, 35], compulsive disorder [36, 37], major depression [38, 39], PTSD [40, 41], and schizophrenia [42–44].

Since the Neurosynth database contains a broad set of term-to-activation mappings, it will allow us to quantitatively identify emotional/pathological states from patterns of observed brain activity [15]. This however carries the problem of ‘reverse inference’, as most neuroimaging studies are designed to identify neural changes that result from known psychological manipulations or pathological status and not to determine what emotional/pathological state(s) a given pattern of activity implies, while the reverse inference requires knowledge of which brain regions/circuits are selectively, and not just consistently, associated with particular emotional/pathological states [15, 23, 24].

### Overlap regions among different mental disorders

Although each mental disorder is associated with different characteristic symptoms, the boundaries between mental disorders are complex and contentious. For example, schizophrenia, major depression, and bipolar disorder display overlapping clinical symptoms, particularly within the mood and psychosis spectrum [45, 46]. As different brain regions are involved in different specific functions, symptoms may be regarded as the causes or effects of dysfunction in certain regions. A large body of evidence suggests the brain regions and neuromodulatory systems involved in different mental disorders exhibit great overlap, and ultimate behavioral output circuits may be largely shared among disorders [47]. In this regard, identifying overlap regions associated with different mental disorders may be especially useful in delineating the etiology of mental disorders.

We found that the MedFG/SMA are involved in 7 disorders, including ADHD, PTSD, anxiety disorder, autism spectrum disorder, compulsive disorder, major depression, and schizophrenia. A large number of studies have revealed that the MedFG plays a crucial role in social cognition, an umbrella term for cognitive and emotional processes and abilities involved in effective interaction with the self and others, including representations of internal somatic states, knowledge about the self, perceptions of others and interpersonal motivations [48–51]. For instance, children with autism spectrum disorder (ASD) are characterized as having impairments in social communication and interaction as well as a range of stereotypic behaviors and language abilities [52]. Also, patients with ADHD become distracted because their focus on one object in the moment silences other, weaker messages. The frontal areas, especially the MedFG, are the most frequently reported regions of functional impairment in ASD and ADHD [53–56].

The SMA plays a key role in cognitive performance, especially in working memory. In a recent study, researchers found that damage to the SMA does not affect cognitive processes other than working memory, and as such, working memory impairment should be recognized as part of SMA syndrome [57]. Literature has suggested that working memory may be compromised in various mental disorders [58, 59]. It has been particularly shown that anxiety constrains cognition by biasing attention toward the anticipation of threat, and that anxiety may reflect the highest level of normal motivational control in working memory [60, 61].

In addition, from the perspective of genetics, studies have suggested that approximately 80% of genetic syndromes currently known to be associated with ASD are characterized by motor impairments, which are closely related to SMA. These disturbances may be the earliest identifiable clinical abnormalities in ASD patients [62, 63].

We found that the right dlPFC is a notable region that is involved in 5 disorders (anxiety disorder, autism spectrum disorder, bipolar disorder, major depression, and schizophrenia). Literature suggests that the neural basis for emotion regulation deficits in mental disorders centers upon abnormalities within the fronto-limbic (cognitive-emotion) pathway [64, 65]. Thus, normalization of the fronto-limbic pathway would be beneficial to emotional regulation. For instance, previous studies revealed that compared with healthy controls, adults and children with anxiety disorders demonstrated a decreased functional connectivity between the amygdala and frontal cortex, especially the dlPFC [66, 67], as well as an increased gray matter volume and decreased structural connectivity between these regions [4]. Also, the dlPFC is widely used as a target for anxiety disorder [68], autism spectrum disorder [69], bipolar disorder [70], major depression [8], and schizophrenia [7], and achieves significant clinical improvement.

We also found that anxiety disorder, bipolar disorder, compulsive disorder, major depression, and schizophrenia displayed overlap in the temporal and occipital regions. The temporal lobe has prominently been featured in studies of mental disorders due to its role in auditory and language processing. A study conducted by Anderson et al. revealed that various temporal lobe abnormalities cause the characteristic deficits in schizophrenia [71]. In addition, a previous study observed that patients with anxiety disorders demonstrate a significantly larger volume of white and grey matter in the STG [72]. Zhao and colleagues applied fMRI to investigate the activation and connectivity of the STG in patients with anxiety during different tasks and found that the activity of the STG increased during the silence task, while the functional connectivity decreased between the left and right STG during the threat-related task [73]. In another MRI study, researchers applied voxel-based morphometry analysis and a Likert-type scale, used to measure anxiety symptoms in 177 healthy individuals and found a positive correlation between the rating of anxiety symptoms and the grey matter volume in the prefrontal cortex and MTG [74], consistent with previous findings that these regions are involved in emotion regulation and are altered in patients with anxiety disorders [75, 76].

The occipital lobe contains most of the anatomical regions of the visual cortex and contributes to visual information processing, integration, and interpretation. Occipital lobe abnormalities have been detected in patients with certain mental disorders, including anxiety disorder, bipolar disorder, compulsive disorder, major depression, and schizophrenia [77–80]. These results are consistent with our findings from the current study. Take anxiety disorder for example, the anxious response and sensory-related fear are associated with regional instability of the occipital lobe, which plays a key role in emotional experience [80, 81]. A recent meta-analysis demonstrated that patients with anxiety disorders presented increased activation in the IOG [82]. Furthermore, anxiety patients, compared to healthy individuals, showed decreased activity in the SOG during both neutral and anxiety-inducing distractors in the working memory task [83], highlighting the principle role of the occipital lobe in emotional regulation and cognitive function.

Additionally, we found that ADHD, autism spectrum disorder, and bipolar disorder demonstrate overlaps in the parietal lobe, SMG, and AG. Literature has illustrated that ADHD, autism spectrum disorder, and bipolar disorder present with social cognitive dysfunction, which generally refers to abnormalities in mental operations underlying social interactions, including the perception and interpretation of intentions, dispositions, behaviors of others, and the generation of response to these behaviors [84–86]. These disorders present abnormalities in the parietal lobe, SMG, and AG, which play a critical role in social cognition, especially in learning abilities, language development and movement representation [87, 88]. In a recent neuroimaging study, researchers applied a graph-theoretic approach to investigate the organization of structural brain networks in adults with ADHD. They found that IPL, SMG, and AG were affected at the nodal level in relation to local efficiency and clustering. Lower local efficiency of SMG was associated with higher ADHD symptom scores, and lower local clustering of SMG correlated with ADHD symptom severity [89].

Finally, we found that autism spectrum disorder and major depression display an overlap on the PCu. The PCu is a key component of the default mode network (DMN), which is closely associated with social communication and interaction, as well as patterns of stereotypic and repetitive behaviors, and theory of mind; therefore, greatly contributing to various mental disorders [90–92]. In a recent meta-analysis of abnormal resting-state function connectivity (rsFC) in autism, Wang et al. observed a decreased resting-state brain activity in several DMN regions, including the PCu [93]. The result is consistent with a previous study, in which Jann et al. found decreased rsFC in the PCu/posterior cingulate cortex areas of the DMN in children with ASD [94]. Moreover, accumulating studies have highlighted the self-reflective role of the DMN involved in major depression [95, 96].

### Differences and similarities between the proposed neuroimaging-based target protocol and literature-documented stimulation targets

Our target protocols are partly consistent with current prescriptions of scalp acupuncture or traditional acupuncture, and neuromodulation studies [2, 3, 9, 97]. In addition, our findings have extended previous knowledge on treatment of mental disorders with scalp stimulation method.

Take anxiety disorder as an example, the literature suggests that the middle line of the forehead (Ezhongxian, 1 cun long from Shenting [Governor Vessel {GV} 24] straight downward along the meridian), middle line of vertex (Dingzhongxian, from Baihui [GV 20] to Qianding [GV 21] along the midline of head), posterior temporal line (Niehouxian, from Shuaigu [GB 8] to Qubin [GB 7]), and upper-middle line of occiput (Zhenshang zhengzhongxian, from Qiangjian [GV 18] to Naohu [GV 17]) are widely recognized for alleviating anxiety using scalp acupuncture [2, 98]. Overlapping regions exist in the comparison between the neuroimaging-based targets and the literature-documented targets, involving the frontal gyrus (SFG/MFG/MedFG/IFG), temporal gyrus (MTG/STG), and occipital gyrus (MOG/IOG). In addition, we also incorporated the dlPFC and the SMA in the current neuroimaging-based protocol.

In terms of neuromodulation interventions, a recent study summarized the stimulation targets of transcranial direct current stimulation in patients with an anxiety disorder, and found that 11 of the included research studies targeted the dlPFC in the treatment of anxiety [97]. In addition, transcranial alternating current stimulation on the dlPFC and the occipital cortex have been shown in several studies to reduce the severity of anxiety symptoms [9, 10]. Furthermore, patients with anxiety disorders showed symptom remission after receiving repetitive transcranial magnetic stimulation (rTMS) on the dlPFC [14]. Most of the studies applying different neuromodulation techniques mainly used the dlPFC as the stimulation target for alleviating anxiety [9].

Nevertheless, our target protocol also includes additional brain areas such as the frontal gyrus (SFG/MFG/MedFG/IFG), the SMA, and the temporal gyrus (MTG/STG), which may expand the selection of potential targets in neuromodulation techniques for the treatment of anxiety.

We believe that the additional brain regions included in the neuroimaging-based targets reflect an enhanced understanding of the neural network involved in mental disorders and thus should be incorporated into the current scalp stimulation protocol. Further carefully designed and properly controlled studies are assuredly needed to evaluate the true potential of neuroimaging-based scalp stimulation targets.

### Limitations

There are several limitations to our study. First, the keywords we used in searching the meta-analysis literature are the umbrella terms for the disorder; subtypes of the disorder are not included in this manuscript due to 1) each of the psychological disorders may be associated with multiple subtypes, and including these subtypes would considerably complicate the manuscript. 2) the literature on some subtypes may not be extensive enough to perform the meta-analysis. Further research on specific subtypes of these mental disorders is needed. Secondly, as our protocol is based on a brain imaging meta-analysis, clinical studies are needed to validate our findings. Additional functional and anatomical analyses, such as diffusion tensor imaging (DTI) and resting-state functional connectivity (rsFC), may further enhance the proposed protocols, particularly the individualized targets for each patient. Finally, medication information is not included in the Neurosynth database; thus, we cannot exclude the potential influence of medication. Further studies are needed to validate our finding on medication-free patients.

Furthermore, the aim of this study was to explore the potential targets for mental disorders. Thus, our study may just represent one step of scalp stimulation protocols, the application and optimization of different treatment techniques (methods)/parameters/paradigms/field map to modulate these brain regions is beyond the scope of this manuscript. The optimal target selection for different scalp stimulation approaches to treat mental disorders could be an important future research endeavor. Additionally, to facilitate clinical application, we simplified the target protocol by including only eight or nine peak targets. Other brain regions may also play a critical role and should be applied in practice. Also, the locations of each target on the scalp are approximate, these locations may change when different parameters are applied or different studies are included in the analysis. Revision of the protocol will be needed as we advance our understanding of the brain physiology of mental disorders and scalp stimulation. Finally, to help the readers understand the brain function of identified areas, we have summarized the brain functions of each identified surface region associated with a corresponding mental disorder. It is worth noting that this summary may not be complete and accurate as we are still in the early stages of understanding the association between the brain and psychiatric disorders.

In summary, we have initiated an attempt to develop neuroimaging-based scalp stimulation target protocols for the treatment of eight common mental disorders. Our findings may facilitate the development and extend the clinical applications of scalp acupuncture, tES, and other neuromodulation techniques for the treatment of mental disorders.

## Supplementary Information

**Additional file 1.** Additional figures and tables.

## Data Availability

Data supporting the findings of this study are available from the corresponding author, upon reasonable request.
