# Supplementary material for: Potential scalp stimulation targets for mental disorders: evidence from neuroimaging studies
Source: J Transl Med. 2021 Aug 10;19:343. doi: 10.1186/s12967-021-02993-1 (PMC8353731; doi:10.1186/s12967-021-02993-1)
Supplement: Supplementary file 1 — Additional file 1. Additional figures and tables. [file 12967_2021_2993_MOESM1_ESM.docx]

**Additional Materials**

**Potential scalp stimulation targets for mental disorders – Evidence from neuroimaging studies**

Jin Cao^1^, Thalia Celeste Chai-Zhang^1^, Yiting Huang^1^, Maya Nicole Eshel^1^, and Jian Kong^1*^

^1^ Department of Psychiatry, Massachusetts General Hospital, Harvard Medical School, Charlestown, MA 02129, USA

**
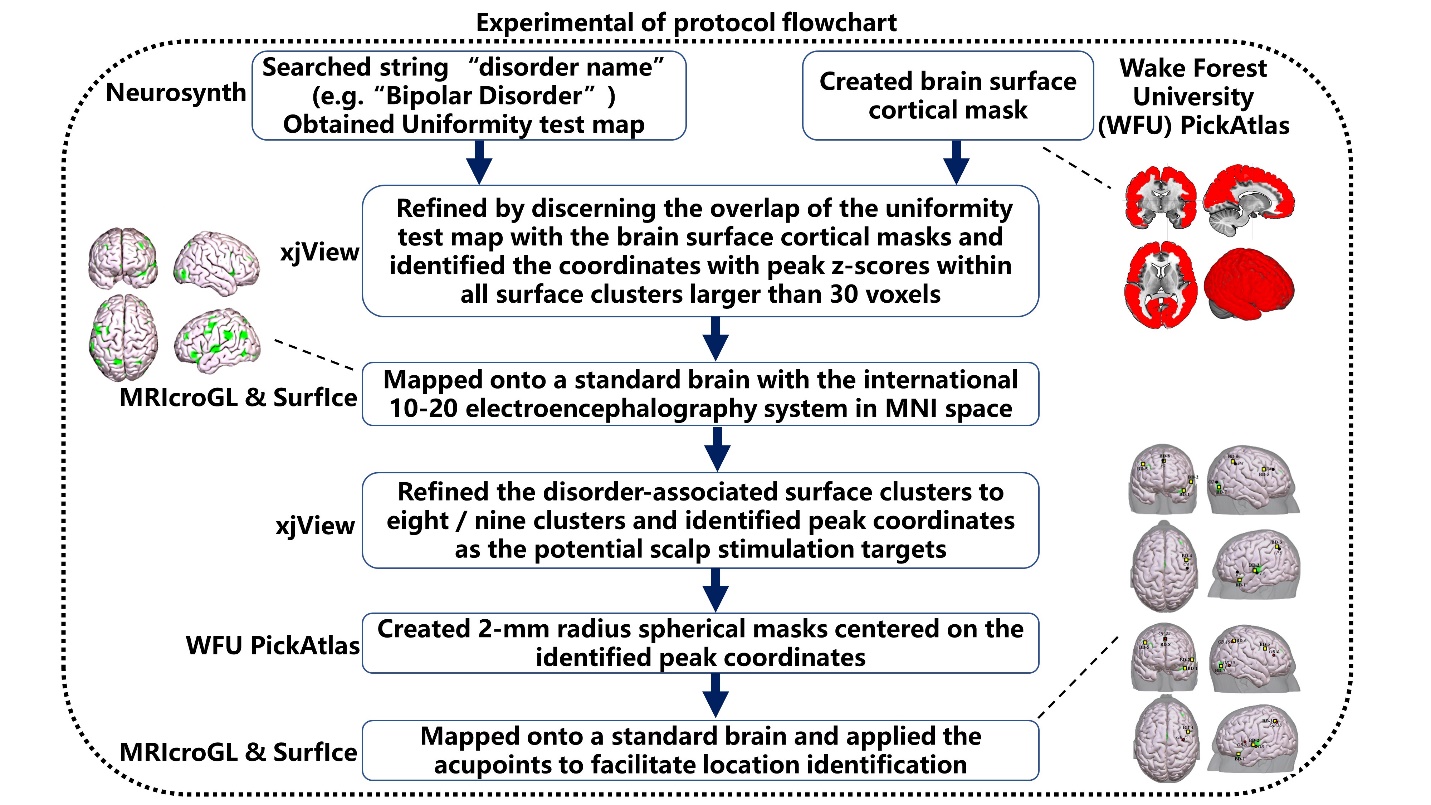
**

**Additional file 1: Figure S1.** Experimental of protocol flowchart.

**Additional file 1: Table S1.** List of 144 studies extracted from Neurosynth under search term “ADHD” (attention-deficit/hyperactivity disorder) on September 21, 2020

| **No.** | **Title** | **Authors** | **Journal** |
| --- | --- | --- | --- |
| 1 | [A multi-methodological MR resting state network analysis to assess the changes in brain physiology of children with ADHD.](https://neurosynth.org/studies/24945408/) | Alonso Bde C, Hidalgo Tobon S, Dies Suarez P, Garcia Flores J, de Celis Carrillo B, Barragan Perez E | PloS one |
| 2 | [A phenotypic structure and neural correlates of compulsive behaviors in adolescents.](https://neurosynth.org/studies/24244633/) | Montigny C, Castellanos-Ryan N, Whelan R, Banaschewski T, Barker GJ, Buchel C, Gallinat J, Flor H, Mann K, Paillere-Martinot ML, Nees F, Lathrop M, Loth E, Paus T, Pausova Z, Rietschel M, Schumann G, Smolka MN, Struve M, Robbins TW, Garavan H, Conrod PJ | PloS one |
| 3 | [A review of fronto-striatal and fronto-cortical brain abnormalities in children and adults with Attention Deficit Hyperactivity Disorder (ADHD) and new evidence for dysfunction in adults with ADHD du](https://neurosynth.org/studies/21575934/) | Cubillo A, Halari R, Smith A, Taylor E, Rubia K | Cortex; a journal devoted to the study of the nervous system and behavior |
| 4 | [Abnormal functional connectivity between the anterior cingulate and the default mode network in drug-naive boys with attention deficit hyperactivity disorder.](https://neurosynth.org/studies/22424873/) | Sun L, Cao Q, Long X, Sui M, Cao X, Zhu C, Zuo X, An L, Song Y, Zang Y, Wang Y | Psychiatry research |
| 5 | [Abnormal resting-state functional connectivity patterns of the putamen in medication-naive children with attention deficit hyperactivity disorder.](https://neurosynth.org/studies/19699190/) | Cao X, Cao Q, Long X, Sun L, Sui M, Zhu C, Zuo X, Zang Y, Wang Y | Brain research |
| 6 | [Abnormal spontaneous brain activity in medication-naive ADHD children: a resting state fMRI study.](https://neurosynth.org/studies/21810451/) | Yang H, Wu QZ, Guo LT, Li QQ, Long XY, Huang XQ, Chan RC, Gong QY | Neuroscience letters |
| 7 | [Abnormal striatal BOLD responses to reward anticipation and reward delivery in ADHD.](https://neurosynth.org/studies/24586543/) | Furukawa E, Bado P, Tripp G, Mattos P, Wickens JR, Bramati IE, Alsop B, Ferreira FM, Lima D, Tovar-Moll F, Sergeant JA, Moll J | PloS one |
| 8 | [Abnormalities of structural covariance networks in drug-naive boys with attention deficit hyperactivity disorder.](https://neurosynth.org/studies/25682468/) | Li X, Cao Q, Pu F, Li D, Fan Y, An L, Wang P, Wu Z, Sun L, Li S, Wang Y | Psychiatry research |
| 9 | [Activation in ventral prefrontal cortex is sensitive to genetic vulnerability for attention-deficit hyperactivity disorder.](https://neurosynth.org/studies/16712804/) | Durston S, Mulder M, Casey BJ, Ziermans T, van Engeland H | Biological psychiatry |
| 10 | [ADHD related behaviors are associated with brain activation in the reward system.](https://neurosynth.org/studies/21163276/) | Stark R, Bauer E, Merz CJ, Zimmermann M, Reuter M, Plichta MM, Kirsch P, Lesch KP, Fallgatter AJ, Vaitl D, Herrmann MJ | Neuropsychologia |
| 11 | [Alerting deficits in children with attention deficit/hyperactivity disorder: event-related fMRI evidence.](https://neurosynth.org/studies/18534567/) | Cao Q, Zang Y, Zhu C, Cao X, Sun L, Zhou X, Wang Y | Brain research |
| 12 | [Alterations in regional homogeneity of resting-state brain activity in autism spectrum disorders.](https://neurosynth.org/studies/20053346/) | Paakki JJ, Rahko J, Long X, Moilanen I, Tervonen O, Nikkinen J, Starck T, Remes J, Hurtig T, Haapsamo H, Jussila K, Kuusikko-Gauffin S, Mattila ML, Zang Y, Kiviniemi V | Brain research |
| 13 | [Altered cortical morphology in sensorimotor processing regions in adolescents and adults with attention-deficit/hyperactivity disorder.](https://neurosynth.org/studies/22325095/) | Duerden EG, Tannock R, Dockstader C | Brain research |
| 14 | [Altered functional brain connectivity in a non-clinical sample of young adults with attention-deficit/hyperactivity disorder.](https://neurosynth.org/studies/23223295/) | Cocchi L, Bramati IE, Zalesky A, Furukawa E, Fontenelle LF, Moll J, Tripp G, Mattos P | The Journal of neuroscience : the official journal of the Society for Neuroscience |
| 15 | [Altered neural circuits related to sustained attention and executive control in children with ADHD: an event-related fMRI study.](https://neurosynth.org/studies/23800705/) | Wang S, Yang Y, Xing W, Chen J, Liu C, Luo X | Clinical neurophysiology : official journal of the International Federation of Clinical Neurophysiology |
| 16 | [Altered neural connectivity during response inhibition in adolescents with attention-deficit/hyperactivity disorder and their unaffected siblings.](https://neurosynth.org/studies/25610797/) | van Rooij D, Hartman CA, Mennes M, Oosterlaan J, Franke B, Rommelse N, Heslenfeld D, Faraone SV, Buitelaar JK, Hoekstra PJ | NeuroImage. Clinical |
| 17 | [Altered resting-state functional connectivity patterns of anterior cingulate cortex in adolescents with attention deficit hyperactivity disorder.](https://neurosynth.org/studies/16510242/) | Tian L, Jiang T, Wang Y, Zang Y, He Y, Liang M, Sui M, Cao Q, Hu S, Peng M, Zhuo Y | Neuroscience letters |
| 18 | [Altered salience processing in attention deficit hyperactivity disorder.](https://neurosynth.org/studies/25648705/) | Tegelbeckers J, Bunzeck N, Duzel E, Bonath B, Flechtner HH, Krauel K | Human brain mapping |
| 19 | [Amygdala-prefrontal cortical functional connectivity during implicit emotion processing differentiates youth with bipolar spectrum from youth with externalizing disorders.](https://neurosynth.org/studies/27756046/) | Hafeman D, Bebko G, Bertocci MA, Fournier JC, Chase HW, Bonar L, Perlman SB, Travis M, Gill MK, Diwadkar VA, Sunshine JL, Holland SK, Kowatch RA, Birmaher B, Axelson D, Horwitz SM, Arnold LE, Fristad MA, Frazier TW, Youngstrom EA, Findling RL, Phillips ML | Journal of affective disorders |
| 20 | [Anomalous prefrontal-limbic activation and connectivity in youth at high-risk for bipolar disorder.](https://neurosynth.org/studies/28667891/) | Chang K, Garrett A, Kelley R, Howe M, Sanders EM, Acquaye T, Bararpour L, Li S, Singh M, Jo B, Hallmayer J, Reiss A | Journal of affective disorders |
| 21 | [Anterior cingulate cortex dysfunction in attention-deficit/hyperactivity disorder revealed by fMRI and the Counting Stroop.](https://neurosynth.org/studies/10376114/) | Bush G, Frazier JA, Rauch SL, Seidman LJ, Whalen PJ, Jenike MA, Rosen BR, Biederman J | Biological psychiatry |
| 22 | [Antisaccade-related brain activation in children with attention-deficit/hyperactivity disorder--A pilot study.](https://neurosynth.org/studies/26459074/) | Schwarz NF, Krafft CE, Chi L, Weinberger AL, Schaeffer DJ, Pierce JE, Rodrigue AL, Williams CF, DiBattisto CH, Maria BL, Davis CL, McDowell JE | Psychiatry research |
| 23 | [Atomoxetine increases fronto-parietal functional MRI activation in attention-deficit/hyperactivity disorder: a pilot study.](https://neurosynth.org/studies/23146254/) | Bush G, Holmes J, Shin LM, Surman C, Makris N, Mick E, Seidman LJ, Biederman J | Psychiatry research |
| 24 | [Attention Network Dysfunction in Bulimia Nervosa - An fMRI Study.](https://neurosynth.org/studies/27607439/) | Seitz J, Hueck M, Dahmen B, Schulte-Ruther M, Legenbauer T, Herpertz-Dahlmann B, Konrad K | PloS one |
| 25 | [Attention-deficit/hyperactivity disorder in childhood epilepsy: a neuropsychological and functional imaging study.](https://neurosynth.org/studies/22242637/) | Bechtel N, Kobel M, Penner IK, Specht K, Klarhofer M, Scheffler K, Opwis K, Schmitt-Mechelke T, Capone A, Weber P | Epilepsia |
| 26 | [Attentional control activation relates to working memory in attention-deficit/hyperactivity disorder.](https://neurosynth.org/studies/20060961/) | Burgess GC, Depue BE, Ruzic L, Willcutt EG, Du YP, Banich MT | Biological psychiatry |
| 27 | [Atypical default network connectivity in youth with attention-deficit/hyperactivity disorder.](https://neurosynth.org/studies/20728873/) | Fair DA, Posner J, Nagel BJ, Bathula D, Dias TG, Mills KL, Blythe MS, Giwa A, Schmitt CF, Nigg JT | Biological psychiatry |
| 28 | [Atypical motor and sensory cortex activation in attention-deficit/hyperactivity disorder: a functional magnetic resonance imaging study of simple sequential finger tapping.](https://neurosynth.org/studies/16139806/) | Mostofsky SH, Rimrodt SL, Schafer JG, Boyce A, Goldberg MC, Pekar JJ, Denckla MB | Biological psychiatry |
| 29 | [Atypical within- and between-hemisphere motor network functional connections in children with developmental coordination disorder and attention-deficit/hyperactivity disorder.](https://neurosynth.org/studies/27419066/) | McLeod KR, Langevin LM, Dewey D, Goodyear BG | NeuroImage. Clinical |
| 30 | [Behavioural treatment increases activity in the cognitive neuronal networks in children with attention deficit/hyperactivity disorder.](https://neurosynth.org/studies/22392009/) | Siniatchkin M, Glatthaar N, von Muller GG, Prehn-Kristensen A, Wolff S, Knochel S, Steinmann E, Sotnikova A, Stephani U, Petermann F, Gerber WD | Brain topography |
| 31 | [Boys with conduct problems and callous-unemotional traits: Neural response to reward and punishment and associations with treatment response.](https://neurosynth.org/studies/29324299/) | Byrd AL, Hawes SW, Burke JD, Loeber R, Pardini DA | Developmental cognitive neuroscience |
| 32 | [Brain activation deficit in increased-load working memory tasks among adults with ADHD using fMRI.](https://neurosynth.org/studies/23645101/) | Ko CH, Yen JY, Yen CF, Chen CS, Lin WC, Wang PW, Liu GC | European archives of psychiatry and clinical neuroscience |
| 33 | [Brain activation to cues predicting inescapable delay in adolescent Attention Deficit/Hyperactivity Disorder: an fMRI pilot study.](https://neurosynth.org/studies/22406068/) | Lemiere J, Danckaerts M, Van Hecke W, Mehta MA, Peeters R, Sunaert S, Sonuga-Barke E | Brain research |
| 34 | [Brain morphology in children with epilepsy and ADHD.](https://neurosynth.org/studies/24760032/) | Saute R, Dabbs K, Jones JE, Jackson DC, Seidenberg M, Hermann BP | PloS one |
| 35 | [Childhood methylphenidate treatment of ADHD and response to affective stimuli.](https://neurosynth.org/studies/20570115/) | Schlochtermeier L, Stoy M, Schlagenhauf F, Wrase J, Park SQ, Friedel E, Huss M, Lehmkuhl U, Heinz A, Strohle A | European neuropsychopharmacology : the journal of the European College of Neuropsychopharmacology |
| 36 | [Cingulate-precuneus interactions: a new locus of dysfunction in adult attention-deficit/hyperactivity disorder.](https://neurosynth.org/studies/17888409/) | Castellanos FX, Margulies DS, Kelly C, Uddin LQ, Ghaffari M, Kirsch A, Shaw D, Shehzad Z, Di Martino A, Biswal B, Sonuga-Barke EJ, Rotrosen J, Adler LA, Milham MP | Biological psychiatry |
| 37 | [Cognitive and neural correlates of the 5-repeat allele of the dopamine D4 receptor gene in a population lacking the 7-repeat allele.](https://neurosynth.org/studies/25659462/) | Takeuchi H, Tomita H, Taki Y, Kikuchi Y, Ono C, Yu Z, Sekiguchi A, Nouchi R, Kotozaki Y, Nakagawa S, Miyauchi CM, Iizuka K, Yokoyama R, Shinada T, Yamamoto Y, Hanawa S, Araki T, Hashizume H, Kunitoki K, Sassa Y, Kawashima R | NeuroImage |
| 38 | [Connectivity supporting attention in children with attention deficit hyperactivity disorder.](https://neurosynth.org/studies/25610768/) | Barber AD, Jacobson LA, Wexler JL, Nebel MB, Caffo BS, Pekar JJ, Mostofsky SH | NeuroImage. Clinical |
| 39 | [Cortical thickness, mental absorption and meditative practice: possible implications for disorders of attention.](https://neurosynth.org/studies/23046904/) | Grant JA, Duerden EG, Courtemanche J, Cherkasova M, Duncan GH, Rainville P | Biological psychology |
| 40 | [Default mode network activity and neuropsychological profile in male children and adolescents with attention deficit hyperactivity disorder and conduct disorder.](https://neurosynth.org/studies/27738997/) | Uytun MC, Karakaya E, Oztop DB, Gengec S, Gumus K, Ozmen S, Doganay S, Icer S, Demirci E, Ozsoy SD | Brain imaging and behavior |
| 41 | [Differences in feedback- and inhibition-related neural activity in adult ADHD.](https://neurosynth.org/studies/19201515/) | Dibbets P, Evers L, Hurks P, Marchetta N, Jolles J | Brain and cognition |
| 42 | [Differences in functional activity between boys with pure oppositional defiant disorder and controls during a response inhibition task: a preliminary study.](https://neurosynth.org/studies/24390655/) | Zhu Y, Ying K, Wang J, Su L, Chen J, Lin F, Cai D, Zhou M, Wu D, Guo C, Wang S | Brain imaging and behavior |
| 43 | [Differential Associations between Cortical Thickness and Striatal Dopamine in Treatment-Naive Adults with ADHD vs. Healthy Controls.](https://neurosynth.org/studies/28878639/) | Cherkasova MV, Faridi N, Casey KF, Larcher K, O'Driscoll GA, Hechtman L, Joober R, Baker GB, Palmer J, Evans AC, Dagher A, Benkelfat C, Leyton M | Frontiers in human neuroscience |
| 44 | [Differential fractional anisotropy abnormalities in adolescents with ADHD or schizophrenia.](https://neurosynth.org/studies/20153608/) | Davenport ND, Karatekin C, White T, Lim KO | Psychiatry research |
| 45 | [Differential patterns of striatal activation in young children with and without ADHD.](https://neurosynth.org/studies/12742674/) | Durston S, Tottenham NT, Thomas KM, Davidson MC, Eigsti IM, Yang Y, Ulug AM, Casey BJ | Biological psychiatry |
| 46 | [Differential therapeutic effects of 12-week treatment of atomoxetine and methylphenidate on drug-naive children with attention deficit/hyperactivity disorder: A counting Stroop functional MRI study.](https://neurosynth.org/studies/26409297/) | Chou TL, Chia S, Shang CY, Gau SS | European neuropsychopharmacology : the journal of the European College of Neuropsychopharmacology |
| 47 | [Differentiating neural reward responsiveness in autism versus ADHD.](https://neurosynth.org/studies/25190643/) | Kohls G, Thonessen H, Bartley GK, Grossheinrich N, Fink GR, Herpertz-Dahlmann B, Konrad K | Developmental cognitive neuroscience |
| 48 | [Dimensional brain-behavior relationships in children with attention-deficit/hyperactivity disorder.](https://neurosynth.org/studies/21974788/) | Chabernaud C, Mennes M, Kelly C, Nooner K, Di Martino A, Castellanos FX, Milham MP | Biological psychiatry |
| 49 | [Disorder-specific dysfunction in right inferior prefrontal cortex during two inhibition tasks in boys with attention-deficit hyperactivity disorder compared to boys with obsessive-compulsive disorder.](https://neurosynth.org/studies/19777552/) | Rubia K, Cubillo A, Smith AB, Woolley J, Heyman I, Brammer MJ | Human brain mapping |
| 50 | [Disorder-specific dysfunctions in patients with attention-deficit/hyperactivity disorder compared to patients with obsessive-compulsive disorder during interference inhibition and attention allocation](https://neurosynth.org/studies/21391250/) | Rubia K, Cubillo A, Woolley J, Brammer MJ, Smith A | Human brain mapping |
| 51 | [Disorder-specific predictive classification of adolescents with attention deficit hyperactivity disorder (ADHD) relative to autism using structural magnetic resonance imaging.](https://neurosynth.org/studies/23696841/) | Lim L, Marquand A, Cubillo AA, Smith AB, Chantiluke K, Simmons A, Mehta M, Rubia K | PloS one |
| 52 | [Disturbed structural connectivity is related to inattention and impulsivity in adult attention deficit hyperactivity disorder.](https://neurosynth.org/studies/20374289/) | Konrad A, Dielentheis TF, El Masri D, Bayerl M, Fehr C, Gesierich T, Vucurevic G, Stoeter P, Winterer G | The European journal of neuroscience |
| 53 | [Dopamine transporter gene variation modulates activation of striatum in youth with ADHD.](https://neurosynth.org/studies/20026227/) | Bedard AC, Schulz KP, Cook EH Jr, Fan J, Clerkin SM, Ivanov I, Halperin JM, Newcorn JH | NeuroImage |
| 54 | [Dopaminergic dysfunction in abstinent dexamphetamine users: results from a pharmacological fMRI study using a reward anticipation task and a methylphenidate challenge.](https://neurosynth.org/studies/23142493/) | Schouw ML, De Ruiter MB, Kaag AM, van den Brink W, Lindauer RJ, Reneman L | Drug and alcohol dependence |
| 55 | [Effects of methylphenidate during emotional processing in amphetamine users: preliminary findings.](https://neurosynth.org/studies/25563230/) | Bottelier MA, Schouw ML, de Ruiter MB, Ruhe HG, Lindauer RJ, Reneman L | Brain imaging and behavior |
| 56 | [Effects of Stimulants on Brain Function in Attention-Deficit/Hyperactivity Disorder: A Systematic Review and Meta-Analysis.](https://neurosynth.org/studies/24314347/) | Rubia K, Alegria AA, Cubillo AI, Smith AB, Brammer MJ, Radua J | Biological psychiatry |
| 57 | [Effects of the DRD4 genotype on neural networks associated with executive functions in children and adolescents.](https://neurosynth.org/studies/22727763/) | Gilsbach S, Neufang S, Scherag S, Vloet TD, Fink GR, Herpertz-Dahlmann B, Konrad K | Developmental cognitive neuroscience |
| 58 | [Enhanced neural activity in frontal and cerebellar circuits after cognitive training in children with attention-deficit/hyperactivity disorder.](https://neurosynth.org/studies/20336653/) | Hoekzema E, Carmona S, Tremols V, Gispert JD, Guitart M, Fauquet J, Rovira M, Bielsa A, Soliva JC, Tomas X, Bulbena A, Ramos-Quiroga A, Casas M, Tobena A, Vilarroya O | Human brain mapping |
| 59 | [Executive attention control and emotional responding in attention-deficit/hyperactivity disorder--A functional MRI study.](https://neurosynth.org/studies/26640766/) | Hwang S, White SF, Nolan ZT, Craig Williams W, Sinclair S, Blair RJ | NeuroImage. Clinical |
| 60 | [Exploiting the brain's network structure in identifying ADHD subjects.](https://neurosynth.org/studies/23162440/) | Dey S, Rao AR, Shah M | Frontiers in systems neuroscience |
| 61 | [Familial risk and ADHD-specific neural activity revealed by case-control, discordant twin pair design.](https://neurosynth.org/studies/26256128/) | Godinez DA, Willcutt EG, Burgess GC, Depue BE, Andrews-Hanna JR, Banich MT | Psychiatry research |
| 62 | [Feedback associated with expectation for larger-reward improves visuospatial working memory performances in children with ADHD.](https://neurosynth.org/studies/26142072/) | Hammer R, Tennekoon M, Cooke GE, Gayda J, Stein MA, Booth JR | Developmental cognitive neuroscience |
| 63 | [Fluid reasoning deficits in children with ADHD: evidence from fMRI.](https://neurosynth.org/studies/22613230/) | Tamm L, Juranek J | Brain research |
| 64 | [fMRI activation during response inhibition and error processing: the role of the DAT1 gene in typically developing adolescents and those diagnosed with ADHD.](https://neurosynth.org/studies/21232548/) | Braet W, Johnson KA, Tobin CT, Acheson R, McDonnell C, Hawi Z, Barry E, Mulligan A, Gill M, Bellgrove MA, Robertson IH, Garavan H | Neuropsychologia |
| 65 | [Fronto-striatal underactivation during interference inhibition and attention allocation in grown up children with attention deficit/hyperactivity disorder and persistent symptoms.](https://neurosynth.org/studies/21601434/) | Cubillo A, Halari R, Giampietro V, Taylor E, Rubia K | Psychiatry research |
| 66 | [Frontostriatal neuroimaging findings differ in patients with bipolar disorder who have or do not have ADHD comorbidity.](https://neurosynth.org/studies/23057969/) | Townsend JD, Sugar CA, Walshaw PD, Vasquez RE, Foland-Ross LC, Moody TD, Bookheimer SY, McGough JJ, Altshuler LL | Journal of affective disorders |
| 67 | [Functional brain correlates of response time variability in children.](https://neurosynth.org/studies/17350054/) | Simmonds DJ, Fotedar SG, Suskauer SJ, Pekar JJ, Denckla MB, Mostofsky SH | Neuropsychologia |
| 68 | [Functional connectivity of substantia nigra and ventral tegmental area: maturation during adolescence and effects of ADHD.](https://neurosynth.org/studies/23242198/) | Tomasi D, Volkow ND | Cerebral cortex (New York, N.Y. : 1991) |
| 69 | [Functional neuroanatomy of working memory in adults with attention-deficit/hyperactivity disorder.](https://neurosynth.org/studies/15737657/) | Valera EM, Faraone SV, Biederman J, Poldrack RA, Seidman LJ | Biological psychiatry |
| 70 | [Grey matter volumes in treatment naive vs. chronically treated children with attention deficit/hyperactivity disorder: a combined approach.](https://neurosynth.org/studies/25934396/) | Villemonteix T, De Brito SA, Kavec M, Baleriaux D, Metens T, Slama H, Baijot S, Mary A, Peigneux P, Massat I | European neuropsychopharmacology : the journal of the European College of Neuropsychopharmacology |
| 71 | [Hypoactivation in right inferior frontal cortex is specifically associated with motor response inhibition in adult ADHD.](https://neurosynth.org/studies/24819224/) | Morein-Zamir S, Dodds C, van Hartevelt TJ, Schwarzkopf W, Sahakian B, Muller U, Robbins T | Human brain mapping |
| 72 | [Impact of early life adversity on reward processing in young adults: EEG-fMRI results from a prospective study over 25 years.](https://neurosynth.org/studies/25118701/) | Boecker R, Holz NE, Buchmann AF, Blomeyer D, Plichta MM, Wolf I, Baumeister S, Meyer-Lindenberg A, Banaschewski T, Brandeis D, Laucht M | PloS one |
| 73 | [Impact of emotional salience on episodic memory in attention-deficit/hyperactivity disorder: a functional magnetic resonance imaging study.](https://neurosynth.org/studies/17210138/) | Krauel K, Duzel E, Hinrichs H, Santel S, Rellum T, Baving L | Biological psychiatry |
| 74 | [Impairment of fronto-striatal and parietal cerebral networks correlates with attention deficit hyperactivity disorder (ADHD) psychopathology in adults - a functional magnetic resonance imaging (fMRI)](https://neurosynth.org/studies/20558047/) | Schneider MF, Krick CM, Retz W, Hengesch G, Retz-Junginger P, Reith W, Rosler M | Psychiatry research |
| 75 | [Inconsistency in Abnormal Brain Activity across Cohorts of ADHD-200 in Children with Attention Deficit Hyperactivity Disorder.](https://neurosynth.org/studies/28634439/) | Wang JB, Zheng LJ, Cao QJ, Wang YF, Sun L, Zang YF, Zhang H | Frontiers in neuroscience |
| 76 | [Increased cerebral perfusion in adult attention deficit hyperactivity disorder is normalised by stimulant treatment: a non-invasive MRI pilot study.](https://neurosynth.org/studies/18511306/) | O'Gorman RL, Mehta MA, Asherson P, Zelaya FO, Brookes KJ, Toone BK, Alsop DC, Williams SC | NeuroImage |
| 77 | [Interaction between COMT Val(158)Met polymorphism and childhood adversity affects reward processing in adulthood.](https://neurosynth.org/studies/26879624/) | Boecker-Schlier R, Holz NE, Buchmann AF, Blomeyer D, Plichta MM, Jennen-Steinmetz C, Wolf I, Baumeister S, Treutlein J, Rietschel M, Meyer-Lindenberg A, Banaschewski T, Brandeis D, Laucht M | NeuroImage |
| 78 | [Intergenerational transmission of fronto-parietal dysfunction during forethought in attention deficit/hyperactivity disorder: a pilot study.](https://neurosynth.org/studies/25443178/) | Poissant H, Rapin L, Mendrek A | Psychiatry research |
| 79 | [Inverse fluoxetine effects on inhibitory brain activation in non-comorbid boys with ADHD and with ASD.](https://neurosynth.org/studies/25533997/) | Chantiluke K, Barrett N, Giampietro V, Santosh P, Brammer M, Simmons A, Murphy DG, Rubia K | Psychopharmacology |
| 80 | [Laminar thickness alterations in the fronto-parietal cortical mantle of patients with attention-deficit/hyperactivity disorder.](https://neurosynth.org/studies/23239964/) | Hoekzema E, Carmona S, Ramos-Quiroga JA, Richarte Fernandez V, Picado M, Bosch R, Soliva JC, Rovira M, Vives Y, Bulbena A, Tobena A, Casas M, Vilarroya O | PloS one |
| 81 | [Mapping brain structure in attention deficit-hyperactivity disorder: a voxel-based MRI study of regional grey and white matter volume.](https://neurosynth.org/studies/17291727/) | McAlonan GM, Cheung V, Cheung C, Chua SE, Murphy DG, Suckling J, Tai KS, Yip LK, Leung P, Ho TP | Psychiatry research |
| 82 | [Methylphenidate does not improve interference control during a working memory task in young patients with attention-deficit hyperactivity disorder.](https://neurosynth.org/studies/21385569/) | Prehn-Kristensen A, Krauel K, Hinrichs H, Fischer J, Malecki U, Schuetze H, Wolff S, Jansen O, Duezel E, Baving L | Brain research |
| 83 | [Methylphenidate normalizes frontocingulate underactivation during error processing in attention-deficit/hyperactivity disorder.](https://neurosynth.org/studies/21664605/) | Rubia K, Halari R, Mohammad AM, Taylor E, Brammer M | Biological psychiatry |
| 84 | [Modality-spanning deficits in attention-deficit/hyperactivity disorder in functional networks, gray matter, and white matter.](https://neurosynth.org/studies/25505309/) | Kessler D, Angstadt M, Welsh RC, Sripada C | The Journal of neuroscience : the official journal of the Society for Neuroscience |
| 85 | [Morphological abnormalities in prefrontal surface area and thalamic volume in attention deficit/hyperactivity disorder.](https://neurosynth.org/studies/26190555/) | Batty MJ, Palaniyappan L, Scerif G, Groom MJ, Liddle EB, Liddle PF, Hollis C | Psychiatry research |
| 86 | [Morphometric correlation of impulsivity in medial prefrontal cortex.](https://neurosynth.org/studies/23274773/) | Cho SS, Pellecchia G, Aminian K, Ray N, Segura B, Obeso I, Strafella AP | Brain topography |
| 87 | [MR imaging of the effects of methylphenidate on brain structure and function in attention-deficit/hyperactivity disorder.](https://neurosynth.org/studies/23165220/) | Schweren LJ, de Zeeuw P, Durston S | European neuropsychopharmacology : the journal of the European College of Neuropsychopharmacology |
| 88 | [Multi-modal, Multi-measure, and Multi-class Discrimination of ADHD with Hierarchical Feature Extraction and Extreme Learning Machine Using Structural and Functional Brain MRI.](https://neurosynth.org/studies/28420972/) | Qureshi MNI, Oh J, Min B, Jo HJ, Lee B | Frontiers in human neuroscience |
| 89 | [Neural activation during response inhibition in adult attention-deficit/hyperactivity disorder: preliminary findings on the effects of medication and symptom severity.](https://neurosynth.org/studies/24581734/) | Congdon E, Altshuler LL, Mumford JA, Karlsgodt KH, Sabb FW, Ventura J, McGough JJ, London ED, Cannon TD, Bilder RM, Poldrack RA | Psychiatry research |
| 90 | [Neural basis of reward anticipation and its genetic determinants.](https://neurosynth.org/studies/27001827/) | Jia T, Macare C, Desrivieres S, Gonzalez DA, Tao C, Ji X, Ruggeri B, Nees F, Banaschewski T, Barker GJ, Bokde AL, Bromberg U, Buchel C, Conrod PJ, Dove R, Frouin V, Gallinat J, Garavan H, Gowland PA, Heinz A, Ittermann B, Lathrop M, Lemaitre H, Martinot JL, Paus T, Pausova Z, Poline JB, Rietschel M, Robbins T, Smolka MN, Muller CP, Feng J, Rothenfluh A, Flor H, Schumann G | Proceedings of the National Academy of Sciences of the United States of America |
| 91 | [Neural circuitry underlying sustained attention in healthy adolescents and in ADHD symptomatology.](https://neurosynth.org/studies/29274748/) | O'Halloran L, Cao Z, Ruddy K, Jollans L, Albaugh MD, Aleni A, Potter AS, Vahey N, Banaschewski T, Hohmann S, Bokde ALW, Bromberg U, Buchel C, Quinlan EB, Desrivieres S, Flor H, Frouin V, Gowland P, Heinz A, Ittermann B, Nees F, Orfanos DP, Paus T, Smolka MN, Walter H, Schumann G, Garavan H, Kelly C, Whelan R | NeuroImage |
| 92 | [Neural correlates (ERP/fMRI) of voluntary selection in adult ADHD patients.](https://neurosynth.org/studies/19907927/) | Karch S, Thalmeier T, Lutz J, Cerovecki A, Opgen-Rhein M, Hock B, Leicht G, Hennig-Fast K, Meindl T, Riedel M, Mulert C, Pogarell O | European archives of psychiatry and clinical neuroscience |
| 93 | [Neural correlates of inhibitory control in adult attention deficit/hyperactivity disorder: evidence from the Milwaukee longitudinal sample.](https://neurosynth.org/studies/21937201/) | Mulligan RC, Knopik VS, Sweet LH, Fischer M, Seidenberg M, Rao SM | Psychiatry research |
| 94 | [Neural correlates of interference inhibition, action withholding and action cancelation in adult ADHD.](https://neurosynth.org/studies/22475505/) | Sebastian A, Gerdes B, Feige B, Kloppel S, Lange T, Philipsen A, Tebartz van Elst L, Lieb K, Tuscher O | Psychiatry research |
| 95 | [Neural correlates of response inhibition in children with attention-deficit/hyperactivity disorder: A controlled version of the stop-signal task.](https://neurosynth.org/studies/26195296/) | Janssen TW, Heslenfeld DJ, van Mourik R, Logan GD, Oosterlaan J | Psychiatry research |
| 96 | [Neural correlates of response inhibition in pediatric bipolar disorder and attention deficit hyperactivity disorder.](https://neurosynth.org/studies/19926457/) | Passarotti AM, Sweeney JA, Pavuluri MN | Psychiatry research |
| 97 | [Neural correlates of visuospatial working memory in attention-deficit/hyperactivity disorder and healthy controls.](https://neurosynth.org/studies/26190554/) | van Ewijk H, Weeda WD, Heslenfeld DJ, Luman M, Hartman CA, Hoekstra PJ, Faraone SV, Franke B, Buitelaar JK, Oosterlaan J | Psychiatry research |
| 98 | [Neural dysfunction during temporal discounting in paediatric Attention-Deficit/Hyperactivity Disorder and Obsessive-Compulsive Disorder.](https://neurosynth.org/studies/28988149/) | Norman LJ, Carlisi CO, Christakou A, Chantiluke K, Murphy C, Simmons A, Giampietro V, Brammer M, Mataix-Cols D, Rubia K | Psychiatry research. Neuroimaging |
| 99 | [Neural dysfunction in ADHD with Reading Disability during a word rhyming Continuous Performance Task.](https://neurosynth.org/studies/26188845/) | Mohl B, Ofen N, Jones LL, Robin AL, Rosenberg DR, Diwadkar VA, Casey JE, Stanley JA | Brain and cognition |
| 100 | [Neural recruitment during failed motor inhibition differentiates youths with bipolar disorder and severe mood dysregulation.](https://neurosynth.org/studies/22008364/) | Deveney CM, Connolly ME, Jenkins SE, Kim P, Fromm SJ, Pine DS, Leibenluft E | Biological psychology |
| 101 | [Neural substrates of impaired sensorimotor timing in adult attention-deficit/hyperactivity disorder.](https://neurosynth.org/studies/20619827/) | Valera EM, Spencer RM, Zeffiro TA, Makris N, Spencer TJ, Faraone SV, Biederman J, Seidman LJ | Biological psychiatry |
| 102 | [Neuroanatomical deficits correlate with executive dysfunction in boys with attention deficit hyperactivity disorder.](https://neurosynth.org/studies/26049007/) | He N, Li F, Li Y, Guo L, Chen L, Huang X, Lui S, Gong Q | Neuroscience letters |
| 103 | [Neurofunctional effects of methylphenidate and atomoxetine in boys with attention-deficit/hyperactivity disorder during time discrimination.](https://neurosynth.org/studies/23731741/) | Smith A, Cubillo A, Barrett N, Giampietro V, Simmons A, Brammer M, Rubia K | Biological psychiatry |
| 104 | [Orbitofrontal reward sensitivity and impulsivity in adult attention deficit hyperactivity disorder.](https://neurosynth.org/studies/22197790/) | Wilbertz G, van Elst LT, Delgado MR, Maier S, Feige B, Philipsen A, Blechert J | NeuroImage |
| 105 | [Organizing heterogeneous samples using community detection of GIMME-derived resting state functional networks.](https://neurosynth.org/studies/24642753/) | Gates KM, Molenaar PC, Iyer SP, Nigg JT, Fair DA | PloS one |
| 106 | [Parental substance abuse and function of the motivation and behavioral inhibition systems in drug-naive youth.](https://neurosynth.org/studies/22386967/) | Ivanov I, Liu X, Shulz K, Fan J, London E, Friston K, Halperin JM, Newcorn JH | Psychiatry research |
| 107 | [Pattern classification of response inhibition in ADHD: Toward the development of neurobiological markers for ADHD.](https://neurosynth.org/studies/24123508/) | Hart H, Chantiluke K, Cubillo AI, Smith AB, Simmons A, Brammer MJ, Marquand AF, Rubia K | Human brain mapping |
| 108 | [Polygenic risk for five psychiatric disorders and cross-disorder and disorder-specific neural connectivity in two independent populations.](https://neurosynth.org/studies/28275544/) | Wang T, Zhang X, Li A, Zhu M, Liu S, Qin W, Li J, Yu C, Jiang T, Liu B | NeuroImage. Clinical |
| 109 | [Prediction of brain maturity based on cortical thickness at different spatial resolutions.](https://neurosynth.org/studies/25731999/) | Khundrakpam BS, Tohka J, Evans AC | NeuroImage |
| 110 | [Prefrontal and parietal correlates of cognitive control related to the adult outcome of attention-deficit/hyperactivity disorder diagnosed in childhood.](https://neurosynth.org/studies/28292705/) | Schulz KP, Li X, Clerkin SM, Fan J, Berwid OG, Newcorn JH, Halperin JM | Cortex; a journal devoted to the study of the nervous system and behavior |
| 111 | [Reduced cortical gray matter volume in male adolescents with substance and conduct problems.](https://neurosynth.org/studies/21592680/) | Dalwani M, Sakai JT, Mikulich-Gilbertson SK, Tanabe J, Raymond K, McWilliams SK, Thompson LL, Banich MT, Crowley TJ | Drug and alcohol dependence |
| 112 | [Regional brain activation changes and abnormal functional connectivity of the ventrolateral prefrontal cortex during working memory processing in adults with attention-deficit/hyperactivity disorder.](https://neurosynth.org/studies/19107748/) | Wolf RC, Plichta MM, Sambataro F, Fallgatter AJ, Jacob C, Lesch KP, Herrmann MJ, Schonfeldt-Lecuona C, Connemann BJ, Gron G, Vasic N | Human brain mapping |
| 113 | [Relationship of DAT1 and adult ADHD to task-positive and task-negative working memory networks.](https://neurosynth.org/studies/21596533/) | Brown AB, Biederman J, Valera E, Makris N, Doyle A, Whitfield-Gabrieli S, Mick E, Spencer T, Faraone S, Seidman L | Psychiatry research |
| 114 | [Resting state fMRI entropy probes complexity of brain activity in adults with ADHD.](https://neurosynth.org/studies/24183857/) | Sokunbi MO, Fung W, Sawlani V, Choppin S, Linden DE, Thome J | Psychiatry research |
| 115 | [Reward anticipation and outcomes in adult males with attention-deficit/hyperactivity disorder.](https://neurosynth.org/studies/17996464/) | Strohle A, Stoy M, Wrase J, Schwarzer S, Schlagenhauf F, Huss M, Hein J, Nedderhut A, Neumann B, Gregor A, Juckel G, Knutson B, Lehmkuhl U, Bauer M, Heinz A | NeuroImage |
| 116 | [Reward circuit connectivity relates to delay discounting in children with attention-deficit/hyperactivity disorder.](https://neurosynth.org/studies/23206930/) | Costa Dias TG, Wilson VB, Bathula DR, Iyer SP, Mills KL, Thurlow BL, Stevens CA, Musser ED, Carpenter SD, Grayson DS, Mitchell SH, Nigg JT, Fair DA | European neuropsychopharmacology : the journal of the European College of Neuropsychopharmacology |
| 117 | [Reward processing in male adults with childhood ADHD--a comparison between drug-naive and methylphenidate-treated subjects.](https://neurosynth.org/studies/21298512/) | Stoy M, Schlagenhauf F, Schlochtermeier L, Wrase J, Knutson B, Lehmkuhl U, Huss M, Heinz A, Strohle A | Psychopharmacology |
| 118 | [Sex differences in anterior cingulate cortex activation during impulse inhibition and behavioral correlates.](https://neurosynth.org/studies/22285718/) | Liu J, Zubieta JK, Heitzeg M | Psychiatry research |
| 119 | [Shared and disorder-specific task-positive and default mode network dysfunctions during sustained attention in paediatric Attention-Deficit/Hyperactivity Disorder and obsessive/compulsive disorder.](https://neurosynth.org/studies/28529874/) | Norman LJ, Carlisi CO, Christakou A, Cubillo A, Murphy CM, Chantiluke K, Simmons A, Giampietro V, Brammer M, Mataix-Cols D, Rubia K | NeuroImage. Clinical |
| 120 | [Shared and drug-specific effects of atomoxetine and methylphenidate on inhibitory brain dysfunction in medication-naive ADHD boys.](https://neurosynth.org/studies/23048018/) | Cubillo A, Smith AB, Barrett N, Giampietro V, Brammer MJ, Simmons A, Rubia K | Cerebral cortex (New York, N.Y. : 1991) |
| 121 | [Short-term test-retest reliability of resting state fMRI metrics in children with and without attention-deficit/hyperactivity disorder.](https://neurosynth.org/studies/26365788/) | Somandepalli K, Kelly C, Reiss PT, Zuo XN, Craddock RC, Yan CG, Petkova E, Castellanos FX, Milham MP, Di Martino A | Developmental cognitive neuroscience |
| 122 | [Structural and functional imaging approaches in attention deficit/hyperactivity disorder: does the temporal lobe play a key role?](https://neurosynth.org/studies/20702071/) | Kobel M, Bechtel N, Specht K, Klarhofer M, Weber P, Scheffler K, Opwis K, Penner IK | Psychiatry research |
| 123 | [Structural brain imaging of attention-deficit/hyperactivity disorder.](https://neurosynth.org/studies/15949998/) | Seidman LJ, Valera EM, Makris N | Biological psychiatry |
| 124 | [Symptom-correlated brain regions in young adults with combined-type ADHD: their organization, variability, and relation to behavioral performance.](https://neurosynth.org/studies/20399622/) | Depue BE, Burgess GC, Willcutt EG, Bidwell LC, Ruzic L, Banich MT | Psychiatry research |
| 125 | [Temporal information processing in ADHD: findings to date and new methods.](https://neurosynth.org/studies/16378641/) | Toplak ME, Dockstader C, Tannock R | Journal of neuroscience methods |
| 126 | [Temporal lobe dysfunction in medication-naive boys with attention-deficit/hyperactivity disorder during attention allocation and its relation to response variability.](https://neurosynth.org/studies/17585887/) | Rubia K, Smith AB, Brammer MJ, Taylor E | Biological psychiatry |
| 127 | [The attenuation of dysfunctional emotional processing with stimulant medication: an fMRI study of adolescents with ADHD.](https://neurosynth.org/studies/21778039/) | Posner J, Maia TV, Fair D, Peterson BS, Sonuga-Barke EJ, Nagel BJ | Psychiatry research |
| 128 | [The dopamine transporter haplotype and reward-related striatal responses in adult ADHD.](https://neurosynth.org/studies/22749356/) | Hoogman M, Onnink M, Cools R, Aarts E, Kan C, Arias Vasquez A, Buitelaar J, Franke B | European neuropsychopharmacology : the journal of the European College of Neuropsychopharmacology |
| 129 | [The effects of stimulant medication on working memory functional connectivity in attention-deficit/hyperactivity disorder.](https://neurosynth.org/studies/22209640/) | Wong CG, Stevens MC | Biological psychiatry |
| 130 | [The executive control network and symptomatic improvement in attention-deficit/hyperactivity disorder.](https://neurosynth.org/studies/26363140/) | Francx W, Oldehinkel M, Oosterlaan J, Heslenfeld D, Hartman CA, Hoekstra PJ, Franke B, Beckmann CF, Buitelaar JK, Mennes M | Cortex; a journal devoted to the study of the nervous system and behavior |
| 131 | [The interaction between 5-HTTLPR and stress exposure influences connectivity of the executive control and default mode brain networks.](https://neurosynth.org/studies/27738993/) | van der Meer D, Hartman CA, Pruim RHR, Mennes M, Heslenfeld D, Oosterlaan J, Faraone SV, Franke B, Buitelaar JK, Hoekstra PJ | Brain imaging and behavior |
| 132 | [The link between callous-unemotional traits and neural mechanisms of reward processing: An fMRI study.](https://neurosynth.org/studies/27564545/) | Veroude K, von Rhein D, Chauvin RJ, van Dongen EV, Mennes MJ, Franke B, Heslenfeld DJ, Oosterlaan J, Hartman CA, Hoekstra PJ, Glennon JC, Buitelaar JK | Psychiatry research. Neuroimaging |
| 133 | [The neural basis of sustained and transient attentional control in young adults with ADHD.](https://neurosynth.org/studies/19619566/) | Banich MT, Burgess GC, Depue BE, Ruzic L, Bidwell LC, Hitt-Laustsen S, Du YP, Willcutt EG | Neuropsychologia |
| 134 | [The relationship between grey-matter and ASD and ADHD traits in typical adults.](https://neurosynth.org/studies/23138728/) | Geurts HM, Ridderinkhof KR, Scholte HS | Journal of autism and developmental disorders |
| 135 | [Topological organization of the "small-world" visual attention network in children with attention deficit/hyperactivity disorder (ADHD).](https://neurosynth.org/studies/24688465/) | Xia S, Foxe JJ, Sroubek AE, Branch C, Li X | Frontiers in human neuroscience |
| 136 | [Transcranial Direct Current Stimulation Modulates Neuronal Networks in Attention Deficit Hyperactivity Disorder.](https://neurosynth.org/studies/28213645/) | Sotnikova A, Soff C, Tagliazucchi E, Becker K, Siniatchkin M | Brain topography |
| 137 | [Treatment effect of methylphenidate on intrinsic functional brain network in medication-naive ADHD children: A multivariate analysis.](https://neurosynth.org/studies/28417219/) | Yoo JH, Kim D, Choi J, Jeong B | Brain imaging and behavior |
| 138 | [Ventral striatal hyperconnectivity during rewarded interference control in adolescents with ADHD.](https://neurosynth.org/studies/27399612/) | Ma I, van Holstein M, Mies GW, Mennes M, Buitelaar J, Cools R, Cillessen AHN, Krebs RM, Scheres A | Cortex; a journal devoted to the study of the nervous system and behavior |
| 139 | [White matter tract integrity of frontostriatal circuit in attention deficit hyperactivity disorder: association with attention performance and symptoms.](https://neurosynth.org/studies/22936578/) | Wu YH, Gau SS, Lo YC, Tseng WY | Human brain mapping |
| 140 | [White-matter abnormalities in attention deficit hyperactivity disorder: a diffusion tensor imaging study.](https://neurosynth.org/studies/19107752/) | Silk TJ, Vance A, Rinehart N, Bradshaw JL, Cunnington R | Human brain mapping |
| 141 | [Widespread Reductions in Cortical Thickness Following Severe Early-Life Deprivation: A Neurodevelopmental Pathway to Attention-Deficit/Hyperactivity Disorder.](https://neurosynth.org/studies/24090797/) | McLaughlin KA, Sheridan MA, Winter W, Fox NA, Zeanah CH, Nelson CA | Biological psychiatry |
| 142 | [Withholding and canceling a response in ADHD adolescents.](https://neurosynth.org/studies/25328838/) | Bhaijiwala M, Chevrier A, Schachar R | Brain and behavior |
| 143 | [Working memory in attention deficit/hyperactivity disorder is characterized by a lack of specialization of brain function.](https://neurosynth.org/studies/22102882/) | Fassbender C, Schweitzer JB, Cortes CR, Tagamets MA, Windsor TA, Reeves GM, Gullapalli R | PloS one |
| 144 | [Working memory-related functional brain patterns in never medicated children with ADHD.](https://neurosynth.org/studies/23166657/) | Massat I, Slama H, Kavec M, Linotte S, Mary A, Baleriaux D, Metens T, Mendlewicz J, Peigneux P | PloS one |

**Additional file 1: Table S2.** List of 95 studies extracted from Neurosynth under search term “anxiety disorders” September 21, 2020

| **No.** | **Year** | **Title** | **Authors** | **Journal** |
| --- | --- | --- | --- | --- |
| 1 | 2008 | [A functional magnetic resonance imaging investigation of uncertainty in adolescents with anxiety disorders.](https://neurosynth.org/studies/17719566/) | Krain AL, Gotimer K, Hefton S, Ernst M, Castellanos FX, Pine DS, Milham MP | Biological psychiatry |
| 2 | 2016 | [Aberrant Functional Connectivity between the Amygdala and the Temporal Pole in Drug-Free Generalized Anxiety Disorder.](https://neurosynth.org/studies/27867352/) | Li W, Cui H, Zhu Z, Kong L, Guo Q, Zhu Y, Hu Q, Zhang L, Li H, Li Q, Jiang J, Meyers J, Li J, Wang J, Yang Z, Li C | Frontiers in human neuroscience |
| 3 | 2017 | [Active Avoidance: Neural Mechanisms and Attenuation of Pavlovian Conditioned Responding.](https://neurosynth.org/studies/28408411/) | Boeke EA, Moscarello JM, LeDoux JE, Phelps EA, Hartley CA | The Journal of neuroscience : the official journal of the Society for Neuroscience |
| 4 | 2013 | [Allopregnanolone elevations following pregnenolone administration are associated with enhanced activation of emotion regulation neurocircuits.](https://neurosynth.org/studies/23348009/) | Sripada RK, Marx CE, King AP, Rampton JC, Ho SS, Liberzon I | Biological psychiatry |
| 5 | 2014 | [Alterations in amygdala functional connectivity reflect early temperament.](https://neurosynth.org/studies/25261727/) | Roy AK, Benson BE, Degnan KA, Perez-Edgar K, Pine DS, Fox NA, Ernst M | Biological psychology |
| 6 | 2018 | [Alterations of the amplitude of low-frequency fluctuations in anxiety in Parkinson's disease.](https://neurosynth.org/studies/29309855/) | Wang X, Li J, Wang M, Yuan Y, Zhu L, Shen Y, Zhang H, Zhang K | Neuroscience letters |
| 7 | 2016 | [Altered engagement of autobiographical memory networks in adult offspring of postnatally depressed mothers.](https://neurosynth.org/studies/27208693/) | Macdonald B, Murray L, Moutsiana C, Fearon P, Cooper PJ, Halligan SL, Johnstone T | Biological psychology |
| 8 | 2016 | [Altered striatal intrinsic functional connectivity in pediatric anxiety.](https://neurosynth.org/studies/27004799/) | Dorfman J, Benson B, Farber M, Pine D, Ernst M | Neuropsychologia |
| 9 | 2003 | [Amygdala and insular responses to emotionally valenced human faces in small animal specific phobia.](https://neurosynth.org/studies/14625149/) | Wright CI, Martis B, McMullin K, Shin LM, Rauch SL | Biological psychiatry |
| 10 | 2016 | [Amygdala-based intrinsic functional connectivity and anxiety disorders in adolescents and young adults.](https://neurosynth.org/studies/27716544/) | Toazza R, Franco AR, Buchweitz A, Molle RD, Rodrigues DM, Reis RS, Mucellini AB, Esper NB, Aguzzoli C, Silveira PP, Salum GA, Manfro GG | Psychiatry research. Neuroimaging |
| 11 | 2013 | [Anterior insular cortex mediates bodily sensibility and social anxiety.](https://neurosynth.org/studies/22977199/) | Terasawa Y, Shibata M, Moriguchi Y, Umeda S | Social cognitive and affective neuroscience |
| 12 | 2014 | [Anticipation of peer evaluation in anxious adolescents: divergence in neural activation and maturation.](https://neurosynth.org/studies/25552568/) | Spielberg JM, Jarcho JM, Dahl RE, Pine DS, Ernst M, Nelson EE | Social cognitive and affective neuroscience |
| 13 | 2007 | [Attention alters neural responses to evocative faces in behaviorally inhibited adolescents.](https://neurosynth.org/studies/17376704/) | Perez-Edgar K, Roberson-Nay R, Hardin MG, Poeth K, Guyer AE, Nelson EE, McClure EB, Henderson HA, Fox NA, Pine DS, Ernst M | NeuroImage |
| 14 | 2010 | [BDNF gene polymorphism (Val66Met) predicts amygdala and anterior hippocampus responses to emotional faces in anxious and depressed adolescents.](https://neurosynth.org/studies/19931400/) | Lau JY, Goldman D, Buzas B, Hodgkinson C, Leibenluft E, Nelson E, Sankin L, Pine DS, Ernst M | NeuroImage |
| 15 | 2003 | [Brain activation by disgust-inducing pictures in obsessive-compulsive disorder.](https://neurosynth.org/studies/14512216/) | Shapira NA, Liu Y, He AG, Bradley MM, Lessig MC, James GA, Stein DJ, Lang PJ, Goodman WK | Biological psychiatry |
| 16 | 2005 | [Brain regions associated with the expression and contextual regulation of anxiety in primates.](https://neurosynth.org/studies/16043132/) | Kalin NH, Shelton SE, Fox AS, Oakes TR, Davidson RJ | Biological psychiatry |
| 17 | 2012 | [Catechol-O-methyltransferase gene variation: impact on amygdala response to aversive stimuli.](https://neurosynth.org/studies/22387174/) | Domschke K, Baune BT, Havlik L, Stuhrmann A, Suslow T, Kugel H, Zwanzger P, Grotegerd D, Sehlmeyer C, Arolt V, Dannlowski U | NeuroImage |
| 18 | 2003 | [Change the mind and you change the brain: effects of cognitive-behavioral therapy on the neural correlates of spider phobia.](https://neurosynth.org/studies/12595193/) | Paquette V, Levesque J, Mensour B, Leroux JM, Beaudoin G, Bourgouin P, Beauregard M | NeuroImage |
| 19 | 2015 | [Controllability modulates the neural response to predictable but not unpredictable threat in humans.](https://neurosynth.org/studies/26149610/) | Wood KH, Wheelock MD, Shumen JR, Bowen KH, Ver Hoef LW, Knight DC | NeuroImage |
| 20 | 2017 | [Corticolimbic structural alterations linked to health status and trait anxiety in functional neurological disorder.](https://neurosynth.org/studies/28844071/) | Perez DL, Williams B, Matin N, LaFrance WC Jr, Costumero-Ramos V, Fricchione GL, Sepulcre J, Keshavan MS, Dickerson BC | Journal of neurology, neurosurgery, and psychiatry |
| 21 | 2015 | [Decreased amygdala-insula resting state connectivity in behaviorally and emotionally dysregulated youth.](https://neurosynth.org/studies/25433424/) | Bebko G, Bertocci M, Chase H, Dwojak A, Bonar L, Almeida J, Perlman SB, Versace A, Schirda C, Travis M, Gill MK, Demeter C, Diwadkar V, Sunshine J, Holland S, Kowatch R, Birmaher B, Axelson D, Horwitz S, Frazier T, Arnold LE, Fristad M, Youngstrom E, Findling R, Phillips ML | Psychiatry research |
| 22 | 2015 | [Developmental sex differences in resting state functional connectivity of amygdala sub-regions.](https://neurosynth.org/studies/25887261/) | Alarcon G, Cservenka A, Rudolph MD, Fair DA, Nagel BJ | NeuroImage |
| 23 | 2011 | [Different white matter abnormalities between the first-episode, treatment-naive patients with posttraumatic stress disorder and generalized anxiety disorder without comorbid conditions.](https://neurosynth.org/studies/21497403/) | Zhang L, Zhang Y, Li L, Li Z, Li W, Ma N, Hou C, Zhang Z, Zhang Z, Wang L, Duan L, Lu G | Journal of affective disorders |
| 24 | 2016 | [Differential Activation in Amygdala and Plasma Noradrenaline during Colorectal Distention by Administration of Corticotropin-Releasing Hormone between Healthy Individuals and Patients with Irritable B](https://neurosynth.org/studies/27448273/) | Tanaka Y, Kanazawa M, Kano M, Morishita J, Hamaguchi T, Van Oudenhove L, Ly HG, Dupont P, Tack J, Yamaguchi T, Yanai K, Tashiro M, Fukudo S | PloS one |
| 25 | 2013 | [Dissociable roles of ventromedial prefrontal cortex (vmPFC) and rostral anterior cingulate cortex (rACC) in value representation and optimistic bias.](https://neurosynth.org/studies/23567883/) | Blair KS, Otero M, Teng C, Jacobs M, Odenheimer S, Pine DS, Blair RJ | NeuroImage |
| 26 | 2006 | [Effects of cognitive-behavioral therapy on brain activation in specific phobia.](https://neurosynth.org/studies/16087353/) | Straube T, Glauer M, Dilger S, Mentzel HJ, Miltner WH | NeuroImage |
| 27 | 2013 | [Effects of Cognitive-Behavioral Therapy on Brain Responses to Subliminal and Supraliminal Threat and Their Functional Significance in Specific Phobia.](https://neurosynth.org/studies/24393393/) | Lipka J, Hoffmann M, Miltner WH, Straube T | Biological psychiatry |
| 28 | 2015 | [Effects of oxytocin and vasopressin on the neural response to unreciprocated cooperation within brain regions involved in stress and anxiety in men and women.](https://neurosynth.org/studies/26040978/) | Chen X, Hackett PD, DeMarco AC, Feng C, Stair S, Haroon E, Ditzen B, Pagnoni G, Rilling JK | Brain imaging and behavior |
| 29 | 2015 | [Effects of post-extinction l-DOPA administration on the spontaneous recovery and reinstatement of fear in a human fMRI study.](https://neurosynth.org/studies/26238968/) | Haaker J, Lonsdorf TB, Kalisch R | European neuropsychopharmacology : the journal of the European College of Neuropsychopharmacology |
| 30 | 2012 | [Effects of rapid eye movement sleep deprivation on fear extinction recall and prediction error signaling.](https://neurosynth.org/studies/21826762/) | Spoormaker VI, Schroter MS, Andrade KC, Dresler M, Kiem SA, Goya-Maldonado R, Wetter TC, Holsboer F, Samann PG, Czisch M | Human brain mapping |
| 31 | 2017 | [Effects of subclinical depression, anxiety and somatization on brain structure in healthy subjects.](https://neurosynth.org/studies/28319687/) | Besteher B, Gaser C, Langbein K, Dietzek M, Sauer H, Nenadic I | Journal of affective disorders |
| 32 | 2013 | [Enhanced amygdala reactivity to emotional faces in adults reporting childhood emotional maltreatment.](https://neurosynth.org/studies/22258799/) | van Harmelen AL, van Tol MJ, Demenescu LR, van der Wee NJ, Veltman DJ, Aleman A, van Buchem MA, Spinhoven P, Penninx BW, Elzinga BM | Social cognitive and affective neuroscience |
| 33 | 2016 | [Enhanced functional connectivity between sensorimotor and visual cortex predicts covariation bias in spider phobia.](https://neurosynth.org/studies/26805508/) | Wiemer J, Pauli P | Biological psychology |
| 34 | 2014 | [Equal pain-Unequal fear response: enhanced susceptibility of tooth pain to fear conditioning.](https://neurosynth.org/studies/25100974/) | Meier ML, de Matos NM, Brugger M, Ettlin DA, Lukic N, Cheetham M, Jancke L, Lutz K | Frontiers in human neuroscience |
| 35 | 2014 | [Evidence-based guidelines on the therapeutic use of repetitive transcranial magnetic stimulation (rTMS).](https://neurosynth.org/studies/25034472/) | Lefaucheur JP, Andre-Obadia N, Antal A, Ayache SS, Baeken C, Benninger DH, Cantello RM, Cincotta M, de Carvalho M, De Ridder D, Devanne H, Di Lazzaro V, Filipovic SR, Hummel FC, Jaaskelainen SK, Kimiskidis VK, Koch G, Langguth B, Nyffeler T, Oliviero A, Padberg F, Poulet E, Rossi S, Rossini PM, Rothwell JC, Schonfeldt-Lecuona C, Siebner HR, Slotema CW, Stagg CJ, Valls-Sole J, Ziemann U, Paulus W, Garcia-Larrea L | Clinical neurophysiology : official journal of the International Federation of Clinical Neurophysiology |
| 36 | 2011 | [Expectation and temperament moderate amygdala and dorsal anterior cingulate cortex responses to fear faces.](https://neurosynth.org/studies/21264642/) | Clauss JA, Cowan RL, Blackford JU | Cognitive, affective & behavioral neuroscience |
| 37 | 2004 | [Experience-dependent plasticity for attention to threat: Behavioral and neurophysiological evidence in humans.](https://neurosynth.org/studies/15476691/) | Monk CS, Nelson EE, Woldehawariat G, Montgomery LA, Zarahn E, McClure EB, Guyer AE, Leibenluft E, Charney DS, Ernst M, Pine DS | Biological psychiatry |
| 38 | 2011 | [Experiential, autonomic, and neural responses during threat anticipation vary as a function of threat intensity and neuroticism.](https://neurosynth.org/studies/21093595/) | Drabant EM, Kuo JR, Ramel W, Blechert J, Edge MD, Cooper JR, Goldin PR, Hariri AR, Gross JJ | NeuroImage |
| 39 | 2012 | [Exposure therapy triggers lasting reorganization of neural fear processing.](https://neurosynth.org/studies/22623532/) | Hauner KK, Mineka S, Voss JL, Paller KA | Proceedings of the National Academy of Sciences of the United States of America |
| 40 | 2017 | [Extinction of Conditioned Fear in Adolescents and Adults: A Human fMRI Study.](https://neurosynth.org/studies/29358913/) | Ganella DE, Drummond KD, Ganella EP, Whittle S, Kim JH | Frontiers in human neuroscience |
| 41 | 2016 | [First Steps in Using Multi-Voxel Pattern Analysis to Disentangle Neural Processes Underlying Generalization of Spider Fear.](https://neurosynth.org/studies/27303278/) | Visser RM, Haver P, Zwitser RJ, Scholte HS, Kindt M | Frontiers in human neuroscience |
| 42 | 2008 | [From fear to safety and back: reversal of fear in the human brain.](https://neurosynth.org/studies/18987188/) | Schiller D, Levy I, Niv Y, LeDoux JE, Phelps EA | The Journal of neuroscience : the official journal of the Society for Neuroscience |
| 43 | 2012 | [Functional magnetic resonance imaging correlates of emotional word encoding and recognition in depression and anxiety disorders.](https://neurosynth.org/studies/22206877/) | van Tol MJ, Demenescu LR, van der Wee NJ, Kortekaas R, Marjan M A N, Boer JA, Renken RJ, van Buchem MA, Zitman FG, Aleman A, Veltman DJ | Biological psychiatry |
| 44 | 2006 | [Functional neuroanatomy of aversion and its anticipation.](https://neurosynth.org/studies/16181793/) | Nitschke JB, Sarinopoulos I, Mackiewicz KL, Schaefer HS, Davidson RJ | NeuroImage |
| 45 | 2017 | [GABA Concentrations in the Anterior Cingulate Cortex Are Associated with Fear Network Function and Fear Recovery in Humans.](https://neurosynth.org/studies/28496404/) | Levar N, van Leeuwen JMC, Puts NAJ, Denys D, van Wingen GA | Frontiers in human neuroscience |
| 46 | 2009 | [Gender difference in relationship between anxiety-related personality traits and cerebral brain glucose metabolism.](https://neurosynth.org/studies/19682867/) | Hakamata Y, Iwase M, Iwata H, Kobayashi T, Tamaki T, Nishio M, Matsuda H, Ozaki N, Inada T | Psychiatry research |
| 47 | 2013 | [General emotion processing in social anxiety disorder: neural issues of cognitive control.](https://neurosynth.org/studies/23146247/) | Bruhl AB, Herwig U, Delsignore A, Jancke L, Rufer M | Psychiatry research |
| 48 | 2013 | [Gray matter abnormalities in social anxiety disorder: primary, replication, and specificity studies.](https://neurosynth.org/studies/22748614/) | Talati A, Pantazatos SP, Schneier FR, Weissman MM, Hirsch J | Biological psychiatry |
| 49 | 2009 | [High-frequency heart rate variability and cortico-striatal activity in men and women with social phobia.](https://neurosynth.org/studies/19505585/) | Ahs F, Sollers JJ 3rd, Furmark T, Fredrikson M, Thayer JF | NeuroImage |
| 50 | 2005 | [Hormonal cycle modulates arousal circuitry in women using functional magnetic resonance imaging.](https://neurosynth.org/studies/16207891/) | Goldstein JM, Jerram M, Poldrack R, Ahern T, Kennedy DN, Seidman LJ, Makris N | The Journal of neuroscience : the official journal of the Society for Neuroscience |
| 51 | 2011 | [How specific is specific phobia? Different neural response patterns in two subtypes of specific phobia.](https://neurosynth.org/studies/21316468/) | Lueken U, Kruschwitz JD, Muehlhan M, Siegert J, Hoyer J, Wittchen HU | NeuroImage |
| 52 | 2010 | [Human bed nucleus of the stria terminalis indexes hypervigilant threat monitoring.](https://neurosynth.org/studies/20497902/) | Somerville LH, Whalen PJ, Kelley WM | Biological psychiatry |
| 53 | 2010 | [Human dopamine receptor D2/D3 availability predicts amygdala reactivity to unpleasant stimuli.](https://neurosynth.org/studies/19904802/) | Kobiella A, Vollstadt-Klein S, Buhler M, Graf C, Buchholz HG, Bernow N, Yakushev IY, Landvogt C, Schreckenberger M, Grunder G, Bartenstein P, Fehr C, Smolka MN | Human brain mapping |
| 54 | 2009 | [Human fear conditioning and extinction in neuroimaging: a systematic review.](https://neurosynth.org/studies/19517024/) | Sehlmeyer C, Schoning S, Zwitserlood P, Pfleiderer B, Kircher T, Arolt V, Konrad C | PloS one |
| 55 | 2014 | [Hypoactive medial prefrontal cortex functioning in adults reporting childhood emotional maltreatment.](https://neurosynth.org/studies/24493840/) | van Harmelen AL, van Tol MJ, Dalgleish T, van der Wee NJ, Veltman DJ, Aleman A, Spinhoven P, Penninx BW, Elzinga BM | Social cognitive and affective neuroscience |
| 56 | 2011 | [Impact of mindfulness on the neural responses to emotional pictures in experienced and beginner meditators.](https://neurosynth.org/studies/21679770/) | Taylor VA, Grant J, Daneault V, Scavone G, Breton E, Roffe-Vidal S, Courtemanche J, Lavarenne AS, Beauregard M | NeuroImage |
| 57 | 2015 | [Increased activity of frontal and limbic regions to emotional stimuli in children at-risk for anxiety disorders.](https://neurosynth.org/studies/25978933/) | Christensen R, Van Ameringen M, Hall G | Psychiatry research |
| 58 | 2012 | [Increased neural habituation in the amygdala and orbitofrontal cortex in social anxiety disorder revealed by FMRI.](https://neurosynth.org/studies/23209643/) | Sladky R, Hoflich A, Atanelov J, Kraus C, Baldinger P, Moser E, Lanzenberger R, Windischberger C | PloS one |
| 59 | 2008 | [Intolerance of uncertainty correlates with insula activation during affective ambiguity.](https://neurosynth.org/studies/18079060/) | Simmons A, Matthews SC, Paulus MP, Stein MB | Neuroscience letters |
| 60 | 2013 | [Learning to like disgust: neuronal correlates of counterconditioning.](https://neurosynth.org/studies/23847514/) | Schweckendiek J, Klucken T, Merz CJ, Kagerer S, Walter B, Vaitl D, Stark R | Frontiers in human neuroscience |
| 61 | 2014 | [Left and right amygdala - mediofrontal cortical functional connectivity is differentially modulated by harm avoidance.](https://neurosynth.org/studies/24760033/) | Baeken C, Marinazzo D, Van Schuerbeek P, Wu GR, De Mey J, Luypaert R, De Raedt R | PloS one |
| 62 | 2013 | [MBSR vs aerobic exercise in social anxiety: fMRI of emotion regulation of negative self-beliefs.](https://neurosynth.org/studies/22586252/) | Goldin P, Ziv M, Jazaieri H, Hahn K, Gross JJ | Social cognitive and affective neuroscience |
| 63 | 2015 | [Modulation of prefrontal functioning in attention systems by NPSR1 gene variation.](https://neurosynth.org/studies/25842293/) | Neufang S, Geiger MJ, Homola GA, Mahr M, Akhrif A, Nowak J, Reif A, Romanos M, Deckert J, Solymosi L, Domschke K | NeuroImage |
| 64 | 2013 | [Neural correlates of a computerized attention modification program in anxious subjects.](https://neurosynth.org/studies/23934417/) | Taylor CT, Aupperle RL, Flagan T, Simmons AN, Amir N, Stein MB, Paulus MP | Social cognitive and affective neuroscience |
| 65 | 2010 | [Neural correlates of aversive conditioning: development of a functional imaging paradigm for the investigation of anxiety disorders.](https://neurosynth.org/studies/20148332/) | Reinhardt I, Jansen A, Kellermann T, Schuppen A, Kohn N, Gerlach AL, Kircher T | European archives of psychiatry and clinical neuroscience |
| 66 | 2011 | [Neural correlates of personality dimensions and affective measures during the anticipation of emotional stimuli.](https://neurosynth.org/studies/21264550/) | Bruhl AB, Viebke MC, Baumgartner T, Kaffenberger T, Herwig U | Brain imaging and behavior |
| 67 | 2015 | [Neural correlates of valence generalization in an affective conditioning paradigm.](https://neurosynth.org/studies/26057359/) | Schick A, Adam R, Vollmayr B, Kuehner C, Kanske P, Wessa M | Behavioural brain research |
| 68 | 2007 | [Neural responses to auditory stimulus deviance under threat of electric shock revealed by spatially-filtered magnetoencephalography.](https://neurosynth.org/studies/17566766/) | Cornwell BR, Baas JM, Johnson L, Holroyd T, Carver FW, Lissek S, Grillon C | NeuroImage |
| 69 | 2013 | [Neural substrates of classically conditioned fear-generalization in humans: a parametric fMRI study.](https://neurosynth.org/studies/23748500/) | Lissek S, Bradford DE, Alvarez RP, Burton P, Espensen-Sturges T, Reynolds RC, Grillon C | Social cognitive and affective neuroscience |
| 70 | 2011 | [Neurobehavioral mechanisms of human fear generalization.](https://neurosynth.org/studies/21256233/) | Dunsmoor JE, Prince SE, Murty VP, Kragel PA, LaBar KS | NeuroImage |
| 71 | 2014 | [Neurodevelopmental changes in the responsiveness of systems involved in top down attention and emotional responding.](https://neurosynth.org/studies/25128588/) | Hwang S, White SF, Nolan ZT, Sinclair S, Blair RJ | Neuropsychologia |
| 72 | 2016 | [No evidence for blocking the return of fear by disrupting reconsolidation prior to extinction learning.](https://neurosynth.org/studies/27111105/) | Klucken T, Kruse O, Schweckendiek J, Kuepper Y, Mueller EM, Hennig J, Stark R | Cortex; a journal devoted to the study of the nervous system and behavior |
| 73 | 2014 | [No evidence for enhanced extinction memory consolidation through noradrenergic reuptake inhibition-delayed memory test and reinstatement in human fMRI.](https://neurosynth.org/studies/24193372/) | Lonsdorf TB, Haaker J, Fadai T, Kalisch R | Psychopharmacology |
| 74 | 2009 | [Noradrenergic enhancement of amygdala responses to fear.](https://neurosynth.org/studies/19246474/) | Onur OA, Walter H, Schlaepfer TE, Rehme AK, Schmidt C, Keysers C, Maier W, Hurlemann R | Social cognitive and affective neuroscience |
| 75 | 2014 | [Of 'disgrace' and 'pain' - corticolimbic interaction patterns for disorder-relevant and emotional words in social phobia.](https://neurosynth.org/studies/25396729/) | Laeger I, Dobel C, Radenz B, Kugel H, Keuper K, Eden A, Arolt V, Zwitserlood P, Dannlowski U, Zwanzger P | PloS one |
| 76 | 2014 | [Oxytocin Facilitates the Extinction of Conditioned Fear in Humans.](https://neurosynth.org/studies/25542304/) | Eckstein M, Becker B, Scheele D, Scholz C, Preckel K, Schlaepfer TE, Grinevich V, Kendrick KM, Maier W, Hurlemann R | Biological psychiatry |
| 77 | 2009 | [Preclinical atherosclerosis covaries with individual differences in reactivity and functional connectivity of the amygdala.](https://neurosynth.org/studies/19013557/) | Gianaros PJ, Hariri AR, Sheu LK, Muldoon MF, Sutton-Tyrrell K, Manuck SB | Biological psychiatry |
| 78 | 2017 | [Prefrontal and amygdala engagement during emotional reactivity and regulation in generalized anxiety disorder.](https://neurosynth.org/studies/28501740/) | Fitzgerald JM, Phan KL, Kennedy AE, Shankman SA, Langenecker SA, Klumpp H | Journal of affective disorders |
| 79 | 2017 | [Prefrontal-Amygdala Connectivity and State Anxiety during Fear Extinction Recall in Adolescents.](https://neurosynth.org/studies/29255411/) | Ganella DE, Barendse MEA, Kim JH, Whittle S | Frontiers in human neuroscience |
| 80 | 2012 | [Pregabalin effects on neural response to emotional faces.](https://neurosynth.org/studies/22470326/) | Aupperle RL, Tankersley D, Ravindran LN, Flagan T, Stein NR, Stein MB, Paulus MP | Frontiers in human neuroscience |
| 81 | 2015 | [Preschool anxiety disorders predict different patterns of amygdala-prefrontal connectivity at school-age.](https://neurosynth.org/studies/25625285/) | Carpenter KL, Angold A, Chen NK, Copeland WE, Gaur P, Pelphrey K, Song AW, Egger HL | PloS one |
| 82 | 2015 | [PTSD symptom severity is associated with increased recruitment of top-down attentional control in a trauma-exposed sample.](https://neurosynth.org/studies/25610763/) | White SF, Costanzo ME, Blair JR, Roy MJ | NeuroImage. Clinical |
| 83 | 2010 | [Reduced medial prefrontal cortex volume in adults reporting childhood emotional maltreatment.](https://neurosynth.org/studies/20692648/) | van Harmelen AL, van Tol MJ, van der Wee NJ, Veltman DJ, Aleman A, Spinhoven P, van Buchem MA, Zitman FG, Penninx BW, Elzinga BM | Biological psychiatry |
| 84 | 2011 | [Reduced resting-state functional connectivity between amygdala and orbitofrontal cortex in social anxiety disorder.](https://neurosynth.org/studies/21356318/) | Hahn A, Stein P, Windischberger C, Weissenbacher A, Spindelegger C, Moser E, Kasper S, Lanzenberger R | NeuroImage |
| 85 | 2017 | [Sex-dependent neural effect of oxytocin during subliminal processing of negative emotion faces.](https://neurosynth.org/studies/28877512/) | Luo L, Becker B, Geng Y, Zhao Z, Gao S, Zhao W, Yao S, Zheng X, Ma X, Gao Z, Hu J, Kendrick KM | NeuroImage |
| 86 | 2010 | [The impact of anxiety-inducing distraction on cognitive performance: a combined brain imaging and personality investigation.](https://neurosynth.org/studies/21152391/) | Denkova E, Wong G, Dolcos S, Sung K, Wang L, Coupland N, Dolcos F | PloS one |
| 87 | 2015 | [The many faces of anxiety-neurobiological correlates of anxiety phenotypes.](https://neurosynth.org/studies/26347412/) | Andreescu C, Mennin D, Tudorascu D, Sheu LK, Walker S, Banihashemi L, Aizenstein H | Psychiatry research |
| 88 | 2015 | [The neuroanatomical basis of panic disorder and social phobia in schizophrenia: a voxel based morphometric study.](https://neurosynth.org/studies/25774979/) | Picado M, Carmona S, Hoekzema E, Pailhez G, Berge D, Mane A, Fauquet J, Hilferty J, Moreno A, Cortizo R, Vilarroya O, Bulbena A | PloS one |
| 89 | 2013 | [The role of serotonin in the neurocircuitry of negative affective bias: serotonergic modulation of the dorsal medial prefrontal-amygdala 'aversive amplification' circuit.](https://neurosynth.org/studies/23583742/) | Robinson OJ, Overstreet C, Allen PS, Letkiewicz A, Vytal K, Pine DS, Grillon C | NeuroImage |
| 90 | 2013 | [Tired and apprehensive: anxiety amplifies the impact of sleep loss on aversive brain anticipation.](https://neurosynth.org/studies/23804084/) | Goldstein AN, Greer SM, Saletin JM, Harvey AG, Nitschke JB, Walker MP | The Journal of neuroscience : the official journal of the Society for Neuroscience |
| 91 | 2011 | [Uncovering putative neural markers of risk avoidance.](https://neurosynth.org/studies/21354189/) | Roy AK, Gotimer K, Kelly AM, Castellanos FX, Milham MP, Ernst M | Neuropsychologia |
| 92 | 2017 | [Unreliability of putative fMRI biomarkers during emotional face processing.](https://neurosynth.org/studies/28506872/) | Nord CL, Gray A, Charpentier CJ, Robinson OJ, Roiser JP | NeuroImage |
| 93 | 2010 | [Vasopressin modulates medial prefrontal cortex-amygdala circuitry during emotion processing in humans.](https://neurosynth.org/studies/20484643/) | Zink CF, Stein JL, Kempf L, Hakimi S, Meyer-Lindenberg A | The Journal of neuroscience : the official journal of the Society for Neuroscience |
| 94 | 2013 | [Ventromedial prefrontal cortex and the regulation of physiological arousal.](https://neurosynth.org/studies/23620600/) | Zhang S, Hu S, Chao HH, Ide JS, Luo X, Farr OM, Li CS | Social cognitive and affective neuroscience |
| 95 | 2014 | [Ventromedial Prefrontal Cortex Is Critical for the Regulation of Amygdala Activity in Humans.](https://neurosynth.org/studies/24673881/) | Motzkin JC, Philippi CL, Wolf RC, Baskaya MK, Koenigs M | Biological psychiatry |

**Additional file 1: Table S3.** List of 170 studies extracted from Neurosynth under search term “autism spectrum” on September 21, 2020

| **No.** | **Title** | **Authors** | **Journal** |
| --- | --- | --- | --- |
| 1 | [A comparison of neural correlates underlying social cognition in Klinefelter syndrome and autism.](https://neurosynth.org/studies/24396006/) | Brandenburg-Goddard MN, van Rijn S, Rombouts SA, Veer IM, Swaab H | Social cognitive and affective neuroscience |
| 2 | [A greater involvement of posterior brain areas in interhemispheric transfer in autism: fMRI, DWI and behavioral evidences.](https://neurosynth.org/studies/26106551/) | Barbeau EB, Lewis JD, Doyon J, Benali H, Zeffiro TA, Mottron L | NeuroImage. Clinical |
| 3 | [Aberrant functional connectivity in autism: evidence from low-frequency BOLD signal fluctuations.](https://neurosynth.org/studies/19401185/) | Noonan SK, Haist F, Muller RA | Brain research |
| 4 | [Aberrant neural mediation of verbal fluency in autism spectrum disorders.](https://neurosynth.org/studies/24056237/) | Kenworthy L, Wallace GL, Birn R, Milleville SC, Case LK, Bandettini PA, Martin A | Brain and cognition |
| 5 | [Aberrant striatal functional connectivity in children with autism.](https://neurosynth.org/studies/21195388/) | Di Martino A, Kelly C, Grzadzinski R, Zuo XN, Mennes M, Mairena MA, Lord C, Castellanos FX, Milham MP | Biological psychiatry |
| 6 | [Abnormal autonomic and associated brain activities during rest in autism spectrum disorder.](https://neurosynth.org/studies/24424916/) | Eilam-Stock T, Xu P, Cao M, Gu X, Van Dam NT, Anagnostou E, Kolevzon A, Soorya L, Park Y, Siller M, He Y, Hof PR, Fan J | Brain : a journal of neurology |
| 7 | [Abnormal functional connectivity of default mode sub-networks in autism spectrum disorder patients.](https://neurosynth.org/studies/20621638/) | Assaf M, Jagannathan K, Calhoun VD, Miller L, Stevens MC, Sahl R, O'Boyle JG, Schultz RT, Pearlson GD | NeuroImage |
| 8 | [Abnormal Neural Activation to Faces in the Parents of Children with Autism.](https://neurosynth.org/studies/25056573/) | Yucel GH, Belger A, Bizzell J, Parlier M, Adolphs R, Piven J | Cerebral cortex (New York, N.Y. : 1991) |
| 9 | [Abnormalities of Inter- and Intra-Hemispheric Functional Connectivity in Autism Spectrum Disorders: A Study Using the Autism Brain Imaging Data Exchange Database.](https://neurosynth.org/studies/27199653/) | Lee JM, Kyeong S, Kim E, Cheon KA | Frontiers in neuroscience |
| 10 | [Abnormalities of intrinsic functional connectivity in autism spectrum disorders.](https://neurosynth.org/studies/19409498/) | Monk CS, Peltier SJ, Wiggins JL, Weng SJ, Carrasco M, Risi S, Lord C | NeuroImage |
| 11 | [Action simulation and mirroring in children with autism spectrum disorders.](https://neurosynth.org/studies/29247748/) | Wadsworth HM, Maximo JO, Donnelly RJ, Kana RK | Behavioural brain research |
| 12 | [Age related changes in striatal resting state functional connectivity in autism.](https://neurosynth.org/studies/24348363/) | Padmanabhan A, Lynn A, Foran W, Luna B, O'Hearn K | Frontiers in human neuroscience |
| 13 | [Age-dependent changes in the neural substrates of empathy in autism spectrum disorder.](https://neurosynth.org/studies/23784073/) | Schulte-Ruther M, Greimel E, Piefke M, Kamp-Becker I, Remschmidt H, Fink GR, Herpertz-Dahlmann B, Konrad K | Social cognitive and affective neuroscience |
| 14 | [Age-related abnormalities in white matter microstructure in autism spectrum disorders.](https://neurosynth.org/studies/22902768/) | Kleinhans NM, Pauley G, Richards T, Neuhaus E, Martin N, Corrigan NM, Shaw DW, Estes A, Dager SR | Brain research |
| 15 | [Alterations in regional homogeneity of resting-state brain activity in autism spectrum disorders.](https://neurosynth.org/studies/20053346/) | Paakki JJ, Rahko J, Long X, Moilanen I, Tervonen O, Nikkinen J, Starck T, Remes J, Hurtig T, Haapsamo H, Jussila K, Kuusikko-Gauffin S, Mattila ML, Zang Y, Kiviniemi V | Brain research |
| 16 | [Alterations of resting state functional connectivity in the default network in adolescents with autism spectrum disorders.](https://neurosynth.org/studies/20004180/) | Weng SJ, Wiggins JL, Peltier SJ, Carrasco M, Risi S, Lord C, Monk CS | Brain research |
| 17 | [Altered integration of speech and gesture in children with autism spectrum disorders.](https://neurosynth.org/studies/23139906/) | Hubbard AL, McNealy K, Scott-Van Zeeland AA, Callan DE, Bookheimer SY, Dapretto M | Brain and behavior |
| 18 | [Altered network topologies and hub organization in adults with autism: a resting-state FMRI study.](https://neurosynth.org/studies/24714805/) | Itahashi T, Yamada T, Watanabe H, Nakamura M, Jimbo D, Shioda S, Toriizuka K, Kato N, Hashimoto R | PloS one |
| 19 | [Altered resting perfusion and functional connectivity of default mode network in youth with autism spectrum disorder.](https://neurosynth.org/studies/26445698/) | Jann K, Hernandez LM, Beck-Pancer D, McCarron R, Smith RX, Dapretto M, Wang DJ | Brain and behavior |
| 20 | [Amygdala dysfunction in men with the fragile X premutation.](https://neurosynth.org/studies/17166860/) | Hessl D, Rivera S, Koldewyn K, Cordeiro L, Adams J, Tassone F, Hagerman PJ, Hagerman RJ | Brain : a journal of neurology |
| 21 | [Amygdala engagement in response to subthreshold presentations of anxious face stimuli in adults with autism spectrum disorders: preliminary insights.](https://neurosynth.org/studies/20520836/) | Hall GB, Doyle KA, Goldberg J, West D, Szatmari P | PloS one |
| 22 | [An fMRI study of reduced perceptual load-dependent modulation of task-irrelevant activity in adults with autism spectrum conditions.](https://neurosynth.org/studies/22465842/) | Ohta H, Yamada T, Watanabe H, Kanai C, Tanaka E, Ohno T, Takayama Y, Iwanami A, Kato N, Hashimoto R | NeuroImage |
| 23 | [Approaches to local connectivity in autism using resting state functional connectivity MRI.](https://neurosynth.org/studies/24155702/) | Maximo JO, Keown CL, Nair A, Muller RA | Frontiers in human neuroscience |
| 24 | [Association between amygdala response to emotional faces and social anxiety in autism spectrum disorders.](https://neurosynth.org/studies/20655320/) | Kleinhans NM, Richards T, Weaver K, Johnson LC, Greenson J, Dawson G, Aylward E | Neuropsychologia |
| 25 | [Attenuation of the contingency detection effect in the extrastriate body area in autism spectrum disorder.](https://neurosynth.org/studies/25066523/) | Okamoto Y, Kitada R, Tanabe HC, Hayashi MJ, Kochiyama T, Munesue T, Ishitobi M, Saito DN, Yanaka HT, Omori M, Wada Y, Okazawa H, Sasaki AT, Morita T, Itakura S, Kosaka H, Sadato N | Neuroscience research |
| 26 | [Attribution of emotions to body postures: An independent component analysis study of functional connectivity in autism.](https://neurosynth.org/studies/24838987/) | Libero LE, Stevens CE Jr, Kana RK | Human brain mapping |
| 27 | [Atypical [corrected] participation of visual cortex during word processing in autism: an fMRI study of semantic decision.](https://neurosynth.org/studies/17336346/) | Gaffrey MS, Kleinhans NM, Haist F, Akshoomoff N, Campbell A, Courchesne E, Muller RA | Neuropsychologia |
| 28 | [Atypical activation of action-semantic network in adolescents with autism spectrum disorder.](https://neurosynth.org/studies/28629645/) | Knaus TA, Burns C, Kamps J, Foundas AL | Brain and cognition |
| 29 | [Atypical activation of the mirror neuron system during perception of hand motion in autism.](https://neurosynth.org/studies/20096269/) | Martineau J, Andersson F, Barthelemy C, Cottier JP, Destrieux C | Brain research |
| 30 | [Atypical brain activation patterns during a face-to-face joint attention game in adults with autism spectrum disorder.](https://neurosynth.org/studies/22505330/) | Redcay E, Dodell-Feder D, Mavros PL, Kleiner M, Pearrow MJ, Triantafyllou C, Gabrieli JD, Saxe R | Human brain mapping |
| 31 | [Atypical functional lateralization of language in autism spectrum disorders.](https://neurosynth.org/studies/18555209/) | Kleinhans NM, Muller RA, Cohen DN, Courchesne E | Brain research |
| 32 | [Atypical lexicosemantic function of extrastriate cortex in autism spectrum disorder: evidence from functional and effective connectivity.](https://neurosynth.org/studies/22699044/) | Shen MD, Shih P, Ottl B, Keehn B, Leyden KM, Gaffrey MS, Muller RA | NeuroImage |
| 33 | [Atypical modulation of cognitive control by arousal in autism.](https://neurosynth.org/studies/18954965/) | Dichter GS, Belger A | Psychiatry research |
| 34 | [Atypical modulation of distant functional connectivity by cognitive state in children with Autism Spectrum Disorders.](https://neurosynth.org/studies/23986678/) | You X, Norr M, Murphy E, Kuschner ES, Bal E, Gaillard WD, Kenworthy L, Vaidya CJ | Frontiers in human neuroscience |
| 35 | [Atypical neural networks for social orienting in autism spectrum disorders.](https://neurosynth.org/studies/21334443/) | Greene DJ, Colich N, Iacoboni M, Zaidel E, Bookheimer SY, Dapretto M | NeuroImage |
| 36 | [Atypical neural self-representation in autism.](https://neurosynth.org/studies/20008375/) | Lombardo MV, Chakrabarti B, Bullmore ET, Sadek SA, Pasco G, Wheelwright SJ, Suckling J, Baron-Cohen S | Brain : a journal of neurology |
| 37 | [Atypical neural substrates of Embedded Figures Task performance in children with Autism Spectrum Disorder.](https://neurosynth.org/studies/17707658/) | Lee PS, Foss-Feig J, Henderson JG, Kenworthy LE, Gilotty L, Gaillard WD, Vaidya CJ | NeuroImage |
| 38 | [Atypical perception of affective prosody in Autism Spectrum Disorder.](https://neurosynth.org/studies/25379450/) | Gebauer L, Skewes J, Horlyck L, Vuust P | NeuroImage. Clinical |
| 39 | [Atypical recruitment of medial prefrontal cortex in autism spectrum disorders: an fMRI study of two executive function tasks.](https://neurosynth.org/studies/18485420/) | Gilbert SJ, Bird G, Brindley R, Frith CD, Burgess PW | Neuropsychologia |
| 40 | [Autism is characterized by dorsal anterior cingulate hyperactivation during social target detection.](https://neurosynth.org/studies/19574440/) | Dichter GS, Felder JN, Bodfish JW | Social cognitive and affective neuroscience |
| 41 | [Autism Spectrum Disorder Related Functional Connectivity Changes in the Language Network in Children, Adolescents and Adults.](https://neurosynth.org/studies/28867997/) | Lee Y, Park BY, James O, Kim SG, Park H | Frontiers in human neuroscience |
| 42 | [Autism spectrum traits in the typical population predict structure and function in the posterior superior temporal sulcus.](https://neurosynth.org/studies/20439317/) | von dem Hagen EA, Nummenmaa L, Yu R, Engell AD, Ewbank MP, Calder AJ | Cerebral cortex (New York, N.Y. : 1991) |
| 43 | [Autism spectrum traits predict the neural response to eye gaze in typical individuals.](https://neurosynth.org/studies/22062191/) | Nummenmaa L, Engell AD, von dem Hagen E, Henson RN, Calder AJ | NeuroImage |
| 44 | [Autonomic and brain responses associated with empathy deficits in autism spectrum disorder.](https://neurosynth.org/studies/25995134/) | Gu X, Eilam-Stock T, Zhou T, Anagnostou E, Kolevzon A, Soorya L, Hof PR, Friston KJ, Fan J | Human brain mapping |
| 45 | [Body expressions of emotion do not trigger fear contagion in autism spectrum disorder.](https://neurosynth.org/studies/19151375/) | Hadjikhani N, Joseph RM, Manoach DS, Naik P, Snyder J, Dominick K, Hoge R, Van den Stock J, Tager Flusberg H, de Gelder B | Social cognitive and affective neuroscience |
| 46 | [Brain activation during semantic processing in autism spectrum disorders via functional magnetic resonance imaging.](https://neurosynth.org/studies/16473449/) | Harris GJ, Chabris CF, Clark J, Urban T, Aharon I, Steele S, McGrath L, Condouris K, Tager-Flusberg H | Brain and cognition |
| 47 | [Brain activity of adolescents with high functioning autism in response to emotional words and facial emoticons.](https://neurosynth.org/studies/24621866/) | Han DH, Yoo HJ, Kim BN, McMahon W, Renshaw PF | PloS one |
| 48 | [Brain Mechanisms for Processing Affective (and Nonaffective) Touch Are Atypical in Autism.](https://neurosynth.org/studies/26048952/) | Kaiser MD, Yang DY, Voos AC, Bennett RH, Gordon I, Pretzsch C, Beam D, Keifer C, Eilbott J, McGlone F, Pelphrey KA | Cerebral cortex (New York, N.Y. : 1991) |
| 49 | [Brain organization underlying superior mathematical abilities in children with autism.](https://neurosynth.org/studies/23954299/) | Iuculano T, Rosenberg-Lee M, Supekar K, Lynch CJ, Khouzam A, Phillips J, Uddin LQ, Menon V | Biological psychiatry |
| 50 | [Changes in intrinsic connectivity of the brain's reading network following intervention in children with autism.](https://neurosynth.org/studies/26058572/) | Murdaugh DL, Maximo JO, Kana RK | Human brain mapping |
| 51 | [Changes in intrinsic local connectivity after reading intervention in children with autism.](https://neurosynth.org/studies/28869842/) | Maximo JO, Murdaugh DL, O'Kelley S, Kana RK | Brain and language |
| 52 | [Coherent motion processing in autism spectrum disorder (ASD): an fMRI study.](https://neurosynth.org/studies/20153764/) | Brieber S, Herpertz-Dahlmann B, Fink GR, Kamp-Becker I, Remschmidt H, Konrad K | Neuropsychologia |
| 53 | [Common and distinct neural features of social and non-social reward processing in autism and social anxiety disorder.](https://neurosynth.org/studies/23223206/) | Richey JA, Rittenberg A, Hughes L, Damiano CR, Sabatino A, Miller S, Hanna E, Bodfish JW, Dichter GS | Social cognitive and affective neuroscience |
| 54 | [Comparative Multimodal Meta-analysis of Structural and Functional Brain Abnormalities in Autism Spectrum Disorder and Obsessive-Compulsive Disorder.](https://neurosynth.org/studies/27887721/) | Carlisi CO, Norman LJ, Lukito SS, Radua J, Mataix-Cols D, Rubia K | Biological psychiatry |
| 55 | [Connectivity-based parcellation increases network detection sensitivity in resting state fMRI: An investigation into the cingulate cortex in autism.](https://neurosynth.org/studies/27114898/) | Balsters JH, Mantini D, Apps MA, Eickhoff SB, Wenderoth N | NeuroImage. Clinical |
| 56 | [Convergent Findings of Altered Functional and Structural Brain Connectivity in Individuals with High Functioning Autism: A Multimodal MRI Study.](https://neurosynth.org/studies/23825652/) | Mueller S, Keeser D, Samson AC, Kirsch V, Blautzik J, Grothe M, Erat O, Hegenloh M, Coates U, Reiser MF, Hennig-Fast K, Meindl T | PloS one |
| 57 | [Cortical responses to dynamic emotional facial expressions generalize across stimuli, and are sensitive to task-relevance, in adults with and without Autism.](https://neurosynth.org/studies/29554540/) | Kliemann D, Richardson H, Anzellotti S, Ayyash D, Haskins AJ, Gabrieli JDE, Saxe RR | Cortex; a journal devoted to the study of the nervous system and behavior |
| 58 | [Decoding versus comprehension: Brain responses underlying reading comprehension in children with autism.](https://neurosynth.org/studies/28242518/) | Bednarz HM, Maximo JO, Murdaugh DL, O'Kelley S, Kana RK | Brain and language |
| 59 | [Default mode network segregation and social deficits in autism spectrum disorder: Evidence from non-medicated children.](https://neurosynth.org/studies/26484047/) | Yerys BE, Gordon EM, Abrams DN, Satterthwaite TD, Weinblatt R, Jankowski KF, Strang J, Kenworthy L, Gaillard WD, Vaidya CJ | NeuroImage. Clinical |
| 60 | [Describing the brain in autism in five dimensions--magnetic resonance imaging-assisted diagnosis of autism spectrum disorder using a multiparameter classification approach.](https://neurosynth.org/studies/20702694/) | Ecker C, Marquand A, Mourao-Miranda J, Johnston P, Daly EM, Brammer MJ, Maltezos S, Murphy CM, Robertson D, Williams SC, Murphy DG | The Journal of neuroscience : the official journal of the Society for Neuroscience |
| 61 | [Deviant functional magnetic resonance imaging patterns of brain activity to speech in 2-3-year-old children with autism spectrum disorder.](https://neurosynth.org/studies/18672231/) | Redcay E, Courchesne E | Biological psychiatry |
| 62 | [Differences in global and local level information processing in autism: an fMRI investigation.](https://neurosynth.org/studies/23768913/) | Gadgil M, Peterson E, Tregellas J, Hepburn S, Rojas DC | Psychiatry research |
| 63 | [Differences in neural activity when processing emotional arousal and valence in autism spectrum disorders.](https://neurosynth.org/studies/26526072/) | Tseng A, Wang Z, Huo Y, Goh S, Russell JA, Peterson BS | Human brain mapping |
| 64 | [Differential Fairness Decisions and Brain Responses After Expressed Emotions of Others in Boys with Autism Spectrum Disorders.](https://neurosynth.org/studies/28516421/) | Klapwijk ET, Aghajani M, Lelieveld GJ, van Lang NDJ, Popma A, van der Wee NJA, Colins OF, Vermeiren RRJM | Journal of autism and developmental disorders |
| 65 | [Differential role of temporoparietal junction and medial prefrontal cortex in causal inference in autism: An independent component analysis.](https://neurosynth.org/studies/24695086/) | Murdaugh DL, Nadendla KD, Kana RK | Neuroscience letters |
| 66 | [Differentiating neural reward responsiveness in autism versus ADHD.](https://neurosynth.org/studies/25190643/) | Kohls G, Thonessen H, Bartley GK, Grossheinrich N, Fink GR, Herpertz-Dahlmann B, Konrad K | Developmental cognitive neuroscience |
| 67 | [Diminished medial prefrontal activity behind autistic social judgments of incongruent information.](https://neurosynth.org/studies/22745788/) | Watanabe T, Yahata N, Abe O, Kuwabara H, Inoue H, Takano Y, Iwashiro N, Natsubori T, Aoki Y, Takao H, Sasaki H, Gonoi W, Murakami M, Katsura M, Kunimatsu A, Kawakubo Y, Matsuzaki H, Tsuchiya KJ, Kato N, Kano Y, Miyashita Y, Kasai K, Yamasue H | PloS one |
| 68 | [Diminished neural adaptation during implicit learning in autism.](https://neurosynth.org/studies/26484826/) | Schipul SE, Just MA | NeuroImage |
| 69 | [Direct Gaze Elicits Atypical Activation of the Theory-of-Mind Network in Autism Spectrum Conditions.](https://neurosynth.org/studies/23324559/) | von dem Hagen EA, Stoyanova RS, Rowe JB, Baron-Cohen S, Calder AJ | Cerebral cortex (New York, N.Y. : 1991) |
| 70 | [Discrete neural substrates underlie complementary audiovisual speech integration processes.](https://neurosynth.org/studies/21195198/) | Stevenson RA, VanDerKlok RM, Pisoni DB, James TW | NeuroImage |
| 71 | [Disorder-specific predictive classification of adolescents with attention deficit hyperactivity disorder (ADHD) relative to autism using structural magnetic resonance imaging.](https://neurosynth.org/studies/23696841/) | Lim L, Marquand A, Cubillo AA, Smith AB, Chantiluke K, Simmons A, Mehta M, Rubia K | PloS one |
| 72 | [Disruption of structural covariance networks for language in autism is modulated by verbal ability.](https://neurosynth.org/studies/25445842/) | Sharda M, Khundrakpam BS, Evans AC, Singh NC | Brain structure & function |
| 73 | [DNA methylation of the oxytocin receptor gene predicts neural response to ambiguous social stimuli.](https://neurosynth.org/studies/23087634/) | Jack A, Connelly JJ, Morris JP | Frontiers in human neuroscience |
| 74 | [Do distinct atypical cortical networks process biological motion information in adults with Autism Spectrum Disorders?](https://neurosynth.org/studies/21888982/) | McKay LS, Simmons DR, McAleer P, Marjoram D, Piggot J, Pollick FE | NeuroImage |
| 75 | [Effects of intranasal oxytocin on the neural basis of face processing in autism spectrum disorder.](https://neurosynth.org/studies/23510581/) | Domes G, Heinrichs M, Kumbier E, Grossmann A, Hauenstein K, Herpertz SC | Biological psychiatry |
| 76 | [Equivalent neural responses in children and adolescents with and without autism during judgments of affect.](https://neurosynth.org/studies/24016745/) | Vander Wyk BC, Hoffman F, Pelphrey KA | Developmental cognitive neuroscience |
| 77 | [Evaluation of enhanced attention to local detail in anorexia nervosa using the embedded figures test; an FMRI study.](https://neurosynth.org/studies/23691129/) | Fonville L, Lao-Kaim NP, Giampietro V, Van den Eynde F, Davies H, Lounes N, Andrew C, Dalton J, Simmons A, Williams SC, Baron-Cohen S, Tchanturia K | PloS one |
| 78 | [Feature selection and classification of imbalanced datasets: application to PET images of children with autistic spectrum disorders.](https://neurosynth.org/studies/21600290/) | Duchesnay E, Cachia A, Boddaert N, Chabane N, Mangin JF, Martinot JL, Brunelle F, Zilbovicius M | NeuroImage |
| 79 | [Female children with autism spectrum disorder: an insight from mass-univariate and pattern classification analyses.](https://neurosynth.org/studies/21896334/) | Calderoni S, Retico A, Biagi L, Tancredi R, Muratori F, Tosetti M | NeuroImage |
| 80 | [Fractionation of social brain circuits in autism spectrum disorders.](https://neurosynth.org/studies/22791801/) | Gotts SJ, Simmons WK, Milbury LA, Wallace GL, Cox RW, Martin A | Brain : a journal of neurology |
| 81 | [Functional alterations in neural substrates of geometric reasoning in adults with high-functioning autism.](https://neurosynth.org/studies/22912831/) | Yamada T, Ohta H, Watanabe H, Kanai C, Tani M, Ohno T, Takayama Y, Iwanami A, Kato N, Hashimoto R | PloS one |
| 82 | [Functional anatomy of impaired selective attention and compensatory processing in autism.](https://neurosynth.org/studies/14561452/) | Belmonte MK, Yurgelun-Todd DA | Brain research. Cognitive brain research |
| 83 | [Functional brain networks and white matter underlying theory-of-mind in autism.](https://neurosynth.org/studies/22977198/) | Kana RK, Libero LE, Hu CP, Deshpande HD, Colburn JS | Social cognitive and affective neuroscience |
| 84 | [Functional connectivity for an "island of sparing" in autism spectrum disorder: an fMRI study of visual search.](https://neurosynth.org/studies/22495745/) | Keehn B, Shih P, Brenner LA, Townsend J, Muller RA | Human brain mapping |
| 85 | [Functional connectivity of the inferior frontal cortex changes with age in children with autism spectrum disorders: a fcMRI study of response inhibition.](https://neurosynth.org/studies/19068486/) | Lee PS, Yerys BE, Della Rosa A, Foss-Feig J, Barnes KA, James JD, VanMeter J, Vaidya CJ, Gaillard WD, Kenworthy LE | Cerebral cortex (New York, N.Y. : 1991) |
| 86 | [Functional deficits of the attentional networks in autism.](https://neurosynth.org/studies/23139910/) | Fan J, Bernardi S, Van Dam NT, Anagnostou E, Gu X, Martin L, Park Y, Liu X, Kolevzon A, Soorya L, Grodberg D, Hollander E, Hof PR | Brain and behavior |
| 87 | [Functional differentiation of posterior superior temporal sulcus in autism: a functional connectivity magnetic resonance imaging study.](https://neurosynth.org/studies/21601832/) | Shih P, Keehn B, Oram JK, Leyden KM, Keown CL, Muller RA | Biological psychiatry |
| 88 | [Functional Organization of the Action Observation Network in Autism: A Graph Theory Approach.](https://neurosynth.org/studies/26317222/) | Alaerts K, Geerlings F, Herremans L, Swinnen SP, Verhoeven J, Sunaert S, Wenderoth N | PloS one |
| 89 | [Gender-specific modulation of neural mechanisms underlying social reward processing by Autism Quotient.](https://neurosynth.org/studies/25944965/) | Barman A, Richter S, Soch J, Deibele A, Richter A, Assmann A, Wustenberg T, Walter H, Seidenbecher CI, Schott BH | Social cognitive and affective neuroscience |
| 90 | [Hard to "tune in": neural mechanisms of live face-to-face interaction with high-functioning autistic spectrum disorder.](https://neurosynth.org/studies/23060772/) | Tanabe HC, Kosaka H, Saito DN, Koike T, Hayashi MJ, Izuma K, Komeda H, Ishitobi M, Omori M, Munesue T, Okazawa H, Wada Y, Sadato N | Frontiers in human neuroscience |
| 91 | [Heterogeneity of neural mechanisms of response to pivotal response treatment.](https://neurosynth.org/studies/25370452/) | Ventola P, Yang DY, Friedman HE, Oosting D, Wolf J, Sukhodolsky DG, Pelphrey KA | Brain imaging and behavior |
| 92 | [Histological and magnetic resonance imaging assessment of cortical layering and thickness in autism spectrum disorders.](https://neurosynth.org/studies/16580643/) | Hutsler JJ, Love T, Zhang H | Biological psychiatry |
| 93 | [Indices of repetitive behaviour are correlated with patterns of intrinsic functional connectivity in youth with autism spectrum disorder.](https://neurosynth.org/studies/29453959/) | Traynor JM, Doyle-Thomas KAR, Hanford LC, Foster NE, Tryfon A, Hyde KL, Anagnostou E, Evans AC, Zwaigenbaum L, Hall GBC | Brain research |
| 94 | [Inferring a dual-stream model of mentalizing from associative white matter fibres disconnection.](https://neurosynth.org/studies/24519980/) | Herbet G, Lafargue G, Bonnetblanc F, Moritz-Gasser S, Menjot de Champfleur N, Duffau H | Brain : a journal of neurology |
| 95 | [Intact brain processing of musical emotions in autism spectrum disorder, but more cognitive load and arousal in happy vs. sad music.](https://neurosynth.org/studies/25076869/) | Gebauer L, Skewes J, Westphael G, Heaton P, Vuust P | Frontiers in neuroscience |
| 96 | [Inverse fluoxetine effects on inhibitory brain activation in non-comorbid boys with ADHD and with ASD.](https://neurosynth.org/studies/25533997/) | Chantiluke K, Barrett N, Giampietro V, Santosh P, Brammer M, Simmons A, Murphy DG, Rubia K | Psychopharmacology |
| 97 | [It's all in the eyes: subcortical and cortical activation during grotesqueness perception in autism.](https://neurosynth.org/studies/23342130/) | Zurcher NR, Donnelly N, Rogier O, Russo B, Hippolyte L, Hadwin J, Lemonnier E, Hadjikhani N | PloS one |
| 98 | [Language comprehension and brain function in individuals with an optimal outcome from autism.](https://neurosynth.org/studies/26862477/) | Eigsti IM, Stevens MC, Schultz RT, Barton M, Kelley E, Naigles L, Orinstein A, Troyb E, Fein DA | NeuroImage. Clinical |
| 99 | [Latent and Abnormal Functional Connectivity Circuits in Autism Spectrum Disorder.](https://neurosynth.org/studies/28377688/) | Chen S, Xing Y, Kang J | Frontiers in neuroscience |
| 100 | [Linked alterations in gray and white matter morphology in adults with high-functioning autism spectrum disorder: A multimodal brain imaging study.](https://neurosynth.org/studies/25610777/) | Itahashi T, Yamada T, Nakamura M, Watanabe H, Yamagata B, Jimbo D, Shioda S, Kuroda M, Toriizuka K, Kato N, Hashimoto R | NeuroImage. Clinical |
| 101 | [Lost for emotion words: What motor and limbic brain activity reveals about autism and semantic theory.](https://neurosynth.org/studies/25278250/) | Moseley RL, Shtyrov Y, Mohr B, Lombardo MV, Baron-Cohen S, Pulvermuller F | NeuroImage |
| 102 | [Mapping the brain in autism. A voxel-based MRI study of volumetric differences and intercorrelations in autism.](https://neurosynth.org/studies/15548557/) | McAlonan GM, Cheung V, Cheung C, Suckling J, Lam GY, Tai KS, Yip L, Murphy DG, Chua SE | Brain : a journal of neurology |
| 103 | [Multivariate searchlight classification of structural magnetic resonance imaging in children and adolescents with autism.](https://neurosynth.org/studies/21890111/) | Uddin LQ, Menon V, Young CB, Ryali S, Chen T, Khouzam A, Minshew NJ, Hardan AY | Biological psychiatry |
| 104 | [Neocerebellar contributions to social perception in adolescents with autism spectrum disorder.](https://neurosynth.org/studies/25170555/) | Jack A, Morris JP | Developmental cognitive neuroscience |
| 105 | [Neural and behavioral responses during self-evaluative processes differ in youth with and without autism.](https://neurosynth.org/studies/22760337/) | Pfeifer JH, Merchant JS, Colich NL, Hernandez LM, Rudie JD, Dapretto M | Journal of autism and developmental disorders |
| 106 | [Neural and cortisol responses during play with human and computer partners in children with autism.](https://neurosynth.org/studies/25552572/) | Edmiston EK, Merkle K, Corbett BA | Social cognitive and affective neuroscience |
| 107 | [Neural bases for impaired social cognition in schizophrenia and autism spectrum disorders.](https://neurosynth.org/studies/18053686/) | Pinkham AE, Hopfinger JB, Pelphrey KA, Piven J, Penn DL | Schizophrenia research |
| 108 | [Neural bases of gaze and emotion processing in children with autism spectrum disorders.](https://neurosynth.org/studies/22398976/) | Davies MS, Dapretto M, Sigman M, Sepeta L, Bookheimer SY | Brain and behavior |
| 109 | [Neural basis of irony comprehension in children with autism: the role of prosody and context.](https://neurosynth.org/studies/16481375/) | Wang AT, Lee SS, Sigman M, Dapretto M | Brain : a journal of neurology |
| 110 | [Neural basis of self and other representation in autism: an FMRI study of self-face recognition.](https://neurosynth.org/studies/18958161/) | Uddin LQ, Davies MS, Scott AA, Zaidel E, Bookheimer SY, Iacoboni M, Dapretto M | PloS one |
| 111 | [Neural Basis of Visual Attentional Orienting in Childhood Autism Spectrum Disorders.](https://neurosynth.org/studies/27696176/) | Murphy ER, Norr M, Strang JF, Kenworthy L, Gaillard WD, Vaidya CJ | Journal of autism and developmental disorders |
| 112 | [Neural correlate of autistic-like traits and a common allele in the oxytocin receptor gene.](https://neurosynth.org/studies/23946005/) | Saito Y, Suga M, Tochigi M, Abe O, Yahata N, Kawakubo Y, Liu X, Kawamura Y, Sasaki T, Kasai K, Yamasue H | Social cognitive and affective neuroscience |
| 113 | [Neural correlates of inhibition of socially relevant stimuli in adults with autism spectrum disorder.](https://neurosynth.org/studies/23962468/) | Duerden EG, Taylor MJ, Soorya LV, Wang T, Fan J, Anagnostou E | Brain research |
| 114 | [Neural correlates of moral reasoning in autism spectrum disorder.](https://neurosynth.org/studies/22569187/) | Schneider K, Pauly KD, Gossen A, Mevissen L, Michel TM, Gur RC, Schneider F, Habel U | Social cognitive and affective neuroscience |
| 115 | [Neural correlates of pragmatic language comprehension in autism spectrum disorders.](https://neurosynth.org/studies/19423680/) | Tesink CM, Buitelaar JK, Petersson KM, van der Gaag RJ, Kan CC, Tendolkar I, Hagoort P | Brain : a journal of neurology |
| 116 | [Neural Mechanisms of Emotion Regulation in Autism Spectrum Disorder.](https://neurosynth.org/studies/25618212/) | Richey JA, Damiano CR, Sabatino A, Rittenberg A, Petty C, Bizzell J, Voyvodic J, Heller AS, Coffman MC, Smoski M, Davidson RJ, Dichter GS | Journal of autism and developmental disorders |
| 117 | [Neural Mechanisms Underlying Conscious and Unconscious Gaze-Triggered Attentional Orienting in Autism Spectrum Disorder.](https://neurosynth.org/studies/28701942/) | Sato W, Kochiyama T, Uono S, Yoshimura S, Toichi M | Frontiers in human neuroscience |
| 118 | [Neural networks underlying language and social cognition during self-other processing in Autism spectrum disorders.](https://neurosynth.org/studies/28619530/) | Kana RK, Sartin EB, Stevens C Jr, Deshpande HD, Klein C, Klinger MR, Klinger LG | Neuropsychologia |
| 119 | [Neural responses to emotional expression information in high- and low-spatial frequency in autism: evidence for a cortical dysfunction.](https://neurosynth.org/studies/24782735/) | Corradi-Dell'acqua C, Schwartz S, Meaux E, Hubert B, Vuilleumier P, Deruelle C | Frontiers in human neuroscience |
| 120 | [Neural signatures of autism.](https://neurosynth.org/studies/21078973/) | Kaiser MD, Hudac CM, Shultz S, Lee SM, Cheung C, Berken AM, Deen B, Pitskel NB, Sugrue DR, Voos AC, Saulnier CA, Ventola P, Wolf JM, Klin A, Vander Wyk BC, Pelphrey KA | Proceedings of the National Academy of Sciences of the United States of America |
| 121 | [Neural substrates of numerosity estimation in autism.](https://neurosynth.org/studies/24639374/) | Meaux E, Taylor MJ, Pang EW, Vara AS, Batty M | Human brain mapping |
| 122 | [Neural systems for cognitive reappraisal in children and adolescents with autism spectrum disorder.](https://neurosynth.org/studies/25198094/) | Pitskel NB, Bolling DZ, Kaiser MD, Pelphrey KA, Crowley MJ | Developmental cognitive neuroscience |
| 123 | [Neuroanatomical Alterations in High-Functioning Adults with Autism Spectrum Disorder.](https://neurosynth.org/studies/27313505/) | Eilam-Stock T, Wu T, Spagna A, Egan LJ, Fan J | Frontiers in neuroscience |
| 124 | [No neural evidence of statistical learning during exposure to artificial languages in children with autism spectrum disorders.](https://neurosynth.org/studies/20303070/) | Scott-Van Zeeland AA, McNealy K, Wang AT, Sigman M, Bookheimer SY, Dapretto M | Biological psychiatry |
| 125 | [Noise Reduction in Arterial Spin Labeling Based Functional Connectivity Using Nuisance Variables.](https://neurosynth.org/studies/27601973/) | Jann K, Smith RX, Rios Piedra EA, Dapretto M, Wang DJ | Frontiers in neuroscience |
| 126 | [Oxytocin and vasopressin effects on the neural response to social cooperation are modulated by sex in humans.](https://neurosynth.org/studies/25416642/) | Feng C, Hackett PD, DeMarco AC, Chen X, Stair S, Haroon E, Ditzen B, Pagnoni G, Rilling JK | Brain imaging and behavior |
| 127 | [Oxytocin enhances brain function in children with autism.](https://neurosynth.org/studies/24297883/) | Gordon I, Vander Wyk BC, Bennett RH, Cordeaux C, Lucas MV, Eilbott JA, Zagoory-Sharon O, Leckman JF, Feldman R, Pelphrey KA | Proceedings of the National Academy of Sciences of the United States of America |
| 128 | [Oxytocin improves behavioural and neural deficits in inferring others' social emotions in autism.](https://neurosynth.org/studies/25149412/) | Aoki Y, Yahata N, Watanabe T, Takano Y, Kawakubo Y, Kuwabara H, Iwashiro N, Natsubori T, Inoue H, Suga M, Takao H, Sasaki H, Gonoi W, Kunimatsu A, Kasai K, Yamasue H | Brain : a journal of neurology |
| 129 | [Perception of biological motion in autism spectrum disorders.](https://neurosynth.org/studies/18262208/) | Freitag CM, Konrad C, Haberlen M, Kleser C, von Gontard A, Reith W, Troje NF, Krick C | Neuropsychologia |
| 130 | [Perception of social cues of danger in autism spectrum disorders.](https://neurosynth.org/studies/24324679/) | Zurcher NR, Rogier O, Boshyan J, Hippolyte L, Russo B, Gillberg N, Helles A, Ruest T, Lemonnier E, Gillberg C, Hadjikhani N | PloS one |
| 131 | [Phonological processing in first-degree relatives of individuals with autism: an fMRI study.](https://neurosynth.org/studies/22419478/) | Wilson LB, Tregellas JR, Slason E, Pasko BE, Hepburn S, Rojas DC | Human brain mapping |
| 132 | [Reduced cognitive control of response inhibition by the anterior cingulate cortex in autism spectrum disorders.](https://neurosynth.org/studies/20394829/) | Agam Y, Joseph RM, Barton JJ, Manoach DS | NeuroImage |
| 133 | [Reduced functional connectivity within and between 'social' resting state networks in autism spectrum conditions.](https://neurosynth.org/studies/22563003/) | von dem Hagen EA, Stoyanova RS, Baron-Cohen S, Calder AJ | Social cognitive and affective neuroscience |
| 134 | [Reduced functional integration and segregation of distributed neural systems underlying social and emotional information processing in autism spectrum disorders.](https://neurosynth.org/studies/21784971/) | Rudie JD, Shehzad Z, Hernandez LM, Colich NL, Bookheimer SY, Iacoboni M, Dapretto M | Cerebral cortex (New York, N.Y. : 1991) |
| 135 | [Reduced Gray Matter Volume in the Social Brain Network in Adults with Autism Spectrum Disorder.](https://neurosynth.org/studies/28824399/) | Sato W, Kochiyama T, Uono S, Yoshimura S, Kubota Y, Sawada R, Sakihama M, Toichi M | Frontiers in human neuroscience |
| 136 | [Regional homogeneity of fMRI time series in autism spectrum disorders.](https://neurosynth.org/studies/20381584/) | Shukla DK, Keehn B, Muller RA | Neuroscience letters |
| 137 | [Response monitoring, repetitive behaviour and anterior cingulate abnormalities in autism spectrum disorders (ASD).](https://neurosynth.org/studies/18550622/) | Thakkar KN, Polli FE, Joseph RM, Tuch DS, Hadjikhani N, Barton JJ, Manoach DS | Brain : a journal of neurology |
| 138 | [Resting State Functional Connectivity MRI among Spectral MEG Current Sources in Children on the Autism Spectrum.](https://neurosynth.org/studies/27375419/) | Datko M, Gougelet R, Huang MX, Pineda JA | Frontiers in neuroscience |
| 139 | [Resting-State Time-Varying Analysis Reveals Aberrant Variations of Functional Connectivity in Autism.](https://neurosynth.org/studies/27695408/) | Yao Z, Hu B, Xie Y, Zheng F, Liu G, Chen X, Zheng W | Frontiers in human neuroscience |
| 140 | [Reward circuitry function in autism during face anticipation and outcomes.](https://neurosynth.org/studies/22187105/) | Dichter GS, Richey JA, Rittenberg AM, Sabatino A, Bodfish JW | Journal of autism and developmental disorders |
| 141 | [Reward circuitry function in autism spectrum disorders.](https://neurosynth.org/studies/21148176/) | Dichter GS, Felder JN, Green SR, Rittenberg AM, Sasson NJ, Bodfish JW | Social cognitive and affective neuroscience |
| 142 | [Reward system dysfunction in autism spectrum disorders.](https://neurosynth.org/studies/22419119/) | Kohls G, Schulte-Ruther M, Nehrkorn B, Muller K, Fink GR, Kamp-Becker I, Herpertz-Dahlmann B, Schultz RT, Konrad K | Social cognitive and affective neuroscience |
| 143 | [Sex differences and autism: brain function during verbal fluency and mental rotation.](https://neurosynth.org/studies/22701630/) | Beacher FD, Radulescu E, Minati L, Baron-Cohen S, Lombardo MV, Lai MC, Walker A, Howard D, Gray MA, Harrison NA, Critchley HD | PloS one |
| 144 | [Sex Differences in the Default Mode Network with Regard to Autism Spectrum Traits: A Resting State fMRI Study.](https://neurosynth.org/studies/26600385/) | Jung M, Mody M, Saito DN, Tomoda A, Okazawa H, Wada Y, Kosaka H | PloS one |
| 145 | [Sex differences in the neural basis of false-belief and pragmatic language comprehension.](https://neurosynth.org/studies/25264229/) | Frank CK, Baron-Cohen S, Ganzel BL | NeuroImage |
| 146 | [Sex-linked white matter microstructure of the social and analytic brain.](https://neurosynth.org/studies/20633662/) | Chou KH, Cheng Y, Chen IY, Lin CP, Chu WC | NeuroImage |
| 147 | [Smaller insula and inferior frontal volumes in young adults with pervasive developmental disorders.](https://neurosynth.org/studies/20123027/) | Kosaka H, Omori M, Munesue T, Ishitobi M, Matsumura Y, Takahashi T, Narita K, Murata T, Saito DN, Uchiyama H, Morita T, Kikuchi M, Mizukami K, Okazawa H, Sadato N, Wada Y | NeuroImage |
| 148 | [Social cognition, the male brain and the autism spectrum.](https://neurosynth.org/studies/23300517/) | Hall J, Philip RC, Marwick K, Whalley HC, Romaniuk L, McIntosh AM, Santos I, Sprengelmeyer R, Johnstone EC, Stanfield AC, Young AW, Lawrie SM | PloS one |
| 149 | [Social perception in autism spectrum disorders: impaired category selectivity for dynamic but not static images in ventral temporal cortex.](https://neurosynth.org/studies/23019245/) | Weisberg J, Milleville SC, Kenworthy L, Wallace GL, Gotts SJ, Beauchamp MS, Martin A | Cerebral cortex (New York, N.Y. : 1991) |
| 150 | [Social stimuli interfere with cognitive control in autism.](https://neurosynth.org/studies/17321151/) | Dichter GS, Belger A | NeuroImage |
| 151 | [Social-cognitive brain function and connectivity during visual perspective-taking in autism and schizophrenia.](https://neurosynth.org/studies/28291690/) | Eack SM, Wojtalik JA, Keshavan MS, Minshew NJ | Schizophrenia research |
| 152 | [Sources of group differences in functional connectivity: an investigation applied to autism spectrum disorder.](https://neurosynth.org/studies/19646533/) | Jones TB, Bandettini PA, Kenworthy L, Case LK, Milleville SC, Martin A, Birn RM | NeuroImage |
| 153 | [Structural alterations of the social brain: a comparison between schizophrenia and autism.](https://neurosynth.org/studies/25188200/) | Radeloff D, Ciaramidaro A, Siniatchkin M, Hainz D, Schlitt S, Weber B, Poustka F, Bolte S, Walter H, Freitag CM | PloS one |
| 154 | [Structural and functional underconnectivity as a negative predictor for language in autism.](https://neurosynth.org/studies/24375710/) | Verly M, Verhoeven J, Zink I, Mantini D, Oudenhove LV, Lagae L, Sunaert S, Rommel N | Human brain mapping |
| 155 | [Synchrony between sensory and cognitive networks is associated with subclinical variation in autistic traits.](https://neurosynth.org/studies/25852527/) | Young JS, Smith DV, Coutlee CG, Huettel SA | Frontiers in human neuroscience |
| 156 | [The control of automatic imitation based on bottom-up and top-down cues to animacy: insights from brain and behavior.](https://neurosynth.org/studies/24742157/) | Klapper A, Ramsey R, Wigboldus D, Cross ES | Journal of cognitive neuroscience |
| 157 | [The Development of the Neural Substrates of Cognitive Control in Adolescents with Autism Spectrum Disorders.](https://neurosynth.org/studies/24209777/) | Solomon M, Yoon JH, Ragland JD, Niendam TA, Lesh TA, Fairbrother W, Carter CS | Biological psychiatry |
| 158 | [The effect of perceptual expectation on repetition suppression to faces is not modulated by variation in autistic traits.](https://neurosynth.org/studies/26613972/) | Ewbank MP, von dem Hagen EA, Powell TE, Henson RN, Calder AJ | Cortex; a journal devoted to the study of the nervous system and behavior |
| 159 | [The impact of serotonin transporter (5-HTTLPR) genotype on the development of resting-state functional connectivity in children and adolescents: a preliminary report.](https://neurosynth.org/studies/22032950/) | Wiggins JL, Bedoyan JK, Peltier SJ, Ashinoff S, Carrasco M, Weng SJ, Welsh RC, Martin DM, Monk CS | NeuroImage |
| 160 | [The neural basis of hyperlexic reading: an FMRI case study.](https://neurosynth.org/studies/14715131/) | Turkeltaub PE, Flowers DL, Verbalis A, Miranda M, Gareau L, Eden GF | Neuron |
| 161 | [The neural substrates of cognitive control deficits in autism spectrum disorders.](https://neurosynth.org/studies/19410583/) | Solomon M, Ozonoff SJ, Ursu S, Ravizza S, Cummings N, Ly S, Carter CS | Neuropsychologia |
| 162 | [The role of the amygdala in atypical gaze on emotional faces in autism spectrum disorders.](https://neurosynth.org/studies/22787032/) | Kliemann D, Dziobek I, Hatri A, Baudewig J, Heekeren HR | The Journal of neuroscience : the official journal of the Society for Neuroscience |
| 163 | [The superior temporal sulcus differentiates communicative and noncommunicative auditory signals.](https://neurosynth.org/studies/22360624/) | Shultz S, Vouloumanos A, Pelphrey K | Journal of cognitive neuroscience |
| 164 | [Trait-level temporal lobe hypoactivation to social exclusion in unaffected siblings of children and adolescents with autism spectrum disorders.](https://neurosynth.org/studies/26011751/) | Bolling DZ, Pelphrey KA, Vander Wyk BC | Developmental cognitive neuroscience |
| 165 | [Transdiagnostic deviant facial recognition for implicit negative emotion in autism and schizophrenia.](https://neurosynth.org/studies/29275843/) | Ciaramidaro A, Bolte S, Schlitt S, Hainz D, Poustka F, Weber B, Freitag C, Walter H | European neuropsychopharmacology : the journal of the European College of Neuropsychopharmacology |
| 166 | [Typical and atypical neurodevelopment for face specialization: an FMRI study.](https://neurosynth.org/studies/25479816/) | Joseph JE, Zhu X, Gundran A, Davies F, Clark JD, Ruble L, Glaser P, Bhatt RS | Journal of autism and developmental disorders |
| 167 | [Under-reactive but easily distracted: An fMRI investigation of attentional capture in autism spectrum disorder.](https://neurosynth.org/studies/26708773/) | Keehn B, Nair A, Lincoln AJ, Townsend J, Muller RA | Developmental cognitive neuroscience |
| 168 | [Using a self-organizing map algorithm to detect age-related changes in functional connectivity during rest in autism spectrum disorders.](https://neurosynth.org/studies/21047495/) | Wiggins JL, Peltier SJ, Ashinoff S, Weng SJ, Carrasco M, Welsh RC, Lord C, Monk CS | Brain research |
| 169 | [What Are You Doing With That Object? Comparing the Neural Responses of Action Understanding in Adolescents With and Without Autism.](https://neurosynth.org/studies/29168088/) | Pokorny JJ, Hatt NV, Rogers SJ, Rivera SM | Journal of autism and developmental disorders |
| 170 | [White Matter Integrity and Treatment-Based Change in Speech Performance in Minimally Verbal Children with Autism Spectrum Disorder.](https://neurosynth.org/studies/28424605/) | Chenausky K, Kernbach J, Norton A, Schlaug G | Frontiers in human neuroscience |

**Additional file 1: Table S4.** List of 130 studies extracted from Neurosynth under search term “bipolar disorder” on September 21, 2020

| **No.** | **Title** | **Authors** | **Journal** |
| --- | --- | --- | --- |
| 1 | [A developmental study on the neural circuitry mediating response flexibility in bipolar disorder.](https://neurosynth.org/studies/23958598/) | Weathers J, Brotman MA, Deveney CM, Kim P, Zarate C Jr, Fromm S, Pine D, Leibenluft E | Psychiatry research |
| 2 | [A functional MRI study of a paced motor activation task to evaluate frontal-subcortical circuit function in bipolar depression.](https://neurosynth.org/studies/17588725/) | Marchand WR, Lee JN, Thatcher GW, Jensen C, Stewart D, Dilda V, Thatcher J, Creem-Regehr SH | Psychiatry research |
| 3 | [A genome-wide supported variant in CACNA1C influences hippocampal activation during episodic memory encoding and retrieval.](https://neurosynth.org/studies/23860750/) | Krug A, Witt SH, Backes H, Dietsche B, Nieratschker V, Shah NJ, Nothen MM, Rietschel M, Kircher T | European archives of psychiatry and clinical neuroscience |
| 4 | [A group ICA based framework for evaluating resting fMRI markers when disease categories are unclear: application to schizophrenia, bipolar, and schizoaffective disorders.](https://neurosynth.org/studies/26216278/) | Du Y, Pearlson GD, Liu J, Sui J, Yu Q, He H, Castro E, Calhoun VD | NeuroImage |
| 5 | [A systematic review of associations between functional MRI activity and polygenic risk for schizophrenia and bipolar disorder.](https://neurosynth.org/studies/29748770/) | Dezhina Z, Ranlund S, Kyriakopoulos M, Williams SCR, Dima D | Brain imaging and behavior |
| 6 | [Abnormal baseline brain activity in bipolar depression: a resting state functional magnetic resonance imaging study.](https://neurosynth.org/studies/23017873/) | Liu CH, Li F, Li SF, Wang YJ, Tie CL, Wu HY, Zhou Z, Zhang D, Dong J, Yang Z, Wang CY | Psychiatry research |
| 7 | [Affective neural circuitry during facial emotion processing in pediatric bipolar disorder.](https://neurosynth.org/studies/17097071/) | Pavuluri MN, O'Connor MM, Harral E, Sweeney JA | Biological psychiatry |
| 8 | [Alteration of cortico-limbic-striatal neural system in major depressive disorder and bipolar disorder.](https://neurosynth.org/studies/28668591/) | Jiang X, Dai X, Kale Edmiston E, Zhou Q, Xu K, Zhou Y, Wu F, Kong L, Wei S, Zhou Y, Chang M, Geng H, Wang D, Wang Y, Cui W, Wang F, Tang Y | Journal of affective disorders |
| 9 | [Alterations in functional activation in euthymic bipolar disorder and schizophrenia during a working memory task.](https://neurosynth.org/studies/19449330/) | Hamilton LS, Altshuler LL, Townsend J, Bookheimer SY, Phillips OR, Fischer J, Woods RP, Mazziotta JC, Toga AW, Nuechterlein KH, Narr KL | Human brain mapping |
| 10 | [Alterations in regional homogeneity of resting-state brain activity in patients with major depressive disorder screening positive on the 32-item hypomania checklist (HCL-32).](https://neurosynth.org/studies/27280965/) | Yang H, Li L, Peng H, Liu T, Young AH, Angst J, Ye R, Rong H, Ji E, Qiu Y, Li L | Journal of affective disorders |
| 11 | [Altered affective processing in bipolar disorder: an fMRI study.](https://neurosynth.org/studies/23726779/) | Sagar KA, Dahlgren MK, Gonenc A, Gruber SA | Journal of affective disorders |
| 12 | [Altered Brain Activation during Emotional Face Processing in Relation to Both Diagnosis and Polygenic Risk of Bipolar Disorder.](https://neurosynth.org/studies/26222050/) | Tesli M, Kauppi K, Bettella F, Brandt CL, Kaufmann T, Espeseth T, Mattingsdal M, Agartz I, Melle I, Djurovic S, Westlye LT, Andreassen OA | PloS one |
| 13 | [Altered functional connectivity during self- and close other-reflection in patients with bipolar disorder with past psychosis and patients with schizophrenia.](https://neurosynth.org/studies/27693668/) | Zhang L, Vander Meer L, Opmeer EM, Marsman JC, Ruhe HG, Aleman A | Neuropsychologia |
| 14 | [Altered regional homogeneity in pediatric bipolar disorder during manic state: a resting-state fMRI study.](https://neurosynth.org/studies/23526961/) | Xiao Q, Zhong Y, Lu D, Gao W, Jiao Q, Lu G, Su L | PloS one |
| 15 | [Amplitude of low-frequency fluctuations in first-episode, drug-naive depressive patients: A 5-year retrospective study.](https://neurosynth.org/studies/28384269/) | Zhang K, Liu Z, Cao X, Yang C, Xu Y, Xu T, Xu C, Yang Z | PloS one |
| 16 | [Amygdala-prefrontal cortex resting-state functional connectivity varies with first depressive or manic episode in bipolar disorder.](https://neurosynth.org/studies/28130184/) | Wei S, Geng H, Jiang X, Zhou Q, Chang M, Zhou Y, Xu K, Tang Y, Wang F | Neuroscience letters |
| 17 | [An fMRI study of affective state and medication on cortical and subcortical brain regions during motor performance in bipolar disorder.](https://neurosynth.org/studies/12928105/) | Caligiuri MP, Brown GG, Meloy MJ, Eberson SC, Kindermann SS, Frank LR, Zorrilla LE, Lohr JB | Psychiatry research |
| 18 | [An fMRI study of attentional control in the context of emotional distracters in euthymic adults with bipolar disorder.](https://neurosynth.org/studies/22510433/) | Mullin BC, Perlman SB, Versace A, de Almeida JR, Labarbara EJ, Klein C, Ladouceur CD, Phillips ML | Psychiatry research |
| 19 | [Anomalous prefrontal-limbic activation and connectivity in youth at high-risk for bipolar disorder.](https://neurosynth.org/studies/28667891/) | Chang K, Garrett A, Kelley R, Howe M, Sanders EM, Acquaye T, Bararpour L, Li S, Singh M, Jo B, Hallmayer J, Reiss A | Journal of affective disorders |
| 20 | [Anticipation-related brain connectivity in bipolar and unipolar depression: a graph theory approach.](https://neurosynth.org/studies/27368345/) | Manelis A, Almeida JR, Stiffler R, Lockovich JC, Aslam HA, Phillips ML | Brain : a journal of neurology |
| 21 | [Are child-, adolescent-, and adult-onset depression one and the same disorder?](https://neurosynth.org/studies/11430841/) | Kaufman J, Martin A, King RA, Charney D | Biological psychiatry |
| 22 | [Assessment of white matter abnormalities in paranoid schizophrenia and bipolar mania patients.](https://neurosynth.org/studies/22079662/) | Cui L, Chen Z, Deng W, Huang X, Li M, Ma X, Huang C, Jiang L, Wang Y, Wang Q, Collier DA, Gong Q, Li T | Psychiatry research |
| 23 | [Behavioral and Neural Sustained Attention Deficits in Bipolar Disorder and Familial Risk of Bipolar Disorder.](https://neurosynth.org/studies/27837919/) | Pagliaccio D, Wiggins JL, Adleman NE, Harkins E, Curhan A, Towbin KE, Brotman MA, Pine DS, Leibenluft E | Biological psychiatry |
| 24 | [Bipolar and borderline patients display differential patterns of functional connectivity among resting state networks.](https://neurosynth.org/studies/24793833/) | Das P, Calhoun V, Malhi GS | NeuroImage |
| 25 | [Blunted activation in orbitofrontal cortex during mania: a functional magnetic resonance imaging study.](https://neurosynth.org/studies/16310510/) | Altshuler LL, Bookheimer SY, Townsend J, Proenza MA, Eisenberger N, Sabb F, Mintz J, Cohen MS | Biological psychiatry |
| 26 | [Brain gray matter phenotypes across the psychosis dimension.](https://neurosynth.org/studies/23177922/) | Ivleva EI, Bidesi AS, Thomas BP, Meda SA, Francis A, Moates AF, Witte B, Keshavan MS, Tamminga CA | Psychiatry research |
| 27 | [Brain structural and functional correlates of resilience to Bipolar Disorder.](https://neurosynth.org/studies/22363273/) | Frangou S | Frontiers in human neuroscience |
| 28 | [Brain structural signature of familial predisposition for bipolar disorder: replicable evidence for involvement of the right inferior frontal gyrus.](https://neurosynth.org/studies/22818781/) | Hajek T, Cullis J, Novak T, Kopecek M, Blagdon R, Propper L, Stopkova P, Duffy A, Hoschl C, Uher R, Paus T, Young LT, Alda M | Biological psychiatry |
| 29 | [CACNA1C risk variant and amygdala activity in bipolar disorder, schizophrenia and healthy controls.](https://neurosynth.org/studies/23437284/) | Tesli M, Skatun KC, Ousdal OT, Brown AA, Thoresen C, Agartz I, Melle I, Djurovic S, Jensen J, Andreassen OA | PloS one |
| 30 | [Cerebral Correlates of Abnormal Emotion Conflict Processing in Euthymic Bipolar Patients: A Functional MRI Study.](https://neurosynth.org/studies/26244883/) | Favre P, Polosan M, Pichat C, Bougerol T, Baciu M | PloS one |
| 31 | [Changes in brain activation during working memory and facial recognition tasks in patients with bipolar disorder with Lamotrigine monotherapy.](https://neurosynth.org/studies/17618089/) | Haldane M, Jogia J, Cobb A, Kozuch E, Kumari V, Frangou S | European neuropsychopharmacology : the journal of the European College of Neuropsychopharmacology |
| 32 | [Changes in gray matter volume in patients with bipolar disorder.](https://neurosynth.org/studies/15922309/) | Adler CM, Levine AD, DelBello MP, Strakowski SM | Biological psychiatry |
| 33 | [Classification of adolescent psychotic disorders using linear discriminant analysis.](https://neurosynth.org/studies/16797923/) | Pardo PJ, Georgopoulos AP, Kenny JT, Stuve TA, Findling RL, Schulz SC | Schizophrenia research |
| 34 | [Cognitive generation of affect in bipolar depression: an fMRI study.](https://neurosynth.org/studies/14984424/) | Malhi GS, Lagopoulos J, Ward PB, Kumari V, Mitchell PB, Parker GB, Ivanovski B, Sachdev P | The European journal of neuroscience |
| 35 | [Common and distinct structural features of schizophrenia and bipolar disorder: The European Network on Psychosis, Affective disorders and Cognitive Trajectory (ENPACT) study.](https://neurosynth.org/studies/29136642/) | Maggioni E, Crespo-Facorro B, Nenadic I, Benedetti F, Gaser C, Sauer H, Roiz-Santianez R, Poletti S, Marinelli V, Bellani M, Perlini C, Ruggeri M, Altamura AC, Diwadkar VA, Brambilla P | PloS one |
| 36 | [Contrasting and convergent patterns of amygdala connectivity in mania and depression: a resting-state study.](https://neurosynth.org/studies/25462396/) | Li M, Huang C, Deng W, Ma X, Han Y, Wang Q, Li Z, Guo W, Li Y, Jiang L, Lei W, Hu X, Gong Q, Merikangas KR, Palaniyappan L, Li T | Journal of affective disorders |
| 37 | [Contrasting variability patterns in the default mode and sensorimotor networks balance in bipolar depression and mania.](https://neurosynth.org/studies/27071087/) | Martino M, Magioncalda P, Huang Z, Conio B, Piaggio N, Duncan NW, Rocchi G, Escelsior A, Marozzi V, Wolff A, Inglese M, Amore M, Northoff G | Proceedings of the National Academy of Sciences of the United States of America |
| 38 | [Control-related frontal-striatal function is associated with past suicidal ideation and behavior in patients with recent-onset psychotic major mood disorders.](https://neurosynth.org/studies/26363618/) | Minzenberg MJ, Lesh TA, Niendam TA, Yoon JH, Cheng Y, Rhoades RN, Carter CS | Journal of affective disorders |
| 39 | [Cortical thickness in symptomatic and asymptomatic bipolar offspring.](https://neurosynth.org/studies/27107808/) | Hanford LC, Sassi RB, Minuzzi L, Hall GB | Psychiatry research. Neuroimaging |
| 40 | [Corticolimbic functional connectivity in adolescents with bipolar disorder.](https://neurosynth.org/studies/23185566/) | Wang F, Bobrow L, Liu J, Spencer L, Blumberg HP | PloS one |
| 41 | [Decreased Cingulate Cortex activation during cognitive control processing in bipolar disorder.](https://neurosynth.org/studies/28199893/) | Gruber SA, Dahlgren MK, Sagar KA, Gonenc A, Norris L, Cohen BM, Ongur D, Lewandowski KE | Journal of affective disorders |
| 42 | [Decreased medial prefrontal cortex activation during self-referential processing in bipolar mania.](https://neurosynth.org/studies/28551555/) | Herold D, Usnich T, Spengler S, Sajonz B, Bauer M, Bermpohl F | Journal of affective disorders |
| 43 | [Dissociable functional connectivity changes during the Stroop task relating to risk, resilience and disease expression in bipolar disorder.](https://neurosynth.org/studies/21570470/) | Pompei F, Dima D, Rubia K, Kumari V, Frangou S | NeuroImage |
| 44 | [Dorsolateral and dorsomedial prefrontal gray matter density changes associated with bipolar depression.](https://neurosynth.org/studies/19351579/) | Brooks JO 3rd, Bonner JC, Rosen AC, Wang PW, Hoblyn JC, Hill SJ, Ketter TA | Psychiatry research |
| 45 | [Effect of acute tryptophan depletion on pre-frontal engagement.](https://neurosynth.org/studies/16823591/) | Allen PP, Cleare AJ, Lee F, Fusar-Poli P, Tunstall N, Fu CH, Brammer MJ, McGuire PK | Psychopharmacology |
| 46 | [Effect of CACNA1C rs1006737 on neural correlates of verbal fluency in healthy individuals.](https://neurosynth.org/studies/19781653/) | Krug A, Nieratschker V, Markov V, Krach S, Jansen A, Zerres K, Eggermann T, Stocker T, Shah NJ, Treutlein J, Muhleisen TW, Kircher T | NeuroImage |
| 47 | [Effects of childhood trauma on working memory in affective and non-affective psychotic disorders.](https://neurosynth.org/studies/27090803/) | Quide Y, O'Reilly N, Rowland JE, Carr VJ, Elzinga BM, Green MJ | Brain imaging and behavior |
| 48 | [Effects of risk for bipolar disorder on brain function: A twin and family study.](https://neurosynth.org/studies/28392151/) | Sugihara G, Kane F, Picchioni MM, Chaddock CA, Kravariti E, Kalidindi S, Rijsdijk F, Toulopoulou T, Curtis VA, McDonald C, Murray RM, McGuire P | European neuropsychopharmacology : the journal of the European College of Neuropsychopharmacology |
| 49 | [Emotional response inhibition in bipolar disorder: a functional magnetic resonance imaging study of trait- and state-related abnormalities.](https://neurosynth.org/studies/22871393/) | Hummer TA, Hulvershorn LA, Karne HS, Gunn AD, Wang Y, Anand A | Biological psychiatry |
| 50 | [Evidence for deficient modulation of amygdala response by prefrontal cortex in bipolar mania.](https://neurosynth.org/studies/18063349/) | Foland LC, Altshuler LL, Bookheimer SY, Eisenberger N, Townsend J, Thompson PM | Psychiatry research |
| 51 | [fMRI abnormalities in dorsolateral prefrontal cortex during a working memory task in manic, euthymic and depressed bipolar subjects.](https://neurosynth.org/studies/20227857/) | Townsend J, Bookheimer SY, Foland-Ross LC, Sugar CA, Altshuler LL | Psychiatry research |
| 52 | [Frontal lobe hypoactivation in medication-free adults with bipolar II depression during response inhibition.](https://neurosynth.org/studies/25555505/) | Penfold C, Vizueta N, Townsend JD, Bookheimer SY, Altshuler LL | Psychiatry research |
| 53 | [Frontal-amygdala connectivity alterations during emotion downregulation in bipolar I disorder.](https://neurosynth.org/studies/22858151/) | Townsend JD, Torrisi SJ, Lieberman MD, Sugar CA, Bookheimer SY, Altshuler LL | Biological psychiatry |
| 54 | [Fronto-limbic dysfunction in mania pre-treatment and persistent amygdala over-activity post-treatment in pediatric bipolar disorder.](https://neurosynth.org/studies/21390505/) | Passarotti AM, Sweeney JA, Pavuluri MN | Psychopharmacology |
| 55 | [Fronto-temporal dysregulation in asymptomatic bipolar I patients: a paired associate functional MRI study.](https://neurosynth.org/studies/20063304/) | Glahn DC, Robinson JL, Tordesillas-Gutierrez D, Monkul ES, Holmes MK, Green MJ, Bearden CE | Human brain mapping |
| 56 | [Fronto-temporal spontaneous resting state functional connectivity in pediatric bipolar disorder.](https://neurosynth.org/studies/20739018/) | Dickstein DP, Gorrostieta C, Ombao H, Goldberg LD, Brazel AC, Gable CJ, Kelly C, Gee DG, Zuo XN, Castellanos FX, Milham MP | Biological psychiatry |
| 57 | [Frontostriatal neuroimaging findings differ in patients with bipolar disorder who have or do not have ADHD comorbidity.](https://neurosynth.org/studies/23057969/) | Townsend JD, Sugar CA, Walshaw PD, Vasquez RE, Foland-Ross LC, Moody TD, Bookheimer SY, McGough JJ, Altshuler LL | Journal of affective disorders |
| 58 | [Functional connectivity and neuronal variability of resting state activity in bipolar disorder-reduction and decoupling in anterior cortical midline structures.](https://neurosynth.org/studies/25307723/) | Magioncalda P, Martino M, Conio B, Escelsior A, Piaggio N, Presta A, Marozzi V, Rocchi G, Anastasio L, Vassallo L, Ferri F, Huang Z, Roccatagliata L, Pardini M, Northoff G, Amore M | Human brain mapping |
| 59 | [Functional connectivity during masked and unmasked face emotion processing in bipolar disorder.](https://neurosynth.org/studies/27814457/) | Tseng WL, Thomas LA, Harkins E, Stoddard J, Zarate CA Jr, Pine DS, Leibenluft E, Brotman MA | Psychiatry research. Neuroimaging |
| 60 | [Functional connectivity pattern during rest within the episodic memory network in association with episodic memory performance in bipolar disorder.](https://neurosynth.org/studies/25575881/) | Oertel-Knochel V, Reinke B, Matura S, Prvulovic D, Linden DE, Ven VV | Psychiatry research |
| 61 | [Functional Dysconnection of the Inferior Frontal Gyrus in Young People With Bipolar Disorder or at Genetic High Risk.](https://neurosynth.org/studies/28031150/) | Roberts G, Lord A, Frankland A, Wright A, Lau P, Levy F, Lenroot RK, Mitchell PB, Breakspear M | Biological psychiatry |
| 62 | [Functional magnetic resonance imaging brain activation in bipolar mania: evidence for disruption of the ventrolateral prefrontal-amygdala emotional pathway.](https://neurosynth.org/studies/21051038/) | Strakowski SM, Eliassen JC, Lamy M, Cerullo MA, Allendorfer JB, Madore M, Lee JH, Welge JA, DelBello MP, Fleck DE, Adler CM | Biological psychiatry |
| 63 | [Genetic liability for bipolar disorder is characterized by excess frontal activation in response to a working memory task.](https://neurosynth.org/studies/18571627/) | Drapier D, Surguladze S, Marshall N, Schulze K, Fern A, Hall MH, Walshe M, Murray RM, McDonald C | Biological psychiatry |
| 64 | [Genetic variation in G72 correlates with brain activation in the right middle temporal gyrus in a verbal fluency task in healthy individuals.](https://neurosynth.org/studies/20336655/) | Krug A, Markov V, Krach S, Jansen A, Zerres K, Eggermann T, Stocker T, Shah NJ, Nothen MM, Georgi A, Strohmaier J, Rietschel M, Kircher T | Human brain mapping |
| 65 | [Genetic variation in neuregulin1 is associated with differences in prefrontal engagement in children.](https://neurosynth.org/studies/19449332/) | Mechelli A, Viding E, Pettersson-Yeo W, Tognin S, McGuire PK | Human brain mapping |
| 66 | [Global prefrontal and fronto-amygdala dysconnectivity in bipolar I disorder with psychosis history.](https://neurosynth.org/studies/22980587/) | Anticevic A, Brumbaugh MS, Winkler AM, Lombardo LE, Barrett J, Corlett PR, Kober H, Gruber J, Repovs G, Cole MW, Krystal JH, Pearlson GD, Glahn DC | Biological psychiatry |
| 67 | [Hippocampal and Frontolimbic Function as Intermediate Phenotype for Psychosis: Evidence from Healthy Relatives and a Common Risk Variant in CACNA1C.](https://neurosynth.org/studies/24411473/) | Erk S, Meyer-Lindenberg A, Schmierer P, Mohnke S, Grimm O, Garbusow M, Haddad L, Poehland L, Muhleisen TW, Witt SH, Tost H, Kirsch P, Romanczuk-Seiferth N, Schott BH, Cichon S, Nothen MM, Rietschel M, Heinz A, Walter H | Biological psychiatry |
| 68 | [Human reward system activation is modulated by a single dose of olanzapine in healthy subjects in an event-related, double-blind, placebo-controlled fMRI study.](https://neurosynth.org/studies/17265148/) | Abler B, Erk S, Walter H | Psychopharmacology |
| 69 | [Identification of the neural correlates of cyclothymic temperament using a working memory task in fMRI.](https://neurosynth.org/studies/25282143/) | Kodama K, Terao T, Hatano K, Kohno K, Makino M, Mizokami Y, Kamei K, Katayama Y, Hoaki Y, Sakai A, Shirahama M, Watanabe S, Shimomura T, Fujiki M, Kochiyama T | Journal of affective disorders |
| 70 | [Identify changes of brain regional homogeneity in bipolar disorder and unipolar depression using resting-state FMRI.](https://neurosynth.org/studies/24324588/) | Liang MJ, Zhou Q, Yang KR, Yang XL, Fang J, Chen WL, Huang Z | PloS one |
| 71 | [Impaired sensory processing measured by functional MRI in Bipolar disorder manic and depressed mood states.](https://neurosynth.org/studies/28674759/) | Shaffer JJ Jr, Johnson CP, Fiedorowicz JG, Christensen GE, Wemmie JA, Magnotta VA | Brain imaging and behavior |
| 72 | [Impairment in semantic retrieval is associated with symptoms in schizophrenia but not bipolar disorder.](https://neurosynth.org/studies/22985694/) | Jamadar S, O'Neil KM, Pearlson GD, Ansari M, Gill A, Jagannathan K, Assaf M | Biological psychiatry |
| 73 | [Impairments in "top-down" processing in bipolar disorder: a simultaneous fMRI-GSR study.](https://neurosynth.org/studies/21493046/) | Lagopoulos J, Malhi G | Psychiatry research |
| 74 | [Increased cerebral blood flow among adolescents with bipolar disorder at rest is reduced following acute aerobic exercise.](https://neurosynth.org/studies/27792964/) | MacIntosh BJ, Shirzadi Z, Scavone A, Metcalfe AW, Islam AH, Korczak D, Goldstein BI | Journal of affective disorders |
| 75 | [Increased inferior frontal activation during word generation: a marker of genetic risk for schizophrenia but not bipolar disorder?](https://neurosynth.org/studies/19479729/) | Costafreda SG, Fu CH, Picchioni M, Kane F, McDonald C, Prata DP, Kalidindi S, Walshe M, Curtis V, Bramon E, Kravariti E, Marshall N, Toulopoulou T, Barker GJ, David AS, Brammer MJ, Murray RM, McGuire PK | Human brain mapping |
| 76 | [Interaction between effects of genes coding for dopamine and glutamate transmission on striatal and parahippocampal function.](https://neurosynth.org/studies/22438288/) | Pauli A, Prata DP, Mechelli A, Picchioni M, Fu CH, Chaddock CA, Kane F, Kalidindi S, McDonald C, Kravariti E, Toulopoulou T, Bramon E, Walshe M, Ehlert N, Georgiades A, Murray R, Collier DA, McGuire P | Human brain mapping |
| 77 | [Is aberrant functional connectivity a psychosis endophenotype? A resting state functional magnetic resonance imaging study.](https://neurosynth.org/studies/23746539/) | Khadka S, Meda SA, Stevens MC, Glahn DC, Calhoun VD, Sweeney JA, Tamminga CA, Keshavan MS, O'Neil K, Schretlen D, Pearlson GD | Biological psychiatry |
| 78 | [Lithium monotherapy associated clinical improvement effects on amygdala-ventromedial prefrontal cortex resting state connectivity in bipolar disorder.](https://neurosynth.org/studies/28772145/) | Altinay M, Karne H, Anand A | Journal of affective disorders |
| 79 | [Local functional connectivity alterations in schizophrenia, bipolar disorder, and major depressive disorder.](https://neurosynth.org/studies/29751242/) | Wei Y, Chang M, Womer FY, Zhou Q, Yin Z, Wei S, Zhou Y, Jiang X, Yao X, Duan J, Xu K, Zuo XN, Tang Y, Wang F | Journal of affective disorders |
| 80 | [Meta-analysis of functional magnetic resonance imaging studies of timing and cognitive control in schizophrenia and bipolar disorder: Evidence of a primary time deficit.](https://neurosynth.org/studies/28169089/) | Alustiza I, Radua J, Pla M, Martin R, Ortuno F | Schizophrenia research |
| 81 | [Network dysfunction of emotional and cognitive processes in those at genetic risk of bipolar disorder.](https://neurosynth.org/studies/26373604/) | Breakspear M, Roberts G, Green MJ, Nguyen VT, Frankland A, Levy F, Lenroot R, Mitchell PB | Brain : a journal of neurology |
| 82 | [Neural activation during facial emotion processing in unmedicated bipolar depression, euthymia, and mania.](https://neurosynth.org/studies/22206876/) | Hulvershorn LA, Karne H, Gunn AD, Hartwick SL, Wang Y, Hummer TA, Anand A | Biological psychiatry |
| 83 | [Neural basis of abnormal response to negative feedback in unmedicated mood disorders.](https://neurosynth.org/studies/18586109/) | Taylor Tavares JV, Clark L, Furey ML, Williams GB, Sahakian BJ, Drevets WC | NeuroImage |
| 84 | [Neural complexity as a potential translational biomarker for psychosis.](https://neurosynth.org/studies/27814962/) | Hager B, Yang AC, Brady R, Meda S, Clementz B, Pearlson GD, Sweeney JA, Tamminga C, Keshavan M | Journal of affective disorders |
| 85 | [Neural correlates of delusion in bipolar depression.](https://neurosynth.org/studies/24200366/) | Radaelli D, Poletti S, Gorni I, Locatelli C, Smeraldi E, Colombo C, Benedetti F | Psychiatry research |
| 86 | [Neural correlates of response inhibition in pediatric bipolar disorder and attention deficit hyperactivity disorder.](https://neurosynth.org/studies/19926457/) | Passarotti AM, Sweeney JA, Pavuluri MN | Psychiatry research |
| 87 | [Neural correlates of treatment response in depressed bipolar adolescents during emotion processing.](https://neurosynth.org/studies/23355265/) | Diler RS, Ladouceur CD, Segreti A, Almeida JR, Birmaher B, Axelson DA, Phillips ML, Pan LA | Brain imaging and behavior |
| 88 | [Neural recruitment during failed motor inhibition differentiates youths with bipolar disorder and severe mood dysregulation.](https://neurosynth.org/studies/22008364/) | Deveney CM, Connolly ME, Jenkins SE, Kim P, Fromm SJ, Pine DS, Leibenluft E | Biological psychology |
| 89 | [Neural response during explicit and implicit face processing varies developmentally in bipolar disorder.](https://neurosynth.org/studies/24493839/) | Deveney CM, Brotman MA, Thomas LA, Hinton KE, Muhrer EM, Reynolds RC, Adleman NE, Zarate CA Jr, Pine DS, Leibenluft E | Social cognitive and affective neuroscience |
| 90 | [Normal amygdala activation but deficient ventrolateral prefrontal activation in adults with bipolar disorder during euthymia.](https://neurosynth.org/studies/21854858/) | Foland-Ross LC, Bookheimer SY, Lieberman MD, Sugar CA, Townsend JD, Fischer J, Torrisi S, Penfold C, Madsen SK, Thompson PM, Altshuler LL | NeuroImage |
| 91 | [Olfactocentric paralimbic cortex morphology in adolescents with bipolar disorder.](https://neurosynth.org/studies/21666263/) | Wang F, Kalmar JH, Womer FY, Edmiston EE, Chepenik LG, Chen R, Spencer L, Blumberg HP | Brain : a journal of neurology |
| 92 | [Partial support for ZNF804A genotype-dependent alterations in prefrontal connectivity.](https://neurosynth.org/studies/22042765/) | Paulus FM, Krach S, Bedenbender J, Pyka M, Sommer J, Krug A, Knake S, Nothen MM, Witt SH, Rietschel M, Kircher T, Jansen A | Human brain mapping |
| 93 | [Pathological amygdala activation during working memory performance: Evidence for a pathophysiological trait marker in bipolar affective disorder.](https://neurosynth.org/studies/19603410/) | Gruber O, Tost H, Henseler I, Schmael C, Scherk H, Ende G, Ruf M, Falkai P, Rietschel M | Human brain mapping |
| 94 | [Pattern recognition of magnetic resonance imaging-based gray matter volume measurements classifies bipolar disorder and major depressive disorder.](https://neurosynth.org/studies/29156364/) | Rubin-Falcone H, Zanderigo F, Thapa-Chhetry B, Lan M, Miller JM, Sublette ME, Oquendo MA, Hellerstein DJ, McGrath PJ, Stewart JW, Mann JJ | Journal of affective disorders |
| 95 | [Pharmacotherapy impacts functional connectivity among affective circuits during response inhibition in pediatric mania.](https://neurosynth.org/studies/22004983/) | Pavuluri MN, Ellis JA, Wegbreit E, Passarotti AM, Stevens MC | Behavioural brain research |
| 96 | [Polygenic risk for five psychiatric disorders and cross-disorder and disorder-specific neural connectivity in two independent populations.](https://neurosynth.org/studies/28275544/) | Wang T, Zhang X, Li A, Zhu M, Liu S, Qin W, Li J, Yu C, Jiang T, Liu B | NeuroImage. Clinical |
| 97 | [Posterior cerebellar vermal deficits in bipolar disorder.](https://neurosynth.org/studies/23769608/) | Kim D, Cho HB, Dager SR, Yurgelun-Todd DA, Yoon S, Lee JH, Lee SH, Lee S, Renshaw PF, Lyoo IK | Journal of affective disorders |
| 98 | [Prefrontal-temporal gray matter deficits in bipolar disorder patients with persecutory delusions.](https://neurosynth.org/studies/19419772/) | Tost H, Ruf M, Schmal C, Schulze TG, Knorr C, Vollmert C, Bosshenz K, Ende G, Meyer-Lindenberg A, Henn FA, Rietschel M | Journal of affective disorders |
| 99 | [Preliminary investigation of the relationships between sleep duration, reward circuitry function, and mood dysregulation in youth offspring of parents with bipolar disorder.](https://neurosynth.org/studies/27442458/) | Soehner AM, Bertocci MA, Manelis A, Bebko G, Ladouceur CD, Graur S, Monk K, Bonar LK, Hickey MB, Axelson D, Goldstein BI, Goldstein TR, Birmaher B, Phillips ML | Journal of affective disorders |
| 100 | [Prolonged hemodynamic response during incidental facial emotion processing in inter-episode bipolar I disorder.](https://neurosynth.org/studies/23975275/) | Rosenfeld ES, Pearlson GD, Sweeney JA, Tamminga CA, Keshavan MS, Nonterah C, Stevens MC | Brain imaging and behavior |
| 101 | [Reduced activation to implicit affect induction in euthymic bipolar patients: an fMRI study.](https://neurosynth.org/studies/16837058/) | Malhi GS, Lagopoulos J, Owen AM, Ivanovski B, Shnier R, Sachdev P | Journal of affective disorders |
| 102 | [Reduced brain activation in euthymic bipolar patients during response inhibition: an event-related fMRI study.](https://neurosynth.org/studies/19442494/) | Kaladjian A, Jeanningros R, Azorin JM, Nazarian B, Roth M, Mazzola-Pomietto P | Psychiatry research |
| 103 | [Reduced gray matter volume in ventral prefrontal cortex but not amygdala in bipolar disorder: significant effects of gender and trait anxiety.](https://neurosynth.org/studies/19101126/) | Almeida JR, Akkal D, Hassel S, Travis MJ, Banihashemi L, Kerr N, Kupfer DJ, Phillips ML | Psychiatry research |
| 104 | [Reduced inferior frontal gyrus activation during response inhibition to emotional stimuli in youth at high risk of bipolar disorder.](https://neurosynth.org/studies/23245750/) | Roberts G, Green MJ, Breakspear M, McCormack C, Frankland A, Wright A, Levy F, Lenroot R, Chan HN, Mitchell PB | Biological psychiatry |
| 105 | [Resting-state functional network connectivity in prefrontal regions differs between unmedicated patients with bipolar and major depressive disorders.](https://neurosynth.org/studies/26551408/) | He H, Yu Q, Du Y, Vergara V, Victor TA, Drevets WC, Savitz JB, Jiang T, Sui J, Calhoun VD | Journal of affective disorders |
| 106 | [Reward anticipation revisited- evidence from an fMRI study in euthymic bipolar I patients and healthy first-degree relatives.](https://neurosynth.org/studies/28558365/) | Kollmann B, Scholz V, Linke J, Kirsch P, Wessa M | Journal of affective disorders |
| 107 | [Risk for affective disorders is associated with greater prefrontal gray matter volumes: A prospective longitudinal study.](https://neurosynth.org/studies/29527486/) | Macoveanu J, Baare W, Madsen KH, Kessing LV, Siebner HR, Vinberg M | NeuroImage. Clinical |
| 108 | [Shared dimensions of performance and activation dysfunction in cognitive control in females with mood disorders.](https://neurosynth.org/studies/25818869/) | Ryan KA, Dawson EL, Kassel MT, Weldon AL, Marshall DF, Meyers KK, Gabriel LB, Vederman AC, Weisenbach SL, McInnis MG, Zubieta JK, Langenecker SA | Brain : a journal of neurology |
| 109 | [Similarities and differences of white matter connectivity and water diffusivity in bipolar I and II disorder.](https://neurosynth.org/studies/22008503/) | Ha TH, Her JY, Kim JH, Chang JS, Cho HS, Ha K | Neuroscience letters |
| 110 | [Structural Abnormalities in Bipolar Euthymia: A Multicontrast Molecular Diffusion Imaging Study.](https://neurosynth.org/studies/24199669/) | Canales-Rodriguez EJ, Pomarol-Clotet E, Radua J, Sarro S, Alonso-Lana S, Del Mar Bonnin C, Goikolea JM, Maristany T, Garcia-Alvarez R, Vieta E, McKenna P, Salvador R | Biological psychiatry |
| 111 | [Structural and functional correlates of serum soluble IL-6 receptor level in patients with bipolar disorder.](https://neurosynth.org/studies/28558364/) | Tu PC, Li CT, Lin WC, Chen MH, Su TP, Bai YM | Journal of affective disorders |
| 112 | [Structural brain abnormalities in patients with type I bipolar disorder and suicidal behavior.](https://neurosynth.org/studies/28494347/) | Duarte DGG, Neves MCL, Albuquerque MR, Turecki G, Ding Y, de Souza-Duran FL, Busatto G, Correa H | Psychiatry research. Neuroimaging |
| 113 | [Structural brain features of borderline personality and bipolar disorders.](https://neurosynth.org/studies/23146251/) | Rossi R, Pievani M, Lorenzi M, Boccardi M, Beneduce R, Bignotti S, Borsci G, Cotelli M, Giannakopoulos P, Magni LR, Rillosi L, Rosini S, Rossi G, Frisoni GB | Psychiatry research |
| 114 | [Structural studies of the hypothalamus and its nuclei in mood disorders.](https://neurosynth.org/studies/22285717/) | Schindler S, Geyer S, Strauss M, Anwander A, Hegerl U, Turner R, Schonknecht P | Psychiatry research |
| 115 | [Subcortical and ventral prefrontal cortical neural responses to facial expressions distinguish patients with bipolar disorder and major depression.](https://neurosynth.org/studies/15013826/) | Lawrence NS, Williams AM, Surguladze S, Giampietro V, Brammer MJ, Andrew C, Frangou S, Ecker C, Phillips ML | Biological psychiatry |
| 116 | [The effects of DISC1 risk variants on brain activation in controls, patients with bipolar disorder and patients with schizophrenia.](https://neurosynth.org/studies/21376542/) | Chakirova G, Whalley HC, Thomson PA, Hennah W, Moorhead TW, Welch KA, Giles S, Hall J, Johnstone EC, Lawrie SM, Porteous DJ, Brown VJ, McIntosh AM | Psychiatry research |
| 117 | [The effects of mindfulness-based cognitive therapy in patients with bipolar disorder: a controlled functional MRI investigation.](https://neurosynth.org/studies/23790741/) | Ives-Deliperi VL, Howells F, Stein DJ, Meintjes EM, Horn N | Journal of affective disorders |
| 118 | [The effects of neuregulin1 on brain function in controls and patients with schizophrenia and bipolar disorder.](https://neurosynth.org/studies/18585932/) | Mechelli A, Prata DP, Fu CH, Picchioni M, Kane F, Kalidindi S, McDonald C, Demjaha A, Kravariti E, Toulopoulou T, Murray R, Collier DA, McGuire PK | NeuroImage |
| 119 | [The impact of psychosis on brain anatomy in bipolar disorder: A structural MRI study.](https://neurosynth.org/studies/29223329/) | Altamura AC, Maggioni E, Dhanoa T, Ciappolino V, Paoli RA, Cremaschi L, Prunas C, Orsenigo G, Caletti E, Cinnante CM, Triulzi FM, Dell'Osso B, Yatham L, Brambilla P | Journal of affective disorders |
| 120 | [The neural basis of familial risk and temperamental variation in individuals at high risk of bipolar disorder.](https://neurosynth.org/studies/21601834/) | Whalley HC, Sussmann JE, Chakirova G, Mukerjee P, Peel A, McKirdy J, Hall J, Johnstone EC, Lawrie SM, McIntosh AM | Biological psychiatry |
| 121 | [The neural correlates of cognitive control in bipolar I disorder: an fMRI study of medial frontal cortex activation during a Go/No-go task.](https://neurosynth.org/studies/23778236/) | Welander-Vatn A, Jensen J, Otnaess MK, Agartz I, Server A, Melle I, Andreassen OA | Neuroscience letters |
| 122 | [Time course of recovery showing initial prefrontal cortex changes at 16 weeks, extending to subcortical changes by 3 years in pediatric bipolar disorder.](https://neurosynth.org/studies/23517886/) | Yang H, Lu LH, Wu M, Stevens M, Wegbreit E, Fitzgerald J, Levitan B, Shankman S, Pavuluri MN | Journal of affective disorders |
| 123 | [Trait and state dependent functional impairments in bipolar disorder.](https://neurosynth.org/studies/21050725/) | Van der Schot A, Kahn R, Ramsey N, Nolen W, Vink M | Psychiatry research |
| 124 | [Vertex-based morphometry in euthymic bipolar disorder implicates striatal regions involved in psychomotor function.](https://neurosynth.org/studies/24508205/) | Liberg B, Ekman CJ, Sellgren C, Johansson A, Landen M | Psychiatry research |
| 125 | [Voxel-based morphometric analysis on the volume of gray matter in bipolar I disorder.](https://neurosynth.org/studies/21236649/) | Li M, Cui L, Deng W, Ma X, Huang C, Jiang L, Wang Y, Collier DA, Gong Q, Li T | Psychiatry research |
| 126 | [Voxel-based morphometry study of the insular cortex in bipolar depression.](https://neurosynth.org/studies/25218414/) | Tang LR, Liu CH, Jing B, Ma X, Li HY, Zhang Y, Li F, Wang YP, Yang Z, Wang CY | Psychiatry research |
| 127 | [Voxel-based study of structural changes in first-episode patients with bipolar disorder.](https://neurosynth.org/studies/17027928/) | Adler CM, DelBello MP, Jarvis K, Levine A, Adams J, Strakowski SM | Biological psychiatry |
| 128 | [Where, when, how high, and how long? The hemodynamics of emotional response in psychotropic-naive patients with adolescent bipolar disorder.](https://neurosynth.org/studies/23261134/) | Wegbreit E, Passarotti AM, Ellis JA, Wu M, Witowski N, Fitzgerald JM, Stevens MC, Pavuluri MN | Journal of affective disorders |
| 129 | [White matter density in patients with schizophrenia, bipolar disorder and their unaffected relatives.](https://neurosynth.org/studies/15939409/) | McIntosh AM, Job DE, Moorhead TW, Harrison LK, Lawrie SM, Johnstone EC | Biological psychiatry |
| 130 | [Whole brain expression of bipolar disorder associated genes: structural and genetic analyses.](https://neurosynth.org/studies/24941232/) | McCarthy MJ, Liang S, Spadoni AD, Kelsoe JR, Simmons AN | PloS one |

**Additional file 1: Table S5.** List of 92 studies extracted from Neurosynth under search term “compulsive disorder” on September 21, 2020

| **No.** | **Title** | **Authors** | **Journal** |
| --- | --- | --- | --- |
| 1 | [A functional magnetic resonance imaging study of inhibitory control in obsessive-compulsive disorder.](https://neurosynth.org/studies/19906516/) | Page LA, Rubia K, Deeley Q, Daly E, Toal F, Mataix-Cols D, Giampietro V, Schmitz N, Murphy DG | Psychiatry research |
| 2 | [A functional MRI comparison of patients with obsessive-compulsive disorder and normal controls during a Chinese character Stroop task.](https://neurosynth.org/studies/15970434/) | Nakao T, Nakagawa A, Yoshiura T, Nakatani E, Nabeyama M, Yoshizato C, Kudoh A, Tada K, Yoshioka K, Kawamoto M | Psychiatry research |
| 3 | [A manual and automated MRI study of anterior cingulate and orbito-frontal cortices, and caudate nucleus in obsessive-compulsive disorder: comparison with healthy controls and patients with schizophren](https://neurosynth.org/studies/15766634/) | Riffkin J, Yucel M, Maruff P, Wood SJ, Soulsby B, Olver J, Kyrios M, Velakoulis D, Pantelis C | Psychiatry research |
| 4 | [A phenotypic structure and neural correlates of compulsive behaviors in adolescents.](https://neurosynth.org/studies/24244633/) | Montigny C, Castellanos-Ryan N, Whelan R, Banaschewski T, Barker GJ, Buchel C, Gallinat J, Flor H, Mann K, Paillere-Martinot ML, Nees F, Lathrop M, Loth E, Paus T, Pausova Z, Rietschel M, Schumann G, Smolka MN, Struve M, Robbins TW, Garavan H, Conrod PJ | PloS one |
| 5 | [Aberrant anterior cingulate activation in obsessive-compulsive disorder is related to task complexity.](https://neurosynth.org/studies/22349440/) | Koch K, Wagner G, Schachtzabel C, Peikert G, Schultz CC, Sauer H, Schlosser RG | Neuropsychologia |
| 6 | [Aberrant error processing in relation to symptom severity in obsessive-compulsive disorder: A multimodal neuroimaging study.](https://neurosynth.org/studies/25057466/) | Agam Y, Greenberg JL, Isom M, Falkenstein MJ, Jenike E, Wilhelm S, Manoach DS | NeuroImage. Clinical |
| 7 | [Abnormal processing of deontological guilt in obsessive-compulsive disorder.](https://neurosynth.org/studies/23681167/) | Basile B, Mancini F, Macaluso E, Caltagirone C, Bozzali M | Brain structure & function |
| 8 | [Abnormal regional spontaneous neuronal activity associated with symptom severity in treatment-naive patients with obsessive-compulsive disorder revealed by resting-state functional MRI.](https://neurosynth.org/studies/28104431/) | Qiu L, Fu X, Wang S, Tang Q, Chen X, Cheng L, Zhang F, Zhou Z, Tian L | Neuroscience letters |
| 9 | [Abnormal Spontaneous Neural Activity in Obsessive-Compulsive Disorder: A Resting-State Functional Magnetic Resonance Imaging Study.](https://neurosynth.org/studies/23826251/) | Ping L, Su-Fang L, Hai-Ying H, Zhang-Ye D, Jia L, Zhi-Hua G, Hong-Fang X, Yu-Feng Z, Zhan-Jiang L | PloS one |
| 10 | [Abnormal striatal resting-state functional connectivity in adolescents with obsessive-compulsive disorder.](https://neurosynth.org/studies/26674413/) | Bernstein GA, Mueller BA, Schreiner MW, Campbell SM, Regan EK, Nelson PM, Houri AK, Lee SS, Zagoloff AD, Lim KO, Yacoub ES, Cullen KR | Psychiatry research. Neuroimaging |
| 11 | [Alterations of Gray and White Matter Networks in Patients with Obsessive-Compulsive Disorder: A Multimodal Fusion Analysis of Structural MRI and DTI Using mCCA+jICA.](https://neurosynth.org/studies/26038825/) | Kim SG, Jung WH, Kim SN, Jang JH, Kwon JS | PloS one |
| 12 | [Altered activation in fronto-striatal circuits during sequential processing of conflict in unmedicated adults with obsessive-compulsive disorder.](https://neurosynth.org/studies/23489416/) | Marsh R, Horga G, Parashar N, Wang Z, Peterson BS, Simpson HB | Biological psychiatry |
| 13 | [Altered brain activity during reward anticipation in pathological gambling and obsessive-compulsive disorder.](https://neurosynth.org/studies/23029329/) | Choi JS, Shin YC, Jung WH, Jang JH, Kang DH, Choi CH, Choi SW, Lee JY, Hwang JY, Kwon JS | PloS one |
| 14 | [Altered connectivity within and between the default mode, central executive, and salience networks in obsessive-compulsive disorder.](https://neurosynth.org/studies/28743059/) | Fan J, Zhong M, Gan J, Liu W, Niu C, Liao H, Zhang H, Yi J, Chan RCK, Tan C, Zhu X | Journal of affective disorders |
| 15 | [Altered EEG lagged coherence during rest in obsessive-compulsive disorder.](https://neurosynth.org/studies/23968842/) | Olbrich S, Olbrich H, Adamaszek M, Jahn I, Hegerl U, Stengler K | Clinical neurophysiology : official journal of the International Federation of Clinical Neurophysiology |
| 16 | [Altered emotional and BOLD responses to negative, positive and ambiguous performance feedback in OCD.](https://neurosynth.org/studies/23893850/) | Becker MP, Nitsch AM, Schlosser R, Koch K, Schachtzabel C, Wagner G, Miltner WH, Straube T | Social cognitive and affective neuroscience |
| 17 | [Altered function and connectivity of the medial frontal cortex in pediatric obsessive-compulsive disorder.](https://neurosynth.org/studies/20947065/) | Fitzgerald KD, Stern ER, Angstadt M, Nicholson-Muth KC, Maynor MR, Welsh RC, Hanna GL, Taylor SF | Biological psychiatry |
| 18 | [Altered inhibition-related frontolimbic connectivity in obsessive-compulsive disorder.](https://neurosynth.org/studies/26183689/) | van Velzen LS, de Wit SJ, Curcic-Blake B, Cath DC, de Vries FE, Veltman DJ, van der Werf YD, van den Heuvel OA | Human brain mapping |
| 19 | [Altered olfactory processing and increased insula activity in patients with obsessive-compulsive disorder: An fMRI study.](https://neurosynth.org/studies/28208068/) | Berlin HA, Stern ER, Ng J, Zhang S, Rosenthal D, Turetzky R, Tang C, Goodman W | Psychiatry research. Neuroimaging |
| 20 | [Altered source memory retrieval is associated with pathological doubt in obsessive-compulsive disorder.](https://neurosynth.org/studies/26315458/) | Olson CA, Hale LR, Hamilton N, Powell JN, Martin LE, Savage CR | Behavioural brain research |
| 21 | [An fMRI study in monozygotic twins discordant for obsessive-compulsive symptoms.](https://neurosynth.org/studies/18342423/) | den Braber A, Ent Dv, Blokland GA, van Grootheest DS, Cath DC, Veltman DJ, de Ruiter MB, Boomsma DI | Biological psychology |
| 22 | [BOLD response during visual perception of biological motion in obsessive-compulsive disorder : an fMRI study using the dynamic point-light animation paradigm.](https://neurosynth.org/studies/18587523/) | Jung WH, Gu BM, Kang DH, Park JY, Yoo SY, Choi CH, Lee JM, Kwon JS | European archives of psychiatry and clinical neuroscience |
| 23 | [Brain activation by disgust-inducing pictures in obsessive-compulsive disorder.](https://neurosynth.org/studies/14512216/) | Shapira NA, Liu Y, He AG, Bradley MM, Lessig MC, James GA, Stein DJ, Lang PJ, Goodman WK | Biological psychiatry |
| 24 | [Brain activation during cognitive planning in twins discordant or concordant for obsessive-compulsive symptoms.](https://neurosynth.org/studies/20823085/) | den Braber A, van 't Ent D, Cath DC, Wagner J, Boomsma DI, de Geus EJ | Brain : a journal of neurology |
| 25 | [Brain activation during implicit sequence learning in individuals with trichotillomania.](https://neurosynth.org/studies/17321724/) | Rauch SL, Wright CI, Savage CR, Martis B, McMullin KG, Wedig MM, Gold AL, Keuthen NJ | Psychiatry research |
| 26 | [Brain activation of patients with obsessive-compulsive disorder during neuropsychological and symptom provocation tasks before and after symptom improvement: a functional magnetic resonance imaging st](https://neurosynth.org/studies/15820711/) | Nakao T, Nakagawa A, Yoshiura T, Nakatani E, Nabeyama M, Yoshizato C, Kudoh A, Tada K, Yoshioka K, Kawamoto M, Togao O, Kanba S | Biological psychiatry |
| 27 | [Brain activation of the defensive and appetitive survival systems in obsessive compulsive disorder.](https://neurosynth.org/studies/24760279/) | Goncalves OF, Soares JM, Carvalho S, Leite J, Ganho A, Fernandes-Goncalves A, Frank B, Pocinho F, Relvas J, Carracedo A, Sampaio A | Brain imaging and behavior |
| 28 | [Brain alterations in low-frequency fluctuations across multiple bands in obsessive compulsive disorder.](https://neurosynth.org/studies/27771857/) | Gimenez M, Guinea-Izquierdo A, Villalta-Gil V, Martinez-Zalacain I, Segalas C, Subira M, Real E, Pujol J, Harrison BJ, Haro JM, Sato JR, Hoexter MQ, Cardoner N, Alonso P, Menchon JM, Soriano-Mas C | Brain imaging and behavior |
| 29 | [Brain correlates of negative and positive visuospatial priming in adults.](https://neurosynth.org/studies/16300966/) | Wright CI, Keuthen NJ, Savage CR, Martis B, Williams D, Wedig M, McMullin K, Rauch SL | NeuroImage |
| 30 | [Brain correlates of negative visuospatial priming in healthy children.](https://neurosynth.org/studies/15932793/) | Wright CI, McMullin K, Martis B, Fischer H, Rauch SL | Psychiatry research |
| 31 | [Brain corticostriatal systems and the major clinical symptom dimensions of obsessive-compulsive disorder.](https://neurosynth.org/studies/23200527/) | Harrison BJ, Pujol J, Cardoner N, Deus J, Alonso P, Lopez-Sola M, Contreras-Rodriguez O, Real E, Segalas C, Blanco-Hinojo L, Menchon JM, Soriano-Mas C | Biological psychiatry |
| 32 | [Brain Gray Matter Abnormalities in First-Episode, Treatment-Naive Children with Obsessive-Compulsive Disorder.](https://neurosynth.org/studies/27445736/) | Cheng B, Cai W, Wang X, Lei D, Guo Y, Yang X, Wu Q, Gong J, Gong Q, Ning G | Frontiers in behavioral neuroscience |
| 33 | [Brain network dysfunction in youth with obsessive-compulsive disorder induced by simple uni-manual behavior: The role of the dorsal anterior cingulate cortex.](https://neurosynth.org/studies/27992792/) | Friedman AL, Burgess A, Ramaseshan K, Easter P, Khatib D, Chowdury A, Arnold PD, Hanna GL, Rosenberg DR, Diwadkar VA | Psychiatry research. Neuroimaging |
| 34 | [Brain structural alterations in obsessive-compulsive disorder patients with autogenous and reactive obsessions.](https://neurosynth.org/studies/24098688/) | Subira M, Alonso P, Segalas C, Real E, Lopez-Sola C, Pujol J, Martinez-Zalacain I, Harrison BJ, Menchon JM, Cardoner N, Soriano-Mas C | PloS one |
| 35 | [Change the mind and you change the brain: effects of cognitive-behavioral therapy on the neural correlates of spider phobia.](https://neurosynth.org/studies/12595193/) | Paquette V, Levesque J, Mensour B, Leroux JM, Beaudoin G, Bourgouin P, Beauregard M | NeuroImage |
| 36 | [Cognitive control of a simple mental image in patients with obsessive--compulsive disorder.](https://neurosynth.org/studies/21507542/) | Kocak OM, Ozpolat AY, Atbasoglu C, Cicek M | Brain and cognition |
| 37 | [Comparative Multimodal Meta-analysis of Structural and Functional Brain Abnormalities in Autism Spectrum Disorder and Obsessive-Compulsive Disorder.](https://neurosynth.org/studies/27887721/) | Carlisi CO, Norman LJ, Lukito SS, Radua J, Mataix-Cols D, Rubia K | Biological psychiatry |
| 38 | [Comparison of brain activation patterns during executive function tasks in hoarding disorder and non-hoarding OCD.](https://neurosynth.org/studies/27522332/) | Hough CM, Luks TL, Lai K, Vigil O, Guillory S, Nongpiur A, Fekri SM, Kupferman E, Mathalon DH, Mathews CA | Psychiatry research. Neuroimaging |
| 39 | [Compensatory Frontoparietal Activity During Working Memory: An Endophenotype of Obsessive-Compulsive Disorder.](https://neurosynth.org/studies/24365484/) | de Vries FE, de Wit SJ, Cath DC, van der Werf YD, van der Borden V, van Rossum TB, van Balkom AJ, van der Wee NJ, Veltman DJ, van den Heuvel OA | Biological psychiatry |
| 40 | [Cortical thinning in obsessive compulsive disorder.](https://neurosynth.org/studies/17525985/) | Shin YW, Yoo SY, Lee JK, Ha TH, Lee KJ, Lee JM, Kim IY, Kim SI, Kwon JS | Human brain mapping |
| 41 | [Degree connectivity in body dysmorphic disorder and relationships with obsessive and compulsive symptoms.](https://neurosynth.org/studies/27514293/) | Beucke JC, Sepulcre J, Buhlmann U, Kathmann N, Moody T, Feusner JD | European neuropsychopharmacology : the journal of the European College of Neuropsychopharmacology |
| 42 | [Deontological and altruistic guilt: evidence for distinct neurobiological substrates.](https://neurosynth.org/studies/20842749/) | Basile B, Mancini F, Macaluso E, Caltagirone C, Frackowiak RS, Bozzali M | Human brain mapping |
| 43 | [Disorder-specific dysfunction in right inferior prefrontal cortex during two inhibition tasks in boys with attention-deficit hyperactivity disorder compared to boys with obsessive-compulsive disorder.](https://neurosynth.org/studies/19777552/) | Rubia K, Cubillo A, Smith AB, Woolley J, Heyman I, Brammer MJ | Human brain mapping |
| 44 | [Disorder-specific dysfunctions in patients with attention-deficit/hyperactivity disorder compared to patients with obsessive-compulsive disorder during interference inhibition and attention allocation](https://neurosynth.org/studies/21391250/) | Rubia K, Cubillo A, Woolley J, Brammer MJ, Smith A | Human brain mapping |
| 45 | [Doubt in the Insula: Risk Processing in Obsessive-Compulsive Disorder.](https://neurosynth.org/studies/27378883/) | Luigjes J, Figee M, Tobler PN, van den Brink W, de Kwaasteniet B, van Wingen G, Denys D | Frontiers in human neuroscience |
| 46 | [Dysfunctional Activation and Brain Network Profiles in Youth with Obsessive-Compulsive Disorder: A Focus on the Dorsal Anterior Cingulate during Working Memory.](https://neurosynth.org/studies/25852529/) | Diwadkar VA, Burgess A, Hong E, Rix C, Arnold PD, Hanna GL, Rosenberg DR | Frontiers in human neuroscience |
| 47 | [Dysfunctional brain circuitry in obsessive-compulsive disorder: source and coherence analysis of EEG rhythms.](https://neurosynth.org/studies/19683062/) | Velikova S, Locatelli M, Insacco C, Smeraldi E, Comi G, Leocani L | NeuroImage |
| 48 | [Dysfunctional reward circuitry in obsessive-compulsive disorder.](https://neurosynth.org/studies/21272861/) | Figee M, Vink M, de Geus F, Vulink N, Veltman DJ, Westenberg H, Denys D | Biological psychiatry |
| 49 | [Effectiveness of cognitive-coping therapy and alteration of resting-state brain function in obsessive-compulsive disorder.](https://neurosynth.org/studies/27792961/) | Zhao HZ, Wang CH, Gao ZZ, Ma JD, Huang P, Li HF, Sang DE, Shan XW, Kou SJ, Li ZR, Ma L, Zhang ZH, Zhang JH, Ouyang H, Lian HK, Zang YF, Hu XZ | Journal of affective disorders |
| 50 | [Event-related functional magnetic resonance imaging of response inhibition in obsessive-compulsive disorder.](https://neurosynth.org/studies/17511967/) | Roth RM, Saykin AJ, Flashman LA, Pixley HS, West JD, Mamourian AC | Biological psychiatry |
| 51 | [Evidence-based guidelines on the therapeutic use of repetitive transcranial magnetic stimulation (rTMS).](https://neurosynth.org/studies/25034472/) | Lefaucheur JP, Andre-Obadia N, Antal A, Ayache SS, Baeken C, Benninger DH, Cantello RM, Cincotta M, de Carvalho M, De Ridder D, Devanne H, Di Lazzaro V, Filipovic SR, Hummel FC, Jaaskelainen SK, Kimiskidis VK, Koch G, Langguth B, Nyffeler T, Oliviero A, Padberg F, Poulet E, Rossi S, Rossini PM, Rothwell JC, Schonfeldt-Lecuona C, Siebner HR, Slotema CW, Stagg CJ, Valls-Sole J, Ziemann U, Paulus W, Garcia-Larrea L | Clinical neurophysiology : official journal of the International Federation of Clinical Neurophysiology |
| 52 | [Fronto-cingulate effective connectivity in obsessive compulsive disorder: a study with fMRI and dynamic causal modeling.](https://neurosynth.org/studies/20162605/) | Schlosser RG, Wagner G, Schachtzabel C, Peikert G, Koch K, Reichenbach JR, Sauer H | Human brain mapping |
| 53 | [Functional and structural neural indices of risk aversion in obsessive-compulsive disorder (OCD).](https://neurosynth.org/studies/22959813/) | Admon R, Bleich-Cohen M, Weizmant R, Poyurovsky M, Faragian S, Hendler T | Psychiatry research |
| 54 | [Functional connectivity in fronto-subcortical circuitry during the resting state in obsessive-compulsive disorder.](https://neurosynth.org/studies/20302914/) | Jang JH, Kim JH, Jung WH, Choi JS, Jung MH, Lee JM, Choi CH, Kang DH, Kwon JS | Neuroscience letters |
| 55 | [Functional magnetic resonance imaging study of regional brain activation during implicit sequence learning in obsessive-compulsive disorder.](https://neurosynth.org/studies/16497278/) | Rauch SL, Wedig MM, Wright CI, Martis B, McMullin KG, Shin LM, Cannistraro PA, Wilhelm S | Biological psychiatry |
| 56 | [Functional MRI study of brain activation alterations in patients with obsessive-compulsive disorder after symptom improvement.](https://neurosynth.org/studies/18667293/) | Nabeyama M, Nakagawa A, Yoshiura T, Nakao T, Nakatani E, Togao O, Yoshizato C, Yoshioka K, Tomita M, Kanba S | Psychiatry research |
| 57 | [Global resting-state functional magnetic resonance imaging analysis identifies frontal cortex, striatal, and cerebellar dysconnectivity in obsessive-compulsive disorder.](https://neurosynth.org/studies/24314349/) | Anticevic A, Hu S, Zhang S, Savic A, Billingslea E, Wasylink S, Repovs G, Cole MW, Bednarski S, Krystal JH, Bloch MH, Li CS, Pittenger C | Biological psychiatry |
| 58 | [Hoarding disorder and obsessive-compulsive disorder show different patterns of neural activity during response inhibition.](https://neurosynth.org/studies/24389161/) | Tolin DF, Witt ST, Stevens MC | Psychiatry research |
| 59 | [Hyper-influence of the orbitofrontal cortex over the ventral striatum in obsessive-compulsive disorder.](https://neurosynth.org/studies/26395293/) | Abe Y, Sakai Y, Nishida S, Nakamae T, Yamada K, Fukui K, Narumoto J | European neuropsychopharmacology : the journal of the European College of Neuropsychopharmacology |
| 60 | [Hyperactive error responses and altered connectivity in ventromedial and frontoinsular cortices in obsessive-compulsive disorder.](https://neurosynth.org/studies/21144497/) | Stern ER, Welsh RC, Fitzgerald KD, Gehring WJ, Lister JJ, Himle JA, Abelson JL, Taylor SF | Biological psychiatry |
| 61 | [Intolerance of uncertainty correlates with insula activation during affective ambiguity.](https://neurosynth.org/studies/18079060/) | Simmons A, Matthews SC, Paulus MP, Stein MB | Neuroscience letters |
| 62 | [Itch and motivation to scratch: an investigation of the central and peripheral correlates of allergen- and histamine-induced itch in humans.](https://neurosynth.org/studies/16914620/) | Leknes SG, Bantick S, Willis CM, Wilkinson JD, Wise RG, Tracey I | Journal of neurophysiology |
| 63 | [Localization of cerebral functional deficits in patients with obsessive-compulsive disorder: a resting-state fMRI study.](https://neurosynth.org/studies/22331021/) | Hou J, Wu W, Lin Y, Wang J, Zhou D, Guo J, Gu S, He M, Ahmed S, Hu J, Qu W, Li H | Journal of affective disorders |
| 64 | [Morphologic and functional connectivity alterations of corticostriatal and default mode network in treatment-naive patients with obsessive-compulsive disorder.](https://neurosynth.org/studies/24358320/) | Hou J, Song L, Zhang W, Wu W, Wang J, Zhou D, Qu W, Guo J, Gu S, He M, Xie B, Li H | PloS one |
| 65 | [Multispectral brain morphometry in Tourette syndrome persisting into adulthood.](https://neurosynth.org/studies/21071387/) | Draganski B, Martino D, Cavanna AE, Hutton C, Orth M, Robertson MM, Critchley HD, Frackowiak RS | Brain : a journal of neurology |
| 66 | [Neural correlates associated with symptom provocation in pediatric obsessive compulsive disorder after a single session of sham-controlled repetitive transcranial magnetic stimulation.](https://neurosynth.org/studies/26228567/) | Pedapati E, DiFrancesco M, Wu S, Giovanetti C, Nash T, Mantovani A, Ammerman R, Harris E | Psychiatry research |
| 67 | [Neural correlates of anxiety associated with obsessive-compulsive symptom dimensions in normal volunteers.](https://neurosynth.org/studies/12644353/) | Mataix-Cols D, Cullen S, Lange K, Zelaya F, Andrew C, Amaro E, Brammer MJ, Williams SC, Speckens A, Phillips ML | Biological psychiatry |
| 68 | [Neural correlates of blink suppression and the buildup of a natural bodily urge.](https://neurosynth.org/studies/21906689/) | Berman BD, Horovitz SG, Morel B, Hallett M | NeuroImage |
| 69 | [Neural correlates of cognitive inflexibility during task-switching in obsessive-compulsive disorder.](https://neurosynth.org/studies/18065438/) | Gu BM, Park JY, Kang DH, Lee SJ, Yoo SY, Jo HJ, Choi CH, Lee JM, Kwon JS | Brain : a journal of neurology |
| 70 | [Neural correlates of processing harmonic expectancy violations in children and adolescents with OCD.](https://neurosynth.org/studies/26900566/) | Buse J, Roessner V | NeuroImage. Clinical |
| 71 | [Neural correlates of working memory deficits and associations to response inhibition in obsessive compulsive disorder.](https://neurosynth.org/studies/29159055/) | Heinzel S, Kaufmann C, Grutzmann R, Hummel R, Klawohn J, Riesel A, Bey K, Lennertz L, Wagner M, Kathmann N | NeuroImage. Clinical |
| 72 | [Neural dysfunction during temporal discounting in paediatric Attention-Deficit/Hyperactivity Disorder and Obsessive-Compulsive Disorder.](https://neurosynth.org/studies/28988149/) | Norman LJ, Carlisi CO, Christakou A, Chantiluke K, Murphy C, Simmons A, Giampietro V, Brammer M, Mataix-Cols D, Rubia K | Psychiatry research. Neuroimaging |
| 73 | [Neural response in obsessive-compulsive washers depends on individual fit of triggers.](https://neurosynth.org/studies/23630478/) | Baioui A, Pilgramm J, Merz CJ, Walter B, Vaitl D, Stark R | Frontiers in human neuroscience |
| 74 | [Neural responses of OCD patients towards disorder-relevant, generally disgust-inducing and fear-inducing pictures.](https://neurosynth.org/studies/15935263/) | Schienle A, Schafer A, Stark R, Walter B, Vaitl D | International journal of psychophysiology : official journal of the International Organization of Psychophysiology |
| 75 | [Neural responses to facial expressions of disgust but not fear are modulated by washing symptoms in OCD.](https://neurosynth.org/studies/17097073/) | Lawrence NS, An SK, Mataix-Cols D, Ruths F, Speckens A, Phillips ML | Biological psychiatry |
| 76 | [Neurocognitive endophenotypes of obsessive-compulsive disorder.](https://neurosynth.org/studies/17855376/) | Menzies L, Achard S, Chamberlain SR, Fineberg N, Chen CH, del Campo N, Sahakian BJ, Robbins TW, Bullmore E | Brain : a journal of neurology |
| 77 | [Presupplementary Motor Area Contributes to Altered Error Monitoring in Obsessive-Compulsive Disorder.](https://neurosynth.org/studies/25659234/) | Grutzmann R, Endrass T, Kaufmann C, Allen E, Eichele T, Kathmann N | Biological psychiatry |
| 78 | [Provocation of symmetry/ordering symptoms in Anorexia nervosa: a functional neuroimaging study.](https://neurosynth.org/studies/24844926/) | Suda M, Brooks SJ, Giampietro V, Uher R, Mataix-Cols D, Brammer MJ, Williams SC, Treasure J, Campbell IC | PloS one |
| 79 | [Quantitative morphology of the corpus callosum in obsessive-compulsive disorder.](https://neurosynth.org/studies/23453697/) | Lopez KC, Lalonde F, Mattai A, Wade B, Clasen L, Rapoport J, Giedd JN | Psychiatry research |
| 80 | [Reduced functional connectivity within the limbic cortico-striato-thalamo-cortical loop in unmedicated adults with obsessive-compulsive disorder.](https://neurosynth.org/studies/24123377/) | Posner J, Marsh R, Maia TV, Peterson BS, Gruber A, Simpson HB | Human brain mapping |
| 81 | [Regional homogeneity of spontaneous brain activity in adult patients with obsessive-compulsive disorder before and after cognitive behavioural therapy.](https://neurosynth.org/studies/26378734/) | Yang XY, Sun J, Luo J, Zhong ZX, Li P, Yao SM, Xiong HF, Huang FF, Li ZJ | Journal of affective disorders |
| 82 | [Response perseveration in stimulant dependence is associated with striatal dysfunction and can be ameliorated by a D(2/3) receptor agonist.](https://neurosynth.org/studies/21967987/) | Ersche KD, Roiser JP, Abbott S, Craig KJ, Muller U, Suckling J, Ooi C, Shabbir SS, Clark L, Sahakian BJ, Fineberg NA, Merlo-Pich EV, Robbins TW, Bullmore ET | Biological psychiatry |
| 83 | [Resting-state connectivity of the amygdala predicts response to cognitive behavioral therapy in obsessive compulsive disorder.](https://neurosynth.org/studies/26388257/) | Gottlich M, Kramer UM, Kordon A, Hohagen F, Zurowski B | Biological psychology |
| 84 | [Resting-state functional connectivity between fronto-parietal and default mode networks in obsessive-compulsive disorder.](https://neurosynth.org/studies/22570705/) | Stern ER, Fitzgerald KD, Welsh RC, Abelson JL, Taylor SF | PloS one |
| 85 | [Resting-state functional connectivity between right anterior insula and right orbital frontal cortex correlate with insight level in obsessive-compulsive disorder.](https://neurosynth.org/studies/28458998/) | Fan J, Zhong M, Zhu X, Gan J, Liu W, Niu C, Liao H, Zhang H, Yi J, Tan C | NeuroImage. Clinical |
| 86 | [Sex differences in neural responses to disgusting visual stimuli: implications for disgust-related psychiatric disorders.](https://neurosynth.org/studies/17306771/) | Caseras X, Mataix-Cols D, An SK, Lawrence NS, Speckens A, Giampietro V, Brammer MJ, Phillips ML | Biological psychiatry |
| 87 | [Shared and disorder-specific task-positive and default mode network dysfunctions during sustained attention in paediatric Attention-Deficit/Hyperactivity Disorder and obsessive/compulsive disorder.](https://neurosynth.org/studies/28529874/) | Norman LJ, Carlisi CO, Christakou A, Cubillo A, Murphy CM, Chantiluke K, Simmons A, Giampietro V, Brammer M, Mataix-Cols D, Rubia K | NeuroImage. Clinical |
| 88 | [Subjective uncertainty and limbic hyperactivation in obsessive-compulsive disorder.](https://neurosynth.org/studies/22461182/) | Stern ER, Welsh RC, Gonzalez R, Fitzgerald KD, Abelson JL, Taylor SF | Human brain mapping |
| 89 | [Switch the itch: a naturalistic follow-up study on the neural correlates of cognitive flexibility in obsessive-compulsive disorder.](https://neurosynth.org/studies/23693090/) | Vriend C, de Wit SJ, Remijnse PL, van Balkom AJ, Veltman DJ, van den Heuvel OA | Psychiatry research |
| 90 | [The major symptom dimensions of obsessive-compulsive disorder are mediated by partially distinct neural systems.](https://neurosynth.org/studies/18952675/) | van den Heuvel OA, Remijnse PL, Mataix-Cols D, Vrenken H, Groenewegen HJ, Uylings HB, van Balkom AJ, Veltman DJ | Brain : a journal of neurology |
| 91 | [The parametric, psychological, neuropsychological, and neuroanatomical properties of self and world evaluation.](https://neurosynth.org/studies/22348093/) | Simmons AN, Thayer RE, Spadoni AD, Matthews SC, Strigo IA, Tapert SF | PloS one |
| 92 | [Widespread abnormality of the gamma-aminobutyric acid-ergic system in Tourette syndrome.](https://neurosynth.org/studies/22577221/) | Lerner A, Bagic A, Simmons JM, Mari Z, Bonne O, Xu B, Kazuba D, Herscovitch P, Carson RE, Murphy DL, Drevets WC, Hallett M | Brain : a journal of neurology |

**Additional file 1: Table S6.** List of 77 studies extracted from Neurosynth under search term “major depression” on September 21, 2020

| **No.** | **Title** | **Authors** | **Journal** |
| --- | --- | --- | --- |
| 1 | [A failure of suppression within the default mode network in depressed adolescents with compulsive internet game play.](https://neurosynth.org/studies/26802508/) | Han DH, Kim SM, Bae S, Renshaw PF, Anderson JS | Journal of affective disorders |
| 2 | [A genome-wide supported variant in CACNA1C influences hippocampal activation during episodic memory encoding and retrieval.](https://neurosynth.org/studies/23860750/) | Krug A, Witt SH, Backes H, Dietsche B, Nieratschker V, Shah NJ, Nothen MM, Rietschel M, Kircher T | European archives of psychiatry and clinical neuroscience |
| 3 | [A longitudinal functional magnetic resonance imaging study of verbal working memory in depression after antidepressant therapy.](https://neurosynth.org/studies/17601497/) | Walsh ND, Williams SC, Brammer MJ, Bullmore ET, Kim J, Suckling J, Mitterschiffthaler MT, Cleare AJ, Pich EM, Mehta MA, Fu CH | Biological psychiatry |
| 4 | [A Neurocomputational Account of How Inflammation Enhances Sensitivity to Punishments Versus Rewards.](https://neurosynth.org/studies/26359113/) | Harrison NA, Voon V, Cercignani M, Cooper EA, Pessiglione M, Critchley HD | Biological psychiatry |
| 5 | [A pilot resting-state functional connectivity study of the kynurenine pathway in adolescents with depression and healthy controls.](https://neurosynth.org/studies/29254065/) | DeWitt SJ, Bradley KA, Lin N, Yu C, Gabbay V | Journal of affective disorders |
| 6 | [Abnormal neural filtering of irrelevant visual information in depression.](https://neurosynth.org/studies/19193886/) | Desseilles M, Balteau E, Sterpenich V, Dang-Vu TT, Darsaud A, Vandewalle G, Albouy G, Salmon E, Peters F, Schmidt C, Schabus M, Gais S, Degueldre C, Phillips C, Luxen A, Ansseau M, Maquet P, Schwartz S | The Journal of neuroscience : the official journal of the Society for Neuroscience |
| 7 | [Abnormal temporal difference reward-learning signals in major depression.](https://neurosynth.org/studies/18579575/) | Kumar P, Waiter G, Ahearn T, Milders M, Reid I, Steele JD | Brain : a journal of neurology |
| 8 | [Acute vagus nerve stimulation using different pulse widths produces varying brain effects.](https://neurosynth.org/studies/15050863/) | Mu Q, Bohning DE, Nahas Z, Walker J, Anderson B, Johnson KA, Denslow S, Lomarev M, Moghadam P, Chae JH, George MS | Biological psychiatry |
| 9 | [Alteration of spontaneous neuronal activity within the salience network in partially remitted depression.](https://neurosynth.org/studies/25553621/) | Liu CH, Ma X, Song LP, Tang LR, Jing B, Zhang Y, Li F, Zhou Z, Fan J, Wang CY | Brain research |
| 10 | [Alterations in regional homogeneity of spontaneous brain activity in late-life subthreshold depression.](https://neurosynth.org/studies/23301035/) | Ma Z, Li R, Yu J, He Y, Li J | PloS one |
| 11 | [Altered brain function underlying verbal memory encoding and retrieval in psychotic major depression.](https://neurosynth.org/studies/23149036/) | Kelley R, Garrett A, Cohen J, Gomez R, Lembke A, Keller J, Reiss AL, Schatzberg A | Psychiatry research |
| 12 | [Altered cerebellar functional connectivity with intrinsic connectivity networks in adults with major depressive disorder.](https://neurosynth.org/studies/22724025/) | Liu L, Zeng LL, Li Y, Ma Q, Li B, Shen H, Hu D | PloS one |
| 13 | [Altered cerebellar-cerebral resting-state functional connectivity reliably identifies major depressive disorder.](https://neurosynth.org/studies/23228724/) | Ma Q, Zeng LL, Shen H, Liu L, Hu D | Brain research |
| 14 | [Altered emotional interference processing in affective and cognitive-control brain circuitry in major depression.](https://neurosynth.org/studies/17719567/) | Fales CL, Barch DM, Rundle MM, Mintun MA, Snyder AZ, Cohen JD, Mathews J, Sheline YI | Biological psychiatry |
| 15 | [Altered Intrinsic Functional Brain Architecture in Children at Familial Risk of Major Depression.](https://neurosynth.org/studies/26826874/) | Chai XJ, Hirshfeld-Becker D, Biederman J, Uchida M, Doehrmann O, Leonard JA, Salvatore J, Kenworthy T, Brown A, Kagan E, de Los Angeles C, Gabrieli JDE, Whitfield-Gabrieli S | Biological psychiatry |
| 16 | [Altered neural function during episodic memory encoding and retrieval in major depression.](https://neurosynth.org/studies/24639328/) | Dietsche B, Backes H, Stratmann M, Konrad C, Kircher T, Krug A | Human brain mapping |
| 17 | [An analysis of functional neuroimaging studies of dorsolateral prefrontal cortical activity in depression.](https://neurosynth.org/studies/17029760/) | Fitzgerald PB, Oxley TJ, Laird AR, Kulkarni J, Egan GF, Daskalakis ZJ | Psychiatry research |
| 18 | [Antidepressant short-term and long-term brain effects during self-referential processing in major depression.](https://neurosynth.org/studies/26655583/) | Delaveau P, Jabourian M, Lemogne C, Allaili N, Choucha W, Girault N, Lehericy S, Laredo J, Fossati P | Psychiatry research. Neuroimaging |
| 19 | [Antidepressant treatment normalizes hypoactivity in dorsolateral prefrontal cortex during emotional interference processing in major depression.](https://neurosynth.org/studies/18559283/) | Fales CL, Barch DM, Rundle MM, Mintun MA, Mathews J, Snyder AZ, Sheline YI | Journal of affective disorders |
| 20 | [Antidepressant treatment normalizes white matter volume in patients with major depression.](https://neurosynth.org/studies/22957005/) | Zeng LL, Liu L, Liu Y, Shen H, Li Y, Hu D | PloS one |
| 21 | [Association between habenula dysfunction and motivational symptoms in unmedicated major depressive disorder.](https://neurosynth.org/studies/28575424/) | Liu WH, Valton V, Wang LZ, Zhu YH, Roiser JP | Social cognitive and affective neuroscience |
| 22 | [Attentional modulation of emotional stimulus processing in patients with major depression--alterations in prefrontal cortical regions.](https://neurosynth.org/studies/19632301/) | Bermpohl F, Walter M, Sajonz B, Lucke C, Hagele C, Sterzer P, Adli M, Heinz A, Northoff G | Neuroscience letters |
| 23 | [Blood pressure and white matter integrity in geriatric depression.](https://neurosynth.org/studies/18805589/) | Hoptman MJ, Gunning-Dixon FM, Murphy CF, Ardekani BA, Hrabe J, Lim KO, Etwaroo GR, Kanellopoulos D, Alexopoulos GS | Journal of affective disorders |
| 24 | [Brain functional connectivity correlates of coping styles.](https://neurosynth.org/studies/29572771/) | Santarnecchi E, Sprugnoli G, Tatti E, Mencarelli L, Neri F, Momi D, Di Lorenzo G, Pascual-Leone A, Rossi S, Rossi A | Cognitive, affective & behavioral neuroscience |
| 25 | [Brain imaging correlates of depressive symptom severity and predictors of symptom improvement after antidepressant treatment.](https://neurosynth.org/studies/17217921/) | Chen CH, Ridler K, Suckling J, Williams S, Fu CH, Merlo-Pich E, Bullmore E | Biological psychiatry |
| 26 | [Cannabinoid receptor 1 (CNR1) gene: impact on antidepressant treatment response and emotion processing in major depression.](https://neurosynth.org/studies/18579347/) | Domschke K, Dannlowski U, Ohrmann P, Lawford B, Bauer J, Kugel H, Heindel W, Young R, Morris P, Arolt V, Deckert J, Suslow T, Baune BT | European neuropsychopharmacology : the journal of the European College of Neuropsychopharmacology |
| 27 | [Change the mind and you change the brain: effects of cognitive-behavioral therapy on the neural correlates of spider phobia.](https://neurosynth.org/studies/12595193/) | Paquette V, Levesque J, Mensour B, Leroux JM, Beaudoin G, Bourgouin P, Beauregard M | NeuroImage |
| 28 | [Childhood maltreatment is associated with an automatic negative emotion processing bias in the amygdala.](https://neurosynth.org/studies/22696400/) | Dannlowski U, Kugel H, Huber F, Stuhrmann A, Redlich R, Grotegerd D, Dohm K, Sehlmeyer C, Konrad C, Baune BT, Arolt V, Heindel W, Zwitserlood P, Suslow T | Human brain mapping |
| 29 | [Cognitive control and brain resources in major depression: an fMRI study using the n-back task.](https://neurosynth.org/studies/15955496/) | Harvey PO, Fossati P, Pochon JB, Levy R, Lebastard G, Lehericy S, Allilaire JF, Dubois B | NeuroImage |
| 30 | [Cortico-limbic response to personally challenging emotional stimuli after complete recovery from depression.](https://neurosynth.org/studies/19176279/) | Hooley JM, Gruber SA, Parker HA, Guillaumot J, Rogowska J, Yurgelun-Todd DA | Psychiatry research |
| 31 | [Cross-sectional and longitudinal assessment of structural brain alterations in melancholic depression.](https://neurosynth.org/studies/20875637/) | Soriano-Mas C, Hernandez-Ribas R, Pujol J, Urretavizcaya M, Deus J, Harrison BJ, Ortiz H, Lopez-Sola M, Menchon JM, Cardoner N | Biological psychiatry |
| 32 | [Crossmodal emotional integration in major depression.](https://neurosynth.org/studies/23576809/) | Muller VI, Cieslik EC, Kellermann TS, Eickhoff SB | Social cognitive and affective neuroscience |
| 33 | [Decreased regional homogeneity in insula and cerebellum: a resting-state fMRI study in patients with major depression and subjects at high risk for major depression.](https://neurosynth.org/studies/20493670/) | Liu Z, Xu C, Xu Y, Wang Y, Zhao B, Lv Y, Cao X, Zhang K, Du C | Psychiatry research |
| 34 | [Difference in amplitude of low-frequency fluctuation between currently depressed and remitted females with major depressive disorder.](https://neurosynth.org/studies/24121137/) | Jing B, Liu CH, Ma X, Yan HG, Zhuo ZZ, Zhang Y, Wang SH, Li HY, Wang CY | Brain research |
| 35 | [Early impact of 5-HTTLPR polymorphism on the neural correlates of sadness.](https://neurosynth.org/studies/20851164/) | Fortier E, Noreau A, Lepore F, Boivin M, Perusse D, Rouleau GA, Beauregard M | Neuroscience letters |
| 36 | [Effect of CACNA1C rs1006737 on neural correlates of verbal fluency in healthy individuals.](https://neurosynth.org/studies/19781653/) | Krug A, Nieratschker V, Markov V, Krach S, Jansen A, Zerres K, Eggermann T, Stocker T, Shah NJ, Treutlein J, Muhleisen TW, Kircher T | NeuroImage |
| 37 | [Emotion-related brain activity to conflicting socio-emotional cues in unmedicated depression.](https://neurosynth.org/studies/23769293/) | Greening SG, Osuch EA, Williamson PC, Mitchell DG | Journal of affective disorders |
| 38 | [Failure to regulate: counterproductive recruitment of top-down prefrontal-subcortical circuitry in major depression.](https://neurosynth.org/studies/17699669/) | Johnstone T, van Reekum CM, Urry HL, Kalin NH, Davidson RJ | The Journal of neuroscience : the official journal of the Society for Neuroscience |
| 39 | [Fronto-striatal correlates of impaired implicit sequence learning in major depression: an fMRI study.](https://neurosynth.org/studies/20219248/) | Naismith SL, Lagopoulos J, Ward PB, Davey CG, Little C, Hickie IB | Journal of affective disorders |
| 40 | [Functional and structural brain correlates of risk for major depression in children with familial depression.](https://neurosynth.org/studies/26106565/) | Chai XJ, Hirshfeld-Becker D, Biederman J, Uchida M, Doehrmann O, Leonard JA, Salvatore J, Kenworthy T, Brown A, Kagan E, de Los Angeles C, Whitfield-Gabrieli S, Gabrieli JD | NeuroImage. Clinical |
| 41 | [Functional brain activation to emotionally valenced faces in school-aged children with a history of preschool-onset major depression.](https://neurosynth.org/studies/22770650/) | Barch DM, Gaffrey MS, Botteron KN, Belden AC, Luby JL | Biological psychiatry |
| 42 | [Functional connectivity bias of the orbitofrontal cortex in drug-free patients with major depression.](https://neurosynth.org/studies/19811772/) | Frodl T, Bokde AL, Scheuerecker J, Lisiecka D, Schoepf V, Hampel H, Moller HJ, Bruckmann H, Wiesmann M, Meisenzahl E | Biological psychiatry |
| 43 | [Functional connectivity in apathy of late-life depression: a preliminary study.](https://neurosynth.org/studies/23261142/) | Alexopoulos GS, Hoptman MJ, Yuen G, Kanellopoulos D, Seirup JK, Lim KO, Gunning FM | Journal of affective disorders |
| 44 | [Functional magnetic resonance imaging correlates of emotion recognition and voluntary attentional regulation in depression: A generalized psycho-physiological interaction study.](https://neurosynth.org/studies/27814960/) | Tozzi L, Doolin K, Farrel C, Joseph S, O'Keane V, Frodl T | Journal of affective disorders |
| 45 | [Genetic variation in G72 correlates with brain activation in the right middle temporal gyrus in a verbal fluency task in healthy individuals.](https://neurosynth.org/studies/20336655/) | Krug A, Markov V, Krach S, Jansen A, Zerres K, Eggermann T, Stocker T, Shah NJ, Nothen MM, Georgi A, Strohmaier J, Rietschel M, Kircher T | Human brain mapping |
| 46 | [Habitual emotion regulation strategies and depressive symptoms in healthy subjects predict fMRI brain activation patterns related to major depression.](https://neurosynth.org/studies/20630713/) | Abler B, Hofer C, Walter H, Erk S, Hoffmann H, Traue HC, Kessler H | Psychiatry research |
| 47 | [Healthy brooders employ more attentional resources when disengaging from the negative: an event-related fMRI study.](https://neurosynth.org/studies/21373973/) | Vanderhasselt MA, Kuhn S, De Raedt R | Cognitive, affective & behavioral neuroscience |
| 48 | [Hippocampal and Frontolimbic Function as Intermediate Phenotype for Psychosis: Evidence from Healthy Relatives and a Common Risk Variant in CACNA1C.](https://neurosynth.org/studies/24411473/) | Erk S, Meyer-Lindenberg A, Schmierer P, Mohnke S, Grimm O, Garbusow M, Haddad L, Poehland L, Muhleisen TW, Witt SH, Tost H, Kirsch P, Romanczuk-Seiferth N, Schott BH, Cichon S, Nothen MM, Rietschel M, Heinz A, Walter H | Biological psychiatry |
| 49 | [In search of the depressive self: extended medial prefrontal network during self-referential processing in major depression.](https://neurosynth.org/studies/19307251/) | Lemogne C, le Bastard G, Mayberg H, Volle E, Bergouignan L, Lehericy S, Allilaire JF, Fossati P | Social cognitive and affective neuroscience |
| 50 | [Increased neural activity during overt and continuous semantic verbal fluency in major depression: mainly a failure to deactivate.](https://neurosynth.org/studies/24557502/) | Backes H, Dietsche B, Nagels A, Stratmann M, Konrad C, Kircher T, Krug A | European archives of psychiatry and clinical neuroscience |
| 51 | [Inter-individual differences in the experience of negative emotion predict variations in functional brain architecture.](https://neurosynth.org/studies/26302674/) | Petrican R, Saverino C, Shayna Rosenbaum R, Grady C | NeuroImage |
| 52 | [Limbic and frontal cortical degeneration is associated with psychiatric symptoms in PINK1 mutation carriers.](https://neurosynth.org/studies/18261714/) | Reetz K, Lencer R, Steinlechner S, Gaser C, Hagenah J, Buchel C, Petersen D, Kock N, Djarmati A, Siebner HR, Klein C, Binkofski F | Biological psychiatry |
| 53 | [Low prefrontal perfusion linked to depression symptoms in methadone-maintained opiate-dependent patients.](https://neurosynth.org/studies/18674871/) | Suh JJ, Langleben DD, Ehrman RN, Hakun JG, Wang Z, Li Y, Busch SI, O'Brien CP, Childress AR | Drug and alcohol dependence |
| 54 | [Negative affectivity, self-referential processing and the cortical midline structures.](https://neurosynth.org/studies/20519253/) | Lemogne C, Gorwood P, Bergouignan L, Pelissolo A, Lehericy S, Fossati P | Social cognitive and affective neuroscience |
| 55 | [Neural correlates of depressive realism--an fMRI study on causal attribution in depression.](https://neurosynth.org/studies/22377511/) | Seidel EM, Satterthwaite TD, Eickhoff SB, Schneider F, Gur RC, Wolf DH, Habel U, Derntl B | Journal of affective disorders |
| 56 | [Neural correlates of idiographic goal priming in depression: goal-specific dysfunctions in the orbitofrontal cortex.](https://neurosynth.org/studies/19433416/) | Eddington KM, Dolcos F, McLean AN, Krishnan KR, Cabeza R, Strauman TJ | Social cognitive and affective neuroscience |
| 57 | [Neural correlates of self-perceptions in adolescents with major depressive disorder.](https://neurosynth.org/studies/26943454/) | Bradley KA, Colcombe S, Henderson SE, Alonso CM, Milham MP, Gabbay V | Developmental cognitive neuroscience |
| 58 | [Neural correlates of trauma script-imagery in posttraumatic stress disorder with and without comorbid major depression: a functional MRI investigation.](https://neurosynth.org/studies/17412567/) | Lanius RA, Frewen PA, Girotti M, Neufeld RW, Stevens TK, Densmore M | Psychiatry research |
| 59 | [Neural representation of reward in recovered depressed patients.](https://neurosynth.org/studies/19529923/) | McCabe C, Cowen PJ, Harmer CJ | Psychopharmacology |
| 60 | [Neural responses to sad facial expressions in major depression following cognitive behavioral therapy.](https://neurosynth.org/studies/18550030/) | Fu CH, Williams SC, Cleare AJ, Scott J, Mitterschiffthaler MT, Walsh ND, Donaldson C, Suckling J, Andrew C, Steiner H, Murray RM | Biological psychiatry |
| 61 | [Neural systems underlying thought suppression in young women with, and at-risk, for depression.](https://neurosynth.org/studies/24055881/) | Carew CL, Milne AM, Tatham EL, MacQueen GM, Hall GB | Behavioural brain research |
| 62 | [Prediction of antidepressant treatment response from gray matter volume across diagnostic categories.](https://neurosynth.org/studies/23920122/) | Samann PG, Hohn D, Chechko N, Kloiber S, Lucae S, Ising M, Holsboer F, Czisch M | European neuropsychopharmacology : the journal of the European College of Neuropsychopharmacology |
| 63 | [Prediction of SSRI treatment response in major depression based on serotonin transporter interplay between median raphe nucleus and projection areas.](https://neurosynth.org/studies/22828162/) | Lanzenberger R, Kranz GS, Haeusler D, Akimova E, Savli M, Hahn A, Mitterhauser M, Spindelegger C, Philippe C, Fink M, Wadsak W, Karanikas G, Kasper S | NeuroImage |
| 64 | [Prefrontal direct current stimulation modulates resting EEG and event-related potentials in healthy subjects: a standardized low resolution tomography (sLORETA) study.](https://neurosynth.org/studies/21146614/) | Keeser D, Padberg F, Reisinger E, Pogarell O, Kirsch V, Palm U, Karch S, Moller HJ, Nitsche MA, Mulert C | NeuroImage |
| 65 | [Prognostic prediction of therapeutic response in depression using high-field MR imaging.](https://neurosynth.org/studies/21134472/) | Gong Q, Wu Q, Scarpazza C, Lui S, Jia Z, Marquand A, Huang X, McGuire P, Mechelli A | NeuroImage |
| 66 | [Resting-state functional connectivity in major depression: abnormally increased contributions from subgenual cingulate cortex and thalamus.](https://neurosynth.org/studies/17210143/) | Greicius MD, Flores BH, Menon V, Glover GH, Solvason HB, Kenna H, Reiss AL, Schatzberg AF | Biological psychiatry |
| 67 | [Risk for affective disorders is associated with greater prefrontal gray matter volumes: A prospective longitudinal study.](https://neurosynth.org/studies/29527486/) | Macoveanu J, Baare W, Madsen KH, Kessing LV, Siebner HR, Vinberg M | NeuroImage. Clinical |
| 68 | [Sleep deprivation amplifies reactivity of brain reward networks, biasing the appraisal of positive emotional experiences.](https://neurosynth.org/studies/21430147/) | Gujar N, Yoo SS, Hu P, Walker MP | The Journal of neuroscience : the official journal of the Society for Neuroscience |
| 69 | [Striatal and cortical midline activation and connectivity associated with suicidal ideation and depression in bipolar II disorder.](https://neurosynth.org/studies/21621263/) | Marchand WR, Lee JN, Garn C, Thatcher J, Gale P, Kreitschitz S, Johnson S, Wood N | Journal of affective disorders |
| 70 | [The association of serotonin receptor 3A methylation with maternal violence exposure, neural activity, and child aggression.](https://neurosynth.org/studies/27720744/) | Schechter DS, Moser DA, Pointet VC, Aue T, Stenz L, Paoloni-Giacobino A, Adouan W, Manini A, Suardi F, Vital M, Sancho Rossignol A, Cordero MI, Rothenberg M, Ansermet F, Rusconi Serpa S, Dayer AG | Behavioural brain research |
| 71 | [The Danish PET/depression project: performance on Stroop's test linked to white matter lesions in the brain.](https://neurosynth.org/studies/15033182/) | Videbech P, Ravnkilde B, Gammelgaard L, Egander A, Clemmensen K, Rasmussen NA, Gjedde A, Rosenberg R | Psychiatry research |
| 72 | [The influence of positive and negative emotional associations on semantic processing in depression: an fMRI study.](https://neurosynth.org/studies/23033120/) | Sass K, Habel U, Kellermann T, Mathiak K, Gauggel S, Kircher T | Human brain mapping |
| 73 | [Toward a functional neuroanatomy of dysthymia: a functional magnetic resonance imaging study.](https://neurosynth.org/studies/19351572/) | Ravindran AV, Smith A, Cameron C, Bhatla R, Cameron I, Georgescu TM, Hogan MJ | Journal of affective disorders |
| 74 | [Unsupervised classification of major depression using functional connectivity MRI.](https://neurosynth.org/studies/23616377/) | Zeng LL, Shen H, Liu L, Hu D | Human brain mapping |
| 75 | [Vertex-wise examination of depressive symptom dimensions and brain volumes in older adults.](https://neurosynth.org/studies/28039796/) | McLaren ME, Szymkowicz SM, O'Shea A, Woods AJ, Anton SD, Dotson VM | Psychiatry research. Neuroimaging |
| 76 | [Whole brain resting-state analysis reveals decreased functional connectivity in major depression.](https://neurosynth.org/studies/20941370/) | Veer IM, Beckmann CF, van Tol MJ, Ferrarini L, Milles J, Veltman DJ, Aleman A, van Buchem MA, van der Wee NJ, Rombouts SA | Frontiers in systems neuroscience |
| 77 | [Why ruminators won't stop: the structural and resting state correlates of rumination and its relation to depression.](https://neurosynth.org/studies/22497878/) | Kuhn S, Vanderhasselt MA, De Raedt R, Gallinat J | Journal of affective disorders |

**Additional file 1: Table S7.** List of 106 studies extracted from Neurosynth under search term “PTSD” (post-traumatic stress disorder) on September 21, 2020

| **No.** | **Title** | **Authors** | **Journal** |
| --- | --- | --- | --- |
| 1 | [A Deletion Variant of the alpha2b-Adrenoceptor Modulates the Stress-Induced Shift from "Cognitive" to "Habit" Memory.](https://neurosynth.org/studies/28115477/) | Wirz L, Wacker J, Felten A, Reuter M, Schwabe L | The Journal of neuroscience : the official journal of the Society for Neuroscience |
| 2 | [A preliminary study of alterations in default network connectivity in post-traumatic stress disorder patients following recent trauma.](https://neurosynth.org/studies/23010311/) | Qin LD, Wang Z, Sun YW, Wan JQ, Su SS, Zhou Y, Xu JR | Brain research |
| 3 | [Abnormal recruitment of working memory updating networks during maintenance of trauma-neutral information in post-traumatic stress disorder.](https://neurosynth.org/studies/18455372/) | Moores KA, Clark CR, McFarlane AC, Brown GC, Puce A, Taylor DJ | Psychiatry research |
| 4 | [Adolescent earthquake survivors' show increased prefrontal cortex activation to masked earthquake images as adults.](https://neurosynth.org/studies/25486615/) | Du X, Wei D, Ganzel BL, Kim P, Zhang Q, Qiu J | International journal of psychophysiology : official journal of the International Organization of Psychophysiology |
| 5 | [Affect and neural activity in women with PTSD during a task of emotional interference.](https://neurosynth.org/studies/27318594/) | Brown WJ, Wojtalik JA, Dewey D, Bruce SE, Yang Z, Sheline YI | Journal of affective disorders |
| 6 | [Alpha oscillation neurofeedback modulates amygdala complex connectivity and arousal in posttraumatic stress disorder.](https://neurosynth.org/studies/27672554/) | Nicholson AA, Ros T, Frewen PA, Densmore M, Theberge J, Kluetsch RC, Jetly R, Lanius RA | NeuroImage. Clinical |
| 7 | [Alterations in Low-Level Perceptual Networks Related to Clinical Severity in PTSD after an Earthquake: A Resting-State fMRI Study.](https://neurosynth.org/studies/24823717/) | Shang J, Lui S, Meng Y, Zhu H, Qiu C, Gong Q, Liao W, Zhang W | PloS one |
| 8 | [Alterations in the cortical thickness and the amplitude of low-frequency fluctuation in patients with post-traumatic stress disorder.](https://neurosynth.org/studies/23122880/) | Bing X, Ming-Guo Q, Ye Z, Jing-Na Z, Min L, Han C, Yu Z, Jia-Jia Z, Jian W, Wei C, Han-Jian D, Shao-Xiang Z | Brain research |
| 9 | [Alterations in white matter microstructure as vulnerability factors and acquired signs of traffic accident-induced PTSD.](https://neurosynth.org/studies/24349515/) | Sun Y, Wang Z, Ding W, Wan J, Zhuang Z, Zhang Y, Liu Y, Zhou Y, Xu J | PloS one |
| 10 | [Altered functional connectivity in the brain default-mode network of earthquake survivors persists after 2 years despite recovery from anxiety symptoms.](https://neurosynth.org/studies/25862672/) | Du MY, Liao W, Lui S, Huang XQ, Li F, Kuang WH, Li J, Chen HF, Kendrick KM, Gong QY | Social cognitive and affective neuroscience |
| 11 | [Altered resting-state functional connectivity of thalamus in earthquake-induced posttraumatic stress disorder: a functional magnetic resonance imaging study.](https://neurosynth.org/studies/21813114/) | Yin Y, Jin C, Hu X, Duan L, Li Z, Song M, Chen H, Feng B, Jiang T, Jin H, Wong C, Gong Q, Li L | Brain research |
| 12 | [Altered reward processing in the nucleus accumbens and mesial prefrontal cortex of patients with posttraumatic stress disorder.](https://neurosynth.org/studies/18597797/) | Sailer U, Robinson S, Fischmeister FP, Konig D, Oppenauer C, Lueger-Schuster B, Moser E, Kryspin-Exner I, Bauer H | Neuropsychologia |
| 13 | [Altered spontaneous neuronal activity in chronic posttraumatic stress disorder patients before and after a 12-week paroxetine treatment.](https://neurosynth.org/studies/25527996/) | Zhu H, Qiu C, Meng Y, Cui H, Zhang Y, Huang X, Zhang J, Li T, Gong Q, Zhang W, Lui S | Journal of affective disorders |
| 14 | [Amygdala activity correlates with attentional bias in PTSD.](https://neurosynth.org/studies/21440563/) | El Khoury-Malhame M, Reynaud E, Soriano A, Michael K, Salgado-Pineda P, Zendjidjian X, Gellato C, Eric F, Lefebvre MN, Rouby F, Samuelian JC, Anton JL, Blin O, Khalfa S | Neuropsychologia |
| 15 | [An fMRI investigation of memory encoding in PTSD: influence of symptom severity.](https://neurosynth.org/studies/18321537/) | Dickie EW, Brunet A, Akerib V, Armony JL | Neuropsychologia |
| 16 | [An fMRI investigation of posttraumatic flashbacks.](https://neurosynth.org/studies/23207576/) | Whalley MG, Kroes MC, Huntley Z, Rugg MD, Davis SW, Brewin CR | Brain and cognition |
| 17 | [An fMRI study of anterior cingulate function in posttraumatic stress disorder.](https://neurosynth.org/studies/11750889/) | Shin LM, Whalen PJ, Pitman RK, Bush G, Macklin ML, Lasko NB, Orr SP, McInerney SC, Rauch SL | Biological psychiatry |
| 18 | [An fMRI study of the brain responses of traumatized mothers to viewing their toddlers during separation and play.](https://neurosynth.org/studies/22021653/) | Schechter DS, Moser DA, Wang Z, Marsh R, Hao X, Duan Y, Yu S, Gunter B, Murphy D, McCaw J, Kangarlu A, Willheim E, Myers MM, Hofer MA, Peterson BS | Social cognitive and affective neuroscience |
| 19 | [Avoidant symptoms in PTSD predict fear circuit activation during multimodal fear extinction.](https://neurosynth.org/studies/24146643/) | Sripada RK, Garfinkel SN, Liberzon I | Frontiers in human neuroscience |
| 20 | [BDNF Methylation and Maternal Brain Activity in a Violence-Related Sample.](https://neurosynth.org/studies/26649946/) | Moser DA, Paoloni-Giacobino A, Stenz L, Adouan W, Manini A, Suardi F, Cordero MI, Vital M, Sancho Rossignol A, Rusconi-Serpa S, Ansermet F, Dayer AG, Schechter DS | PloS one |
| 21 | [Behavioral and neural correlates of disrupted orienting attention in posttraumatic stress disorder.](https://neurosynth.org/studies/27966102/) | Russman Block S, King AP, Sripada RK, Weissman DH, Welsh R, Liberzon I | Cognitive, affective & behavioral neuroscience |
| 22 | [Brain activation during script-driven imagery induced dissociative responses in PTSD: a functional magnetic resonance imaging investigation.](https://neurosynth.org/studies/12208637/) | Lanius RA, Williamson PC, Boksman K, Densmore M, Gupta M, Neufeld RW, Gati JS, Menon RS | Biological psychiatry |
| 23 | [Brain functional connectivity correlates of coping styles.](https://neurosynth.org/studies/29572771/) | Santarnecchi E, Sprugnoli G, Tatti E, Mencarelli L, Neri F, Momi D, Di Lorenzo G, Pascual-Leone A, Rossi S, Rossi A | Cognitive, affective & behavioral neuroscience |
| 24 | [Brain responses to symptom provocation and trauma-related short-term memory recall in coal mining accident survivors with acute severe PTSD.](https://neurosynth.org/studies/17331476/) | Hou C, Liu J, Wang K, Li L, Liang M, He Z, Liu Y, Zhang Y, Li W, Jiang T | Brain research |
| 25 | [Cognitive reappraisal in trauma-exposed women with borderline personality disorder.](https://neurosynth.org/studies/21907809/) | Lang S, Kotchoubey B, Frick C, Spitzer C, Grabe HJ, Barnow S | NeuroImage |
| 26 | [Connectome-wide investigation of altered resting-state functional connectivity in war veterans with and without posttraumatic stress disorder.](https://neurosynth.org/studies/29527476/) | Misaki M, Phillips R, Zotev V, Wong CK, Wurfel BE, Krueger F, Feldner M, Bodurka J | NeuroImage. Clinical |
| 27 | [Converging evidence for abnormalities of the prefrontal cortex and evaluation of midsagittal structures in pediatric posttraumatic stress disorder: an MRI study.](https://neurosynth.org/studies/19349151/) | Carrion VG, Weems CF, Watson C, Eliez S, Menon V, Reiss AL | Psychiatry research |
| 28 | [Cortical thickness reduction in combat exposed U.S. veterans with and without PTSD.](https://neurosynth.org/studies/28279623/) | Wrocklage KM, Averill LA, Cobb Scott J, Averill CL, Schweinsburg B, Trejo M, Roy A, Weisser V, Kelly C, Martini B, Harpaz-Rotem I, Southwick SM, Krystal JH, Abdallah CG | European neuropsychopharmacology : the journal of the European College of Neuropsychopharmacology |
| 29 | [Cortical thinning in patients with recent onset post-traumatic stress disorder after a single prolonged trauma exposure.](https://neurosynth.org/studies/22720021/) | Liu Y, Li YJ, Luo EP, Lu HB, Yin H | PloS one |
| 30 | [Decreased gray matter volume in the left hippocampus and bilateral calcarine cortex in coal mine flood disaster survivors with recent onset PTSD.](https://neurosynth.org/studies/21498053/) | Zhang J, Tan Q, Yin H, Zhang X, Huan Y, Tang L, Wang H, Xu J, Li L | Psychiatry research |
| 31 | [Decreased premotor cortex volume in victims of urban violence with posttraumatic stress disorder.](https://neurosynth.org/studies/22952599/) | Rocha-Rego V, Pereira MG, Oliveira L, Mendlowicz MV, Fiszman A, Marques-Portella C, Berger W, Chu C, Joffily M, Moll J, Mari JJ, Figueira I, Volchan E | PloS one |
| 32 | [Deployment and post-deployment experiences in OEF/OIF veterans: relationship to gray matter volume.](https://neurosynth.org/studies/24058706/) | Aupperle RL, Connolly CG, Stillman AN, May AC, Paulus MP | PloS one |
| 33 | [Diagnosis of posttraumatic stress disorder (PTSD) based on correlations of prewhitened fMRI data: outcomes and areas involved.](https://neurosynth.org/studies/26070898/) | Christova P, James LM, Engdahl BE, Lewis SM, Georgopoulos AP | Experimental brain research |
| 34 | [Different regional gray matter loss in recent onset PTSD and non PTSD after a single prolonged trauma exposure.](https://neurosynth.org/studies/23155380/) | Chen Y, Fu K, Feng C, Tang L, Zhang J, Huan Y, Cui J, Mu Y, Qi S, Xiong L, Ma C, Wang H, Tan Q, Yin H | PloS one |
| 35 | [Different white matter abnormalities between the first-episode, treatment-naive patients with posttraumatic stress disorder and generalized anxiety disorder without comorbid conditions.](https://neurosynth.org/studies/21497403/) | Zhang L, Zhang Y, Li L, Li Z, Li W, Ma N, Hou C, Zhang Z, Zhang Z, Wang L, Duan L, Lu G | Journal of affective disorders |
| 36 | [Differential time courses and specificity of amygdala activity in posttraumatic stress disorder subjects and normal control subjects.](https://neurosynth.org/studies/15737660/) | Protopopescu X, Pan H, Tuescher O, Cloitre M, Goldstein M, Engelien W, Epstein J, Yang Y, Gorman J, LeDoux J, Silbersweig D, Stern E | Biological psychiatry |
| 37 | [Disruption of caudate working memory activation in chronic blast-related traumatic brain injury.](https://neurosynth.org/studies/26110112/) | Newsome MR, Durgerian S, Mourany L, Scheibel RS, Lowe MJ, Beall EB, Koenig KA, Parsons M, Troyanskaya M, Reece C, Wilde E, Fischer BL, Jones SE, Agarwal R, Levin HS, Rao SM | NeuroImage. Clinical |
| 38 | [Early altered resting-state functional connectivity predicts the severity of post-traumatic stress disorder symptoms in acutely traumatized subjects.](https://neurosynth.org/studies/23056477/) | Zhou Y, Wang Z, Qin LD, Wan JQ, Sun YW, Su SS, Ding WN, Xu JR | PloS one |
| 39 | [Effect of direct eye contact in PTSD related to interpersonal trauma: an fMRI study of activation of an innate alarm system.](https://neurosynth.org/studies/22977200/) | Steuwe C, Daniels JK, Frewen PA, Densmore M, Pannasch S, Beblo T, Reiss J, Lanius RA | Social cognitive and affective neuroscience |
| 40 | [Effect of direct eye contact in women with PTSD related to interpersonal trauma: Psychophysiological interaction analysis of connectivity of an innate alarm system.](https://neurosynth.org/studies/25862529/) | Steuwe C, Daniels JK, Frewen PA, Densmore M, Theberge J, Lanius RA | Psychiatry research |
| 41 | [Egocentric virtual maze learning in adult survivors of childhood abuse with dissociative disorders: evidence from functional magnetic resonance imaging.](https://neurosynth.org/studies/23522878/) | Weniger G, Siemerkus J, Barke A, Lange C, Ruhleder M, Sachsse U, Schmidt-Samoa C, Dechent P, Irle E | Psychiatry research |
| 42 | [Enhanced amygdala and medial prefrontal activation during nonconscious processing of fear in posttraumatic stress disorder: an fMRI study.](https://neurosynth.org/studies/17525984/) | Bryant RA, Kemp AH, Felmingham KL, Liddell B, Olivieri G, Peduto A, Gordon E, Williams LM | Human brain mapping |
| 43 | [Exaggerated and disconnected insular-amygdalar blood oxygenation level-dependent response to threat-related emotional faces in women with intimate-partner violence posttraumatic stress disorder.](https://neurosynth.org/studies/20573339/) | Fonzo GA, Simmons AN, Thorp SR, Norman SB, Paulus MP, Stein MB | Biological psychiatry |
| 44 | [Exposure to traumatic experiences is associated with abnormal neural mechanism during charitable donation.](https://neurosynth.org/studies/23920149/) | Wei D, Wang K, Shen Y, Du X, Li W, Dupuis-Roy N, Qiu J, Zhang Q | Psychiatry research |
| 45 | [Functional activation and neural networks in women with posttraumatic stress disorder related to intimate partner violence.](https://neurosynth.org/studies/18639236/) | Simmons AN, Paulus MP, Thorp SR, Matthews SC, Norman SB, Stein MB | Biological psychiatry |
| 46 | [Functional connectivity of dissociative responses in posttraumatic stress disorder: a functional magnetic resonance imaging investigation.](https://neurosynth.org/studies/15820708/) | Lanius RA, Williamson PC, Bluhm RL, Densmore M, Boksman K, Neufeld RW, Gati JS, Menon RS | Biological psychiatry |
| 47 | [Functional connectivity reveals inefficient working memory systems in post-traumatic stress disorder.](https://neurosynth.org/studies/19398308/) | Shaw ME, Moores KA, Clark RC, McFarlane AC, Strother SC, Bryant RA, Brown GC, Taylor JD | Psychiatry research |
| 48 | [Grey matter density changes of structures involved in Posttraumatic Stress Disorder (PTSD) after recovery following Eye Movement Desensitization and Reprocessing (EMDR) therapy.](https://neurosynth.org/studies/28667881/) | Boukezzi S, El Khoury-Malhame M, Auzias G, Reynaud E, Rousseau PF, Richard E, Zendjidjian X, Roques J, Castelli N, Correard N, Guyon V, Gellato C, Samuelian JC, Cancel A, Comte M, Latinus M, Guedj E, Khalfa S | Psychiatry research. Neuroimaging |
| 49 | [Impaired contextual modulation of memories in PTSD: an fMRI and psychophysiological study of extinction retention and fear renewal.](https://neurosynth.org/studies/25274821/) | Garfinkel SN, Abelson JL, King AP, Sripada RK, Wang X, Gaines LM, Liberzon I | The Journal of neuroscience : the official journal of the Society for Neuroscience |
| 50 | [Incidental retrieval of emotional contexts in post-traumatic stress disorder and depression: an fMRI study.](https://neurosynth.org/studies/18614265/) | Whalley MG, Rugg MD, Smith AP, Dolan RJ, Brewin CR | Brain and cognition |
| 51 | [Increased activation of the left hippocampus region in Complex PTSD during encoding and recognition of emotional words: a pilot study.](https://neurosynth.org/studies/19081708/) | Thomaes K, Dorrepaal E, Draijer NP, de Ruiter MB, Elzinga BM, van Balkom AJ, Smoor PL, Smit J, Veltman DJ | Psychiatry research |
| 52 | [Increased anterior cingulate cortex and hippocampus activation in Complex PTSD during encoding of negative words.](https://neurosynth.org/studies/22156722/) | Thomaes K, Dorrepaal E, Draijer N, de Ruiter MB, Elzinga BM, Sjoerds Z, van Balkom AJ, Smit JH, Veltman DJ | Social cognitive and affective neuroscience |
| 53 | [Increased recruitment of cognitive control in the presence of traumatic stimuli in complex PTSD.](https://neurosynth.org/studies/28712089/) | Herzog JI, Niedtfeld I, Rausch S, Thome J, Mueller-Engelmann M, Steil R, Priebe K, Bohus M, Schmahl C | European archives of psychiatry and clinical neuroscience |
| 54 | [Intranetwork and internetwork functional connectivity alterations in post-traumatic stress disorder.](https://neurosynth.org/studies/26331685/) | Zhang Y, Liu F, Chen H, Li M, Duan X, Xie B, Chen H | Journal of affective disorders |
| 55 | [Limbic scars: long-term consequences of childhood maltreatment revealed by functional and structural magnetic resonance imaging.](https://neurosynth.org/studies/22112927/) | Dannlowski U, Stuhrmann A, Beutelmann V, Zwanzger P, Lenzen T, Grotegerd D, Domschke K, Hohoff C, Ohrmann P, Bauer J, Lindner C, Postert C, Konrad C, Arolt V, Heindel W, Suslow T, Kugel H | Biological psychiatry |
| 56 | [Locus Coeruleus Activity Mediates Hyperresponsiveness in Posttraumatic Stress Disorder.](https://neurosynth.org/studies/29100627/) | Naegeli C, Zeffiro T, Piccirelli M, Jaillard A, Weilenmann A, Hassanpour K, Schick M, Rufer M, Orr SP, Mueller-Pfeiffer C | Biological psychiatry |
| 57 | [Long-Term Effects of Acute Stress on the Prefrontal-Limbic System in the Healthy Adult.](https://neurosynth.org/studies/28045980/) | Li Y, Hou X, Wei D, Du X, Zhang Q, Liu G, Qiu J | PloS one |
| 58 | [Maternal PTSD and corresponding neural activity mediate effects of child exposure to violence on child PTSD symptoms.](https://neurosynth.org/studies/28767657/) | Schechter DS, Moser DA, Aue T, Gex-Fabry M, Pointet VC, Cordero MI, Suardi F, Manini A, Vital M, Sancho Rossignol A, Rothenberg M, Dayer AG, Ansermet F, Rusconi Serpa S | PloS one |
| 59 | [Medial prefrontal cortex and right insula activity predict plasma ACTH response to trauma recall.](https://neurosynth.org/studies/19501653/) | King AP, Abelson JL, Britton JC, Phan KL, Taylor SF, Liberzon I | NeuroImage |
| 60 | [Negative emotion regulation in patients with posttraumatic stress disorder.](https://neurosynth.org/studies/24349161/) | Xiong K, Zhang Y, Qiu M, Zhang J, Sang L, Wang L, Xie B, Wang J, Li M | PloS one |
| 61 | [Negative emotional distraction on neural circuits for working memory in patients with posttraumatic stress disorder.](https://neurosynth.org/studies/23911835/) | Zhang JN, Xiong KL, Qiu MG, Zhang Y, Xie B, Wang J, Li M, Chen H, Zhang Y, Zhang JJ | Brain research |
| 62 | [Neural activity related to cognitive and emotional empathy in post-traumatic stress disorder.](https://neurosynth.org/studies/25555525/) | Mazza M, Tempesta D, Pino MC, Nigri A, Catalucci A, Guadagni V, Gallucci M, Iaria G, Ferrara M | Behavioural brain research |
| 63 | [Neural correlates of altered pain response in women with posttraumatic stress disorder from intimate partner violence.](https://neurosynth.org/studies/20553750/) | Strigo IA, Simmons AN, Matthews SC, Grimes EM, Allard CB, Reinhardt LE, Paulus MP, Stein MB | Biological psychiatry |
| 64 | [Neural correlates of attention bias to threat in post-traumatic stress disorder.](https://neurosynth.org/studies/22414937/) | Fani N, Jovanovic T, Ely TD, Bradley B, Gutman D, Tone EB, Ressler KJ | Biological psychology |
| 65 | [Neural correlates of trauma script-imagery in posttraumatic stress disorder with and without comorbid major depression: a functional MRI investigation.](https://neurosynth.org/studies/17412567/) | Lanius RA, Frewen PA, Girotti M, Neufeld RW, Stevens TK, Densmore M | Psychiatry research |
| 66 | [Neural differences underlying face processing in veterans with TBI and co-occurring TBI and PTSD.](https://neurosynth.org/studies/28753471/) | Bomyea J, Matthews SC, Buchsbaum MS, Spadoni AD, Strigo IA, Simmons AN | Journal of affective disorders |
| 67 | [Neural functional and structural correlates of childhood maltreatment in women with intimate-partner violence-related posttraumatic stress disorder.](https://neurosynth.org/studies/23154098/) | Fonzo GA, Flagan TM, Sullivan S, Allard CB, Grimes EM, Simmons AN, Paulus MP, Stein MB | Psychiatry research |
| 68 | [Neural networks of information processing in posttraumatic stress disorder: a functional magnetic resonance imaging study.](https://neurosynth.org/studies/16038681/) | Bryant RA, Felmingham KL, Kemp AH, Barton M, Peduto AS, Rennie C, Gordon E, Williams LM | Biological psychiatry |
| 69 | [Neural networks supporting autobiographical memory retrieval in posttraumatic stress disorder.](https://neurosynth.org/studies/23483523/) | St Jacques PL, Kragel PA, Rubin DC | Cognitive, affective & behavioral neuroscience |
| 70 | [Neural Response during the Activation of the Attachment System in Patients with Borderline Personality Disorder: An fMRI Study.](https://neurosynth.org/studies/27531977/) | Buchheim A, Erk S, George C, Kachele H, Martius P, Pokorny D, Spitzer M, Walter H | Frontiers in human neuroscience |
| 71 | [Neural responses during emotional processing before and after cognitive trauma therapy for battered women.](https://neurosynth.org/studies/23916537/) | Aupperle RL, Allard CB, Simmons AN, Flagan T, Thorp SR, Norman SB, Paulus MP, Stein MB | Psychiatry research |
| 72 | [Neural responses to auditory stimulus deviance under threat of electric shock revealed by spatially-filtered magnetoencephalography.](https://neurosynth.org/studies/17566766/) | Cornwell BR, Baas JM, Johnson L, Holroyd T, Carver FW, Lissek S, Grillon C | NeuroImage |
| 73 | [Neural systems for executive and emotional processing are modulated by symptoms of posttraumatic stress disorder in Iraq War veterans.](https://neurosynth.org/studies/18093809/) | Morey RA, Petty CM, Cooper DA, Labar KS, McCarthy G | Psychiatry research |
| 74 | [Neurobiological basis of failure to recall extinction memory in posttraumatic stress disorder.](https://neurosynth.org/studies/19748076/) | Milad MR, Pitman RK, Ellis CB, Gold AL, Shin LM, Lasko NB, Zeidan MA, Handwerger K, Orr SP, Rauch SL | Biological psychiatry |
| 75 | [Neurobiological indicators of disinhibition in posttraumatic stress disorder.](https://neurosynth.org/studies/25959594/) | Sadeh N, Spielberg JM, Miller MW, Milberg WP, Salat DH, Amick MM, Fortier CB, McGlinchey RE | Human brain mapping |
| 76 | [Noradrenergic enhancement of amygdala responses to fear.](https://neurosynth.org/studies/19246474/) | Onur OA, Walter H, Schlaepfer TE, Rehme AK, Schmidt C, Keysers C, Maier W, Hurlemann R | Social cognitive and affective neuroscience |
| 77 | [Parahippocampal activation evoked by masked traumatic images in posttraumatic stress disorder: a functional MRI study.](https://neurosynth.org/studies/15955491/) | Sakamoto H, Fukuda R, Okuaki T, Rogers M, Kasai K, Machida T, Shirouzu I, Yamasue H, Akiyama T, Kato N | NeuroImage |
| 78 | [Patterns of altered cortical perfusion and diminished subcortical integrity in posttraumatic stress disorder: an MRI study.](https://neurosynth.org/studies/20483375/) | Schuff N, Zhang Y, Zhan W, Lenoci M, Ching C, Boreta L, Mueller SG, Wang Z, Marmar CR, Weiner MW, Neylan TC | NeuroImage |
| 79 | [Perfusion Deficits and Functional Connectivity Alterations in Memory-Related Regions of Patients with Post-Traumatic Stress Disorder.](https://neurosynth.org/studies/27213610/) | Liu Y, Li B, Feng N, Pu H, Zhang X, Lu H, Yin H | PloS one |
| 80 | [PKCalpha is genetically linked to memory capacity in healthy subjects and to risk for posttraumatic stress disorder in genocide survivors.](https://neurosynth.org/studies/22586106/) | de Quervain DJ, Kolassa IT, Ackermann S, Aerni A, Boesiger P, Demougin P, Elbert T, Ertl V, Gschwind L, Hadziselimovic N, Hanser E, Heck A, Hieber P, Huynh KD, Klarhofer M, Luechinger R, Rasch B, Scheffler K, Spalek K, Stippich C, Vogler C, Vukojevic V, Stetak A, Papassotiropoulos A | Proceedings of the National Academy of Sciences of the United States of America |
| 81 | [Post-traumatic stress influences local and remote functional connectivity: a resting-state functional magnetic resonance imaging study.](https://neurosynth.org/studies/27722829/) | Ke J, Chen F, Qi R, Xu Q, Zhong Y, Chen L, Li J, Zhang L, Lu G | Brain imaging and behavior |
| 82 | [Post-traumatic stress symptoms correlate with smaller subgenual cingulate, caudate, and insula volumes in unmedicated combat veterans.](https://neurosynth.org/studies/23021615/) | Herringa R, Phillips M, Almeida J, Insana S, Germain A | Psychiatry research |
| 83 | [Posterior and prefrontal contributions to the development posttraumatic stress disorder symptom severity: an fMRI study of symptom provocation in acute stress disorder.](https://neurosynth.org/studies/27455992/) | Cwik JC, Sartory G, Nuyken M, Schurholt B, Seitz RJ | European archives of psychiatry and clinical neuroscience |
| 84 | [Posttraumatic stress disorder and fMRI activation patterns of traumatic memory in patients with borderline personality disorder.](https://neurosynth.org/studies/15013829/) | Driessen M, Beblo T, Mertens M, Piefke M, Rullkoetter N, Silva-Saavedra A, Reddemann L, Rau H, Markowitsch HJ, Wulff H, Lange W, Woermann FG | Biological psychiatry |
| 85 | [Precuneal and amygdala spontaneous activity and functional connectivity in war-zone-related PTSD.](https://neurosynth.org/studies/25561375/) | Yan X, Lazar M, Shalev AY, Neylan TC, Wolkowitz OM, Brown AD, Henn-Haase C, Yehuda R, Flory JD, Abu-Amara D, Sodickson DK, Marmar CR | Psychiatry research |
| 86 | [Prefrontal responses to digit span memory phases in patients with post-traumatic stress disorder (PTSD): a functional near infrared spectroscopy study.](https://neurosynth.org/studies/24936431/) | Tian F, Yennu A, Smith-Osborne A, Gonzalez-Lima F, North CS, Liu H | NeuroImage. Clinical |
| 87 | [Preliminary evidence for differential olfactory and trigeminal processing in combat veterans with and without PTSD.](https://neurosynth.org/studies/29159050/) | Cortese BM, Schumann AY, Howell AN, McConnell PA, Yang QX, Uhde TW | NeuroImage. Clinical |
| 88 | [PTSD symptom severity is associated with increased recruitment of top-down attentional control in a trauma-exposed sample.](https://neurosynth.org/studies/25610763/) | White SF, Costanzo ME, Blair JR, Roy MJ | NeuroImage. Clinical |
| 89 | [Quantifiable change in functional brain response to empathic and forgivability judgments with resolution of posttraumatic stress disorder.](https://neurosynth.org/studies/16213690/) | Farrow TF, Hunter MD, Wilkinson ID, Gouneea C, Fawbert D, Smith R, Lee KH, Mason S, Spence SA, Woodruff PW | Psychiatry research |
| 90 | [Recall of emotional states in posttraumatic stress disorder: an fMRI investigation.](https://neurosynth.org/studies/12559652/) | Lanius RA, Williamson PC, Hopper J, Densmore M, Boksman K, Gupta MA, Neufeld RW, Gati JS, Menon RS | Biological psychiatry |
| 91 | [Reduced amygdala and ventral striatal activity to happy faces in PTSD is associated with emotional numbing.](https://neurosynth.org/studies/25184336/) | Felmingham KL, Falconer EM, Williams L, Kemp AH, Allen A, Peduto A, Bryant RA | PloS one |
| 92 | [Reduced neural activation during an inhibition task is associated with impaired fear inhibition in a traumatized civilian sample.](https://neurosynth.org/studies/23020899/) | Jovanovic T, Ely T, Fani N, Glover EM, Gutman D, Tone EB, Norrholm SD, Bradley B, Ressler KJ | Cortex; a journal devoted to the study of the nervous system and behavior |
| 93 | [Regional cerebral changes and functional connectivity during the observation of negative emotional stimuli in subjects with post-traumatic stress disorder.](https://neurosynth.org/studies/23385487/) | Mazza M, Tempesta D, Pino MC, Catalucci A, Gallucci M, Ferrara M | European archives of psychiatry and clinical neuroscience |
| 94 | [Regional homogeneity and resting state functional connectivity: associations with exposure to early life stress.](https://neurosynth.org/studies/24090510/) | Philip NS, Kuras YI, Valentine TR, Sweet LH, Tyrka AR, Price LH, Carpenter LL | Psychiatry research |
| 95 | [Resting cerebral glucose metabolism and perfusion patterns in women with posttraumatic stress disorder related to sexual assault.](https://neurosynth.org/studies/22464826/) | Kim SY, Chung YK, Kim BS, Lee SJ, Yoon JK, An YS | Psychiatry research |
| 96 | [Resting state functional connectivity of the anterior cingulate cortex in veterans with and without post-traumatic stress disorder.](https://neurosynth.org/studies/25137414/) | Kennis M, Rademaker AR, van Rooij SJ, Kahn RS, Geuze E | Human brain mapping |
| 97 | [Sensory overload and imbalance: Resting-state vestibular connectivity in PTSD and its dissociative subtype.](https://neurosynth.org/studies/28911803/) | Harricharan S, Nicholson AA, Densmore M, Theberge J, McKinnon MC, Neufeld RWJ, Lanius RA | Neuropsychologia |
| 98 | [Stress, trauma and PTSD: translational insights into the core synaptic circuitry and its modulation.](https://neurosynth.org/studies/25985955/) | Bennett MR, Hatton SN, Lagopoulos J | Brain structure & function |
| 99 | [Stressed memories: how acute stress affects memory formation in humans.](https://neurosynth.org/studies/19675245/) | Henckens MJ, Hermans EJ, Pu Z, Joels M, Fernandez G | The Journal of neuroscience : the official journal of the Society for Neuroscience |
| 100 | [The association of serotonin receptor 3A methylation with maternal violence exposure, neural activity, and child aggression.](https://neurosynth.org/studies/27720744/) | Schechter DS, Moser DA, Pointet VC, Aue T, Stenz L, Paoloni-Giacobino A, Adouan W, Manini A, Suardi F, Vital M, Sancho Rossignol A, Cordero MI, Rothenberg M, Ansermet F, Rusconi Serpa S, Dayer AG | Behavioural brain research |
| 101 | [The effects of temporal unpredictability in anticipation of negative events in combat veterans with PTSD.](https://neurosynth.org/studies/22910447/) | Simmons AN, Flagan TM, Wittmann M, Strigo IA, Matthews SC, Donovan H, Lohr JB, Paulus MP | Journal of affective disorders |
| 102 | [The innate alarm circuit in post-traumatic stress disorder: Conscious and subconscious processing of fear- and trauma-related cues.](https://neurosynth.org/studies/26749205/) | Rabellino D, Densmore M, Frewen PA, Theberge J, Lanius RA | Psychiatry research. Neuroimaging |
| 103 | [Trauma modulates amygdala and medial prefrontal responses to consciously attended fear.](https://neurosynth.org/studies/16216534/) | Williams LM, Kemp AH, Felmingham K, Barton M, Olivieri G, Peduto A, Gordon E, Bryant RA | NeuroImage |
| 104 | [Unique insula subregion resting-state functional connectivity with amygdala complexes in posttraumatic stress disorder and its dissociative subtype.](https://neurosynth.org/studies/27042977/) | Nicholson AA, Sapru I, Densmore M, Frewen PA, Neufeld RW, Theberge J, McKinnon MC, Lanius RA | Psychiatry research. Neuroimaging |
| 105 | [Voxel-based morphometry in women with borderline personality disorder with and without comorbid posttraumatic stress disorder.](https://neurosynth.org/studies/23776553/) | Niedtfeld I, Schulze L, Krause-Utz A, Demirakca T, Bohus M, Schmahl C | PloS one |
| 106 | [White matter microstructural changes as vulnerability factors and acquired signs of post-earthquake distress.](https://neurosynth.org/studies/24400079/) | Sekiguchi A, Sugiura M, Taki Y, Kotozaki Y, Nouchi R, Takeuchi H, Araki T, Hanawa S, Nakagawa S, Miyauchi CM, Sakuma A, Kawashima R | PloS one |

**Additional file 1: Table S8.** List of 715 studies extracted from Neurosynth under search term “schizophrenia” on September 21, 2020

| **No.** | **Title** | **Authors** | **Journal** |
| --- | --- | --- | --- |
| 1 | [A 4.0-T fMRI study of brain connectivity during word fluency in first-episode schizophrenia.](https://neurosynth.org/studies/15885517/) | Boksman K, Theberge J, Williamson P, Drost DJ, Malla A, Densmore M, Takhar J, Pavlosky W, Menon RS, Neufeld RW | Schizophrenia research |
| 2 | [A behavioural and functional neuroimaging investigation into the effects of nicotine on sensorimotor gating in healthy subjects and persons with schizophrenia.](https://neurosynth.org/studies/16456657/) | Postma P, Gray JA, Sharma T, Geyer M, Mehrotra R, Das M, Zachariah E, Hines M, Williams SC, Kumari V | Psychopharmacology |
| 3 | [A brain-wide association study of DISC1 genetic variants reveals a relationship with the structure and functional connectivity of the precuneus in schizophrenia.](https://neurosynth.org/studies/24909300/) | Gong X, Lu W, Kendrick KM, Pu W, Wang C, Jin L, Lu G, Liu Z, Liu H, Feng J | Human brain mapping |
| 4 | [A CCA+ICA based model for multi-task brain imaging data fusion and its application to schizophrenia.](https://neurosynth.org/studies/20114081/) | Sui J, Adali T, Pearlson G, Yang H, Sponheim SR, White T, Calhoun VD | NeuroImage |
| 5 | [A computational morphometric MRI study of schizophrenia: effects of hallucinations.](https://neurosynth.org/studies/12427683/) | Shapleske J, Rossell SL, Chitnis XA, Suckling J, Simmons A, Bullmore ET, Woodruff PW, David AS | Cerebral cortex (New York, N.Y. : 1991) |
| 6 | [A Data-Driven Investigation of Gray Matter-Function Correlations in Schizophrenia during a Working Memory Task.](https://neurosynth.org/studies/21886614/) | Michael AM, King MD, Ehrlich S, Pearlson G, White T, Holt DJ, Andreasen NC, Sakoglu U, Ho BC, Schulz SC, Calhoun VD | Frontiers in human neuroscience |
| 7 | [A DTI study of white matter microstructure in individuals at high genetic risk for schizophrenia.](https://neurosynth.org/studies/18804959/) | Hoptman MJ, Nierenberg J, Bertisch HC, Catalano D, Ardekani BA, Branch CA, Delisi LE | Schizophrenia research |
| 8 | [A functional magnetic resonance imaging study of working memory abnormalities in schizophrenia.](https://neurosynth.org/studies/16503328/) | Johnson MR, Morris NA, Astur RS, Calhoun VD, Mathalon DH, Kiehl KA, Pearlson GD | Biological psychiatry |
| 9 | [A genome-wide supported variant in CACNA1C influences hippocampal activation during episodic memory encoding and retrieval.](https://neurosynth.org/studies/23860750/) | Krug A, Witt SH, Backes H, Dietsche B, Nieratschker V, Shah NJ, Nothen MM, Rietschel M, Kircher T | European archives of psychiatry and clinical neuroscience |
| 10 | [A group ICA based framework for evaluating resting fMRI markers when disease categories are unclear: application to schizophrenia, bipolar, and schizoaffective disorders.](https://neurosynth.org/studies/26216278/) | Du Y, Pearlson GD, Liu J, Sui J, Yu Q, He H, Castro E, Calhoun VD | NeuroImage |
| 11 | [A hemodynamic model for layered BOLD signals.](https://neurosynth.org/studies/26484827/) | Heinzle J, Koopmans PJ, den Ouden HEM, Raman S, Stephan KE | NeuroImage |
| 12 | [A hypomethylating variant of MTHFR, 677C>T, blunts the neural response to errors in patients with schizophrenia and healthy individuals.](https://neurosynth.org/studies/21980405/) | Roffman JL, Nitenson AZ, Agam Y, Isom M, Friedman JS, Dyckman KA, Brohawn DG, Smoller JW, Goff DC, Manoach DS | PloS one |
| 13 | [A longitudinal study on intrinsic connectivity of hippocampus associated with positive symptom in first-episode schizophrenia.](https://neurosynth.org/studies/25619684/) | Duan HF, Gan JL, Yang JM, Cheng ZX, Gao CY, Shi ZJ, Zhu XQ, Liang XJ, Zhao LM | Behavioural brain research |
| 14 | [A manual and automated MRI study of anterior cingulate and orbito-frontal cortices, and caudate nucleus in obsessive-compulsive disorder: comparison with healthy controls and patients with schizophren](https://neurosynth.org/studies/15766634/) | Riffkin J, Yucel M, Maruff P, Wood SJ, Soulsby B, Olver J, Kyrios M, Velakoulis D, Pantelis C | Psychiatry research |
| 15 | [A method for functional network connectivity among spatially independent resting-state components in schizophrenia.](https://neurosynth.org/studies/18082428/) | Jafri MJ, Pearlson GD, Stevens M, Calhoun VD | NeuroImage |
| 16 | [A method for multi-group inter-participant correlation: abnormal synchrony in patients with schizophrenia during auditory target detection.](https://neurosynth.org/studies/17996465/) | Kim D, Pearlson GD, Kiehl KA, Bedrick E, Demirci O, Calhoun VD | NeuroImage |
| 17 | [A method for multitask fMRI data fusion applied to schizophrenia.](https://neurosynth.org/studies/16342150/) | Calhoun VD, Adali T, Kiehl KA, Astur R, Pekar JJ, Pearlson GD | Human brain mapping |
| 18 | [A multi-site resting state fMRI study on the amplitude of low frequency fluctuations in schizophrenia.](https://neurosynth.org/studies/23964193/) | Turner JA, Damaraju E, van Erp TG, Mathalon DH, Ford JM, Voyvodic J, Mueller BA, Belger A, Bustillo J, McEwen S, Potkin SG, Fbirn, Calhoun VD | Frontiers in neuroscience |
| 19 | [A neuroimaging study of emotion-cognition interaction in schizophrenia: the effect of ziprasidone treatment.](https://neurosynth.org/studies/28210783/) | Stip E, Cherbal A, Luck D, Zhornitsky S, Bentaleb LA, Lungu O | Psychopharmacology |
| 20 | [A resting-state functional magnetic resonance imaging study on the first-degree relatives of persons with schizophrenia.](https://neurosynth.org/studies/22370913/) | Liao H, Wang L, Zhou B, Tang J, Tan L, Zhu X, Yi J, Chen X, Tan C | Brain imaging and behavior |
| 21 | [A reversal of the normal pattern of parahippocampal response to neutral and fearful faces is associated with reality distortion in schizophrenia.](https://neurosynth.org/studies/16487943/) | Surguladze S, Russell T, Kucharska-Pietura K, Travis MJ, Giampietro V, David AS, Phillips ML | Biological psychiatry |
| 22 | [A systematic fMRI investigation of the brain systems subserving different working memory components in schizophrenia.](https://neurosynth.org/studies/19686473/) | Henseler I, Falkai P, Gruber O | The European journal of neuroscience |
| 23 | [A systematic review of associations between functional MRI activity and polygenic risk for schizophrenia and bipolar disorder.](https://neurosynth.org/studies/29748770/) | Dezhina Z, Ranlund S, Kyriakopoulos M, Williams SCR, Dima D | Brain imaging and behavior |
| 24 | [A visual joke fMRI investigation into Theory of Mind and enhanced risk of schizophrenia.](https://neurosynth.org/studies/16624578/) | Marjoram D, Job DE, Whalley HC, Gountouna VE, McIntosh AM, Simonotto E, Cunningham-Owens D, Johnstone EC, Lawrie S | NeuroImage |
| 25 | [Aberrant activity and connectivity of the posterior superior temporal sulcus during social cognition in schizophrenia.](https://neurosynth.org/studies/27770284/) | Mier D, Eisenacher S, Rausch F, Englisch S, Gerchen MF, Zamoscik V, Meyer-Lindenberg A, Zink M, Kirsch P | European archives of psychiatry and clinical neuroscience |
| 26 | [Aberrant localization of synchronous hemodynamic activity in auditory cortex reliably characterizes schizophrenia.](https://neurosynth.org/studies/15050866/) | Calhoun VD, Kiehl KA, Liddle PF, Pearlson GD | Biological psychiatry |
| 27 | [Aberrant salience network (bilateral insula and anterior cingulate cortex) connectivity during information processing in schizophrenia.](https://neurosynth.org/studies/20724114/) | White TP, Joseph V, Francis ST, Liddle PF | Schizophrenia research |
| 28 | [Aberrant spontaneous neural activity and correlation with evoked-brain potentials in first-episode, treatment-naive patients with deficit and non-deficit schizophrenia.](https://neurosynth.org/studies/28092779/) | Li Z, Lei W, Deng W, Zheng Z, Li M, Ma X, Wang Q, Huang C, Li N, Collier DA, Gong Q, Li T | Psychiatry research. Neuroimaging |
| 29 | [Aberrant visual circuitry associated with normal spatial match-to-sample accuracy in schizophrenia.](https://neurosynth.org/studies/21782395/) | Avsar KB, Stoeckel LE, Bolding MS, White DM, Tagamets MA, Holcomb HH, Lahti AC | Psychiatry research |
| 30 | [Abnormal auditory sensory gating-out in first-episode and never-medicated paranoid schizophrenia patients: an fMRI study.](https://neurosynth.org/studies/23820976/) | Ji B, Mei W, Zhang JX, Jing J, Wu Q, Zhuo Y, Xiao Z | Experimental brain research |
| 31 | [Abnormal brain response during the auditory emotional processing in schizophrenic patients with chronic auditory hallucinations.](https://neurosynth.org/studies/18818053/) | Kang JI, Kim JJ, Seok JH, Chun JW, Lee SK, Park HJ | Schizophrenia research |
| 32 | [Abnormal connectivity between attentional, language and auditory networks in schizophrenia.](https://neurosynth.org/studies/22226903/) | Liemburg EJ, Vercammen A, Ter Horst GJ, Curcic-Blake B, Knegtering H, Aleman A | Schizophrenia research |
| 33 | [Abnormal cortical activation during response inhibition in 22q11.2 deletion syndrome.](https://neurosynth.org/studies/17427209/) | Gothelf D, Hoeft F, Hinard C, Hallmayer JF, Stoecker JV, Antonarakis SE, Morris MA, Reiss AL | Human brain mapping |
| 34 | [Abnormal functional motor lateralization in healthy siblings of patients with schizophrenia.](https://neurosynth.org/studies/22901767/) | Altamura M, Fazio L, De Salvia M, Petito A, Blasi G, Taurisano P, Romano R, Gelao B, Bellomo A, Bertolino A | Psychiatry research |
| 35 | [Abnormal neural activity as a potential biomarker for drug-naive first-episode adolescent-onset schizophrenia with coherence regional homogeneity and support vector machine analyses.](https://neurosynth.org/studies/28476336/) | Liu Y, Zhang Y, Lv L, Wu R, Zhao J, Guo W | Schizophrenia research |
| 36 | [Abnormal neural hierarchy in processing of verbal information in patients with schizophrenia.](https://neurosynth.org/studies/29349038/) | Lerner Y, Bleich-Cohen M, Solnik-Knirsh S, Yogev-Seligmann G, Eisenstein T, Madah W, Shamir A, Hendler T, Kremer I | NeuroImage. Clinical |
| 37 | [Abnormal neural processing during emotional salience attribution of affective asymmetry in patients with schizophrenia.](https://neurosynth.org/studies/24619004/) | Lee SK, Chun JW, Lee JS, Park HJ, Jung YC, Seok JH, Kim JJ | PloS one |
| 38 | [Abnormal neural responses to social exclusion in schizophrenia.](https://neurosynth.org/studies/22916139/) | Gradin VB, Waiter G, Kumar P, Stickle C, Milders M, Matthews K, Reid I, Hall J, Steele JD | PloS one |
| 39 | [Abnormal object recall and anterior cingulate overactivation correlate with formal thought disorder in schizophrenia.](https://neurosynth.org/studies/16199012/) | Assaf M, Rivkin PR, Kuzu CH, Calhoun VD, Kraut MA, Groth KM, Yassa MA, Hart J Jr, Pearlson GD | Biological psychiatry |
| 40 | [Abnormal prefrontal cortical activity and connectivity during response selection in first episode psychosis, chronic schizophrenia, and unaffected siblings of individuals with schizophrenia.](https://neurosynth.org/studies/19179050/) | Woodward ND, Waldie B, Rogers B, Tibbo P, Seres P, Purdon SE | Schizophrenia research |
| 41 | [Abnormal processing of speech during oddball target detection in schizophrenia.](https://neurosynth.org/studies/14568459/) | Ngan ET, Vouloumanos A, Cairo TA, Laurens KR, Bates AT, Anderson CM, Werker JF, Liddle PF | NeuroImage |
| 42 | [Abnormal regional homogeneity as a potential imaging biomarker for adolescent-onset schizophrenia: A resting-state fMRI study and support vector machine analysis.](https://neurosynth.org/studies/28587813/) | Wang S, Zhang Y, Lv L, Wu R, Fan X, Zhao J, Guo W | Schizophrenia research |
| 43 | [Abnormal temporal difference reward-learning signals in major depression.](https://neurosynth.org/studies/18579575/) | Kumar P, Waiter G, Ahearn T, Milders M, Reid I, Steele JD | Brain : a journal of neurology |
| 44 | [Abnormalities in large scale functional networks in unmedicated patients with schizophrenia and effects of risperidone.](https://neurosynth.org/studies/26793436/) | Kraguljac NV, White DM, Hadley JA, Visscher K, Knight D, ver Hoef L, Falola B, Lahti AC | NeuroImage. Clinical |
| 45 | [Abnormalities in personal space and parietal-frontal function in schizophrenia.](https://neurosynth.org/studies/26484048/) | Holt DJ, Boeke EA, Coombs G 3rd, DeCross SN, Cassidy BS, Stufflebeam S, Rauch SL, Tootell RB | NeuroImage. Clinical |
| 46 | [Abnormalities of regional homogeneity and its correlation with clinical symptoms in Naive patients with first-episode schizophrenia.](https://neurosynth.org/studies/29736883/) | Zhao X, Yao J, Lv Y, Zhang X, Han C, Chen L, Ren F, Jin Z, Li Y, Sui Y | Brain imaging and behavior |
| 47 | [Action simulation in hallucination-prone adolescents.](https://neurosynth.org/studies/23847502/) | Dahoun T, Eliez S, Chen F, Badoud D, Schneider M, Laroi F, Debbane M | Frontiers in human neuroscience |
| 48 | [Activation and Functional Connectivity of the Left Inferior Temporal Gyrus during Visual Speech Priming in Healthy Listeners and Listeners with Schizophrenia.](https://neurosynth.org/studies/28360829/) | Wu C, Zheng Y, Li J, Zhang B, Li R, Wu H, She S, Liu S, Peng H, Ning Y, Li L | Frontiers in neuroscience |
| 49 | [Activation of midbrain and ventral striatal regions implicates salience processing during a modified beads task.](https://neurosynth.org/studies/23484034/) | Esslinger C, Braun U, Schirmbeck F, Santos A, Meyer-Lindenberg A, Zink M, Kirsch P | PloS one |
| 50 | [Acute effects of single-dose aripiprazole and haloperidol on resting cerebral blood flow (rCBF) in the human brain.](https://neurosynth.org/studies/22451196/) | Handley R, Zelaya FO, Reinders AA, Marques TR, Mehta MA, O'Gorman R, Alsop DC, Taylor H, Johnston A, Williams S, McGuire P, Pariante CM, Kapur S, Dazzan P | Human brain mapping |
| 51 | [Adolescent resting state networks and their associations with schizotypal trait expression.](https://neurosynth.org/studies/20844603/) | Lagioia A, Van De Ville D, Debbane M, Lazeyras F, Eliez S | Frontiers in systems neuroscience |
| 52 | [Alexithymia and regional gray matter alterations in schizophrenia.](https://neurosynth.org/studies/21300113/) | Kubota M, Miyata J, Hirao K, Fujiwara H, Kawada R, Fujimoto S, Tanaka Y, Sasamoto A, Sawamoto N, Fukuyama H, Takahashi H, Murai T | Neuroscience research |
| 53 | [Allelic variation in RGS4 impacts functional and structural connectivity in the human brain.](https://neurosynth.org/studies/17301167/) | Buckholtz JW, Meyer-Lindenberg A, Honea RA, Straub RE, Pezawas L, Egan MF, Vakkalanka R, Kolachana B, Verchinski BA, Sust S, Mattay VS, Weinberger DR, Callicott JH | The Journal of neuroscience : the official journal of the Society for Neuroscience |
| 54 | [Alterations in functional activation in euthymic bipolar disorder and schizophrenia during a working memory task.](https://neurosynth.org/studies/19449330/) | Hamilton LS, Altshuler LL, Townsend J, Bookheimer SY, Phillips OR, Fischer J, Woods RP, Mazziotta JC, Toga AW, Nuechterlein KH, Narr KL | Human brain mapping |
| 55 | [Alterations in regional homogeneity of resting-state brain activity in autism spectrum disorders.](https://neurosynth.org/studies/20053346/) | Paakki JJ, Rahko J, Long X, Moilanen I, Tervonen O, Nikkinen J, Starck T, Remes J, Hurtig T, Haapsamo H, Jussila K, Kuusikko-Gauffin S, Mattila ML, Zang Y, Kiviniemi V | Brain research |
| 56 | [Alterations in regional homogeneity of resting-state brain activity in ketamine addicts.](https://neurosynth.org/studies/22698584/) | Liao Y, Tang J, Fornito A, Liu T, Chen X, Chen H, Xiang X, Wang X, Hao W | Neuroscience letters |
| 57 | [Alterations of theory of mind network activation in chronic cannabis users.](https://neurosynth.org/studies/22695256/) | Roser P, Lissek S, Tegenthoff M, Nicolas V, Juckel G, Brune M | Schizophrenia research |
| 58 | [Altered activation and functional connectivity of neural systems supporting cognitive control of emotion in psychosis proneness.](https://neurosynth.org/studies/20188516/) | Modinos G, Ormel J, Aleman A | Schizophrenia research |
| 59 | [Altered activation in association with reward-related trial-and-error learning in patients with schizophrenia.](https://neurosynth.org/studies/20006717/) | Koch K, Schachtzabel C, Wagner G, Schikora J, Schultz C, Reichenbach JR, Sauer H, Schlosser RG | NeuroImage |
| 60 | [Altered balance of functional brain networks in Schizophrenia.](https://neurosynth.org/studies/26786152/) | Woodward TS, Leong K, Sanford N, Tipper CM, Lavigne KM | Psychiatry research. Neuroimaging |
| 61 | [Altered Basal Ganglia Network Integration in Schizophrenia.](https://neurosynth.org/studies/26528167/) | Duan M, Chen X, He H, Jiang Y, Jiang S, Xie Q, Lai Y, Luo C, Yao D | Frontiers in human neuroscience |
| 62 | [Altered brain activation in dorsolateral prefrontal cortex in adolescents and young adults at genetic risk for schizophrenia: an fMRI study of working memory.](https://neurosynth.org/studies/16632333/) | Seidman LJ, Thermenos HW, Poldrack RA, Peace NK, Koch JK, Faraone SV, Tsuang MT | Schizophrenia research |
| 63 | [Altered default mode and fronto-parietal network subsystems in patients with schizophrenia and their unaffected siblings.](https://neurosynth.org/studies/24675026/) | Chang X, Shen H, Wang L, Liu Z, Xin W, Hu D, Miao D | Brain research |
| 64 | [Altered default mode network functional connectivity in schizotypal personality disorder.](https://neurosynth.org/studies/25458858/) | Zhang Q, Shen J, Wu J, Yu X, Lou W, Fan H, Shi L, Wang D | Schizophrenia research |
| 65 | [Altered engagement of attention and default networks during target detection in schizophrenia.](https://neurosynth.org/studies/20869846/) | Hasenkamp W, James GA, Boshoven W, Duncan E | Schizophrenia research |
| 66 | [Altered error-related activity in patients with schizophrenia.](https://neurosynth.org/studies/19540863/) | Koch K, Wagner G, Schultz C, Schachtzabel C, Nenadic I, Axer M, Reichenbach JR, Sauer H, Schlosser RG | Neuropsychologia |
| 67 | [Altered fronto-limbic activity in children and adolescents with familial high risk for schizophrenia.](https://neurosynth.org/studies/23482245/) | Hart SJ, Bizzell J, McMahon MA, Gu H, Perkins DO, Belger A | Psychiatry research |
| 68 | [Altered functional connectivity during self- and close other-reflection in patients with bipolar disorder with past psychosis and patients with schizophrenia.](https://neurosynth.org/studies/27693668/) | Zhang L, Vander Meer L, Opmeer EM, Marsman JC, Ruhe HG, Aleman A | Neuropsychologia |
| 69 | [Altered Hippocampo-Cerebello-Cortical Circuit in Schizophrenia by a Spatiotemporal Consistency and Causal Connectivity Analysis.](https://neurosynth.org/studies/28194095/) | Chen X, Jiang Y, Chen L, He H, Dong L, Hou C, Duan M, Yang M, Yao D, Luo C | Frontiers in neuroscience |
| 70 | [Altered intrinsic and extrinsic connectivity in schizophrenia.](https://neurosynth.org/studies/29264112/) | Zhou Y, Zeidman P, Wu S, Razi A, Chen C, Yang L, Zou J, Wang G, Wang H, Friston KJ | NeuroImage. Clinical |
| 71 | [Altered medial prefrontal activity during dynamic face processing in schizophrenia spectrum patients.](https://neurosynth.org/studies/24888525/) | Mothersill O, Morris DW, Kelly S, Rose EJ, Bokde A, Reilly R, Gill M, Corvin AP, Donohoe G | Schizophrenia research |
| 72 | [Altered neural basis of the reality processing and its relation to cognitive insight in schizophrenia.](https://neurosynth.org/studies/25793291/) | Lee JS, Chun JW, Lee SH, Kim E, Lee SK, Kim JJ | PloS one |
| 73 | [Altered relationships between age and functional brain activation in adolescents at clinical high risk for psychosis.](https://neurosynth.org/studies/24144510/) | Karlsgodt KH, van Erp TG, Bearden CE, Cannon TD | Psychiatry research |
| 74 | [Altered resting-state functional connectivity and anatomical connectivity of hippocampus in schizophrenia.](https://neurosynth.org/studies/18234476/) | Zhou Y, Shu N, Liu Y, Song M, Hao Y, Liu H, Yu C, Liu Z, Jiang T | Schizophrenia research |
| 75 | [Altered structural connectivity and trait anhedonia in patients with schizophrenia.](https://neurosynth.org/studies/25017826/) | Lee JS, Han K, Lee SK, Seok JH, Kim JJ | Neuroscience letters |
| 76 | [Altered threat and safety neural processing linked to persecutory delusions in schizophrenia: a two-task fMRI study.](https://neurosynth.org/studies/26208746/) | Perez DL, Pan H, Weisholtz DS, Root JC, Tuescher O, Fischer DB, Butler T, Vago DR, Isenberg N, Epstein J, Landa Y, Smith TE, Savitz AJ, Silbersweig DA, Stern E | Psychiatry research |
| 77 | [Altered velocity processing in schizophrenia during pursuit eye tracking.](https://neurosynth.org/studies/22693639/) | Nagel M, Sprenger A, Steinlechner S, Binkofski F, Lencer R | PloS one |
| 78 | [Altered Volume and Functional Connectivity of the Habenula in Schizophrenia.](https://neurosynth.org/studies/29311883/) | Zhang L, Wang H, Luan S, Yang S, Wang Z, Wang J, Zhao H | Frontiers in human neuroscience |
| 79 | [Altered volume and lateralization of language-related regions in first-episode schizophrenia.](https://neurosynth.org/studies/23769260/) | Sheng J, Zhu Y, Lu Z, Liu N, Huang N, Zhang Z, Tan L, Li C, Yu X | Schizophrenia research |
| 80 | [Ambiguity aversion in schizophrenia: An fMRI study of decision-making under risk and ambiguity.](https://neurosynth.org/studies/27623361/) | Fujino J, Hirose K, Tei S, Kawada R, Tsurumi K, Matsukawa N, Miyata J, Sugihara G, Yoshihara Y, Ideno T, Aso T, Takemura K, Fukuyama H, Murai T, Takahashi H | Schizophrenia research |
| 81 | [Amplitude of low-frequency oscillations in schizophrenia: a resting state fMRI study.](https://neurosynth.org/studies/19854028/) | Hoptman MJ, Zuo XN, Butler PD, Javitt DC, D'Angelo D, Mauro CJ, Milham MP | Schizophrenia research |
| 82 | [An anterior-to-posterior shift in midline cortical activity in schizophrenia during self-reflection.](https://neurosynth.org/studies/21144498/) | Holt DJ, Cassidy BS, Andrews-Hanna JR, Lee SM, Coombs G, Goff DC, Gabrieli JD, Moran JM | Biological psychiatry |
| 83 | [An event-related FMRI study of phonological verbal working memory in schizophrenia.](https://neurosynth.org/studies/20725639/) | Kim J, Matthews NL, Park S | PloS one |
| 84 | [An event-related functional magnetic resonance imaging study of an auditory oddball task in schizophrenia.](https://neurosynth.org/studies/11295369/) | Kiehl KA, Liddle PF | Schizophrenia research |
| 85 | [An evoked auditory response fMRI study of the effects of rTMS on putative AVH pathways in healthy volunteers.](https://neurosynth.org/studies/19769994/) | Tracy DK, O'Daly O, Joyce DW, Michalopoulou PG, Basit BB, Dhillon G, McLoughlin DM, Shergill SS | Neuropsychologia |
| 86 | [An fMRI investigation of a novel analogue to the Trail-Making Test.](https://neurosynth.org/studies/21782309/) | Jacobson SC, Blanchard M, Connolly CC, Cannon M, Garavan H | Brain and cognition |
| 87 | [An fMRI investigation of delay discounting in patients with schizophrenia.](https://neurosynth.org/studies/24381810/) | Avsar KB, Weller RE, Cox JE, Reid MA, White DM, Lahti AC | Brain and behavior |
| 88 | [An fMRI investigation of procedural learning in unaffected siblings of individuals with schizophrenia.](https://neurosynth.org/studies/17544630/) | Woodward ND, Tibbo P, Purdon SE | Schizophrenia research |
| 89 | [An fMRI study of "theory of mind" in at-risk states of psychosis: comparison with manifest schizophrenia and healthy controls.](https://neurosynth.org/studies/21147235/) | Brune M, Ozgurdal S, Ansorge N, von Reventlow HG, Peters S, Nicolas V, Tegenthoff M, Juckel G, Lissek S | NeuroImage |
| 90 | [An fMRI study of auditory hallucinations in patients with epilepsy.](https://neurosynth.org/studies/19817808/) | Korsnes MS, Hugdahl K, Nygard M, Bjornaes H | Epilepsia |
| 91 | [An fMRI study of differential neural response to affective pictures in schizophrenia.](https://neurosynth.org/studies/15219596/) | Takahashi H, Koeda M, Oda K, Matsuda T, Matsushima E, Matsuura M, Asai K, Okubo Y | NeuroImage |
| 92 | [An fMRI study of functional abnormalities in the verbal working memory system and the relationship to clinical symptoms in chronic schizophrenia.](https://neurosynth.org/studies/19395526/) | Hashimoto R, Lee K, Preus A, McCarley RW, Wible CG | Cerebral cortex (New York, N.Y. : 1991) |
| 93 | [An fMRI study of reduced left prefrontal activation in schizophrenia during normal inhibitory function.](https://neurosynth.org/studies/11595391/) | Rubia K, Russell T, Bullmore ET, Soni W, Brammer MJ, Simmons A, Taylor E, Andrew C, Giampietro V, Sharma T | Schizophrenia research |
| 94 | [An fMRI study of theory of mind in schizophrenic patients with "passivity" symptoms.](https://neurosynth.org/studies/18329671/) | Brune M, Lissek S, Fuchs N, Witthaus H, Peters S, Nicolas V, Juckel G, Tegenthoff M | Neuropsychologia |
| 95 | [An fMRI study of visual attention and sensorimotor function before and after antipsychotic treatment in first-episode schizophrenia.](https://neurosynth.org/studies/19243925/) | Keedy SK, Rosen C, Khine T, Rajarethinam R, Janicak PG, Sweeney JA | Psychiatry research |
| 96 | [An fMRI study of visual lexical decision in patients with schizophrenia and clinical high-risk individuals.](https://neurosynth.org/studies/24893907/) | Natsubori T, Hashimoto R, Yahata N, Inoue H, Takano Y, Iwashiro N, Koike S, Gonoi W, Sasaki H, Takao H, Abe O, Kasai K, Yamasue H | Schizophrenia research |
| 97 | [An fMRI study of working memory in first-degree unaffected relatives of schizophrenia patients.](https://neurosynth.org/studies/18678469/) | Meda SA, Bhattarai M, Morris NA, Astur RS, Calhoun VD, Mathalon DH, Kiehl KA, Pearlson GD | Schizophrenia research |
| 98 | [An ICA-based method for the identification of optimal FMRI features and components using combined group-discriminative techniques.](https://neurosynth.org/studies/19457398/) | Sui J, Adali T, Pearlson GD, Calhoun VD | NeuroImage |
| 99 | [Anatomically related grey and white matter abnormalities in adolescent-onset schizophrenia.](https://neurosynth.org/studies/17698497/) | Douaud G, Smith S, Jenkinson M, Behrens T, Johansen-Berg H, Vickers J, James S, Voets N, Watkins K, Matthews PM, James A | Brain : a journal of neurology |
| 100 | [Anhedonia and emotional experience in schizophrenia: neural and behavioral indicators.](https://neurosynth.org/studies/20004364/) | Dowd EC, Barch DM | Biological psychiatry |
| 101 | [Anomalous neural circuit function in schizophrenia during a virtual Morris water task.](https://neurosynth.org/studies/19948225/) | Folley BS, Astur R, Jagannathan K, Calhoun VD, Pearlson GD | NeuroImage |
| 102 | [Assessment of white matter abnormalities in paranoid schizophrenia and bipolar mania patients.](https://neurosynth.org/studies/22079662/) | Cui L, Chen Z, Deng W, Huang X, Li M, Ma X, Huang C, Jiang L, Wang Y, Wang Q, Collier DA, Gong Q, Li T | Psychiatry research |
| 103 | [Assessments of function and biochemistry of the anterior cingulate cortex in schizophrenia.](https://neurosynth.org/studies/20570244/) | Reid MA, Stoeckel LE, White DM, Avsar KB, Bolding MS, Akella NS, Knowlton RC, den Hollander JA, Lahti AC | Biological psychiatry |
| 104 | [Association between a longer duration of illness, age and lower frontal lobe grey matter volume in schizophrenia.](https://neurosynth.org/studies/18586335/) | Premkumar P, Fannon D, Kuipers E, Cooke MA, Simmons A, Kumari V | Behavioural brain research |
| 105 | [Association between symptoms of psychosis and reduced functional connectivity of auditory cortex.](https://neurosynth.org/studies/25464916/) | Oertel-Knochel V, Knochel C, Matura S, Stablein M, Prvulovic D, Maurer K, Linden DE, van de Ven V | Schizophrenia research |
| 106 | [Association of 5' end neuregulin-1 (NRG1) gene variation with subcortical medial frontal microstructure in humans.](https://neurosynth.org/studies/18255317/) | Winterer G, Konrad A, Vucurevic G, Musso F, Stoeter P, Dahmen N | NeuroImage |
| 107 | [Association of familial risk for schizophrenia with thalamic and medial prefrontal functional connectivity during attentional control.](https://neurosynth.org/studies/27012899/) | Antonucci LA, Taurisano P, Fazio L, Gelao B, Romano R, Quarto T, Porcelli A, Mancini M, Di Giorgio A, Caforio G, Pergola G, Popolizio T, Bertolino A, Blasi G | Schizophrenia research |
| 108 | [Association of grey matter volume deviation with insight impairment in first-episode affective and non-affective psychosis.](https://neurosynth.org/studies/22673767/) | McFarland J, Cannon DM, Schmidt H, Ahmed M, Hehir S, Emsell L, Barker G, McCarthy P, Elliott MA, McDonald C | European archives of psychiatry and clinical neuroscience |
| 109 | [Association of medial prefrontal resting state functional connectivity and metacognitive capacity in early phase psychosis.](https://neurosynth.org/studies/28208070/) | Francis MM, Hummer TA, Leonhardt BL, Vohs JL, Yung MG, Mehdiyoun NF, Lysaker PH, Breier A | Psychiatry research. Neuroimaging |
| 110 | [Association of poor insight in schizophrenia with structure and function of cortical midline structures and frontopolar cortex.](https://neurosynth.org/studies/22664168/) | Raij TT, Riekki TJ, Hari R | Schizophrenia research |
| 111 | [Association of the SerCys DISC1 polymorphism with human hippocampal formation gray matter and function during memory encoding.](https://neurosynth.org/studies/19046394/) | Di Giorgio A, Blasi G, Sambataro F, Rampino A, Papazacharias A, Gambi F, Romano R, Caforio G, Rizzo M, Latorre V, Popolizio T, Kolachana B, Callicott JH, Nardini M, Weinberger DR, Bertolino A | The European journal of neuroscience |
| 112 | [Associations between polygenic risk for schizophrenia and brain function during probabilistic learning in healthy individuals.](https://neurosynth.org/studies/26510167/) | Lancaster TM, Ihssen N, Brindley LM, Tansey KE, Mantripragada K, O'Donovan MC, Owen MJ, Linden DE | Human brain mapping |
| 113 | [Associative memory encoding and recognition in schizophrenia: an event-related fMRI study.](https://neurosynth.org/studies/16814264/) | Lepage M, Montoya A, Pelletier M, Achim AM, Menear M, Lal S | Biological psychiatry |
| 114 | [Attention orienting dysfunction during salient novel stimulus processing in schizophrenia.](https://neurosynth.org/studies/15885507/) | Laurens KR, Kiehl KA, Ngan ET, Liddle PF | Schizophrenia research |
| 115 | [Attenuated frontal activation in schizophrenia may be task dependent.](https://neurosynth.org/studies/10227106/) | Curtis VA, Bullmore ET, Morris RG, Brammer MJ, Williams SC, Simmons A, Sharma T, Murray RM, McGuire PK | Schizophrenia research |
| 116 | [Attenuated prefrontal activation during decision-making under uncertainty in schizophrenia: a multi-center fMRI study.](https://neurosynth.org/studies/24325976/) | Krug A, Cabanis M, Pyka M, Pauly K, Kellermann T, Walter H, Wagner M, Landsberg M, Shah NJ, Winterer G, Wolwer W, Brinkmeyer J, Muller BW, Kargel C, Wiedemann G, Herrlich J, Vogeley K, Schilbach L, Rapp A, Klingberg S, Kircher T | Schizophrenia research |
| 117 | [Audio-visual speech perception in schizophrenia: an fMRI study.](https://neurosynth.org/studies/11231095/) | Surguladze SA, Calvert GA, Brammer MJ, Campbell R, Bullmore ET, Giampietro V, David AS | Psychiatry research |
| 118 | [Auditory hallucinations in schizophrenia are associated with reduced functional connectivity of the temporo-parietal area.](https://neurosynth.org/studies/20060103/) | Vercammen A, Knegtering H, den Boer JA, Liemburg EJ, Aleman A | Biological psychiatry |
| 119 | [Auditory mismatch impairments are characterized by core neural dysfunctions in schizophrenia.](https://neurosynth.org/studies/25743635/) | Gaebler AJ, Mathiak K, Koten JW Jr, Konig AA, Koush Y, Weyer D, Depner C, Matentzoglu S, Edgar JC, Willmes K, Zvyagintsev M | Brain : a journal of neurology |
| 120 | [Auditory verbal hallucinations predominantly activate the right inferior frontal area.](https://neurosynth.org/studies/18854323/) | Sommer IE, Diederen KM, Blom JD, Willems A, Kushan L, Slotema K, Boks MP, Daalman K, Hoek HW, Neggers SF, Kahn RS | Brain : a journal of neurology |
| 121 | [Autistic disorders and schizophrenia: related or remote? An anatomical likelihood estimation.](https://neurosynth.org/studies/20805880/) | Cheung C, Yu K, Fung G, Leung M, Wong C, Li Q, Sham P, Chua S, McAlonan G | PloS one |
| 122 | [Automated classification of fMRI during cognitive control identifies more severely disorganized subjects with schizophrenia.](https://neurosynth.org/studies/22277668/) | Yoon JH, Nguyen DV, McVay LM, Deramo P, Minzenberg MJ, Ragland JD, Niendham T, Solomon M, Carter CS | Schizophrenia research |
| 123 | [Automatization and working memory capacity in schizophrenia.](https://neurosynth.org/studies/18155446/) | van Raalten TR, Ramsey NF, Jansma JM, Jager G, Kahn RS | Schizophrenia research |
| 124 | [Bilateral functional asymmetry disparity in positive and negative schizophrenia revealed by resting-state fMRI.](https://neurosynth.org/studies/20227250/) | Ke M, Zou R, Shen H, Huang X, Zhou Z, Liu Z, Xue Z, Hu D | Psychiatry research |
| 125 | [Blunted activation in right ventrolateral prefrontal cortex during motor response inhibition in schizophrenia.](https://neurosynth.org/studies/17855057/) | Kaladjian A, Jeanningros R, Azorin JM, Grimault S, Anton JL, Mazzola-Pomietto P | Schizophrenia research |
| 126 | [Both volumetry and functional connectivity of Heschl's gyrus are associated with auditory P300 in first episode schizophrenia.](https://neurosynth.org/studies/25458859/) | Guo Q, Tang Y, Li H, Zhang T, Li J, Sheng J, Liu D, Li C, Wang J | Schizophrenia research |
| 127 | [Brain activation during executive processes in schizophrenia.](https://neurosynth.org/studies/19643585/) | Royer A, Schneider FC, Grosselin A, Pellet J, Barral FG, Laurent B, Brouillet D, Lang F | Psychiatry research |
| 128 | [Brain activation induced by psychological stress in patients with schizophrenia.](https://neurosynth.org/studies/26190301/) | Castro MN, Villarreal MF, Bolotinsky N, Papavero E, Goldschmidt MG, Costanzo EY, Drucaroff L, Wainsztein A, de Achaval D, Pahissa J, Bar KJ, Nemeroff CB, Guinjoan SM | Schizophrenia research |
| 129 | [Brain activation patterns during a selective attention test--a functional MRI study in healthy volunteers and unmedicated patients during an acute episode of schizophrenia.](https://neurosynth.org/studies/17188464/) | Weiss EM, Siedentopf C, Golaszewski S, Mottaghy FM, Hofer A, Kremser C, Felber S, Fleischhacker WW | Psychiatry research |
| 130 | [Brain activation patterns during a selective attention test-a functional MRI study in healthy volunteers and patients with schizophrenia.](https://neurosynth.org/studies/12738340/) | Weiss EM, Golaszewski S, Mottaghy FM, Hofer A, Hausmann A, Kemmler G, Kremser C, Brinkhoff C, Felber SR, Fleischhacker WW | Psychiatry research |
| 131 | [Brain activation patterns during visual episodic memory processing among first-degree relatives of schizophrenia subjects.](https://neurosynth.org/studies/22992490/) | Stolz E, Pancholi KM, Goradia DD, Paul S, Keshavan MS, Nimgaonkar VL, Prasad KM | NeuroImage |
| 132 | [Brain activity during emotionally negative pictures in schizophrenia with and without flat affect: an fMRI study.](https://neurosynth.org/studies/16143498/) | Fahim C, Stip E, Mancini-Marie A, Mensour B, Boulay LJ, Leroux JM, Beaudoin G, Bourgouin P, Beauregard M | Psychiatry research |
| 133 | [Brain Correlates of Self-Evaluation Deficits in Schizophrenia: A Combined Functional and Structural MRI Study.](https://neurosynth.org/studies/26406464/) | Tan S, Zhao Y, Fan F, Zou Y, Jin Z, Zen Y, Zhu X, Yang F, Tan Y, Zhou D | PloS one |
| 134 | [Brain differences in first-episode schizophrenia treated with quetiapine: a deformation-based morphometric study.](https://neurosynth.org/studies/25080851/) | Yang C, Wu S, Lu W, Bai Y, Gao H | Psychopharmacology |
| 135 | [Brain dysfunctions during facial discrimination in schizophrenia: selective association to affect decoding.](https://neurosynth.org/studies/21145212/) | Quintana J, Lee J, Marcus M, Kee K, Wong T, Yerevanian A | Psychiatry research |
| 136 | [Brain effects of cognitive remediation therapy in schizophrenia: a structural and functional neuroimaging study.](https://neurosynth.org/studies/23452665/) | Penades R, Pujol N, Catalan R, Massana G, Rametti G, Garcia-Rizo C, Bargallo N, Gasto C, Bernardo M, Junque C | Biological psychiatry |
| 137 | [Brain gray matter phenotypes across the psychosis dimension.](https://neurosynth.org/studies/23177922/) | Ivleva EI, Bidesi AS, Thomas BP, Meda SA, Francis A, Moates AF, Witte B, Keshavan MS, Tamminga CA | Psychiatry research |
| 138 | [Brain response abnormalities during verbal learning among patients with schizophrenia.](https://neurosynth.org/studies/18055184/) | Eyler LT, Jeste DV, Brown GG | Psychiatry research |
| 139 | [Brain structural changes in schizophrenia patients with persistent hallucinations.](https://neurosynth.org/studies/17720459/) | O'Daly OG, Frangou S, Chitnis X, Shergill SS | Psychiatry research |
| 140 | [Brain structural correlates of schizotypy and psychosis proneness in a non-clinical healthy volunteer sample.](https://neurosynth.org/studies/26164819/) | Nenadic I, Lorenz C, Langbein K, Dietzek M, Smesny S, Schonfeld N, Fananas L, Sauer H, Gaser C | Schizophrenia research |
| 141 | [Brain structure and function correlates of cognitive subtypes in schizophrenia.](https://neurosynth.org/studies/26341950/) | Geisler D, Walton E, Naylor M, Roessner V, Lim KO, Charles Schulz S, Gollub RL, Calhoun VD, Sponheim SR, Ehrlich S | Psychiatry research |
| 142 | [Brain-behaviour relationships in people at high genetic risk of schizophrenia.](https://neurosynth.org/studies/16926102/) | Lymer GK, Job DE, William T, Moorhead J, McIntosh AM, Owens DG, Johnstone EC, Lawrie SM | NeuroImage |
| 143 | [Breakdown of the striatal-default mode network loop in schizophrenia.](https://neurosynth.org/studies/26260079/) | Wang X, Li F, Zheng H, Wang W, Zhang W, Liu Z, Sun Y, Chan RC, Chen A | Schizophrenia research |
| 144 | [CACNA1C risk variant and amygdala activity in bipolar disorder, schizophrenia and healthy controls.](https://neurosynth.org/studies/23437284/) | Tesli M, Skatun KC, Ousdal OT, Brown AA, Thoresen C, Agartz I, Melle I, Djurovic S, Jensen J, Andreassen OA | PloS one |
| 145 | [Cannabis abuse is associated with better emotional memory in schizophrenia: a functional magnetic resonance imaging study.](https://neurosynth.org/studies/23906663/) | Bourque J, Mendrek A, Durand M, Lakis N, Lipp O, Stip E, Lalonde P, Grignon S, Potvin S | Psychiatry research |
| 146 | [Central executive network in young people with familial risk for psychosis - The Oulu Brain and Mind Study.](https://neurosynth.org/studies/25468181/) | Jukuri T, Kiviniemi V, Nikkinen J, Miettunen J, Maki P, Mukkala S, Koivukangas J, Nordstrom T, Parkkisenniemi J, Moilanen I, Barnett JH, Jones PB, Murray GK, Veijola J | Schizophrenia research |
| 147 | [Cerebral blood flow and its connectivity features of auditory verbal hallucinations in schizophrenia: A perfusion study.](https://neurosynth.org/studies/28024236/) | Cui LB, Chen G, Xu ZL, Liu L, Wang HN, Guo L, Liu WM, Liu TT, Qi S, Liu K, Qin W, Sun JB, Xi YB, Yin H | Psychiatry research. Neuroimaging |
| 148 | [Cerebral connectivity and psychotic personality traits. A diffusion tensor imaging study.](https://neurosynth.org/studies/18299790/) | Volpe U, Federspiel A, Mucci A, Dierks T, Frank A, Wahlund LO, Galderisi S, Maj M | European archives of psychiatry and clinical neuroscience |
| 149 | [Cerebral Inefficient Activation in Schizophrenia Patients and Their Unaffected Parents during the N-Back Working Memory Task: A Family fMRI Study.](https://neurosynth.org/studies/26270056/) | Jiang S, Yan H, Chen Q, Tian L, Lu T, Tan HY, Yan J, Zhang D | PloS one |
| 150 | [Changes in prefrontal and amygdala activity during olanzapine treatment in schizophrenia.](https://neurosynth.org/studies/19428222/) | Blasi G, Popolizio T, Taurisano P, Caforio G, Romano R, Di Giorgio A, Sambataro F, Rubino V, Latorre V, Lo Bianco L, Fazio L, Nardini M, Weinberger DR, Bertolino A | Psychiatry research |
| 151 | [Cingulate activity and fronto-temporal connectivity in people with prodromal signs of psychosis.](https://neurosynth.org/studies/19703570/) | Allen P, Stephan KE, Mechelli A, Day F, Ward N, Dalton J, Williams SC, McGuire P | NeuroImage |
| 152 | [Classification of adolescent psychotic disorders using linear discriminant analysis.](https://neurosynth.org/studies/16797923/) | Pardo PJ, Georgopoulos AP, Kenny JT, Stuve TA, Findling RL, Schulz SC | Schizophrenia research |
| 153 | [Classification of schizophrenia and bipolar patients using static and dynamic resting-state fMRI brain connectivity.](https://neurosynth.org/studies/27118088/) | Rashid B, Arbabshirani MR, Damaraju E, Cetin MS, Miller R, Pearlson GD, Calhoun VD | NeuroImage |
| 154 | [Classification of schizophrenia patients based on resting-state functional network connectivity.](https://neurosynth.org/studies/23966903/) | Arbabshirani MR, Kiehl KA, Pearlson GD, Calhoun VD | Frontiers in neuroscience |
| 155 | [Cognitive and neural strategies during control of the anterior cingulate cortex by fMRI neurofeedback in patients with schizophrenia.](https://neurosynth.org/studies/26161073/) | Cordes JS, Mathiak KA, Dyck M, Alawi EM, Gaber TJ, Zepf FD, Klasen M, Zvyagintsev M, Gur RC, Mathiak K | Frontiers in behavioral neuroscience |
| 156 | [Cognitive and psychopathology correlates of brain white/grey matter structure in severely psychotic schizophrenic inpatients.](https://neurosynth.org/studies/29527507/) | Banaj N, Piras F, Piras F, Ciullo V, Iorio M, Battaglia C, Pantoli D, Ducci G, Spalletta G | Schizophrenia research. Cognition |
| 157 | [Cognitive correlates of gray matter abnormalities in adolescent siblings of patients with childhood-onset schizophrenia.](https://neurosynth.org/studies/25541139/) | Wagshal D, Knowlton BJ, Cohen JR, Bookheimer SY, Bilder RM, Fernandez VG, Asarnow RF | Schizophrenia research |
| 158 | [Cognitive insight in first-episode schizophrenia: Further evidence for a role of the ventrolateral prefrontal cortex.](https://neurosynth.org/studies/26004692/) | Buchy L, Hawco C, Joober R, Malla A, Lepage M | Schizophrenia research |
| 159 | [Cognitive performance is related to cortical grey matter volumes in early stages of schizophrenia: a population-based study of first-episode psychosis.](https://neurosynth.org/studies/19616413/) | Minatogawa-Chang TM, Schaufelberger MS, Ayres AM, Duran FL, Gutt EK, Murray RM, Rushe TM, McGuire PK, Menezes PR, Scazufca M, Busatto GF | Schizophrenia research |
| 160 | [Cognitive state and connectivity effects of the genome-wide significant psychosis variant in ZNF804A.](https://neurosynth.org/studies/20946959/) | Esslinger C, Kirsch P, Haddad L, Mier D, Sauer C, Erk S, Schnell K, Arnold C, Witt SH, Rietschel M, Cichon S, Walter H, Meyer-Lindenberg A | NeuroImage |
| 161 | [Color Stroop and negative priming in schizophrenia: an fMRI study.](https://neurosynth.org/studies/19963356/) | Ungar L, Nestor PG, Niznikiewicz MA, Wible CG, Kubicki M | Psychiatry research |
| 162 | [Combination of Resting State fMRI, DTI, and sMRI Data to Discriminate Schizophrenia by N-way MCCA + jICA.](https://neurosynth.org/studies/23755002/) | Sui J, He H, Yu Q, Chen J, Rogers J, Pearlson GD, Mayer A, Bustillo J, Canive J, Calhoun VD | Frontiers in human neuroscience |
| 163 | [Combining fMRI and SNP data to investigate connections between brain function and genetics using parallel ICA.](https://neurosynth.org/studies/18072279/) | Liu J, Pearlson G, Windemuth A, Ruano G, Perrone-Bizzozero NI, Calhoun V | Human brain mapping |
| 164 | [Common and distinct neural effects of risperidone and olanzapine during procedural learning in schizophrenia: a randomised longitudinal fMRI study.](https://neurosynth.org/studies/25980483/) | Kumari V, Ettinger U, Lee SE, Deuschl C, Anilkumar AP, Schmechtig A, Corr PJ, Ffytche DH, Williams SC | Psychopharmacology |
| 165 | [Common and distinct structural features of schizophrenia and bipolar disorder: The European Network on Psychosis, Affective disorders and Cognitive Trajectory (ENPACT) study.](https://neurosynth.org/studies/29136642/) | Maggioni E, Crespo-Facorro B, Nenadic I, Benedetti F, Gaser C, Sauer H, Roiz-Santianez R, Poletti S, Marinelli V, Bellani M, Perlini C, Ruggeri M, Altamura AC, Diwadkar VA, Brambilla P | PloS one |
| 166 | [Common neural circuitry supporting volitional saccades and its disruption in schizophrenia patients and relatives.](https://neurosynth.org/studies/18692173/) | Camchong J, Dyckman KA, Austin BP, Clementz BA, McDowell JE | Biological psychiatry |
| 167 | [Comparing the neural bases of self-referential processing in typically developing and 22q11.2 adolescents.](https://neurosynth.org/studies/22483077/) | Schneider M, Debbane M, Lagioia A, Salomon R, d'Argembeau A, Eliez S | Developmental cognitive neuroscience |
| 168 | [Comparison of structural covariance with functional connectivity approaches exemplified by an investigation of the left anterior insula.](https://neurosynth.org/studies/24844743/) | Clos M, Rottschy C, Laird AR, Fox PT, Eickhoff SB | NeuroImage |
| 169 | [Compensatory mechanisms underlie intact task-switching performance in schizophrenia.](https://neurosynth.org/studies/20036266/) | Jamadar S, Michie P, Karayanidis F | Neuropsychologia |
| 170 | [COMT genotype and its role on hippocampal-prefrontal regions in declarative memory.](https://neurosynth.org/studies/20060911/) | Krach S, Jansen A, Krug A, Markov V, Thimm M, Sheldrick AJ, Eggermann T, Zerres K, Stocker T, Shah NJ, Kircher T | NeuroImage |
| 171 | [Concurrent functional magnetic resonance imaging and electroencephalography assessment of sensory gating in schizophrenia.](https://neurosynth.org/studies/24375687/) | Bak N, Rostrup E, Larsson HB, Glenthoj BY, Oranje B | Human brain mapping |
| 172 | [Consequences of magnocellular dysfunction on processing attended information in schizophrenia.](https://neurosynth.org/studies/21840846/) | Martinez A, Hillyard SA, Bickel S, Dias EC, Butler PD, Javitt DC | Cerebral cortex (New York, N.Y. : 1991) |
| 173 | [Contributions of low and high spatial frequency processing to impaired object recognition circuitry in schizophrenia.](https://neurosynth.org/studies/22735157/) | Calderone DJ, Hoptman MJ, Martinez A, Nair-Collins S, Mauro CJ, Bar M, Javitt DC, Butler PD | Cerebral cortex (New York, N.Y. : 1991) |
| 174 | [Convergence of EEG and fMRI measures of reward anticipation.](https://neurosynth.org/studies/26394333/) | Gorka SM, Phan KL, Shankman SA | Biological psychology |
| 175 | [Convergent evidence from multimodal imaging reveals amygdala abnormalities in schizophrenic patients and their first-degree relatives.](https://neurosynth.org/studies/22174900/) | Tian L, Meng C, Yan H, Zhao Q, Liu Q, Yan J, Han Y, Yuan H, Wang L, Yue W, Zhang Y, Li X, Zhu C, He Y, Zhang D | PloS one |
| 176 | [Converging genetic and functional brain imaging evidence links neuronal excitability to working memory, psychiatric disease, and brain activity.](https://neurosynth.org/studies/24529980/) | Heck A, Fastenrath M, Ackermann S, Auschra B, Bickel H, Coynel D, Gschwind L, Jessen F, Kaduszkiewicz H, Maier W, Milnik A, Pentzek M, Riedel-Heller SG, Ripke S, Spalek K, Sullivan P, Vogler C, Wagner M, Weyerer S, Wolfsgruber S, de Quervain DJ, Papassotiropoulos A | Neuron |
| 177 | [Correlated structural and functional brain abnormalities in the default mode network in schizophrenia patients.](https://neurosynth.org/studies/21095105/) | Salgado-Pineda P, Fakra E, Delaveau P, McKenna PJ, Pomarol-Clotet E, Blin O | Schizophrenia research |
| 178 | [Correlation of passivity symptoms and dysfunctional visuomotor action monitoring in psychosis.](https://neurosynth.org/studies/18713781/) | Schnell K, Heekeren K, Daumann J, Schnell T, Schnitker R, Moller-Hartmann W, Gouzoulis-Mayfrank E | Brain : a journal of neurology |
| 179 | [Cortex and amygdala morphology in psychopathy.](https://neurosynth.org/studies/21676597/) | Boccardi M, Frisoni GB, Hare RD, Cavedo E, Najt P, Pievani M, Rasser PE, Laakso MP, Aronen HJ, Repo-Tiihonen E, Vaurio O, Thompson PM, Tiihonen J | Psychiatry research |
| 180 | [Cortical intercorrelations of frontal area volumes in schizophrenia.](https://neurosynth.org/studies/15990338/) | Mitelman SA, Buchsbaum MS, Brickman AM, Shihabuddin L | NeuroImage |
| 181 | [Cortical intercorrelations of temporal area volumes in schizophrenia.](https://neurosynth.org/studies/15949654/) | Mitelman SA, Shihabuddin L, Brickman AM, Buchsbaum MS | Schizophrenia research |
| 182 | [Cortical signature of neurological soft signs in recent onset schizophrenia.](https://neurosynth.org/studies/23660871/) | Hirjak D, Wolf RC, Stieltjes B, Hauser T, Seidl U, Schroder J, Thomann PA | Brain topography |
| 183 | [Cortical-basal ganglia imbalance in schizophrenia patients and unaffected first-degree relatives.](https://neurosynth.org/studies/22464726/) | Oertel-Knochel V, Knochel C, Matura S, Rotarska-Jagiela A, Magerkurth J, Prvulovic D, Haenschel C, Hampel H, Linden DE | Schizophrenia research |
| 184 | [Cortico-striatal disconnection within the cingulo-opercular network in schizophrenia revealed by intrinsic functional connectivity analysis: a resting fMRI study.](https://neurosynth.org/studies/21840407/) | Tu PC, Hsieh JC, Li CT, Bai YM, Su TP | NeuroImage |
| 185 | [Corticolimbic dysfunction during facial and prosodic emotional recognition in first-episode psychosis patients and individuals at ultra-high risk.](https://neurosynth.org/studies/27747152/) | Tseng HH, Roiser JP, Modinos G, Falkenberg I, Samson C, McGuire P, Allen P | NeuroImage. Clinical |
| 186 | [De-coupling of cognitive performance and cerebral functional response during working memory in schizophrenia.](https://neurosynth.org/studies/11728837/) | Honey GD, Bullmore ET, Sharma T | Schizophrenia research |
| 187 | [Decreased cerebral activation during CPT performance: structural and functional deficits in schizophrenic patients.](https://neurosynth.org/studies/15006650/) | Salgado-Pineda P, Junque C, Vendrell P, Baeza I, Bargallo N, Falcon C, Bernardo M | NeuroImage |
| 188 | [Decreased fMRI activity in the hippocampus of patients with schizophrenia compared to healthy control participants, tested on a wayfinding task in a virtual town.](https://neurosynth.org/studies/23352276/) | Ledoux AA, Phillips JL, Labelle A, Smith A, Bohbot VD, Boyer P | Psychiatry research |
| 189 | [Decreased hemispheric connectivity and decreased intra- and inter- hemisphere asymmetry of resting state functional network connectivity in schizophrenia.](https://neurosynth.org/studies/28434159/) | Agcaoglu O, Miller R, Damaraju E, Rashid B, Bustillo J, Cetin MS, Van Erp TGM, McEwen S, Preda A, Ford JM, Lim KO, Manoach DS, Mathalon DH, Potkin SG, Calhoun VD | Brain imaging and behavior |
| 190 | [Decreased left middle temporal gyrus volume in antipsychotic drug-naive, first-episode schizophrenia patients and their healthy unaffected siblings.](https://neurosynth.org/studies/23360727/) | Hu M, Li J, Eyler L, Guo X, Wei Q, Tang J, Liu F, He Z, Li L, Jin H, Liu Z, Wang J, Liu F, Chen H, Zhao J | Schizophrenia research |
| 191 | [Decreased regional activity of default-mode network in unaffected siblings of schizophrenia patients at rest.](https://neurosynth.org/studies/24491950/) | Guo W, Su Q, Yao D, Jiang J, Zhang J, Zhang Z, Yu L, Zhai J, Xiao C | European neuropsychopharmacology : the journal of the European College of Neuropsychopharmacology |
| 192 | [Decreasing predictability of visual motion enhances feed-forward processing in visual cortex when stimuli are behaviorally relevant.](https://neurosynth.org/studies/27334340/) | Kellermann T, Scholle R, Schneider F, Habel U | Brain structure & function |
| 193 | [Default mode network activity in schizophrenia studied at resting state using probabilistic ICA.](https://neurosynth.org/studies/22578721/) | Mingoia G, Wagner G, Langbein K, Maitra R, Smesny S, Dietzek M, Burmeister HP, Reichenbach JR, Schlosser RG, Gaser C, Sauer H, Nenadic I | Schizophrenia research |
| 194 | [Default mode network connectivity and reciprocal social behavior in 22q11.2 deletion syndrome.](https://neurosynth.org/studies/23912681/) | Schreiner MJ, Karlsgodt KH, Uddin LQ, Chow C, Congdon E, Jalbrzikowski M, Bearden CE | Social cognitive and affective neuroscience |
| 195 | [Default mode network connectivity as a function of familial and environmental risk for psychotic disorder.](https://neurosynth.org/studies/25790002/) | Peeters SC, van de Ven V, Gronenschild EH, Patel AX, Habets P, Goebel R, van Os J, Marcelis M | PloS one |
| 196 | [Default mode network in young people with familial risk for psychosis--the Oulu Brain and Mind study.](https://neurosynth.org/studies/23245776/) | Jukuri T, Kiviniemi V, Nikkinen J, Miettunen J, Maki P, Jaaskelainen E, Mukkala S, Koivukangas J, Nordstrom T, Taanila A, Moilanen I, Heinimaa M, Barnett JH, Jones PB, Murray GK, Veijola J | Schizophrenia research |
| 197 | [Default-mode network dysfunction and self-referential processing in healthy siblings of schizophrenia patients.](https://neurosynth.org/studies/23099059/) | van Buuren M, Vink M, Kahn RS | Schizophrenia research |
| 198 | [Deficit in schizophrenia to recruit the striatum in implicit learning: a functional magnetic resonance imaging investigation.](https://neurosynth.org/studies/16814986/) | Reiss JP, Campbell DW, Leslie WD, Paulus MP, Ryner LN, Polimeni JO, Foot BJ, Sareen J | Schizophrenia research |
| 199 | [Delayed hemodynamic responses in schizophrenia.](https://neurosynth.org/studies/15955502/) | Ford JM, Johnson MB, Whitfield SL, Faustman WO, Mathalon DH | NeuroImage |
| 200 | [Diagnostic classification of schizophrenia patients on the basis of regional reward-related FMRI signal patterns.](https://neurosynth.org/studies/25799236/) | Koch SP, Hagele C, Haynes JD, Heinz A, Schlagenhauf F, Sterzer P | PloS one |
| 201 | [Differences in frontal cortical activation by a working memory task after substitution of risperidone for typical antipsychotic drugs in patients with schizophrenia.](https://neurosynth.org/studies/10557338/) | Honey GD, Bullmore ET, Soni W, Varatheesan M, Williams SC, Sharma T | Proceedings of the National Academy of Sciences of the United States of America |
| 202 | [Differential amygdala response during facial recognition in patients with schizophrenia: an fMRI study.](https://neurosynth.org/studies/12165379/) | Kosaka H, Omori M, Murata T, Iidaka T, Yamada H, Okada T, Takahashi T, Sadato N, Itoh H, Yonekura Y, Wada Y | Schizophrenia research |
| 203 | [Differential effects of erythropoietin on neural and cognitive measures of executive function 3 and 7 days post-administration.](https://neurosynth.org/studies/17828390/) | Miskowiak K, Inkster B, O'Sullivan U, Selvaraj S, Goodwin GM, Harmer CJ | Experimental brain research |
| 204 | [Differential fractional anisotropy abnormalities in adolescents with ADHD or schizophrenia.](https://neurosynth.org/studies/20153608/) | Davenport ND, Karatekin C, White T, Lim KO | Psychiatry research |
| 205 | [Differential frontal activation in schizophrenia and bipolar illness during verbal fluency.](https://neurosynth.org/studies/11578663/) | Curtis VA, Dixon TA, Morris RG, Bullmore ET, Brammer MJ, Williams SC, Sharma T, Murray RM, McGuire PK | Journal of affective disorders |
| 206 | [Differential processing of metacognitive evaluation and the neural circuitry of the self and others in schizophrenia: a pilot study.](https://neurosynth.org/studies/20051318/) | Murphy ER, Brent BK, Benton M, Pruitt P, Diwadkar V, Rajarethinam RP, Keshavan MS | Schizophrenia research |
| 207 | [Differential roles of the frontal and parietal cortices in the control of saccades.](https://neurosynth.org/studies/23867736/) | Bender J, Tark KJ, Reuter B, Kathmann N, Curtis CE | Brain and cognition |
| 208 | [Dimensional schizotypy and social cognition: an fMRI imaging study.](https://neurosynth.org/studies/26074796/) | Wang Y, Liu WH, Li Z, Wei XH, Jiang XQ, Neumann DL, Shum DH, Cheung EF, Chan RC | Frontiers in behavioral neuroscience |
| 209 | [Diminished neural sensitivity to irregular facial expression in first-episode schizophrenia.](https://neurosynth.org/studies/19172653/) | Bleich-Cohen M, Strous RD, Even R, Rotshtein P, Yovel G, Iancu I, Olmer A, Hendler T | Human brain mapping |
| 210 | [Discrete neural substrates underlie complementary audiovisual speech integration processes.](https://neurosynth.org/studies/21195198/) | Stevenson RA, VanDerKlok RM, Pisoni DB, James TW | NeuroImage |
| 211 | [Discriminant analysis of functional connectivity patterns on Grassmann manifold.](https://neurosynth.org/studies/21440643/) | Fan Y, Liu Y, Wu H, Hao Y, Liu H, Liu Z, Jiang T | NeuroImage |
| 212 | [Discriminating imagined from perceived information engages brain areas implicated in schizophrenia.](https://neurosynth.org/studies/16797186/) | Simons JS, Davis SW, Gilbert SJ, Frith CD, Burgess PW | NeuroImage |
| 213 | [Disrupted amplitude of low-frequency fluctuations in antipsychotic-naive adolescents with early-onset schizophrenia.](https://neurosynth.org/studies/27000303/) | Zheng J, Zhang Y, Guo X, Duan X, Zhang J, Zhao J, Chen H | Psychiatry research. Neuroimaging |
| 214 | [Disrupted functional connectivity for controlled visual processing as a basis for impaired spatial working memory in schizophrenia.](https://neurosynth.org/studies/21703287/) | Kang SS, Sponheim SR, Chafee MV, MacDonald AW 3rd | Neuropsychologia |
| 215 | [Disrupted network cross talk, hippocampal dysfunction and hallucinations in schizophrenia.](https://neurosynth.org/studies/29571753/) | Hare SM, Law AS, Ford JM, Mathalon DH, Ahmadi A, Damaraju E, Bustillo J, Belger A, Lee HJ, Mueller BA, Lim KO, Brown GG, Preda A, van Erp TGM, Potkin SG, Calhoun VD, Turner JA | Schizophrenia research |
| 216 | [Disrupted salience processing involved in motivational deficits for real-life activities in patients with schizophrenia.](https://neurosynth.org/studies/29395610/) | Kim BH, Shin YB, Kyeong S, Lee SK, Kim JJ | Schizophrenia research |
| 217 | [Disrupted sensorimotor and social-cognitive networks underlie symptoms in childhood-onset schizophrenia.](https://neurosynth.org/studies/26493637/) | Berman RA, Gotts SJ, McAdams HM, Greenstein D, Lalonde F, Clasen L, Watsky RE, Shora L, Ordonez AE, Raznahan A, Martin A, Gogtay N, Rapoport J | Brain : a journal of neurology |
| 218 | [Disrupted thalamic resting-state functional networks in schizophrenia.](https://neurosynth.org/studies/25762911/) | Wang HL, Rau CL, Li YM, Chen YP, Yu R | Frontiers in behavioral neuroscience |
| 219 | [Disrupted white matter integrity of corticopontine-cerebellar circuitry in schizophrenia.](https://neurosynth.org/studies/19915989/) | Koch K, Wagner G, Dahnke R, Schachtzabel C, Schultz C, Roebel M, Gullmar D, Reichenbach JR, Sauer H, Schlosser RG | European archives of psychiatry and clinical neuroscience |
| 220 | [Disruption of anterior insula modulation of large-scale brain networks in schizophrenia.](https://neurosynth.org/studies/23623456/) | Moran LV, Tagamets MA, Sampath H, O'Donnell A, Stein EA, Kochunov P, Hong LE | Biological psychiatry |
| 221 | [Disruption of learned irrelevance in acute schizophrenia in a novel continuous within-subject paradigm suitable for fMRI.](https://neurosynth.org/studies/15582114/) | Young AM, Kumari V, Mehrotra R, Hemsley DR, Andrew C, Sharma T, Williams SC, Gray JA | Behavioural brain research |
| 222 | [Disruptive changes of cerebellar functional connectivity with the default mode network in schizophrenia.](https://neurosynth.org/studies/25445623/) | Wang L, Zou F, Shao Y, Ye E, Jin X, Tan S, Hu D, Yang Z | Schizophrenia research |
| 223 | [Dissociation of anatomical and functional alterations of the default-mode network in first-episode, drug-naive schizophrenia.](https://neurosynth.org/studies/25746945/) | Guo W, Liu F, Xiao C, Zhang Z, Yu M, Liu J, Liu G, Zhao J | Clinical neurophysiology : official journal of the International Federation of Clinical Neurophysiology |
| 224 | [Dissociation of functional and anatomical brain abnormalities in unaffected siblings of schizophrenia patients.](https://neurosynth.org/studies/25240248/) | Guo W, Song Y, Liu F, Zhang Z, Zhang J, Yu M, Liu J, Xiao C, Liu G, Zhao J | Clinical neurophysiology : official journal of the International Federation of Clinical Neurophysiology |
| 225 | [Distinct neural correlates for attention lapses in patients with schizophrenia and healthy participants.](https://neurosynth.org/studies/26500517/) | Phillips RC, Salo T, Carter CS | Frontiers in human neuroscience |
| 226 | [Distinct structural alterations independently contributing to working memory deficits and symptomatology in paranoid schizophrenia.](https://neurosynth.org/studies/23040316/) | Zierhut KC, Schulte-Kemna A, Kaufmann J, Steiner J, Bogerts B, Schiltz K | Cortex; a journal devoted to the study of the nervous system and behavior |
| 227 | [Distinct structural neural patterns of trait physical and social anhedonia: Evidence from cortical thickness, subcortical volumes and inter-regional correlations.](https://neurosynth.org/studies/25288478/) | Wang Y, Deng Y, Fung G, Liu WH, Wei XH, Jiang XQ, Lui SS, Cheung EF, Chan RC | Psychiatry research |
| 228 | [Disturbed functional connectivity of cortical activation during semantic discrimination in patients with schizophrenia and subjects at genetic high-risk.](https://neurosynth.org/studies/20503118/) | Li X, Branch CA, Nierenberg J, Delisi LE | Brain imaging and behavior |
| 229 | [Disturbed sexual dimorphism of brain activation during mental rotation in schizophrenia.](https://neurosynth.org/studies/20385471/) | Jimenez JA, Mancini-Marie A, Lakis N, Rinaldi M, Mendrek A | Schizophrenia research |
| 230 | [Do you hear what I hear? Neural correlates of thought disorder during listening to speech in schizophrenia.](https://neurosynth.org/studies/16806838/) | Weinstein S, Werker JF, Vouloumanos A, Woodward TS, Ngan ET | Schizophrenia research |
| 231 | [Dopamine precursor depletion impairs timing in healthy volunteers by attenuating activity in putamen and supplementary motor area.](https://neurosynth.org/studies/23175824/) | Coull JT, Hwang HJ, Leyton M, Dagher A | The Journal of neuroscience : the official journal of the Society for Neuroscience |
| 232 | [Dopamine-dependent architecture of cortico-subcortical network connectivity.](https://neurosynth.org/studies/22645252/) | Cole DM, Oei NY, Soeter RP, Both S, van Gerven JM, Rombouts SA, Beckmann CF | Cerebral cortex (New York, N.Y. : 1991) |
| 233 | [Dopaminergic basis of the psychosis-prone personality investigated with functional magnetic resonance imaging of procedural learning.](https://neurosynth.org/studies/23596404/) | Ettinger U, Corr PJ, Mofidi A, Williams SC, Kumari V | Frontiers in human neuroscience |
| 234 | [Dopaminergic modulation of the reward system in schizophrenia: a placebo-controlled dopamine depletion fMRI study.](https://neurosynth.org/studies/23978392/) | da Silva Alves F, Bakker G, Schmitz N, Abeling N, Hasler G, van der Meer J, Nederveen A, de Haan L, Linszen D, van Amelsvoort T | European neuropsychopharmacology : the journal of the European College of Neuropsychopharmacology |
| 235 | [Dorsolateral prefrontal cortex activity predicts responsiveness to cognitive-behavioral therapy in schizophrenia.](https://neurosynth.org/studies/19560121/) | Kumari V, Peters ER, Fannon D, Antonova E, Premkumar P, Anilkumar AP, Williams SC, Kuipers E | Biological psychiatry |
| 236 | [Dynamic functional connectivity analysis reveals transient states of dysconnectivity in schizophrenia.](https://neurosynth.org/studies/25161896/) | Damaraju E, Allen EA, Belger A, Ford JM, McEwen S, Mathalon DH, Mueller BA, Pearlson GD, Potkin SG, Preda A, Turner JA, Vaidya JG, van Erp TG, Calhoun VD | NeuroImage. Clinical |
| 237 | [Dynamic reconfiguration of frontal brain networks during executive cognition in humans.](https://neurosynth.org/studies/26324898/) | Braun U, Schafer A, Walter H, Erk S, Romanczuk-Seiferth N, Haddad L, Schweiger JI, Grimm O, Heinz A, Tost H, Meyer-Lindenberg A, Bassett DS | Proceedings of the National Academy of Sciences of the United States of America |
| 238 | [Dysfunction of the social brain in schizophrenia is modulated by intention type: an fMRI study.](https://neurosynth.org/studies/19287044/) | Walter H, Ciaramidaro A, Adenzato M, Vasic N, Ardito RB, Erk S, Bara BG | Social cognitive and affective neuroscience |
| 239 | [Dysfunction of ventral striatal reward prediction in schizophrenia.](https://neurosynth.org/studies/16139525/) | Juckel G, Schlagenhauf F, Koslowski M, Wustenberg T, Villringer A, Knutson B, Wrase J, Heinz A | NeuroImage |
| 240 | [Dysfunction of ventral striatal reward prediction in schizophrenic patients treated with typical, not atypical, neuroleptics.](https://neurosynth.org/studies/16721614/) | Juckel G, Schlagenhauf F, Koslowski M, Filonov D, Wustenberg T, Villringer A, Knutson B, Kienast T, Gallinat J, Wrase J, Heinz A | Psychopharmacology |
| 241 | [Dysfunctional modulation of emotional interference in the medial prefrontal cortex in patients with schizophrenia.](https://neurosynth.org/studies/18562102/) | Park IH, Park HJ, Chun JW, Kim EY, Kim JJ | Neuroscience letters |
| 242 | [Dysfunctional neural networks associated with impaired social interactions in early psychosis: an ICA analysis.](https://neurosynth.org/studies/23479058/) | Mazza M, Catalucci A, Pino MC, Giusti L, Nigri A, Pollice R, Roncone R, Casacchia M, Gallucci M | Brain imaging and behavior |
| 243 | [Dysfunctional resting-state connectivities of brain regions with structural deficits in drug-naive first-episode schizophrenia adolescents.](https://neurosynth.org/studies/26281967/) | Zhang Y, Zheng J, Fan X, Guo X, Guo W, Yang G, Chen H, Zhao J, Lv L | Schizophrenia research |
| 244 | [Dysregulated but not decreased salience network activity in schizophrenia.](https://neurosynth.org/studies/23471456/) | White TP, Gilleen J, Shergill SS | Frontiers in human neuroscience |
| 245 | [Dysregulation of working memory and default-mode networks in schizophrenia using independent component analysis, an fBIRN and MCIC study.](https://neurosynth.org/studies/19434601/) | Kim DI, Manoach DS, Mathalon DH, Turner JA, Mannell M, Brown GG, Ford JM, Gollub RL, White T, Wible C, Belger A, Bockholt HJ, Clark VP, Lauriello J, O'Leary D, Mueller BA, Lim KO, Andreasen N, Potkin SG, Calhoun VD | Human brain mapping |
| 246 | [Effect of CACNA1C rs1006737 on neural correlates of verbal fluency in healthy individuals.](https://neurosynth.org/studies/19781653/) | Krug A, Nieratschker V, Markov V, Krach S, Jansen A, Zerres K, Eggermann T, Stocker T, Shah NJ, Treutlein J, Muhleisen TW, Kircher T | NeuroImage |
| 247 | [Effect of retrieval effort and switching demand on fMRI activation during semantic word generation in schizophrenia.](https://neurosynth.org/studies/18155880/) | Ragland JD, Moelter ST, Bhati MT, Valdez JN, Kohler CG, Siegel SJ, Gur RC, Gur RE | Schizophrenia research |
| 248 | [Effect of rTMS on brain activation in schizophrenia with negative symptoms: A proof-of-principle study.](https://neurosynth.org/studies/26187147/) | Dlabac-de Lange JJ, Liemburg EJ, Bais L, Renken RJ, Knegtering H, Aleman A | Schizophrenia research |
| 49 | [Effects of age on prefrontal subregions and hippocampal volumes in young and middle-aged healthy humans.](https://neurosynth.org/studies/22488952/) | Wellington RL, Bilder RM, Napolitano B, Szeszko PR | Human brain mapping |
| 250 | [Effects of an alpha 7-nicotinic agonist on default network activity in schizophrenia.](https://neurosynth.org/studies/20728875/) | Tregellas JR, Tanabe J, Rojas DC, Shatti S, Olincy A, Johnson L, Martin LF, Soti F, Kem WR, Leonard S, Freedman R | Biological psychiatry |
| 251 | [Effects of an extra X chromosome on language lateralization: an fMRI study with Klinefelter men (47,XXY).](https://neurosynth.org/studies/18372164/) | van Rijn S, Aleman A, Swaab H, Vink M, Sommer I, Kahn RS | Schizophrenia research |
| 252 | [Effects of aripiprazole and haloperidol on neural activation during the n-back in healthy individuals: A functional MRI study.](https://neurosynth.org/studies/25778615/) | Goozee R, Reinders AATS, Handley R, Marques T, Taylor H, O'Daly O, McQueen G, Hubbard K, Mondelli V, Pariante C, Dazzan P | Schizophrenia research |
| 253 | [Effects of childhood trauma on working memory in affective and non-affective psychotic disorders.](https://neurosynth.org/studies/27090803/) | Quide Y, O'Reilly N, Rowland JE, Carr VJ, Elzinga BM, Green MJ | Brain imaging and behavior |
| 254 | [Effects of endurance training on brain structures in chronic schizophrenia patients and healthy controls.](https://neurosynth.org/studies/25623601/) | Malchow B, Keeser D, Keller K, Hasan A, Rauchmann BS, Kimura H, Schneider-Axmann T, Dechent P, Gruber O, Ertl-Wagner B, Honer WG, Hillmer-Vogel U, Schmitt A, Wobrock T, Niklas A, Falkai P | Schizophrenia research |
| 255 | [Effects of ketamine on prefrontal and striatal regions in an overt verbal fluency task: a functional magnetic resonance imaging study.](https://neurosynth.org/studies/16228196/) | Fu CH, Abel KM, Allin MP, Gasston D, Costafreda SG, Suckling J, Williams SC, McGuire PK | Psychopharmacology |
| 256 | [Effects of ketamine-induced psychopathological symptoms on continuous overt rhyme fluency.](https://neurosynth.org/studies/22189657/) | Nagels A, Kirner-Veselinovic A, Wiese R, Paulus FM, Kircher T, Krach S | European archives of psychiatry and clinical neuroscience |
| 257 | [Effects of minocycline add-on treatment on brain morphometry and cerebral perfusion in recent-onset schizophrenia.](https://neurosynth.org/studies/25497439/) | Chaves C, Marque CR, Maia-de-Oliveira JP, Wichert-Ana L, Ferrari TB, Santos AC, Araujo D, Machado-de-Sousa JP, Bressan RA, Elkis H, Crippa JA, Guimaraes FS, Zuardi AW, Baker GB, Dursun SM, Hallak JE | Schizophrenia research |
| 258 | [Effects of MIR137 on fronto-amygdala functional connectivity.](https://neurosynth.org/studies/24361663/) | Mothersill O, Morris DW, Kelly S, Rose EJ, Fahey C, O'Brien C, Lyne R, Reilly R, Gill M, Corvin AP, Donohoe G | NeuroImage |
| 259 | [Effects of treatment with the atypical neuroleptic quetiapine on working memory function: a functional MRI follow-up investigation.](https://neurosynth.org/studies/17151834/) | Meisenzahl EM, Scheuerecker J, Zipse M, Ufer S, Wiesmann M, Frodl T, Koutsouleris N, Zetzsche T, Schmitt G, Riedel M, Spellmann I, Dehning S, Linn J, Bruckmann H, Moller HJ | European archives of psychiatry and clinical neuroscience |
| 260 | [Efficacy of identifying neural components in the face and emotion processing system in schizophrenia using a dynamic functional localizer.](https://neurosynth.org/studies/26792586/) | Arnold AE, Iaria G, Goghari VM | Psychiatry research. Neuroimaging |
| 261 | [Elaborative verbal encoding and altered anterior parahippocampal activation in adolescents and young adults at genetic risk for schizophrenia using FMRI.](https://neurosynth.org/studies/17276751/) | Thermenos HW, Seidman LJ, Poldrack RA, Peace NK, Koch JK, Faraone SV, Tsuang MT | Biological psychiatry |
| 262 | [Emotional decision-making and its dissociable components in schizophrenia and schizoaffective disorder: a behavioural and MRI investigation.](https://neurosynth.org/studies/18329673/) | Premkumar P, Fannon D, Kuipers E, Simmons A, Frangou S, Kumari V | Neuropsychologia |
| 263 | [Emotional words induce enhanced brain activity in schizophrenic patients with auditory hallucinations.](https://neurosynth.org/studies/17184978/) | Sanjuan J, Lull JJ, Aguilar EJ, Marti-Bonmati L, Moratal D, Gonzalez JC, Robles M, Keshavan MS | Psychiatry research |
| 264 | [Endogenous testosterone levels are associated with neural activity in men with schizophrenia during facial emotion processing.](https://neurosynth.org/studies/25796490/) | Ji E, Weickert CS, Lenroot R, Catts SV, Vercammen A, White C, Gur RE, Weickert TW | Behavioural brain research |
| 265 | [Enhanced cortical effects of auditory stimulation and auditory attention in healthy individuals prone to auditory hallucinations during partial wakefulness.](https://neurosynth.org/studies/21571075/) | Lewis-Hanna LL, Hunter MD, Farrow TF, Wilkinson ID, Woodruff PW | NeuroImage |
| 266 | [Enhanced disease characterization through multi network functional normalization in fMRI.](https://neurosynth.org/studies/25873853/) | Cetin MS, Khullar S, Damaraju E, Michael AM, Baum SA, Calhoun VD | Frontiers in neuroscience |
| 267 | [Enhanced left frontal involvement during novel metaphor comprehension in schizophrenia: evidence from functional neuroimaging.](https://neurosynth.org/studies/23291493/) | Mashal N, Vishne T, Laor N, Titone D | Brain and language |
| 268 | [Episodic memory in schizophrenia: the influence of strategy use on behavior and brain activation.](https://neurosynth.org/studies/18790618/) | Bonner-Jackson A, Yodkovik N, Csernansky JG, Barch DM | Psychiatry research |
| 269 | [Epistasis between the DAT 3' UTR VNTR and the COMT Val158Met SNP on cortical function in healthy subjects and patients with schizophrenia.](https://neurosynth.org/studies/19666577/) | Prata DP, Mechelli A, Fu CH, Picchioni M, Toulopoulou T, Bramon E, Walshe M, Murray RM, Collier DA, McGuire P | Proceedings of the National Academy of Sciences of the United States of America |
| 270 | [ERBB4 polymorphism and family history of psychiatric disorders on age-related cortical changes in healthy children.](https://neurosynth.org/studies/25744101/) | Douet V, Chang L, Lee K, Ernst T | Brain imaging and behavior |
| 271 | [Error detection failures in schizophrenia: ERPs and FMRI.](https://neurosynth.org/studies/19414043/) | Mathalon DH, Jorgensen KW, Roach BJ, Ford JM | International journal of psychophysiology : official journal of the International Organization of Psychophysiology |
| 272 | [Error processing network dynamics in schizophrenia.](https://neurosynth.org/studies/20883800/) | Becerril KE, Repovs G, Barch DM | NeuroImage |
| 273 | [Erythropoietin reduces neural and cognitive processing of fear in human models of antidepressant drug action.](https://neurosynth.org/studies/17553466/) | Miskowiak K, O'Sullivan U, Harmer CJ | Biological psychiatry |
| 274 | [Evaluation of a novel event-related parametric fMRI paradigm investigating prefrontal function.](https://neurosynth.org/studies/16139997/) | Wolf RC, Walter H | Psychiatry research |
| 275 | [Evidence for altered amygdala activation in schizophrenia in an adaptive emotion recognition task.](https://neurosynth.org/studies/24434194/) | Mier D, Lis S, Zygrodnik K, Sauer C, Ulferts J, Gallhofer B, Kirsch P | Psychiatry research |
| 76 | [Evidence for anomalous network connectivity during working memory encoding in schizophrenia: an ICA based analysis.](https://neurosynth.org/studies/19936244/) | Meda SA, Stevens MC, Folley BS, Calhoun VD, Pearlson GD | PloS one |
| 277 | [Evidence for glutamatergic neurotransmission in cognitive control in an auditory attention task.](https://neurosynth.org/studies/19429078/) | van Wageningen H, Jorgensen HA, Specht K, Hugdahl K | Neuroscience letters |
| 278 | [Evidence for progressive brain abnormalities in early schizophrenia: a cross-sectional structural and functional connectivity study.](https://neurosynth.org/studies/25176348/) | Zhang F, Qiu L, Yuan L, Ma H, Ye R, Yu F, Hu P, Dong Y, Wang K | Schizophrenia research |
| 279 | [Evidence of altered cortical and amygdala activation during social decision-making in schizophrenia.](https://neurosynth.org/studies/18261933/) | Baas D, Aleman A, Vink M, Ramsey NF, de Haan EH, Kahn RS | NeuroImage |
| 280 | [Evidence of altered prefrontal-thalamic circuitry in schizophrenia: an optimized diffusion MRI study.](https://neurosynth.org/studies/16626974/) | Rose SE, Chalk JB, Janke AL, Strudwick MW, Windus LC, Hannah DE, McGrath JJ, Pantelis C, Wood SJ, Mowry BJ | NeuroImage |
| 281 | [Evidence of gray matter reduction and dysfunction in chromosome 22q11.2 deletion syndrome.](https://neurosynth.org/studies/19962860/) | Shashi V, Kwapil TR, Kaczorowski J, Berry MN, Santos CS, Howard TD, Goradia D, Prasad K, Vaibhav D, Rajarethinam R, Spence E, Keshavan MS | Psychiatry research |
| 282 | [Evidence-based guidelines on the therapeutic use of repetitive transcranial magnetic stimulation (rTMS).](https://neurosynth.org/studies/25034472/) | Lefaucheur JP, Andre-Obadia N, Antal A, Ayache SS, Baeken C, Benninger DH, Cantello RM, Cincotta M, de Carvalho M, De Ridder D, Devanne H, Di Lazzaro V, Filipovic SR, Hummel FC, Jaaskelainen SK, Kimiskidis VK, Koch G, Langguth B, Nyffeler T, Oliviero A, Padberg F, Poulet E, Rossi S, Rossini PM, Rothwell JC, Schonfeldt-Lecuona C, Siebner HR, Slotema CW, Stagg CJ, Valls-Sole J, Ziemann U, Paulus W, Garcia-Larrea L | Clinical neurophysiology : official journal of the International Federation of Clinical Neurophysiology |
| 283 | [Exaggerated brain activation during emotion processing in unaffected siblings of patients with schizophrenia.](https://neurosynth.org/studies/21531384/) | van Buuren M, Vink M, Rapcencu AE, Kahn RS | Biological psychiatry |
| 284 | [Excessive contralateral motor overflow in schizophrenia measured by fMRI.](https://neurosynth.org/studies/22608155/) | Minzenberg MJ, Yoon JH, Soosman SK, Carter CS | Psychiatry research |
| 285 | [Expanding the response space in chronic schizophrenia: the relevance of left prefrontal cortex.](https://neurosynth.org/studies/15808995/) | Ganesan V, Green RD, Hunter MD, Wilkinson ID, Spence SA | NeuroImage |
| 286 | [Expected value and prediction error abnormalities in depression and schizophrenia.](https://neurosynth.org/studies/21482548/) | Gradin VB, Kumar P, Waiter G, Ahearn T, Stickle C, Milders M, Reid I, Hall J, Steele JD | Brain : a journal of neurology |
| 287 | [Exploring the neural correlates of delusions of reference.](https://neurosynth.org/studies/21831358/) | Menon M, Schmitz TW, Anderson AK, Graff A, Korostil M, Mamo D, Gerretsen P, Addington J, Remington G, Kapur S | Biological psychiatry |
| 288 | [Facial emotion processing in patients with schizophrenia and their non-psychotic siblings: a functional magnetic resonance imaging study.](https://neurosynth.org/studies/22113155/) | Li HJ, Chan RC, Gong QY, Liu Y, Liu SM, Shum D, Ma ZL | Schizophrenia research |
| 289 | [Factors in sensory processing of prosody in schizotypal personality disorder: an fMRI experiment.](https://neurosynth.org/studies/20362418/) | Dickey CC, Morocz IA, Minney D, Niznikiewicz MA, Voglmaier MM, Panych LP, Khan U, Zacks R, Terry DP, Shenton ME, McCarley RW | Schizophrenia research |
| 290 | [fMRI correlates of state and trait effects in subjects at genetically enhanced risk of schizophrenia.](https://neurosynth.org/studies/14749289/) | Whalley HC, Simonotto E, Flett S, Marshall I, Ebmeier KP, Owens DG, Goddard NH, Johnstone EC, Lawrie SM | Brain : a journal of neurology |
| 291 | [fMRI responses to emotional faces in children and adolescents at genetic risk for psychiatric illness share some of the features of depression.](https://neurosynth.org/studies/22222174/) | Barbour T, Pruitt P, Diwadkar VA | Journal of affective disorders |
| 292 | [Frequency dependent alterations in regional homogeneity of baseline brain activity in schizophrenia.](https://neurosynth.org/studies/23483911/) | Yu R, Hsieh MH, Wang HL, Liu CM, Liu CC, Hwang TJ, Chien YL, Hwu HG, Tseng WY | PloS one |
| 293 | [Frequency-specific alternations in the amplitude of low-frequency fluctuations in schizophrenia.](https://neurosynth.org/studies/23125131/) | Yu R, Chien YL, Wang HL, Liu CM, Liu CC, Hwang TJ, Hsieh MH, Hwu HG, Tseng WY | Human brain mapping |
| 294 | [Frontal-striatal-thalamic mediodorsal nucleus dysfunction in schizophrenia-spectrum patients during sensorimotor gating.](https://neurosynth.org/studies/18588988/) | Hazlett EA, Buchsbaum MS, Zhang J, Newmark RE, Glanton CF, Zelmanova Y, Haznedar MM, Chu KW, Nenadic I, Kemether EM, Tang CY, New AS, Siever LJ | NeuroImage |
| 295 | [Fronto-hippocampal function during temporal context monitoring in schizophrenia.](https://neurosynth.org/studies/17020747/) | Weiss AP, Goff D, Schacter DL, Ditman T, Freudenreich O, Henderson D, Heckers S | Biological psychiatry |
| 296 | [Fronto-limbic and autonomic disjunctions to negative emotion distinguish schizophrenia subtypes.](https://neurosynth.org/studies/17398080/) | Williams LM, Das P, Liddell BJ, Olivieri G, Peduto AS, David AS, Gordon E, Harris AW | Psychiatry research |
| 297 | [Fronto-Limbic Brain Dysfunction during the Regulation of Emotion in Schizophrenia.](https://neurosynth.org/studies/26930284/) | Eack SM, Wojtalik JA, Barb SM, Newhill CE, Keshavan MS, Phillips ML | PloS one |
| 298 | [Fronto-parietal hypo-activation during working memory independent of structural abnormalities: conjoint fMRI and sMRI analyses in adolescent offspring of schizophrenia patients.](https://neurosynth.org/studies/21729757/) | Diwadkar VA, Pruitt P, Goradia D, Murphy E, Bakshi N, Keshavan MS, Rajan U, Reid A, Zajac-Benitez C | NeuroImage |
| 299 | [Frontostriatal involvement in task switching depends on genetic differences in d2 receptor density.](https://neurosynth.org/studies/20962241/) | Stelzel C, Basten U, Montag C, Reuter M, Fiebach CJ | The Journal of neuroscience : the official journal of the Society for Neuroscience |
| 300 | [Frontotemporoparietal asymmetry and lack of illness awareness in schizophrenia.](https://neurosynth.org/studies/22213454/) | Gerretsen P, Chakravarty MM, Mamo D, Menon M, Pollock BG, Rajji TK, Graff-Guerrero A | Human brain mapping |
| 301 | [Functional abnormalities of the right posterior insula are related to the altered self-experience in schizophrenia.](https://neurosynth.org/studies/27662482/) | Chen X, Duan M, He H, Yang M, Klugah-Brown B, Xu H, Lai Y, Luo C, Yao D | Psychiatry research. Neuroimaging |
| 302 | [Functional activation abnormalities during facial emotion perception in schizophrenia patients and nonpsychotic relatives.](https://neurosynth.org/studies/26189076/) | Spilka MJ, Arnold AE, Goghari VM | Schizophrenia research |
| 303 | [Functional and anatomical connectivity abnormalities in left inferior frontal gyrus in schizophrenia.](https://neurosynth.org/studies/19569073/) | Jeong B, Wible CG, Hashimoto R, Kubicki M | Human brain mapping |
| 304 | [Functional and structural brain correlates of theory of mind and empathy deficits in schizophrenia.](https://neurosynth.org/studies/19632816/) | Benedetti F, Bernasconi A, Bosia M, Cavallaro R, Dallaspezia S, Falini A, Poletti S, Radaelli D, Riccaboni R, Scotti G, Smeraldi E | Schizophrenia research |
| 305 | [Functional and white matter abnormalities in the language network in patients with schizophrenia: a combined study with diffusion tensor imaging and functional magnetic resonance imaging.](https://neurosynth.org/studies/23916391/) | Leroux E, Delcroix N, Alary M, Razafimandimby A, Brazo P, Delamillieure P, Dollfus S | Schizophrenia research |
| 306 | [Functional Connectivity Anomalies in Adolescents with Psychotic Symptoms.](https://neurosynth.org/studies/28125578/) | Amico F, O'Hanlon E, Kraft D, Oertel-Knochel V, Clarke M, Kelleher I, Higgins N, Coughlan H, Creegan D, Heneghan M, Power E, Power L, Ryan J, Frodl T, Cannon M | PloS one |
| 307 | [Functional connectivity density alterations in schizophrenia.](https://neurosynth.org/studies/25477799/) | Zhuo C, Zhu J, Qin W, Qu H, Ma X, Tian H, Xu Q, Yu C | Frontiers in behavioral neuroscience |
| 308 | [Functional connectivity of left Heschl's gyrus in vulnerability to auditory hallucinations in schizophrenia.](https://neurosynth.org/studies/23287311/) | Shinn AK, Baker JT, Cohen BM, Ongur D | Schizophrenia research |
| 309 | [Functional deficit of the medial prefrontal cortex during emotional sentence attribution in schizophrenia.](https://neurosynth.org/studies/27613508/) | Razafimandimby A, Herve PY, Marzloff V, Brazo P, Tzourio-Mazoyer N, Dollfus S | Schizophrenia research |
| 310 | [Functional disconnections in the direct and indirect amygdala pathways for fear processing in schizophrenia.](https://neurosynth.org/studies/17222539/) | Das P, Kemp AH, Flynn G, Harris AW, Liddell BJ, Whitford TJ, Peduto A, Gordon E, Williams LM | Schizophrenia research |
| 311 | [Functional disconnectivity in subjects at high genetic risk of schizophrenia.](https://neurosynth.org/studies/15930046/) | Whalley HC, Simonotto E, Marshall I, Owens DG, Goddard NH, Johnstone EC, Lawrie SM | Brain : a journal of neurology |
| 312 | [Functional disintegration in paranoid schizophrenia using resting-state fMRI.](https://neurosynth.org/studies/17628434/) | Zhou Y, Liang M, Tian L, Wang K, Hao Y, Liu H, Liu Z, Jiang T | Schizophrenia research |
| 313 | [Functional dysconnectivity in schizophrenia associated with attentional modulation of motor function.](https://neurosynth.org/studies/16183659/) | Honey GD, Pomarol-Clotet E, Corlett PR, Honey RA, McKenna PJ, Bullmore ET, Fletcher PC | Brain : a journal of neurology |
| 314 | [Functional imaging as a predictor of schizophrenia.](https://neurosynth.org/studies/16460690/) | Whalley HC, Simonotto E, Moorhead W, McIntosh A, Marshall I, Ebmeier KP, Owens DG, Goddard NH, Johnstone EC, Lawrie SM | Biological psychiatry |
| 315 | [Functional imaging evidence of the relationship between recurrent psychotic episodes and neurodegenerative course in schizophrenia.](https://neurosynth.org/studies/16054343/) | Seok Jeong B, Kwon JS, Yoon Kim S, Lee C, Youn T, Moon CH, Yoon Kim C | Psychiatry research |
| 316 | [Functional integration in schizophrenia: too little or too much? Preliminary results on fMRI data.](https://neurosynth.org/studies/15907297/) | Foucher JR, Vidailhet P, Chanraud S, Gounot D, Grucker D, Pins D, Damsa C, Danion JM | NeuroImage |
| 317 | [Functional lateralization of the sensorimotor cortex in patients with schizophrenia: effects of treatment with olanzapine.](https://neurosynth.org/studies/15271588/) | Bertolino A, Blasi G, Caforio G, Latorre V, De Candia M, Rubino V, Callicott JH, Mattay VS, Bellomo A, Scarabino T, Weinberger DR, Nardini M | Biological psychiatry |
| 318 | [Functional magnetic resonance imaging during auditory verbal working memory in nonpsychotic relatives of persons with schizophrenia: a pilot study.](https://neurosynth.org/studies/15023577/) | Thermenos HW, Seidman LJ, Breiter H, Goldstein JM, Goodman JM, Poldrack R, Faraone SV, Tsuang MT | Biological psychiatry |
| 319 | [Functional magnetic resonance imaging in schizophrenia: cortical response to motor stimulation.](https://neurosynth.org/studies/15135157/) | Rogowska J, Gruber SA, Yurgelun-Todd DA | Psychiatry research |
| 320 | [Functional magnetic resonance imaging of BDNF val66met polymorphism in unmedicated subjects at high genetic risk of schizophrenia performing a verbal memory task.](https://neurosynth.org/studies/20708907/) | Baig BJ, Whalley HC, Hall J, McIntosh AM, Job DE, Cunningham-Owens DG, Johnstone EC, Lawrie SM | Psychiatry research |
| 321 | [Functional magnetic resonance imaging of inner speech in schizophrenia.](https://neurosynth.org/studies/19846064/) | Simons CJ, Tracy DK, Sanghera KK, O'Daly O, Gilleen J, Dominguez MD, Krabbendam L, Shergill SS | Biological psychiatry |
| 322 | [Functional magnetic resonance imaging of internal source monitoring in schizophrenia: recognition with and without recollection.](https://neurosynth.org/studies/16814525/) | Ragland JD, Valdez JN, Loughead J, Gur RC, Gur RE | Schizophrenia research |
| 323 | [Functional magnetic resonance imaging response to experimental pain in drug-free patients with schizophrenia.](https://neurosynth.org/studies/20609569/) | de la Fuente-Sandoval C, Favila R, Gomez-Martin D, Pellicer F, Graff-Guerrero A | Psychiatry research |
| 324 | [Functional magnetic resonance imaging studies of eye movements in first episode schizophrenia: smooth pursuit, visually guided saccades and the oculomotor delayed response task.](https://neurosynth.org/studies/16571373/) | Keedy SK, Ebens CL, Keshavan MS, Sweeney JA | Psychiatry research |
| 325 | [Functional magnetic resonance imaging study of cognitive control in the healthy relatives of schizophrenia patients.](https://neurosynth.org/studies/16945345/) | MacDonald AW 3rd, Becker TM, Carter CS | Biological psychiatry |
| 326 | [Functional mapping of dynamic happy and fearful facial expressions in young adults with familial risk for psychosis - Oulu Brain and Mind Study.](https://neurosynth.org/studies/25703807/) | Pulkkinen J, Nikkinen J, Kiviniemi V, Maki P, Miettunen J, Koivukangas J, Mukkala S, Nordstrom T, Barnett JH, Jones PB, Moilanen I, Murray GK, Veijola J | Schizophrenia research |
| 327 | [Functional MRI BOLD response to Tower of London performance of first-episode schizophrenia patients using cortical pattern matching.](https://neurosynth.org/studies/15955504/) | Rasser PE, Johnston P, Lagopoulos J, Ward PB, Schall U, Thienel R, Bender S, Toga AW, Thompson PM | NeuroImage |
| 328 | [Functional MRI mapping of brain activation during visually guided saccades and antisaccades: cortical and subcortical networks.](https://neurosynth.org/studies/15313521/) | Matsuda T, Matsuura M, Ohkubo T, Ohkubo H, Matsushima E, Inoue K, Taira M, Kojima T | Psychiatry research |
| 329 | [Functional MRI of facial emotion recognition deficits in schizophrenia and their electrophysiological correlates.](https://neurosynth.org/studies/16176365/) | Johnston PJ, Stojanov W, Devir H, Schall U | The European journal of neuroscience |
| 330 | [Functional neural networks of time perception: challenge and opportunity for schizophrenia research.](https://neurosynth.org/studies/21041067/) | Ortuno F, Guillen-Grima F, Lopez-Garcia P, Gomez J, Pla J | Schizophrenia research |
| 331 | [Functional neuroanatomical correlates of episodic memory impairment in early phase psychosis.](https://neurosynth.org/studies/25749917/) | Francis MM, Hummer TA, Vohs JL, Yung MG, Liffick E, Mehdiyoun NF, Radnovich AJ, McDonald BC, Saykin AJ, Breier A | Brain imaging and behavior |
| 332 | [Functional resting-state networks are differentially affected in schizophrenia.](https://neurosynth.org/studies/21458238/) | Woodward ND, Rogers B, Heckers S | Schizophrenia research |
| 333 | [Functional similarity of facial emotion processing between people with a first episode of psychosis and healthy subjects.](https://neurosynth.org/studies/23830857/) | Villalta-Gil V, Melendez-Perez I, Russell T, Surguladze S, Radua J, Fuste M, Stephan-Otto C, Haro JM | Schizophrenia research |
| 334 | [Further evidence of alerted default network connectivity and association with theory of mind ability in schizophrenia.](https://neurosynth.org/studies/27913157/) | Mothersill O, Tangney N, Morris DW, McCarthy H, Frodl T, Gill M, Corvin A, Donohoe G | Schizophrenia research |
| 335 | [Genes and memory: the neuroanatomical correlates of emotional memory in monozygotic twin discordant for schizophrenia.](https://neurosynth.org/studies/15177789/) | Fahim C, Stip E, Mancini-Marie A, Beauregard M | Brain and cognition |
| 336 | [Genetic associations of brain structural networks in schizophrenia: a preliminary study.](https://neurosynth.org/studies/20691427/) | Jagannathan K, Calhoun VD, Gelernter J, Stevens MC, Liu J, Bolognani F, Windemuth A, Ruano G, Assaf M, Pearlson GD | Biological psychiatry |
| 337 | [Genetic influences of cortical gray matter in language-related regions in healthy controls and schizophrenia.](https://neurosynth.org/studies/21507613/) | Jamadar S, Powers NR, Meda SA, Gelernter J, Gruen JR, Pearlson GD | Schizophrenia research |
| 338 | [Genetic influences of resting state fMRI activity in language-related brain regions in healthy controls and schizophrenia patients: a pilot study.](https://neurosynth.org/studies/22669497/) | Jamadar S, Powers NR, Meda SA, Calhoun VD, Gelernter J, Gruen JR, Pearlson GD | Brain imaging and behavior |
| 339 | [Genetic variation in G72 correlates with brain activation in the right middle temporal gyrus in a verbal fluency task in healthy individuals.](https://neurosynth.org/studies/20336655/) | Krug A, Markov V, Krach S, Jansen A, Zerres K, Eggermann T, Stocker T, Shah NJ, Nothen MM, Georgi A, Strohmaier J, Rietschel M, Kircher T | Human brain mapping |
| 340 | [Genetic variation in neuregulin1 is associated with differences in prefrontal engagement in children.](https://neurosynth.org/studies/19449332/) | Mechelli A, Viding E, Pettersson-Yeo W, Tognin S, McGuire PK | Human brain mapping |
| 341 | [Genetic variation in schizophrenia-risk-gene dysbindin 1 modulates brain activation in anterior cingulate cortex and right temporal gyrus during language production in healthy individuals.](https://neurosynth.org/studies/19497374/) | Markov V, Krug A, Krach S, Whitney C, Eggermann T, Zerres K, Stocker T, Shah NJ, Nothen MM, Treutlein J, Rietschel M, Kircher T | NeuroImage |
| 342 | [Genetic variation in the schizophrenia-risk gene neuregulin 1 correlates with brain activation and impaired speech production in a verbal fluency task in healthy individuals.](https://neurosynth.org/studies/19350564/) | Kircher T, Krug A, Markov V, Whitney C, Krach S, Zerres K, Eggermann T, Stocker T, Shah NJ, Treutlein J, Nothen MM, Becker T, Rietschel M | Human brain mapping |
| 343 | [Global prefrontal and fronto-amygdala dysconnectivity in bipolar I disorder with psychosis history.](https://neurosynth.org/studies/22980587/) | Anticevic A, Brumbaugh MS, Winkler AM, Lombardo LE, Barrett J, Corlett PR, Kober H, Gruber J, Repovs G, Cole MW, Krystal JH, Pearlson GD, Glahn DC | Biological psychiatry |
| 344 | [Gray matter abnormalities in subjects at ultra-high risk for schizophrenia and first-episode schizophrenic patients compared to healthy controls.](https://neurosynth.org/studies/19616415/) | Witthaus H, Kaufmann C, Bohner G, Ozgurdal S, Gudlowski Y, Gallinat J, Ruhrmann S, Brune M, Heinz A, Klingebiel R, Juckel G | Psychiatry research |
| 345 | [Gray matter volume differences specific to formal thought disorder in schizophrenia.](https://neurosynth.org/studies/20418073/) | Horn H, Federspiel A, Wirth M, Muller TJ, Wiest R, Walther S, Strik W | Psychiatry research |
| 346 | [Grey matter changes associated with host genetic variation and exposure to Herpes Simplex Virus 1 (HSV1) in first episode schizophrenia.](https://neurosynth.org/studies/20138739/) | Prasad KM, Bamne MN, Shirts BH, Goradia D, Mannali V, Pancholi KM, Xue B, McClain L, Yolken RH, Keshavan MS, Nimgaonkar VL | Schizophrenia research |
| 347 | [Grey matter changes over time in high risk subjects developing schizophrenia.](https://neurosynth.org/studies/15850721/) | Job DE, Whalley HC, Johnstone EC, Lawrie SM | NeuroImage |
| 348 | [Grey matter correlates of early psychotic symptoms in adolescents at enhanced risk of psychosis: a voxel-based study.](https://neurosynth.org/studies/17320416/) | Spencer MD, Moorhead TW, McIntosh AM, Stanfield AC, Muir WJ, Hoare P, Owens DG, Lawrie SM, Johnstone EC | NeuroImage |
| 349 | [Grey matter deficits and symptom profile in first episode schizophrenia.](https://neurosynth.org/studies/16055311/) | Whitford TJ, Farrow TF, Gomes L, Brennan J, Harris AW, Williams LM | Psychiatry research |
| 350 | [Grey matter morphological anomalies in the caudate head in first-episode psychosis patients with delusions of reference.](https://neurosynth.org/studies/26025014/) | Tao H, Wong GH, Zhang H, Zhou Y, Xue Z, Shan B, Chen EY, Liu Z | Psychiatry research |
| 351 | [Guided exploration of genomic risk for gray matter abnormalities in schizophrenia using parallel independent component analysis with reference.](https://neurosynth.org/studies/23727316/) | Chen J, Calhoun VD, Pearlson GD, Perrone-Bizzozero N, Sui J, Turner JA, Bustillo JR, Ehrlich S, Sponheim SR, Canive JM, Ho BC, Liu J | NeuroImage |
| 352 | [High classification accuracy for schizophrenia with rest and task FMRI data.](https://neurosynth.org/studies/22675292/) | Du W, Calhoun VD, Li H, Ma S, Eichele T, Kiehl KA, Pearlson GD, Adali T | Frontiers in human neuroscience |
| 353 | [Higher or lower? The functional anatomy of perceived allocentric social hierarchies.](https://neurosynth.org/studies/21664277/) | Farrow TF, Jones SC, Kaylor-Hughes CJ, Wilkinson ID, Woodruff PW, Hunter MD, Spence SA | NeuroImage |
| 354 | [Hippocampal activation and memory performance in schizophrenia depend on strategy use in a virtual maze.](https://neurosynth.org/studies/28780430/) | Wilkins LK, Girard TA, Herdman KA, Christensen BK, King J, Kiang M, Bohbot VD | Psychiatry research. Neuroimaging |
| 355 | [Hippocampal and Frontolimbic Function as Intermediate Phenotype for Psychosis: Evidence from Healthy Relatives and a Common Risk Variant in CACNA1C.](https://neurosynth.org/studies/24411473/) | Erk S, Meyer-Lindenberg A, Schmierer P, Mohnke S, Grimm O, Garbusow M, Haddad L, Poehland L, Muhleisen TW, Witt SH, Tost H, Kirsch P, Romanczuk-Seiferth N, Schott BH, Cichon S, Nothen MM, Rietschel M, Heinz A, Walter H | Biological psychiatry |
| 356 | [Hippocampal and orbital inferior frontal gray matter volume abnormalities and cognitive deficit in treatment-naive, first-episode patients with schizophrenia.](https://neurosynth.org/studies/24439000/) | Guo X, Li J, Wang J, Fan X, Hu M, Shen Y, Chen H, Zhao J | Schizophrenia research |
| 357 | [Hippocampal dysfunction during declarative memory encoding in schizophrenia and effects of genetic liability.](https://neurosynth.org/studies/25497222/) | Pirnia T, Woods RP, Hamilton LS, Lyden H, Joshi SH, Asarnow RF, Nuechterlein KH, Narr KL | Schizophrenia research |
| 358 | [Hippocampal dysfunction during free word association in male patients with schizophrenia.](https://neurosynth.org/studies/18356025/) | Kircher T, Whitney C, Krings T, Huber W, Weis S | Schizophrenia research |
| 359 | [Hippocampal underactivation in an fMRI study of word and face memory recognition in schizophrenia.](https://neurosynth.org/studies/19224116/) | Rametti G, Junque C, Vendrell P, Catalan R, Penades R, Bargallo N, Bernardo M | European archives of psychiatry and clinical neuroscience |
| 360 | [How can cognitive remediation therapy modulate brain activations in schizophrenia? An fMRI study.](https://neurosynth.org/studies/21543191/) | Bor J, Brunelin J, d'Amato T, Costes N, Suaud-Chagny MF, Saoud M, Poulet E | Psychiatry research |
| 361 | [Human reward system activation is modulated by a single dose of olanzapine in healthy subjects in an event-related, double-blind, placebo-controlled fMRI study.](https://neurosynth.org/studies/17265148/) | Abler B, Erk S, Walter H | Psychopharmacology |
| 362 | [Hyper-coupling between working memory task-evoked activations and amplitude of spontaneous fluctuations in first-episode schizophrenia.](https://neurosynth.org/studies/25132644/) | Zhou Y, Wang Z, Zuo XN, Zhang H, Wang Y, Jiang T, Liu Z | Schizophrenia research |
| 363 | [Hyperactivation balances sensory processing deficits during mood induction in schizophrenia.](https://neurosynth.org/studies/23051903/) | Dyck M, Loughead J, Gur RC, Schneider F, Mathiak K | Social cognitive and affective neuroscience |
| 364 | [Hyperactivity of caudate, parahippocampal, and prefrontal regions during working memory in never-medicated persons at clinical high-risk for psychosis.](https://neurosynth.org/studies/26965745/) | Thermenos HW, Juelich RJ, DiChiara SR, Mesholam-Gately RI, Woodberry KA, Wojcik J, Makris N, Keshavan MS, Whitfield-Gabrieli S, Woo TU, Petryshen TL, Goldstein JM, Shenton ME, McCarley RW, Seidman LJ | Schizophrenia research |
| 365 | [Hyperfrontality and hypoconnectivity during refreshing in schizophrenia.](https://neurosynth.org/studies/23137808/) | Grillon ML, Oppenheim C, Varoquaux G, Charbonneau F, Devauchelle AD, Krebs MO, Bayle F, Thirion B, Huron C | Psychiatry research |
| 366 | [Hypofrontality in subjects at high genetic risk of schizophrenia with depressive symptoms.](https://neurosynth.org/studies/18164074/) | Whalley HC, Mowatt L, Stanfield AC, Hall J, Johnstone EC, Lawrie SM, McIntosh AM | Journal of affective disorders |
| 367 | [Identifying grey matter changes in schizotypy using partial least squares correlation.](https://neurosynth.org/studies/27208815/) | Wiebels K, Waldie KE, Roberts RP, Park HR | Cortex; a journal devoted to the study of the nervous system and behavior |
| 368 | [Illness denial in schizophrenia spectrum disorders: a function of left hemisphere dominance.](https://neurosynth.org/studies/25209949/) | Gerretsen P, Menon M, Chakravarty MM, Lerch JP, Mamo DC, Remington G, Pollock BG, Graff-Guerrero A | Human brain mapping |
| 369 | [Impact of schizophrenia-risk gene dysbindin 1 on brain activation in bilateral middle frontal gyrus during a working memory task in healthy individuals.](https://neurosynth.org/studies/19650139/) | Markov V, Krug A, Krach S, Jansen A, Eggermann T, Zerres K, Stocker T, Shah NJ, Nothen MM, Treutlein J, Rietschel M, Kircher T | Human brain mapping |
| 370 | [Impaired context processing as a potential marker of psychosis risk state.](https://neurosynth.org/studies/24120302/) | Niendam TA, Lesh TA, Yoon J, Westphal AJ, Hutchison N, Daniel Ragland J, Solomon M, Minzenberg M, Carter CS | Psychiatry research |
| 371 | [Impaired efficiency of functional networks underlying episodic memory-for-context in schizophrenia.](https://neurosynth.org/studies/20881136/) | Wang L, Metzak PD, Honer WG, Woodward TS | The Journal of neuroscience : the official journal of the Society for Neuroscience |
| 372 | [Impaired error-likelihood prediction in medial prefrontal cortex in schizophrenia.](https://neurosynth.org/studies/20851194/) | Krawitz A, Braver TS, Barch DM, Brown JW | NeuroImage |
| 373 | [Impaired hierarchical control within the lateral prefrontal cortex in schizophrenia.](https://neurosynth.org/studies/21481335/) | Barbalat G, Chambon V, Domenech PJ, Ody C, Koechlin E, Franck N, Farrer C | Biological psychiatry |
| 374 | [Impaired hippocampal function during the detection of novel words in schizophrenia.](https://neurosynth.org/studies/15038994/) | Weiss AP, Zalesak M, DeWitt I, Goff D, Kunkel L, Heckers S | Biological psychiatry |
| 375 | [Impaired insight into illness and cognitive insight in schizophrenia spectrum disorders: resting state functional connectivity.](https://neurosynth.org/studies/25458571/) | Gerretsen P, Menon M, Mamo DC, Fervaha G, Remington G, Pollock BG, Graff-Guerrero A | Schizophrenia research |
| 376 | [Impaired prefrontal-basal ganglia functional connectivity and substantia nigra hyperactivity in schizophrenia.](https://neurosynth.org/studies/23290498/) | Yoon JH, Minzenberg MJ, Raouf S, D'Esposito M, Carter CS | Biological psychiatry |
| 377 | [Impaired target detection in schizophrenia and the ventral attentional network: Findings from a joint event-related potential-functional MRI analysis.](https://neurosynth.org/studies/26448909/) | Wynn JK, Jimenez AM, Roach BJ, Korb A, Lee J, Horan WP, Ford JM, Green MF | NeuroImage. Clinical |
| 378 | [Impaired top-down processes in schizophrenia: a DCM study of ERPs.](https://neurosynth.org/studies/20056155/) | Dima D, Dietrich DE, Dillo W, Emrich HM | NeuroImage |
| 379 | [Impairment in basal limbic function in schizophrenia during affect recognition.](https://neurosynth.org/studies/12714175/) | Hempel A, Hempel E, Schonknecht P, Stippich C, Schroder J | Psychiatry research |
| 380 | [Impairment in semantic retrieval is associated with symptoms in schizophrenia but not bipolar disorder.](https://neurosynth.org/studies/22985694/) | Jamadar S, O'Neil KM, Pearlson GD, Ansari M, Gill A, Jagannathan K, Assaf M | Biological psychiatry |
| 381 | [Impulsivity-related brain volume deficits in schizophrenia-addiction comorbidity.](https://neurosynth.org/studies/20647266/) | Schiffer B, Muller BW, Scherbaum N, Forsting M, Wiltfang J, Leygraf N, Gizewski ER | Brain : a journal of neurology |
| 382 | [In Search of Multimodal Neuroimaging Biomarkers of Cognitive Deficits in Schizophrenia.](https://neurosynth.org/studies/25847180/) | Sui J, Pearlson GD, Du Y, Yu Q, Jones TR, Chen J, Jiang T, Bustillo J, Calhoun VD | Biological psychiatry |
| 383 | [Increased activation in Broca's area after cognitive remediation in schizophrenia.](https://neurosynth.org/studies/24507118/) | Vianin P, Urben S, Magistretti P, Marquet P, Fornari E, Jaugey L | Psychiatry research |
| 384 | [Increased amygdala activation during automatic processing of facial emotion in schizophrenia.](https://neurosynth.org/studies/20488680/) | Rauch AV, Reker M, Ohrmann P, Pedersen A, Bauer J, Dannlowski U, Harding L, Koelkebeck K, Konrad C, Kugel H, Arolt V, Heindel W, Suslow T | Psychiatry research |
| 385 | [Increased amygdala and parahippocampal gyrus activation in schizophrenic patients with auditory hallucinations: an fMRI study using independent component analysis.](https://neurosynth.org/studies/20071145/) | Escarti MJ, de la Iglesia-Vaya M, Marti-Bonmati L, Robles M, Carbonell J, Lull JJ, Garcia-Marti G, Manjon JV, Aguilar EJ, Aleman A, Sanjuan J | Schizophrenia research |
| 386 | [Increased anterior cingulate and temporal lobe activity during visuospatial working memory in children and adolescents with schizophrenia.](https://neurosynth.org/studies/21211946/) | White T, Hongwanishkul D, Schmidt M | Schizophrenia research |
| 387 | [Increased inferior frontal activation during word generation: a marker of genetic risk for schizophrenia but not bipolar disorder?](https://neurosynth.org/studies/19479729/) | Costafreda SG, Fu CH, Picchioni M, Kane F, McDonald C, Prata DP, Kalidindi S, Walshe M, Curtis V, Bramon E, Kravariti E, Marshall N, Toulopoulou T, Barker GJ, David AS, Brammer MJ, Murray RM, McGuire PK | Human brain mapping |
| 388 | [Increased medial temporal lobe activation during the passive viewing of emotional and neutral facial expressions in schizophrenia.](https://neurosynth.org/studies/16377154/) | Holt DJ, Kunkel L, Weiss AP, Goff DC, Wright CI, Shin LM, Rauch SL, Hootnick J, Heckers S | Schizophrenia research |
| 389 | [Increased neural response related to neutral faces in individuals at risk for psychosis.](https://neurosynth.org/studies/18187342/) | Seiferth NY, Pauly K, Habel U, Kellermann T, Shah NJ, Ruhrmann S, Klosterkotter J, Schneider F, Kircher T | NeuroImage |
| 390 | [Increased resting-state global functional connectivity density of default mode network in schizophrenia subjects treated with electroconvulsive therapy.](https://neurosynth.org/studies/29117910/) | Huang H, Jiang Y, Xia M, Tang Y, Zhang T, Cui H, Wang J, Li Y, Xu L, Curtin A, Sheng J, Jia Y, Yao D, Li C, Luo C, Wang J | Schizophrenia research |
| 391 | [Increased short-range and long-range functional connectivity in first-episode, medication-naive schizophrenia at rest.](https://neurosynth.org/studies/25982002/) | Guo W, Liu F, Xiao C, Liu J, Yu M, Zhang Z, Zhang J, Zhao J | Schizophrenia research |
| 392 | [Increased superior frontal gyrus activation during working memory processing in psychosis: Significant relation to cumulative antipsychotic medication and to negative symptoms.](https://neurosynth.org/studies/27102424/) | Vogel T, Smieskova R, Schmidt A, Walter A, Harrisberger F, Eckert A, Lang UE, Riecher-Rossler A, Graf M, Borgwardt S | Schizophrenia research |
| 393 | [Increased ventro-medial prefrontal activations in schizophrenia smokers during cigarette cravings.](https://neurosynth.org/studies/27005897/) | Potvin S, Lungu O, Lipp O, Lalonde P, Zaharieva V, Stip E, Melun JP, Mendrek A | Schizophrenia research |
| 394 | [Increased water diffusivity in the frontal and temporal cortices of schizophrenic patients.](https://neurosynth.org/studies/16406258/) | Shin YW, Kwon JS, Ha TH, Park HJ, Kim DJ, Hong SB, Moon WJ, Lee JM, Kim IY, Kim SI, Chung EC | NeuroImage |
| 395 | [Individual differences in psychotic effects of ketamine are predicted by brain function measured under placebo.](https://neurosynth.org/studies/18562599/) | Honey GD, Corlett PR, Absalom AR, Lee M, Pomarol-Clotet E, Murray GK, McKenna PJ, Bullmore ET, Menon DK, Fletcher PC | The Journal of neuroscience : the official journal of the Society for Neuroscience |
| 396 | [Individuals with schizophrenia present hypo- and hyperactivation during implicit cueing in an inhibitory task.](https://neurosynth.org/studies/16766210/) | Arce E, Leland DS, Miller DA, Simmons AN, Winternheimer KC, Paulus MP | NeuroImage |
| 397 | [Inefficient executive cognitive control in schizophrenia is preceded by altered functional activation during information encoding: an fMRI study.](https://neurosynth.org/studies/17707869/) | Schlosser RG, Koch K, Wagner G, Nenadic I, Roebel M, Schachtzabel C, Axer M, Schultz C, Reichenbach JR, Sauer H | Neuropsychologia |
| 398 | [Inferior frontal and insular cortical thinning is related to dysfunctional brain activation/deactivation during working memory task in schizophrenic patients.](https://neurosynth.org/studies/23993992/) | Pujol N, Penades R, Rametti G, Catalan R, Vidal-Pineiro D, Palacios E, Bargallo N, Bernardo M, Junque C | Psychiatry research |
| 399 | [Insular Dysfunction Reflects Altered Between-Network Connectivity and Severity of Negative Symptoms in Schizophrenia during Psychotic Remission.](https://neurosynth.org/studies/23730284/) | Manoliu A, Riedl V, Doll A, Bauml JG, Muhlau M, Schwerthoffer D, Scherr M, Zimmer C, Forstl H, Bauml J, Wohlschlager AM, Koch K, Sorg C | Frontiers in human neuroscience |
| 400 | [Intact hemispheric specialization for spatial and shape working memory in schizophrenia.](https://neurosynth.org/studies/16076549/) | Manoach DS, White N, Lindgren KA, Heckers S, Coleman MJ, Dubal S, Goff DC, Holzman PS | Schizophrenia research |
| 401 | [Intact relational memory and normal hippocampal structure in the early stage of psychosis.](https://neurosynth.org/studies/22055016/) | Williams LE, Avery SN, Woolard AA, Heckers S | Biological psychiatry |
| 402 | [Interaction among subsystems within default mode network diminished in schizophrenia patients: A dynamic connectivity approach.](https://neurosynth.org/studies/26654933/) | Du Y, Pearlson GD, Yu Q, He H, Lin D, Sui J, Wu L, Calhoun VD | Schizophrenia research |
| 403 | [Interaction between COMT Val(158)Met polymorphism and childhood adversity affects reward processing in adulthood.](https://neurosynth.org/studies/26879624/) | Boecker-Schlier R, Holz NE, Buchmann AF, Blomeyer D, Plichta MM, Jennen-Steinmetz C, Wolf I, Baumeister S, Treutlein J, Rietschel M, Meyer-Lindenberg A, Banaschewski T, Brandeis D, Laucht M | NeuroImage |
| 404 | [Interaction between effects of genes coding for dopamine and glutamate transmission on striatal and parahippocampal function.](https://neurosynth.org/studies/22438288/) | Pauli A, Prata DP, Mechelli A, Picchioni M, Fu CH, Chaddock CA, Kane F, Kalidindi S, McDonald C, Kravariti E, Toulopoulou T, Bramon E, Walshe M, Ehlert N, Georgiades A, Murray R, Collier DA, McGuire P | Human brain mapping |
| 405 | [Involvement of the mirror neuron system in blunted affect in schizophrenia.](https://neurosynth.org/studies/24268934/) | Lee JS, Chun JW, Yoon SY, Park HJ, Kim JJ | Schizophrenia research |
| 406 | [Is aberrant functional connectivity a psychosis endophenotype? A resting state functional magnetic resonance imaging study.](https://neurosynth.org/studies/23746539/) | Khadka S, Meda SA, Stevens MC, Glahn DC, Calhoun VD, Sweeney JA, Tamminga CA, Keshavan MS, O'Neil K, Schretlen D, Pearlson GD | Biological psychiatry |
| 407 | [Is it me? Verbal self-monitoring neural network and clinical insight in schizophrenia.](https://neurosynth.org/studies/26549744/) | Sapara A, Ffytche DH, Cooke MA, Williams SC, Kumari V | Psychiatry research |
| 408 | [Isn't it ironic? Neural correlates of irony comprehension in schizophrenia.](https://neurosynth.org/studies/24040207/) | Rapp AM, Langohr K, Mutschler DE, Klingberg S, Wild B, Erb M | PloS one |
| 409 | [Joint prediction of multiple scores captures better individual traits from brain images.](https://neurosynth.org/studies/28676298/) | Rahim M, Thirion B, Bzdok D, Buvat I, Varoquaux G | NeuroImage |
| 410 | [Joint source based morphometry identifies linked gray and white matter group differences.](https://neurosynth.org/studies/18992825/) | Xu L, Pearlson G, Calhoun VD | NeuroImage |
| 411 | [Lack of insula reactivity to aversive stimuli in schizophrenia.](https://neurosynth.org/studies/23201307/) | Linnman C, Coombs G 3rd, Goff DC, Holt DJ | Schizophrenia research |
| 412 | [Lack of self-control as assessed by a personality inventory is related to reduced volume of supplementary motor area.](https://neurosynth.org/studies/12426034/) | Matsui M, Yoneyama E, Sumiyoshi T, Noguchi K, Nohara S, Suzuki M, Kawasaki Y, Seto H, Kurachi M | Psychiatry research |
| 413 | [Language lateralization in left-handed patients with schizophrenia.](https://neurosynth.org/studies/21126527/) | Razafimandimby A, Tzourio-Mazoyer N, Mazoyer B, Maiza O, Dollfus S | Neuropsychologia |
| 414 | [Language lateralization in schizophrenia, an fMRI study.](https://neurosynth.org/studies/11595392/) | Sommer IE, Ramsey NF, Kahn RS | Schizophrenia research |
| 415 | [Language lateralization in unmedicated patients during an acute episode of schizophrenia: a functional MRI study.](https://neurosynth.org/studies/16530393/) | Weiss EM, Hofer A, Golaszewski S, Siedentopf C, Felber S, Fleischhacker WW | Psychiatry research |
| 416 | [Language network dysfunction as a predictor of outcome in youth at clinical high risk for psychosis.](https://neurosynth.org/studies/19861234/) | Sabb FW, van Erp TG, Hardt ME, Dapretto M, Caplan R, Cannon TD, Bearden CE | Schizophrenia research |
| 417 | [Learning from errors: error-related neural activity predicts improvements in future inhibitory control performance.](https://neurosynth.org/studies/19494138/) | Hester R, Madeley J, Murphy K, Mattingley JB | The Journal of neuroscience : the official journal of the Society for Neuroscience |
| 418 | [Left dorsolateral prefrontal cortex dysfunction in medication-naive schizophrenia.](https://neurosynth.org/studies/20724113/) | van Veelen NM, Vink M, Ramsey NF, Kahn RS | Schizophrenia research |
| 419 | [Left inferior prefrontal cortex activation during a semantic decision-making task predicts the degree of semantic organization.](https://neurosynth.org/studies/16009569/) | Simmons A, Miller D, Feinstein JS, Goldberg TE, Paulus MP | NeuroImage |
| 420 | [Left temporo-limbic and orbital dysfunction in schizophrenia during odor familiarity and hedonicity judgments.](https://neurosynth.org/studies/16099179/) | Plailly J, d'Amato T, Saoud M, Royet JP | NeuroImage |
| 421 | [Levels-of-processing effects in first-degree relatives of individuals with schizophrenia.](https://neurosynth.org/studies/17123479/) | Bonner-Jackson A, Csernansky JG, Barch DM | Biological psychiatry |
| 422 | [Limbic and frontal cortical degeneration is associated with psychiatric symptoms in PINK1 mutation carriers.](https://neurosynth.org/studies/18261714/) | Reetz K, Lencer R, Steinlechner S, Gaser C, Hagenah J, Buchel C, Petersen D, Kock N, Djarmati A, Siebner HR, Klein C, Binkofski F | Biological psychiatry |
| 423 | [Linked functional network abnormalities during intrinsic and extrinsic activity in schizophrenia as revealed by a data-fusion approach.](https://neurosynth.org/studies/29527474/) | Hashimoto RI, Itahashi T, Okada R, Hasegawa S, Tani M, Kato N, Mimura M | NeuroImage. Clinical |
| 424 | [Links among resting-state default-mode network, salience network, and symptomatology in schizophrenia.](https://neurosynth.org/studies/23727217/) | Orliac F, Naveau M, Joliot M, Delcroix N, Razafimandimby A, Brazo P, Dollfus S, Delamillieure P | Schizophrenia research |
| 425 | [Local brain gyrification as a marker of neurological soft signs in schizophrenia.](https://neurosynth.org/studies/26031380/) | Hirjak D, Kubera KM, Wolf RC, Thomann AK, Hell SK, Seidl U, Thomann PA | Behavioural brain research |
| 426 | [Local functional connectivity alterations in schizophrenia, bipolar disorder, and major depressive disorder.](https://neurosynth.org/studies/29751242/) | Wei Y, Chang M, Womer FY, Zhou Q, Yin Z, Wei S, Zhou Y, Jiang X, Yao X, Duan J, Xu K, Zuo XN, Tang Y, Wang F | Journal of affective disorders |
| 427 | [Longitudinal functional brain imaging study in early course schizophrenia before and after cognitive enhancement therapy.](https://neurosynth.org/studies/27894892/) | Keshavan MS, Eack SM, Prasad KM, Haller CS, Cho RY | NeuroImage |
| 428 | [Longitudinal loss of gray matter volume in patients with first-episode schizophrenia: DARTEL automated analysis and ROI validation.](https://neurosynth.org/studies/21924364/) | Asami T, Bouix S, Whitford TJ, Shenton ME, Salisbury DF, McCarley RW | NeuroImage |
| 429 | [Longitudinal regional brain volume loss in schizophrenia: Relationship to antipsychotic medication and change in social function.](https://neurosynth.org/studies/26189075/) | Guo JY, Huhtaniska S, Miettunen J, Jaaskelainen E, Kiviniemi V, Nikkinen J, Moilanen J, Haapea M, Maki P, Jones PB, Veijola J, Isohanni M, Murray GK | Schizophrenia research |
| 430 | [LORETA imaging of P300 in schizophrenia with individual MRI and 128-channel EEG.](https://neurosynth.org/studies/14642467/) | Pae JS, Kwon JS, Youn T, Park HJ, Kim MS, Lee B, Park KS | NeuroImage |
| 431 | [Lower effective connectivity between amygdala and parietal regions in response to fearful faces in schizophrenia.](https://neurosynth.org/studies/22019361/) | Mukherjee P, Whalley HC, McKirdy JW, McIntosh AM, Johnstone EC, Lawrie SM, Hall J | Schizophrenia research |
| 432 | [Magnocellular pathway impairment in schizophrenia: evidence from functional magnetic resonance imaging.](https://neurosynth.org/studies/18650327/) | Martinez A, Hillyard SA, Dias EC, Hagler DJ Jr, Butler PD, Guilfoyle DN, Jalbrzikowski M, Silipo G, Javitt DC | The Journal of neuroscience : the official journal of the Society for Neuroscience |
| 433 | [Male and female voices activate distinct regions in the male brain.](https://neurosynth.org/studies/15978839/) | Sokhi DS, Hunter MD, Wilkinson ID, Woodruff PW | NeuroImage |
| 434 | [Mapping Small-World Properties through Development in the Human Brain: Disruption in Schizophrenia.](https://neurosynth.org/studies/24788815/) | Tomasi D, Volkow ND | PloS one |
| 435 | [Me, myself and I: temporal dysfunctions during self-evaluation in patients with schizophrenia.](https://neurosynth.org/studies/24369435/) | Pauly KD, Kircher TT, Schneider F, Habel U | Social cognitive and affective neuroscience |
| 436 | [Medial frontal gyrus alterations in schizophrenia: Relationship with duration of illness and executive dysfunction.](https://neurosynth.org/studies/25498920/) | Frascarelli M, Tognin S, Mirigliani A, Parente F, Buzzanca A, Torti MC, Tinelli E, Caramia F, Di Fabio F, Biondi M, Fusar-Poli P | Psychiatry research |
| 437 | [Medial frontal hyperactivity in reality distortion.](https://neurosynth.org/studies/17434455/) | Taylor SF, Welsh RC, Chen AC, Velander AJ, Liberzon I | Biological psychiatry |
| 438 | [Medial Prefrontal and Anterior Insular Connectivity in Early Schizophrenia and Major Depressive Disorder: A Resting Functional MRI Evaluation of Large-Scale Brain Network Models.](https://neurosynth.org/studies/27064387/) | Penner J, Ford KA, Taylor R, Schaefer B, Theberge J, Neufeld RW, Osuch EA, Menon RS, Rajakumar N, Allman JM, Williamson PC | Frontiers in human neuroscience |
| 439 | [Mentalizing impairment in schizophrenia: a functional MRI study.](https://neurosynth.org/studies/21943555/) | Das P, Lagopoulos J, Coulston CM, Henderson AF, Malhi GS | Schizophrenia research |
| 440 | [Mentalizing in male schizophrenia patients is compromised by virtue of dysfunctional connectivity between task-positive and task-negative networks.](https://neurosynth.org/studies/22795367/) | Das P, Calhoun V, Malhi GS | Schizophrenia research |
| 441 | [Mentalizing in preclinical Huntington's disease: an fMRI study using cartoon picture stories.](https://neurosynth.org/studies/23179063/) | Saft C, Lissek S, Hoffmann R, Nicolas V, Tegenthoff M, Juckel G, Brune M | Brain imaging and behavior |
| 442 | [Mentalizing in schizophrenia: A multivariate functional MRI study.](https://neurosynth.org/studies/27793657/) | Martin AK, Dzafic I, Robinson GA, Reutens D, Mowry B | Neuropsychologia |
| 443 | [Meta-analysis of diffusion tensor imaging studies in schizophrenia.](https://neurosynth.org/studies/19128945/) | Ellison-Wright I, Bullmore E | Schizophrenia research |
| 444 | [Meta-analysis of functional magnetic resonance imaging studies of timing and cognitive control in schizophrenia and bipolar disorder: Evidence of a primary time deficit.](https://neurosynth.org/studies/28169089/) | Alustiza I, Radua J, Pla M, Martin R, Ortuno F | Schizophrenia research |
| 445 | [Method for multimodal analysis of independent source differences in schizophrenia: combining gray matter structural and auditory oddball functional data.](https://neurosynth.org/studies/16108017/) | Calhoun VD, Adali T, Giuliani NR, Pekar JJ, Kiehl KA, Pearlson GD | Human brain mapping |
| 446 | [Modality specific neural correlates of auditory and somatic hallucinations.](https://neurosynth.org/studies/11606687/) | Shergill SS, Cameron LA, Brammer MJ, Williams SC, Murray RM, McGuire PK | Journal of neurology, neurosurgery, and psychiatry |
| 447 | [Model order effects on ICA of resting-state complex-valued fMRI data: Application to schizophrenia.](https://neurosynth.org/studies/29673968/) | Kuang LD, Lin QH, Gong XF, Cong F, Sui J, Calhoun VD | Journal of neuroscience methods |
| 448 | [Modular Organization of Functional Network Connectivity in Healthy Controls and Patients with Schizophrenia during the Resting State.](https://neurosynth.org/studies/22275887/) | Yu Q, Plis SM, Erhardt EB, Allen EA, Sui J, Kiehl KA, Pearlson G, Calhoun VD | Frontiers in systems neuroscience |
| 449 | [Modulation of hippocampal theta and hippocampal-prefrontal cortex function by a schizophrenia risk gene.](https://neurosynth.org/studies/25757652/) | Cousijn H, Tunbridge EM, Rolinski M, Wallis G, Colclough GL, Woolrich MW, Nobre AC, Harrison PJ | Human brain mapping |
| 450 | [MTHFR 677C --> T genotype disrupts prefrontal function in schizophrenia through an interaction with COMT 158Val --> Met.](https://neurosynth.org/studies/18988738/) | Roffman JL, Gollub RL, Calhoun VD, Wassink TH, Weiss AP, Ho BC, White T, Clark VP, Fries J, Andreasen NC, Goff DC, Manoach DS | Proceedings of the National Academy of Sciences of the United States of America |
| 451 | [MTHFR 677C>T effects on anterior cingulate structure and function during response monitoring in schizophrenia: a preliminary study.](https://neurosynth.org/studies/21190096/) | Roffman JL, Brohawn DG, Friedman JS, Dyckman KA, Thakkar KN, Agam Y, Vangel MG, Goff DC, Manoach DS | Brain imaging and behavior |
| 452 | [Multi-center machine learning in imaging psychiatry: A meta-model approach.](https://neurosynth.org/studies/28428048/) | Dluhos P, Schwarz D, Cahn W, van Haren N, Kahn R, Spaniel F, Horacek J, Kasparek T, Schnack H | NeuroImage |
| 453 | [Multi-level comparison of empathy in schizophrenia: an fMRI study of a cartoon task.](https://neurosynth.org/studies/20080395/) | Lee SJ, Kang do H, Kim CW, Gu BM, Park JY, Choi CH, Shin NY, Lee JM, Kwon JS | Psychiatry research |
| 454 | [Multifaceted genomic risk for brain function in schizophrenia.](https://neurosynth.org/studies/22440650/) | Chen J, Calhoun VD, Pearlson GD, Ehrlich S, Turner JA, Ho BC, Wassink TH, Michael AM, Liu J | NeuroImage |
| 455 | [Multimodal analysis of the hippocampus in schizophrenia using proton magnetic resonance spectroscopy and functional magnetic resonance imaging.](https://neurosynth.org/studies/22831772/) | Hutcheson NL, Reid MA, White DM, Kraguljac NV, Avsar KB, Bolding MS, Knowlton RC, den Hollander JA, Lahti AC | Schizophrenia research |
| 456 | [Multiparametric mapping of neurological soft signs in healthy adults.](https://neurosynth.org/studies/25528225/) | Hirjak D, Wolf RC, Kubera KM, Stieltjes B, Thomann PA | Brain structure & function |
| 457 | [Multivariate pattern classification reveals differential brain activation during emotional processing in individuals with psychosis proneness.](https://neurosynth.org/studies/22036677/) | Modinos G, Pettersson-Yeo W, Allen P, McGuire PK, Aleman A, Mechelli A | NeuroImage |
| 458 | [Multivariate prediction of emerging psychosis in adolescents at high risk for schizophrenia.](https://neurosynth.org/studies/23010485/) | Shah J, Eack SM, Montrose DM, Tandon N, Miewald JM, Prasad KM, Keshavan MS | Schizophrenia research |
| 459 | [Music Intervention Leads to Increased Insular Connectivity and Improved Clinical Symptoms in Schizophrenia.](https://neurosynth.org/studies/29410607/) | He H, Yang M, Duan M, Chen X, Lai Y, Xia Y, Shao J, Biswal BB, Luo C, Yao D | Frontiers in neuroscience |
| 460 | [Negative symptoms in schizophrenia are associated with aberrant striato-cortical connectivity in a rewarded perceptual decision-making task.](https://neurosynth.org/studies/26106553/) | Reckless GE, Andreassen OA, Server A, Ostefjells T, Jensen J | NeuroImage. Clinical |
| 461 | [Network analysis of auditory hallucinations in nonpsychotic individuals.](https://neurosynth.org/studies/23426796/) | van Lutterveld R, Diederen KM, Otte WM, Sommer IE | Human brain mapping |
| 462 | [Network dysfunction during associative learning in schizophrenia: Increased activation, but decreased connectivity: an fMRI study.](https://neurosynth.org/studies/23759649/) | Wadehra S, Pruitt P, Murphy ER, Diwadkar VA | Schizophrenia research |
| 463 | [Neural activation abnormalities during self-referential processing in schizophrenia: An fMRI study.](https://neurosynth.org/studies/24795158/) | Liu J, Corbera S, Edward Wexler B | Psychiatry research |
| 464 | [Neural activation during successful and unsuccessful verbal learning in schizophrenia.](https://neurosynth.org/studies/16497485/) | Heinze S, Sartory G, Muller BW, de Greiff A, Forsting M, Juptner M | Schizophrenia research |
| 465 | [Neural activity changes in unaffected children of patients with schizophrenia: A resting-state fMRI study.](https://neurosynth.org/studies/26232869/) | Tang Y, Chen K, Zhou Y, Liu J, Wang Y, Driesen N, Edmiston EK, Chen X, Jiang X, Kong L, Zhou Q, Li H, Wu F, Wang Z, Xu K, Wang F | Schizophrenia research |
| 466 | [Neural activity during emotion recognition after combined cognitive plus social cognitive training in schizophrenia.](https://neurosynth.org/studies/22695257/) | Hooker CI, Bruce L, Fisher M, Verosky SC, Miyakawa A, Vinogradov S | Schizophrenia research |
| 467 | [Neural activity during object perception in schizophrenia patients is associated with illness duration and affective symptoms.](https://neurosynth.org/studies/27130563/) | Stephan-Otto C, Siddi S, Cuevas Esteban J, Senior C, Garcia-Alvarez R, Cambra-Marti MR, Usall J, Brebion G | Schizophrenia research |
| 468 | [Neural and behavioural responses to threat in men with a history of serious violence and schizophrenia or antisocial personality disorder.](https://neurosynth.org/studies/19230621/) | Kumari V, Das M, Taylor PJ, Barkataki I, Andrew C, Sumich A, Williams SC, Ffytche DH | Schizophrenia research |
| 469 | [Neural bases for impaired social cognition in schizophrenia and autism spectrum disorders.](https://neurosynth.org/studies/18053686/) | Pinkham AE, Hopfinger JB, Pelphrey KA, Piven J, Penn DL | Schizophrenia research |
| 470 | [Neural basis for inferring false beliefs and social emotions in others among individuals with schizophrenia and those at ultra-high risk for psychosis.](https://neurosynth.org/studies/27960147/) | Takano Y, Aoki Y, Yahata N, Kawakubo Y, Inoue H, Iwashiro N, Natsubori T, Koike S, Gonoi W, Sasaki H, Takao H, Kasai K, Yamasue H | Psychiatry research. Neuroimaging |
| 471 | [Neural basis of altered physical and social causality judgements in schizophrenia.](https://neurosynth.org/studies/25439393/) | Wende KC, Nagels A, Stratmann M, Chatterjee A, Kircher T, Straube B | Schizophrenia research |
| 472 | [Neural basis of attributional style in schizophrenia.](https://neurosynth.org/studies/19409961/) | Park KM, Kim JJ, Ku J, Kim SY, Lee HR, Kim SI, Yoon KJ | Neuroscience letters |
| 473 | [Neural basis of implicit memory for socio-emotional information in schizophrenia.](https://neurosynth.org/studies/23123045/) | Schwartz BL, Vaidya CJ, Shook D, Deutsch SI | Psychiatry research |
| 474 | [Neural changes following cognitive behaviour therapy for psychosis: a longitudinal study.](https://neurosynth.org/studies/21772062/) | Kumari V, Fannon D, Peters ER, Ffytche DH, Sumich AL, Premkumar P, Anilkumar AP, Andrew C, Phillips ML, Williams SC, Kuipers E | Brain : a journal of neurology |
| 475 | [Neural circuit of verbal humor comprehension in schizophrenia - an fMRI study.](https://neurosynth.org/studies/28652967/) | Adamczyk P, Wyczesany M, Domagalik A, Daren A, Cepuch K, Bladzinski P, Cechnicki A, Marek T | NeuroImage. Clinical |
| 476 | [Neural complexity as a potential translational biomarker for psychosis.](https://neurosynth.org/studies/27814962/) | Hager B, Yang AC, Brady R, Meda S, Clementz B, Pearlson GD, Sweeney JA, Tamminga C, Keshavan M | Journal of affective disorders |
| 477 | [Neural correlates of adjunctive rivastigmine treatment to antipsychotics in schizophrenia: a randomized, placebo-controlled, double-blind fMRI study.](https://neurosynth.org/studies/16181792/) | Kumari V, Aasen I, ffytche D, Williams SC, Sharma T | NeuroImage |
| 478 | [Neural correlates of emotion recognition in schizophrenia.](https://neurosynth.org/studies/20663646/) | Habel U, Chechko N, Pauly K, Koch K, Backes V, Seiferth N, Shah NJ, Stocker T, Schneider F, Kellermann T | Schizophrenia research |
| 479 | [Neural correlates of emotion regulation in patients with schizophrenia and non-affected siblings.](https://neurosynth.org/studies/24941136/) | van der Meer L, Swart M, van der Velde J, Pijnenborg G, Wiersma D, Bruggeman R, Aleman A | PloS one |
| 480 | [Neural correlates of emotional recognition memory in schizophrenia: effects of valence and arousal.](https://neurosynth.org/studies/22079660/) | Lakis N, Jimenez JA, Mancini-Marie A, Stip E, Lavoie ME, Mendrek A | Psychiatry research |
| 481 | [Neural Correlates of Impaired Cognitive Control over Working Memory in Schizophrenia.](https://neurosynth.org/studies/24239131/) | Eich TS, Nee DE, Insel C, Malapani C, Smith EE | Biological psychiatry |
| 482 | [Neural correlates of irony comprehension: the role of schizotypal personality traits.](https://neurosynth.org/studies/20071019/) | Rapp AM, Mutschler DE, Wild B, Erb M, Lengsfeld I, Saur R, Grodd W | Brain and language |
| 483 | [Neural correlates of metaphor processing in schizophrenia.](https://neurosynth.org/studies/17081771/) | Kircher TT, Leube DT, Erb M, Grodd W, Rapp AM | NeuroImage |
| 484 | [Neural correlates of planning performance in patients with schizophrenia - Relationship with apathy.](https://neurosynth.org/studies/25497221/) | Liemburg EJ, Dlabac-De Lange JJ, Bais L, Knegtering H, van Osch MJ, Renken RJ, Aleman A | Schizophrenia research |
| 485 | [Neural correlates of probabilistic category learning in patients with schizophrenia.](https://neurosynth.org/studies/19176832/) | Weickert TW, Goldberg TE, Callicott JH, Chen Q, Apud JA, Das S, Zoltick BJ, Egan MF, Meeter M, Myers C, Gluck MA, Weinberger DR, Mattay VS | The Journal of neuroscience : the official journal of the Society for Neuroscience |
| 486 | [Neural correlates of relational and item-specific encoding during working and long-term memory in schizophrenia.](https://neurosynth.org/studies/21907293/) | Ragland JD, Blumenfeld RS, Ramsay IS, Yonelinas A, Yoon J, Solomon M, Carter CS, Ranganath C | NeuroImage |
| 487 | [Neural correlates of reward processing in healthy siblings of patients with schizophrenia.](https://neurosynth.org/studies/26441601/) | Hanssen E, van der Velde J, Gromann PM, Shergill SS, de Haan L, Bruggeman R, Krabbendam L, Aleman A, van Atteveldt N | Frontiers in human neuroscience |
| 488 | [Neural correlates of S-ketamine induced psychosis during overt continuous verbal fluency.](https://neurosynth.org/studies/20727411/) | Nagels A, Kirner-Veselinovic A, Krach S, Kircher T | NeuroImage |
| 489 | [Neural correlates of semantic associations in patients with schizophrenia.](https://neurosynth.org/studies/23880958/) | Sass K, Heim S, Sachs O, Straube B, Schneider F, Habel U, Kircher T | European archives of psychiatry and clinical neuroscience |
| 490 | [Neural correlates of tactile prepulse inhibition: a functional MRI study in normal and schizophrenic subjects.](https://neurosynth.org/studies/12714174/) | Kumari V, Gray JA, Geyer MA, ffytche D, Soni W, Mitterschiffthaler MT, Vythelingum GN, Simmons A, Williams SC, Sharma T | Psychiatry research |
| 491 | [Neural correlates of the core facets of empathy in schizophrenia.](https://neurosynth.org/studies/22306196/) | Derntl B, Finkelmeyer A, Voss B, Eickhoff SB, Kellermann T, Schneider F, Habel U | Schizophrenia research |
| 492 | [Neural correlates of the object-recall process in semantic memory.](https://neurosynth.org/studies/16938439/) | Assaf M, Calhoun VD, Kuzu CH, Kraut MA, Rivkin PR, Hart J Jr, Pearlson GD | Psychiatry research |
| 493 | [Neural correlates of the relationship between discourse coherence and sensory monitoring in schizophrenia.](https://neurosynth.org/studies/23969195/) | Tagamets MA, Cortes CR, Griego JA, Elvevag B | Cortex; a journal devoted to the study of the nervous system and behavior |
| 494 | [Neural correlates of verbal and nonverbal working memory deficits in individuals with schizophrenia and their high-risk siblings.](https://neurosynth.org/studies/16842976/) | Brahmbhatt SB, Haut K, Csernansky JG, Barch DM | Schizophrenia research |
| 495 | [Neural correlates of working memory dysfunction in first-episode schizophrenia patients: an fMRI multi-center study.](https://neurosynth.org/studies/17010573/) | Schneider F, Habel U, Reske M, Kellermann T, Stocker T, Shah NJ, Zilles K, Braus DF, Schmitt A, Schlosser R, Wagner M, Frommann I, Kircher T, Rapp A, Meisenzahl E, Ufer S, Ruhrmann S, Thienel R, Sauer H, Henn FA, Gaebel W | Schizophrenia research |
| 496 | [Neural disruption to theory of mind predicts daily social functioning in individuals at familial high-risk for schizophrenia.](https://neurosynth.org/studies/24396009/) | Dodell-Feder D, Delisi LE, Hooker CI | Social cognitive and affective neuroscience |
| 497 | [Neural dysfunction and violence in schizophrenia: an fMRI investigation.](https://neurosynth.org/studies/16616832/) | Kumari V, Aasen I, Taylor P, Ffytche DH, Das M, Barkataki I, Goswami S, O'Connell P, Howlett M, Williams SC, Sharma T | Schizophrenia research |
| 498 | [Neural integration of speech and gesture in schizophrenia: evidence for differential processing of metaphoric gestures.](https://neurosynth.org/studies/22378493/) | Straube B, Green A, Sass K, Kirner-Veselinovic A, Kircher T | Human brain mapping |
| 499 | [Neural mechanisms of mood-induced modulation of reality monitoring in schizophrenia.](https://neurosynth.org/studies/28162778/) | Subramaniam K, Ranasinghe KG, Mathalon D, Nagarajan S, Vinogradov S | Cortex; a journal devoted to the study of the nervous system and behavior |
| 400 | [Neural mechanisms of smooth pursuit eye movements in schizotypy.](https://neurosynth.org/studies/25197013/) | Meyhofer I, Steffens M, Kasparbauer A, Grant P, Weber B, Ettinger U | Human brain mapping |
| 501 | [Neural primacy of the salience processing system in schizophrenia.](https://neurosynth.org/studies/23972602/) | Palaniyappan L, Simmonite M, White TP, Liddle EB, Liddle PF | Neuron |
| 502 | [Neural signal during immediate reward anticipation in schizophrenia: Relationship to real-world motivation and function.](https://neurosynth.org/studies/26413478/) | Subramaniam K, Hooker CI, Biagianti B, Fisher M, Nagarajan S, Vinogradov S | NeuroImage. Clinical |
| 503 | [Neural structure and social dysfunction in individuals at clinical high risk for psychosis.](https://neurosynth.org/studies/25443177/) | Lincoln SH, Hooker CI | Psychiatry research |
| 504 | [Neural substrate of unrelenting negative symptoms in schizophrenia: a longitudinal resting-state fMRI study.](https://neurosynth.org/studies/29128871/) | Li M, Deng W, Das T, Li Y, Zhao L, Ma X, Wang Y, Yu H, Li X, Meng YJ, Wang Q, Palaniyappan L, Li T | European archives of psychiatry and clinical neuroscience |
| 505 | [Neural substrates of olfactory processing in schizophrenia patients and their healthy relatives.](https://neurosynth.org/studies/17532193/) | Schneider F, Habel U, Reske M, Toni I, Falkai P, Shah NJ | Psychiatry research |
| 506 | [Neural systems for social cognition in Klinefelter syndrome (47,XXY): evidence from fMRI.](https://neurosynth.org/studies/21737434/) | van Rijn S, Swaab H, Baas D, de Haan E, Kahn RS, Aleman A | Social cognitive and affective neuroscience |
| 507 | [Neuregulin 1 ICE-single nucleotide polymorphism in first episode schizophrenia correlates with cerebral activation in fronto-temporal areas.](https://neurosynth.org/studies/18806920/) | Kircher T, Thienel R, Wagner M, Reske M, Habel U, Kellermann T, Frommann I, Schwab S, Wolwer W, von Wilmsdorf M, Braus DF, Schmitt A, Rapp A, Stocker T, Shah NJ, Henn FA, Sauer H, Gaebel W, Maier W, Schneider F | European archives of psychiatry and clinical neuroscience |
| 508 | [Neuroanatomical correlates of neurological soft signs in antipsychotic-naive schizophrenia.](https://neurosynth.org/studies/19019637/) | Venkatasubramanian G, Jayakumar PN, Gangadhar BN, Keshavan MS | Psychiatry research |
| 509 | [Neuroanatomical substrates of foresight in schizophrenia.](https://neurosynth.org/studies/18603414/) | Eack SM, George MM, Prasad KM, Keshavan MS | Schizophrenia research |
| 510 | [Neurobiological correlates of theory of mind in psychosis proneness.](https://neurosynth.org/studies/20888847/) | Modinos G, Renken R, Shamay-Tsoory SG, Ormel J, Aleman A | Neuropsychologia |
| 511 | [Neurobiology of insight deficits in schizophrenia: An fMRI study.](https://neurosynth.org/studies/25957484/) | Shad MU, Keshavan MS | Schizophrenia research |
| 512 | [Neurobiology of self-awareness in schizophrenia: an fMRI study.](https://neurosynth.org/studies/22480958/) | Shad MU, Keshavan MS, Steinberg JL, Mihalakos P, Thomas BP, Motes MA, Soares JC, Tamminga CA | Schizophrenia research |
| 513 | [Neuroimaging of semantic processing in schizophrenia: a parametric priming approach.](https://neurosynth.org/studies/19765623/) | Han SD, Wible CG | International journal of psychophysiology : official journal of the International Organization of Psychophysiology |
| 514 | [Neurological signs and morphological cerebral changes in schizophrenia: An analysis of NSS subscales in patients with first episode psychosis.](https://neurosynth.org/studies/21498055/) | Heuser M, Thomann PA, Essig M, Bachmann S, Schroder J | Psychiatry research |
| 515 | [Neurological soft signs and gray matter changes: a longitudinal analysis in first-episode schizophrenia.](https://neurosynth.org/studies/22018942/) | Kong L, Bachmann S, Thomann PA, Essig M, Schroder J | Schizophrenia research |
| 516 | [Neuronal correlates of theory of mind and empathy: a functional magnetic resonance imaging study in a nonverbal task.](https://neurosynth.org/studies/16122944/) | Vollm BA, Taylor AN, Richardson P, Corcoran R, Stirling J, McKie S, Deakin JF, Elliott R | NeuroImage |
| 517 | [Nicotine effects on anterior cingulate cortex in schizophrenia and healthy smokers as revealed by EEG-informed fMRI.](https://neurosynth.org/studies/23137805/) | Mobascher A, Warbrick T, Brinkmeyer J, Musso F, Stoecker T, Jon Shah N, Winterer G | Psychiatry research |
| 518 | [Nicotine-induced activation of caudate and anterior cingulate cortex in response to errors in schizophrenia.](https://neurosynth.org/studies/29181816/) | Moran LV, Stoeckel LE, Wang K, Caine CE, Villafuerte R, Calderon V, Baker JT, Ongur D, Janes AC, Evins AE, Pizzagalli DA | Psychopharmacology |
| 519 | [Nodal centrality of functional network in the differentiation of schizophrenia.](https://neurosynth.org/studies/26299706/) | Cheng H, Newman S, Goni J, Kent JS, Howell J, Bolbecker A, Puce A, O'Donnell BF, Hetrick WP | Schizophrenia research |
| 520 | [Non redundant functional brain connectivity in schizophrenia.](https://neurosynth.org/studies/27000096/) | Salvador R, Landin-Romero R, Anguera M, Canales-Rodriguez EJ, Radua J, Guerrero-Pedraza A, Sarro S, Maristany T, McKenna PJ, Pomarol-Clotet E | Brain imaging and behavior |
| 521 | [Nonlinear complexity analysis of brain FMRI signals in schizophrenia.](https://neurosynth.org/studies/24824731/) | Sokunbi MO, Gradin VB, Waiter GD, Cameron GG, Ahearn TS, Murray AD, Steele DJ, Staff RT | PloS one |
| 522 | [Nonlinear response of the anterior cingulate and prefrontal cortex in schizophrenia as a function of variable attentional control.](https://neurosynth.org/studies/19633177/) | Blasi G, Taurisano P, Papazacharias A, Caforio G, Romano R, Lobianco L, Fazio L, Di Giorgio A, Latorre V, Sambataro F, Popolizio T, Nardini M, Mattay VS, Weinberger DR, Bertolino A | Cerebral cortex (New York, N.Y. : 1991) |
| 523 | [Normal brain activation in schizophrenia patients during associative emotional learning.](https://neurosynth.org/studies/24148912/) | Swart M, Liemburg EJ, Kortekaas R, Wiersma D, Bruggeman R, Aleman A | Psychiatry research |
| 524 | [Novelty detection and repetition suppression in a passive picture viewing task: a possible approach for the evaluation of neuropsychiatric disorders.](https://neurosynth.org/studies/12395390/) | Jessen F, Manka C, Scheef L, Granath DO, Schild HH, Heun R | Human brain mapping |
| 525 | [Olanzapine modulation of long- and short-range functional connectivity in the resting brain in a sample of patients with schizophrenia.](https://neurosynth.org/studies/27887859/) | Guo W, Liu F, Chen J, Wu R, Li L, Zhang Z, Zhao J | European neuropsychopharmacology : the journal of the European College of Neuropsychopharmacology |
| 526 | [On the existence of a generalized non-specific task-dependent network.](https://neurosynth.org/studies/26300757/) | Hugdahl K, Raichle ME, Mitra A, Specht K | Frontiers in human neuroscience |
| 527 | [Opposite effects of catechol-O-methyltransferase Val158Met on cortical function in healthy subjects and patients with schizophrenia.](https://neurosynth.org/studies/19054502/) | Prata DP, Mechelli A, Fu CH, Picchioni M, Kane F, Kalidindi S, McDonald C, Howes O, Kravariti E, Demjaha A, Toulopoulou T, Diforti M, Murray RM, Collier DA, McGuire PK | Biological psychiatry |
| 528 | [Oppositional COMT Val158Met effects on resting state functional connectivity in adolescents and adults.](https://neurosynth.org/studies/25319752/) | Meyer BM, Huemer J, Rabl U, Boubela RN, Kalcher K, Berger A, Banaschewski T, Barker G, Bokde A, Buchel C, Conrod P, Desrivieres S, Flor H, Frouin V, Gallinat J, Garavan H, Heinz A, Ittermann B, Jia T, Lathrop M, Martinot JL, Nees F, Rietschel M, Smolka MN, Bartova L, Popovic A, Scharinger C, Sitte HH, Steiner H, Friedrich MH, Kasper S, Perkmann T, Praschak-Rieder N, Haslacher H, Esterbauer H, Moser E, Schumann G, Pezawas L | Brain structure & function |
| 529 | [Optimal transcranial magnetic stimulation coil placement for targeting the dorsolateral prefrontal cortex using novel magnetic resonance image-guided neuronavigation.](https://neurosynth.org/studies/20162598/) | Rusjan PM, Barr MS, Farzan F, Arenovich T, Maller JJ, Fitzgerald PB, Daskalakis ZJ | Human brain mapping |
| 530 | [Optimally-Discriminative Voxel-Based Morphometry significantly increases the ability to detect group differences in schizophrenia, mild cognitive impairment, and Alzheimer's disease.](https://neurosynth.org/studies/23631985/) | Zhang T, Davatzikos C | NeuroImage |
| 531 | [Origins of spatial working memory deficits in schizophrenia: an event-related FMRI and near-infrared spectroscopy study.](https://neurosynth.org/studies/18335036/) | Lee J, Folley BS, Gore J, Park S | PloS one |
| 532 | [Out of touch with reality? Social perception in first-episode schizophrenia.](https://neurosynth.org/studies/22275166/) | Ebisch SJ, Salone A, Ferri F, De Berardis D, Romani GL, Ferro FM, Gallese V | Social cognitive and affective neuroscience |
| 533 | [Overactivation of fear systems to neutral faces in schizophrenia.](https://neurosynth.org/studies/18295746/) | Hall J, Whalley HC, McKirdy JW, Romaniuk L, McGonigle D, McIntosh AM, Baig BJ, Gountouna VE, Job DE, Donaldson DI, Sprengelmeyer R, Young AW, Johnstone EC, Lawrie SM | Biological psychiatry |
| 534 | [Overlapping clusters of gray matter deficits in paranoid schizophrenia and psychotic bipolar mania with family history.](https://neurosynth.org/studies/21138758/) | Cui L, Li M, Deng W, Guo W, Ma X, Huang C, Jiang L, Wang Y, Collier DA, Gong Q, Li T | Neuroscience letters |
| 535 | [Paracingulate sulcus morphology and fMRI activation detection in schizophrenia patients.](https://neurosynth.org/studies/16387476/) | Artiges E, Martelli C, Naccache L, Bartres-Faz D, Leprovost JB, Viard A, Paillere-Martinot ML, Dehaene S, Martinot JL | Schizophrenia research |
| 536 | [Parietal cortex and episodic memory retrieval in schizophrenia.](https://neurosynth.org/studies/20488673/) | Lepage M, Pelletier M, Achim A, Montoya A, Menear M, Lal S | Psychiatry research |
| 537 | [Parietal dysfunction is associated with increased outcome-related decision-making in schizophrenia patients.](https://neurosynth.org/studies/12062884/) | Paulus MP, Hozack NE, Zauscher BE, Frank L, Brown GG, McDowell J, Braff DL | Biological psychiatry |
| 538 | [Partial support for ZNF804A genotype-dependent alterations in prefrontal connectivity.](https://neurosynth.org/studies/22042765/) | Paulus FM, Krach S, Bedenbender J, Pyka M, Sommer J, Krug A, Knake S, Nothen MM, Witt SH, Rietschel M, Kircher T, Jansen A | Human brain mapping |
| 539 | [Patients with Schizophrenia Fail to Up-Regulate Task-Positive and Down-Regulate Task-Negative Brain Networks: An fMRI Study Using an ICA Analysis Approach.](https://neurosynth.org/studies/22666197/) | Nygard M, Eichele T, Loberg EM, Jorgensen HA, Johnsen E, Kroken RA, Berle JO, Hugdahl K | Frontiers in human neuroscience |
| 540 | [Pavlovian reward prediction and receipt in schizophrenia: relationship to anhedonia.](https://neurosynth.org/studies/22574121/) | Dowd EC, Barch DM | PloS one |
| 541 | [Perceived patient-parent relationships and neural representation of parents in schizophrenia.](https://neurosynth.org/studies/22678652/) | Choi SH, Lee SH, Park HJ, Chun JW, Kang JI, Kim JJ | European archives of psychiatry and clinical neuroscience |
| 542 | [Perceptual organization and visual search processes during target detection task performance in schizophrenia, as revealed by fMRI.](https://neurosynth.org/studies/20678981/) | Silverstein SM, Berten S, Essex B, All SD, Kasi R, Little DM | Neuropsychologia |
| 543 | [Physiological dysfunction of the dorsolateral prefrontal cortex in schizophrenia revisited.](https://neurosynth.org/studies/11053229/) | Callicott JH, Bertolino A, Mattay VS, Langheim FJ, Duyn J, Coppola R, Goldberg TE, Weinberger DR | Cerebral cortex (New York, N.Y. : 1991) |
| 544 | [Plasticity of prefrontal cortex connectivity in schizophrenia in response to antisaccade practice.](https://neurosynth.org/studies/27955939/) | Rodrigue AL, Austin BP, McDowell JE | Psychiatry research. Neuroimaging |
| 545 | [Polygenic risk for five psychiatric disorders and cross-disorder and disorder-specific neural connectivity in two independent populations.](https://neurosynth.org/studies/28275544/) | Wang T, Zhang X, Li A, Zhu M, Liu S, Qin W, Li J, Yu C, Jiang T, Liu B | NeuroImage. Clinical |
| 546 | [Polygenic risk for schizophrenia affects working memory and its neural correlates in healthy subjects.](https://neurosynth.org/studies/29409757/) | Krug A, Dietsche B, Zollner R, Yuksel D, Nothen MM, Forstner AJ, Rietschel M, Dannlowski U, Baune BT, Maier R, Witt SH, Kircher T | Schizophrenia research |
| 547 | [Prefrontal cortex connectivity dysfunction in performing the Fist-Edge-Palm task in patients with first-episode schizophrenia and non-psychotic first-degree relatives.](https://neurosynth.org/studies/26594623/) | Chan RC, Huang J, Zhao Q, Wang Y, Lai YY, Hong N, Shum DH, Cheung EF, Yu X, Dazzan P | NeuroImage. Clinical |
| 548 | [Prefrontal cortex function in nonpsychotic siblings of individuals with schizophrenia.](https://neurosynth.org/studies/17631280/) | Delawalla Z, Csernansky JG, Barch DM | Biological psychiatry |
| 549 | [Prefrontal cortical dysfunction during visual perspective-taking in schizophrenia.](https://neurosynth.org/studies/24055199/) | Eack SM, Wojtalik JA, Newhill CE, Keshavan MS, Phillips ML | Schizophrenia research |
| 550 | [Prefrontal cortical thickness in first-episode psychosis: a magnetic resonance imaging study.](https://neurosynth.org/studies/14732592/) | Wiegand LC, Warfield SK, Levitt JJ, Hirayasu Y, Salisbury DF, Heckers S, Dickey CC, Kikinis R, Jolesz FA, McCarley RW, Shenton ME | Biological psychiatry |
| 551 | [Prefrontal functioning during context processing in schizophrenia and major depression: an event-related fMRI study.](https://neurosynth.org/studies/15949653/) | Holmes AJ, MacDonald A 3rd, Carter CS, Barch DM, Andrew Stenger V, Cohen JD | Schizophrenia research |
| 552 | [Prefrontal hyperactivation during a working memory task in early-onset schizophrenia spectrum disorders: an fMRI study.](https://neurosynth.org/studies/22079661/) | Thormodsen R, Jensen J, Holmen A, Juuhl-Langseth M, Emblem KE, Andreassen OA, Rund BR | Psychiatry research |
| 553 | [Prefrontal lobe dysfunction predicts treatment response in medication-naive first-episode schizophrenia.](https://neurosynth.org/studies/21497488/) | van Veelen NM, Vink M, Ramsey NF, van Buuren M, Hoogendam JM, Kahn RS | Schizophrenia research |
| 554 | [Prefrontal-posterior parietal networks in schizophrenia: primary dysfunctions and secondary compensations.](https://neurosynth.org/studies/12513941/) | Quintana J, Wong T, Ortiz-Portillo E, Kovalik E, Davidson T, Marder SR, Mazziotta JC | Biological psychiatry |
| 555 | [Prefrontal-temporal gray matter deficits in bipolar disorder patients with persecutory delusions.](https://neurosynth.org/studies/19419772/) | Tost H, Ruf M, Schmal C, Schulze TG, Knorr C, Vollmert C, Bosshenz K, Ende G, Meyer-Lindenberg A, Henn FA, Rietschel M | Journal of affective disorders |
| 556 | [Preservation and compensation: the functional neuroanatomy of insight and working memory in schizophrenia.](https://neurosynth.org/studies/24332795/) | Sapara A, Ffytche DH, Birchwood M, Cooke MA, Fannon D, Williams SC, Kuipers E, Kumari V | Schizophrenia research |
| 557 | [Procedural learning in schizophrenia investigated with functional magnetic resonance imaging.](https://neurosynth.org/studies/16945506/) | Zedkova L, Woodward ND, Harding I, Tibbo PG, Purdon SE | Schizophrenia research |
| 558 | [Procedural learning in schizophrenia: a functional magnetic resonance imaging investigation.](https://neurosynth.org/studies/12165380/) | Kumari V, Gray JA, Honey GD, Soni W, Bullmore ET, Williams SC, Ng VW, Vythelingum GN, Simmons A, Suckling J, Corr PJ, Sharma T | Schizophrenia research |
| 559 | [Progressive grey matter atrophy over the first 2-3 years of illness in first-episode schizophrenia: a tensor-based morphometry study.](https://neurosynth.org/studies/16677830/) | Whitford TJ, Grieve SM, Farrow TF, Gomes L, Brennan J, Harris AW, Gordon E, Williams LM | NeuroImage |
| 560 | [Progressive temporal lobe grey matter loss in adolescents with schizotypal traits and mild intellectual impairment.](https://neurosynth.org/studies/19833484/) | Moorhead TW, Stanfield A, Spencer M, Hall J, McIntosh A, Owens DC, Lawrie S, Johnstone E | Psychiatry research |
| 561 | [Pseudo-continuous arterial spin labeling MRI study of schizophrenic patients.](https://neurosynth.org/studies/24581548/) | Ota M, Ishikawa M, Sato N, Okazaki M, Maikusa N, Hori H, Hattori K, Teraishi T, Ito K, Kunugi H | Schizophrenia research |
| 562 | [Psychopathy and functional magnetic resonance imaging blood oxygenation level-dependent responses to emotional faces in violent patients with schizophrenia.](https://neurosynth.org/studies/19446795/) | Dolan MC, Fullam RS | Biological psychiatry |
| 563 | [Psychosis-proneness and neural correlates of self-inhibition in theory of mind.](https://neurosynth.org/studies/23874445/) | van der Meer L, Groenewold NA, Pijnenborg M, Aleman A | PloS one |
| 564 | [Putamen-related regional and network functional deficits in first-episode schizophrenia with auditory verbal hallucinations.](https://neurosynth.org/studies/26995674/) | Cui LB, Liu K, Li C, Wang LX, Guo F, Tian P, Wu YJ, Guo L, Liu WM, Xi YB, Wang HN, Yin H | Schizophrenia research |
| 565 | [Re-evaluating dorsolateral prefrontal cortex activation during working memory in schizophrenia.](https://neurosynth.org/studies/19196494/) | Karlsgodt KH, Sanz J, van Erp TG, Bearden CE, Nuechterlein KH, Cannon TD | Schizophrenia research |
| 566 | [Reading impairment in schizophrenia: dysconnectivity within the visual system.](https://neurosynth.org/studies/24144956/) | Vinckier F, Cohen L, Oppenheim C, Salvador A, Picard H, Amado I, Krebs MO, Gaillard R | Neuropsychologia |
| 567 | [Real-time neural activity and connectivity in healthy individuals and schizophrenia patients.](https://neurosynth.org/studies/15488397/) | Ioannides AA, Poghosyan V, Dammers J, Streit M | NeuroImage |
| 568 | [Reduced activation of superior temporal gyrus during auditory comprehension in young offspring of patients with schizophrenia.](https://neurosynth.org/studies/21684722/) | Rajarethinam R, Venkatesh BK, Peethala R, Phan KL, Keshavan M | Schizophrenia research |
| 569 | [Reduced anterior cingulate gyrus volume correlates with executive dysfunction in men with first-episode schizophrenia.](https://neurosynth.org/studies/10858628/) | Szeszko PR, Bilder RM, Lencz T, Ashtari M, Goldman RS, Reiter G, Wu H, Lieberman JA | Schizophrenia research |
| 570 | [Reduced cortical thickness in first episode schizophrenia.](https://neurosynth.org/studies/19926451/) | Schultz CC, Koch K, Wagner G, Roebel M, Schachtzabel C, Gaser C, Nenadic I, Reichenbach JR, Sauer H, Schlosser RG | Schizophrenia research |
| 571 | [Reduced default mode network connectivity in schizophrenia patients.](https://neurosynth.org/studies/25892719/) | Pankow A, Deserno L, Walter M, Fydrich T, Bermpohl F, Schlagenhauf F, Heinz A | Schizophrenia research |
| 572 | [Reduced dorsal prefrontal gray matter after chronic ketamine use.](https://neurosynth.org/studies/21035788/) | Liao Y, Tang J, Corlett PR, Wang X, Yang M, Chen H, Liu T, Chen X, Hao W, Fletcher PC | Biological psychiatry |
| 573 | [Reduced error-related activation in two anterior cingulate circuits is related to impaired performance in schizophrenia.](https://neurosynth.org/studies/18158315/) | Polli FE, Barton JJ, Thakkar KN, Greve DN, Goff DC, Rauch SL, Manoach DS | Brain : a journal of neurology |
| 574 | [Reduced fronto-temporal connectivity is associated with frontal gray matter density reduction and neuropsychological deficit in schizophrenia.](https://neurosynth.org/studies/19097861/) | Spoletini I, Cherubini A, Di Paola M, Banfi G, Rusch N, Martinotti G, Bria P, Rubino IA, Siracusano A, Caltagirone C, Spalletta G | Schizophrenia research |
| 575 | [Reduced functional connectivity and asymmetry of the planum temporale in patients with schizophrenia and first-degree relatives.](https://neurosynth.org/studies/23672819/) | Oertel-Knochel V, Knochel C, Matura S, Prvulovic D, Linden DE, van de Ven V | Schizophrenia research |
| 576 | [Reduced functional connectivity during controlled semantic integration in schizophrenia: A multivariate approach.](https://neurosynth.org/studies/26014890/) | Woodward TS, Tipper CM, Leung A, Lavigne KM, Sanford N, Metzak PD | Human brain mapping |
| 577 | [Reduced functional connectivity in a right-hemisphere network for volitional ocular motor control in schizophrenia.](https://neurosynth.org/studies/20159769/) | Tu P, Buckner RL, Zollei L, Dyckman KA, Goff DC, Manoach DS | Brain : a journal of neurology |
| 578 | [Reduced intrinsic visual cortical connectivity is associated with impaired perceptual closure in schizophrenia.](https://neurosynth.org/studies/28480163/) | van de Ven V, Rotarska Jagiela A, Oertel-Knochel V, Linden DEJ | NeuroImage. Clinical |
| 579 | [Reduced language lateralization in first-episode schizophrenia: an fMRI index of functional asymmetry.](https://neurosynth.org/studies/19185468/) | Bleich-Cohen M, Hendler T, Kotler M, Strous RD | Psychiatry research |
| 580 | [Reduced laterality as a trait marker of schizophrenia--evidence from structural and functional neuroimaging.](https://neurosynth.org/studies/20147555/) | Oertel V, Knochel C, Rotarska-Jagiela A, Schonmeyer R, Lindner M, van de Ven V, Haenschel C, Uhlhaas P, Maurer K, Linden DE | The Journal of neuroscience : the official journal of the Society for Neuroscience |
| 581 | [Reduced microstructural integrity of the white matter underlying anterior cingulate cortex is associated with increased saccadic latency in schizophrenia.](https://neurosynth.org/studies/17590354/) | Manoach DS, Ketwaroo GA, Polli FE, Thakkar KN, Barton JJ, Goff DC, Fischl B, Vangel M, Tuch DS | NeuroImage |
| 582 | [Reduced neuro-integration from the dorsolateral prefrontal cortex to the whole brain and executive dysfunction in schizophrenia patients and their relatives.](https://neurosynth.org/studies/23726722/) | Su TW, Lan TH, Hsu TW, Biswal BB, Tsai PJ, Lin WC, Lin CP | Schizophrenia research |
| 583 | [Reduced neuronal activity in language-related regions after transcranial magnetic stimulation therapy for auditory verbal hallucinations.](https://neurosynth.org/studies/22840762/) | Kindler J, Homan P, Jann K, Federspiel A, Flury R, Hauf M, Strik W, Dierks T, Hubl D | Biological psychiatry |
| 584 | [Reduced prefrontal functional connectivity in the default mode network is related to greater psychopathology in subjects with high genetic loading for schizophrenia.](https://neurosynth.org/studies/21277171/) | Jang JH, Jung WH, Choi JS, Choi CH, Kang DH, Shin NY, Hong KS, Kwon JS | Schizophrenia research |
| 585 | [Reduced prefrontal-parietal effective connectivity and working memory deficits in schizophrenia.](https://neurosynth.org/studies/22219266/) | Deserno L, Sterzer P, Wustenberg T, Heinz A, Schlagenhauf F | The Journal of neuroscience : the official journal of the Society for Neuroscience |
| 586 | [Reduced striatal activation during reward anticipation due to appetite-provoking cues in chronic schizophrenia: a fMRI study.](https://neurosynth.org/studies/22209236/) | Grimm O, Vollstadt-Klein S, Krebs L, Zink M, Smolka MN | Schizophrenia research |
| 587 | [Reduced structural integrity and functional lateralization of the dorsal language pathway correlate with hallucinations in schizophrenia: A combined diffusion spectrum imaging and functional magnetic](https://neurosynth.org/studies/25241043/) | Wu CH, Hwang TJ, Chen PJ, Chou TL, Hsu YC, Liu CM, Wang HL, Chen CM, Hua MS, Hwu HG, Isaac Tseng WY | Psychiatry research |
| 588 | [Reduced task-related suppression during semantic repetition priming in schizophrenia.](https://neurosynth.org/studies/20083395/) | Jeong B, Kubicki M | Psychiatry research |
| 589 | [Regional brain activity during early visual perception in unaffected siblings of schizophrenia patients.](https://neurosynth.org/studies/20494338/) | Lee J, Cohen MS, Engel SA, Glahn D, Nuechterlein KH, Wynn JK, Green MF | Biological psychiatry |
| 590 | [Regional brain atrophy and functional disconnection in Broca's area in individuals at ultra-high risk for psychosis and schizophrenia.](https://neurosynth.org/studies/23251669/) | Jung WH, Jang JH, Shin NY, Kim SN, Choi CH, An SK, Kwon JS | PloS one |
| 591 | [Regional contraction of brain surface area involves three large-scale networks in schizophrenia.](https://neurosynth.org/studies/21497489/) | Palaniyappan L, Mallikarjun P, Joseph V, White TP, Liddle PF | Schizophrenia research |
| 592 | [Remembering verbally-presented items as pictures: Brain activity underlying visual mental images in schizophrenia patients with visual hallucinations.](https://neurosynth.org/studies/28746902/) | Stephan-Otto C, Siddi S, Senior C, Cuevas-Esteban J, Cambra-Marti MR, Ochoa S, Brebion G | Cortex; a journal devoted to the study of the nervous system and behavior |
| 593 | [Resting state and task-induced deactivation: A methodological comparison in patients with schizophrenia and healthy controls.](https://neurosynth.org/studies/19777578/) | Mannell MV, Franco AR, Calhoun VD, Canive JM, Thoma RJ, Mayer AR | Human brain mapping |
| 594 | [Resting state cerebral blood flow and objective motor activity reveal basal ganglia dysfunction in schizophrenia.](https://neurosynth.org/studies/21511443/) | Walther S, Federspiel A, Horn H, Razavi N, Wiest R, Dierks T, Strik W, Muller TJ | Psychiatry research |
| 595 | [Resting-state fMRI mapping of cerebellar functional dysconnections involving multiple large-scale networks in patients with schizophrenia.](https://neurosynth.org/studies/23810119/) | Chen YL, Tu PC, Lee YC, Chen YS, Li CT, Su TP | Schizophrenia research |
| 596 | [Resting-state functional connectivity alterations in the default network of schizophrenia patients with persistent auditory verbal hallucinations.](https://neurosynth.org/studies/25468173/) | Alonso-Solis A, Vives-Gilabert Y, Grasa E, Portella MJ, Rabella M, Sauras RB, Roldan A, Nunez-Marin F, Gomez-Anson B, Perez V, Alvarez E, Corripio I | Schizophrenia research |
| 597 | [Resting-state functional connectivity between right anterior insula and right orbital frontal cortex correlate with insight level in obsessive-compulsive disorder.](https://neurosynth.org/studies/28458998/) | Fan J, Zhong M, Zhu X, Gan J, Liu W, Niu C, Liao H, Zhang H, Yi J, Tan C | NeuroImage. Clinical |
| 598 | [Resting-state functional connectivity in medication-naive schizophrenia patients with and without auditory verbal hallucinations: A preliminary report.](https://neurosynth.org/studies/28130005/) | Chang X, Collin G, Xi Y, Cui L, Scholtens LH, Sommer IE, Wang H, Yin H, Kahn RS, van den Heuvel MP | Schizophrenia research |
| 599 | [Resting-State Time-Varying Analysis Reveals Aberrant Variations of Functional Connectivity in Autism.](https://neurosynth.org/studies/27695408/) | Yao Z, Hu B, Xie Y, Zheng F, Liu G, Chen X, Zheng W | Frontiers in human neuroscience |
| 500 | [Retrosplenial cortex connectivity in schizophrenia.](https://neurosynth.org/studies/19783410/) | Bluhm RL, Miller J, Lanius RA, Osuch EA, Boksman K, Neufeld RW, Theberge J, Schaefer B, Williamson PC | Psychiatry research |
| 601 | [Reversed cerebellar asymmetry in men with first-episode schizophrenia.](https://neurosynth.org/studies/12614998/) | Szeszko PR, Gunning-Dixon F, Ashtari M, Snyder PJ, Lieberman JA, Bilder RM | Biological psychiatry |
| 602 | [Right lateral fusiform gyrus dysfunction during facial information processing in schizophrenia.](https://neurosynth.org/studies/12814861/) | Quintana J, Wong T, Ortiz-Portillo E, Marder SR, Mazziotta JC | Biological psychiatry |
| 603 | [Rostral anterior cingulate cortex dysfunction during error processing in schizophrenia.](https://neurosynth.org/studies/12566282/) | Laurens KR, Ngan ET, Bates AT, Kiehl KA, Liddle PF | Brain : a journal of neurology |
| 604 | [RT distributional analysis of cognitive-control-related brain activity in first-episode schizophrenia.](https://neurosynth.org/studies/24615691/) | Fassbender C, Scangos K, Lesh TA, Carter CS | Cognitive, affective & behavioral neuroscience |
| 605 | [Salience network-midbrain dysconnectivity and blunted reward signals in schizophrenia.](https://neurosynth.org/studies/23146249/) | Gradin VB, Waiter G, O'Connor A, Romaniuk L, Stickle C, Matthews K, Hall J, Douglas Steele J | Psychiatry research |
| 606 | [Schizophrenia and the brain's control network: aberrant within- and between-network connectivity of the frontoparietal network in schizophrenia.](https://neurosynth.org/studies/23706416/) | Tu PC, Lee YC, Chen YS, Li CT, Su TP | Schizophrenia research |
| 607 | [Schizophrenia as a network disease: disruption of emergent brain function in patients with auditory hallucinations.](https://neurosynth.org/studies/23349665/) | Rish I, Cecchi G, Thyreau B, Thirion B, Plaze M, Paillere-Martinot ML, Martelli C, Martinot JL, Poline JB | PloS one |
| 608 | [Schizophrenia patients and their healthy siblings share disruption of white matter integrity in the left prefrontal cortex and the hippocampus but not the anterior cingulate cortex.](https://neurosynth.org/studies/19643580/) | Hao Y, Yan Q, Liu H, Xu L, Xue Z, Song X, Kaneko Y, Jiang T, Liu Z, Shan B | Schizophrenia research |
| 609 | [Searching for a structural endophenotype in psychosis using computational morphometry.](https://neurosynth.org/studies/12694890/) | Marcelis M, Suckling J, Woodruff P, Hofman P, Bullmore E, van Os J | Psychiatry research |
| 610 | [Seeking Optimal Region-Of-Interest (ROI) Single-Value Summary Measures for fMRI Studies in Imaging Genetics.](https://neurosynth.org/studies/26974435/) | Tong Y, Chen Q, Nichols TE, Rasetti R, Callicott JH, Berman KF, Weinberger DR, Mattay VS | PloS one |
[truncated: 29,027 more chars]
